# Genetic determinants of anti-malarial acquired immunity in a large multi-centre study

**DOI:** 10.1186/s12936-015-0833-x

**Published:** 2015-08-28

**Authors:** Jennifer M G Shelton, Patrick Corran, Paul Risley, Nilupa Silva, Christina Hubbart, Anna Jeffreys, Kate Rowlands, Rachel Craik, Victoria Cornelius, Meike Hensmann, Sile Molloy, Nuno Sepulveda, Taane G Clark, Gavin Band, Geraldine M Clarke, Christopher C A Spencer, Angeliki Kerasidou, Susana Campino, Sarah Auburn, Adama Tall, Alioune Badara Ly, Odile Mercereau-Puijalon, Anavaj Sakuntabhai, Abdoulaye Djimdé, Boubacar Maiga, Ousmane Touré, Ogobara K Doumbo, Amagana Dolo, Marita Troye-Blomberg, Valentina D Mangano, Frederica Verra, David Modiano, Edith Bougouma, Sodiomon B Sirima, Muntaser Ibrahim, Ayman Hussain, Nahid Eid, Abier Elzein, Hiba Mohammed, Ahmed Elhassan, Ibrahim Elhassan, Thomas N Williams, Carolyne Ndila, Alexander Macharia, Kevin Marsh, Alphaxard Manjurano, Hugh Reyburn, Martha Lemnge, Deus Ishengoma, Richard Carter, Nadira Karunaweera, Deepika Fernando, Rajika Dewasurendra, Christopher J Drakeley, Eleanor M Riley, Dominic P Kwiatkowski, Kirk A Rockett, In collaboration with the MalariaGEN Consortium

**Affiliations:** Wellcome Trust Centre for Human Genetics, University of Oxford, Roosevelt Drive, Oxford, UK; London School of Hygiene and Tropical Medicine, Keppel Street, London, UK; National Institute for Biological Standards and Controls, South Mimms, Hertfordshire, UK; Wellcome Trust Sanger Institute, Wellcome Trust Genome Campus, Hinxton, Cambridge, CB10 1SA UK; Nuffield Department of Population Health, The Ethox Centre, University of Oxford, Richard Doll Building, Old Road Campus, Oxford, OX3 7LF UK; Infectious Diseases Epidemiology Unit, Institut Pasteur, BP 220, Dakar, Senegal; Parasite Molecular Immunology Unit, Institut Pasteur, 28 rue du Docteur Roux, 75724 Paris Cedex 15, France; Unité de Génétique Fonctionnelle des Maladies Infectieuses, Institut Pasteur, 28 rue du Docteur Roux, 75724 Paris Cedex 15, France; Centre National de la Recherche Scientifique, URA3012, 28 rue du Docteur Roux, 75724 Paris Cedex 15, France; Department of Epidemiology of Parasitic Diseases, Faculty of Medicine, Pharmacy and Odonto-Stomatology, Malaria Research and Training Center, USTTB, BP 1805, Bamako, Mali; Department of Molecular Biosciences, Wenner-Gren Institute, Stockholm University, Svante Arrheniusväg 20B, 106 91 Stockholm, Sweden; Department of Public Health and Infectious Diseases, Sapienza University of Rome, Piazzale Aldo Moro 5, 00185 Rome, Italy; Centre de Recherche et de Formation sur le Paludisme, Ouagadougou, Burkina Faso; Institute of Endemic Diseases, University of Khartoum, Medical Sciences Campus, Qasser Street, Khartoum, Sudan; KEMRI-Wellcome Trust Research Programme, CGMRC, PO Box 230-80108, Kilifi, Kenya; Department of Medicine, Imperial College, St Mary’s Campus, Norfolk Place, London, W2 1PG UK; Kilimanjaro Christian Medical College, Tumaini University, Moshi, Tanzania; National Institute for Medical Research, Ocean Road, Dar es Salaam, Tanzania; Division of Biological Sciences, Ashworth Laboratories, University of Edinburgh, West Mains Rd., Edinburgh, EH9 3JT UK; Department of Parasitology, Faculty of Medicine, University of Colombo, Kynsey Road, Colombo, Sri Lanka; http://www.malariagen.net/projects/host/consortium-members

**Keywords:** Malaria, Antibody, Sickle cell trait, HbAS, CD36, Genotype

## Abstract

**Background:**

Many studies report associations between human genetic factors and immunity to malaria but few have been reliably replicated. These studies are usually country-specific, use small sample sizes and are not directly comparable due to differences in methodologies. This study brings together samples and data collected from multiple sites across Africa and Asia to use standardized methods to look for consistent genetic effects on anti-malarial antibody levels.

**Methods:**

Sera, DNA samples and clinical data were collected from 13,299 individuals from ten sites in Senegal, Mali, Burkina Faso, Sudan, Kenya, Tanzania, and Sri Lanka using standardized methods. DNA was extracted and typed for 202 Single Nucleotide Polymorphisms with known associations to malaria or antibody production, and antibody levels to four clinical grade malarial antigens [AMA1, MSP1, MSP2, and (NANP)_4_] plus total IgE were measured by ELISA techniques. Regression models were used to investigate the associations of clinical and genetic factors with antibody levels.

**Results:**

Malaria infection increased levels of antibodies to malaria antigens and, as expected, stable predictors of anti-malarial antibody levels included age, seasonality, location, and ethnicity. Correlations between antibodies to blood-stage antigens AMA1, MSP1 and MSP2 were higher between themselves than with antibodies to the (NANP)_4_ epitope of the pre-erythrocytic circumsporozoite protein, while there was little or no correlation with total IgE levels. Individuals with sickle cell trait had significantly lower antibody levels to all blood-stage antigens, and recessive homozygotes for CD36 (rs321198) had significantly lower anti-malarial antibody levels to MSP2.

**Conclusion:**

Although the most significant finding with a consistent effect across sites was for sickle cell trait, its effect is likely to be via reducing a microscopically positive parasitaemia rather than directly on antibody levels. However, this study does demonstrate a framework for the feasibility of combining data from sites with heterogeneous malaria transmission levels across Africa and Asia with which to explore genetic effects on anti-malarial immunity.

**Electronic supplementary material:**

The online version of this article (doi:10.1186/s12936-015-0833-x) contains supplementary material, which is available to authorized users.

## Background

Despite many years of research and intervention, malaria remains a major global health concern. Approximately half of the world’s population is at risk, and in 2013 there were 207 million estimated cases of malaria and 627,000 deaths [1]. Malaria has been described as the strongest evolutionary force in the recent history of the human genome (reviewed in [2]) and exerts a selective pressure that has resulted in a number of genetic adaptations. These adaptations predominantly cause erythrocyte defects, which affect the binding, invasion and growth of malaria parasites; however, there is also evidence to suggest that genetic factors influencing humoral immune responses may account for differences in susceptibility to malaria [3].

A number of studies have investigated the heritability of antibody responses against specific malaria antigens. For example, Sjoberg et al. [4] found that antibody response to ring-infected erythrocyte surface antigen (RESA) is more concordant between monozygotic twins than either dizygotic twins or age- and sex-matched siblings in Liberia and Madagascar. In Burkina Faso sibling correlations were observed for IgG and IgG subclass responses to RESA, merozoite surface protein 1 (MSP1), MSP2 and *Plasmodium falciparum* extract, whilst parent-offspring correlations were observed for IgG responses to MSP2 [5]. A study in Papua New Guinea found substantial heritability for IgG subclass responses to RESA and MSP2 and showed that this genetic variation was not dominated by a single major gene, suggesting multifactorial inheritance for IgG responses to malaria antigens [6–8].

Genetic variability in host immune response genes may account for differences in susceptibility to malaria between sympatric ethnic groups. For example, Luoni et al. [9] found in Mali that the *IL4*-524 T allele, associated with immunoglobulin (Ig) switching from IgG/IgM to IgE, is significantly more common in Fulani than in Mossi or Rimaibe and is associated with significantly higher IgG levels to CSP and Pf332 malaria antigens [9]. Also in Mali, differences in IgG sub-class levels have been seen between the Fulani and Dogon [10, 11] for the immunoglobulin receptor *FcRIIa* 131 (R/H) and the *IL4*-590 polymorphisms.

Many such immune-epidemiological studies are country-specific and have involved relatively small sample sizes providing different lines of evidence that have not always been independently replicated. To address this problem a large multi-centre study was undertaken to explore the associations between host genetic factors and their immune responses to malaria antigens at ten locations in Senegal, Mali (2 sites), Burkina Faso, Sudan, Kenya, Tanzania (3 sites), and Sri Lanka. Each site provided serum, DNA and clinical data. Standardized methods were used to genotype 202 polymorphisms (with previous malaria or antibody associations) and four IgG antibody levels to the malaria antigens AMA1, MSP1, MSP2, (NANP)_4_, plus total IgE levels. Despite the challenges of combining data from these heterogeneous study designs and backgrounds, a resource of 13,299 individuals was created to look for consistent genetic effects on anti-malarial antibodies.

## Methods

### Ethical approval

Ethical approval was granted separately for each study by their respective Ethics Review Committee (see Additional file 1) Ethical approval was also granted to MalariaGEN by the Oxford Tropical Research Ethics Committee. Informed consent was obtained from all participants before data or samples were collected.

### Study designs

Studies were conducted at ten sites in seven countries across West Africa, East Africa and Asia; details of study design at individual sites and local epidemiological conditions including malaria endemicity are described elsewhere (see [12–18]). Each site provided a DNA sample, serum sample and clinical data for participants collected at a single time point; at several sites these were provided as part of an ongoing study, while at others these were provided from a new study or as an extension to an earlier study. The clinical parameters measured at each site were influenced by study design, which varied considerably (Additional files 1 and 2). Sites either collected cross-sectional data only, longitudinal data only or a combination of both.

### Clinical data collection

At the outset investigators selected a core set of clinical parameters to be collected by all sites, taking into account variations in study design and implementation, and agreed on methods for recording these clinical measurements to encourage uniformity across the data. These clinical parameters were chosen based on their previous association with malaria infection or anti-malarial immune responses (Additional file 2). Data were submitted to the MalariaGEN resource centre where it was uploaded into a secure central database (no personal information was stored by MalariaGEN or used in any analyses). Once uploaded the data were converted to standardized units, formatted and combined with clinical data from other sites to create a full normalized and consistent dataset.

### Sample collection

Blood samples were collected from participants by venipuncture into plain tubes for serum separation or EDTA-coated tubes for DNA extraction. The volume of blood collected varied from <1 ml to 10 ml depending on the clinical settings and ethical permissions of the individual sites. Clinical data were also collected from each participant at the time of sampling and each participant was assigned a unique identification code to allow their samples and clinical records to be matched for statistical analysis.

### DNA extraction and genotyping

DNA extraction was performed at each site using the local method of choice; either Nucleon™ BACC Genomic Extraction Kits (GE Lifesciences, [19]) or Qiagen DNeasy Blood Kits (Qiagen, Crawley, UK, [20]), and DNA samples were then shipped frozen to the MalariaGEN resource centre in Oxford. Sequenom iPLEX Gold (Agena Bioscience, CA, USA) was chosen for genotyping because of its high-throughput capacity, flexibility for assay design and ability to genotype up to 40 SNPs (Single Nucloetide Polymorphisms) in one reaction. Multiplexes were designed using MassARRAY^®^ Assay Design software (Agena Bioscience) and assays with poor performance or concordance were removed from the multiplex during testing. A total of 202 SNP-assays were designed. These included a set of 65 that have been analysed in a severe malaria case–control study across 11 countries [21] plus a further 137 autosomal SNPs selected in genes associated with or described as playing a role in malaria and antibody production (Additional file 3 contains details of the gene regions and SNPs assay multiplexes; Additional file 4 contains further details of the genotyping methodology).

### Measurement of immune responses

All study sites supplied serum or plasma samples to a central repository established at the National Institute for Biological Standards and Control (NIBSC). Samples were divided into two aliquots and stored at −80 °C in individually barcoded tubes (Matrix systems, Thermo Fisher Scientifics, Horsham, UK) and racks. Before storage, a 5-µl aliquot was removed and diluted to 50 µl in PBS-0.2 % (w/v) sodium azide in a storage plate in order to characterize each sample. This characterization took the form of assaying specific antibody levels to the recombinant *P. falciparum* erythrocytic stage parasite proteins apical membrane antigen 1 (AMA1), merozoite surface protein 2 (MSP2) and merozoite surface protein 1, 19 kDa fragment (MSP1_19_). In addition, antibodies to a synthetic peptide (NANP)_4_ representing the major B cell epitope repeat of the circumsporozoite protein (CSP) of *P. falciparum*, and the amount of total IgE, were measured. Assays were carried out using a single uniform combined protocol for all samples.

### Antigens

Recombinant AMA1 (3D7 sequence) [22], MSP2 (3D7 sequence) [23] and MSP1_19_ (Wellcome sequence) [24] were all of vaccine quality. AMA1 was kind gift of Alan Saul (Malaria Vaccine Development Unit, NIAID, USA), MSP2 of Robin Anders (La Trobe University, Melbourne, Australia) and MSP1_19_ of Shirley Longacre (Pasteur Institute, Paris, France). The 16 residue synthetic peptide (Asn-Ala-Asn-Pro)_4_ (NANP)_4_ was a kind gift of Eric Tongren (CDC, Atlanta, USA).

### Serum references

A reference plasma pool obtained from 20 malaria-exposed adults in the Gambia (Brefet4 pool) [25] was used as a *P. falciparum* standard on each plate coated with malaria antigen and the IgE reference serum, 75/502 (NIBSC), was used for IgE determinations. The negative control serum was a pool of 40 European individuals who had never been exposed to malaria.

### ELISA

ELISA was carried out as previously described [25] and as detailed in Additional file 4 Briefly, ELISA plates (Immulon 4-HBX, Fisher Scientific UK Ltd, Loughborough, UK) were coated with antigen (50 µl in 0.05 M sodium carbonate pH 9.6) at a concentration of 0.5 µg/ml (AMA1, MSP2 and IgE) or 1 µg/ml (MSP1_19_ and (NANP)_4_) or anti-human IgE MAb (M107 from Mabtech AB, Nacka Strand, Sweden) (50 µl of 1 µg/ml), incubated overnight at 4 °C, washed three-fold with PBS-0.05 % Tween 20 (PBS/T) (Sigma, Gillingham, Dorset, UK), blocked with 200 µl of blocking solution (2 % skimmed milk powder in PBS/T) for 3 h at ambient temperature and washed three times with PBS/T. Samples of each characterization sample (see above) were diluted in blocking solution and aliquots added in duplicate to plates as follows : 50 µl of 1:200 final dilution for (NANP)_4_-coated plates; 50 µl of 1:1,000 final dilution for MSP1_19_, MSP2 and IgE and 100 µl of 1:2,000 for AMA1 plates. After overnight incubation at 4 °C, plates were washed six times with PBS/T, 50 µl of horseradish peroxidise-conjugated rabbit anti-human IgG (DAKO) (1:5,000 in PBS/T) added to each well and plates incubated for 3 h at room temperature. Following six-fold washing in PBS/T, 100 µl of Sigma-Fast o-phenylenediamine dihydrochloride (OPD) reagent solution (Sigma) was added to each well. Plates were developed at room temperature for 10–15 min (20–30 min for (NANP)_4_ ELISA), the reaction stopped by addition of 25 µl 2 M H_2_SO_4_ and plates read in a plate reader (Molecular Devices, Wokingham, Berkshire, UK) at 492 nm. A standard curve was fitted to the reference serum data obtained for each antigen as previously outlined [25] with the reference serum assigned an arbitrary concentration of 1,000 U/ml for all antigens. Plate values were normalized using the standard curves, and sample antibody concentrations (in U/ml) were calculated (see Additional file 4). For the Tanzania (Moshi) ELISAs, only three values were obtained, those for AMA1, MSP1_19_ and MSP2, using independently expressed preparations of the same antigens (see Additional file 4).

### Determination of malaria status

Thick and/or thin blood films were used to determine malaria status and all data were transformed to a standard parasites/µl.

### Data cleaning

To create the final dataset for analysis the clinical, genetic and antibody data were merged based on the unique ID code assigned to each participant at the time of sampling. Entries that did not contain all three data components after merging were removed. Duplicated ID codes were resolved if the correct entry could be identified, otherwise both entries were removed. This gave a dataset with 15,216 individuals. Records where gender miss-matched between clinical and genetic gender or DNA samples with <90 % pass rate across 65 core SNP assays [21] were also removed (540 miss-matched gender, 1,237 <90 % pass-rate and 140 both miss-matched gender and pass-rate), leaving 13,299 individuals in the merged dataset eligible for analysis. This dataset was then used to quality control the remaining 137 SNP-assays, removing assays with pass rates <80 % across all samples (n = 3). Assays for SNPs monomorphic across all sites (n = 18) or Amelogenin [gender determining (n = 3)] were also removed from further analysis leaving a total of 178 SNPs for analysis (65 core and 113 extra).

Antibody titres were log base 10 transformed to obtain approximate normal distributions for parametric analyses. Once logged all anti-malarial antibodies showed some evidence of a bimodal distribution, while IgE levels showed a negatively skewed distribution (Additional file 5). In the MSP-1 data (Additional file 5) a number of individuals were identified (log_10_ titre of 4.85, n = 216) that were not measured correctly due to technical difficulties. These were removed and excluded from further analyses. Plots of the residuals from the regression analyses (see below) were made (Additional file 6) in order to check that the antibody data were normally distributed.

### Statistical analysis

All analyses were carried out using the statistical package R [26, 27]. Standard linear regression models were used to investigate associations between non-genetic factors and antibody levels at all sites combined. Covariates were included in the main analyses if they were collected by all sites and were associated with antibody levels; parasite density and bed-net use were not covariates in this model as they were not measured at all sites; separate regression models were run for the subset of sites that provided these data. Details of the linear regression models used are given in Additional file 7.

Tests of association between each SNP and antibody levels with adjustment for age, gender, malaria status, ethnicity, village, and sample month were run using standard linear regression models at each site under a variety of modes of inheritance: additive, dominant, recessive, and heterozygote (Additional file 7). Results at each site were meta-analysed to obtain the effect of genotype on antibody level. Meta-analysis provided a beta coefficient and p-value for each SNP-antibody association using each model. A standard Bonferroni correction based on analysis of association between 178 SNPs and antibody levels to 5 antigens would give threshold for significance of 6 × 10^−5^. A more accurate significance threshold would need to take into account the level of linkage disequilibrium between SNPs, but this is difficult to estimate across multiple different populations. The Bonferroni significance threshold should, therefore, be regarded as an approximation and probably over-conservative.

Logistic regression was used to investigate the effect of SNPs with significant SNP-antibody associations on microscopic malaria infection, adjusted for relevant clinical parameters (Additional file 7). Data from Senegal, Mali (Pongonon), Sudan and Sri Lanka were not included in this analysis as their study populations were either entirely microscopically negative or entirely microscopically positive for malaria at the time of sampling.

Linear regression was used to investigate the effect of SNPs with significant SNP-antibody associations on parasite density, adjusted for relevant clinical parameters (Additional file 7). Data from Senegal, Kenya, Sudan, and Sri Lanka were not included in this analysis as parasite density was not recorded at these sites.

In all regression models, age was included as a grouped rather than continuous variable to allow for a non-linear relationship with antibody levels. Sample month was included as a covariate in regression models as a proxy for rainy season, the timing of which varied between sites.

## Results

### Epidemiological factors

**Fig. 1:**
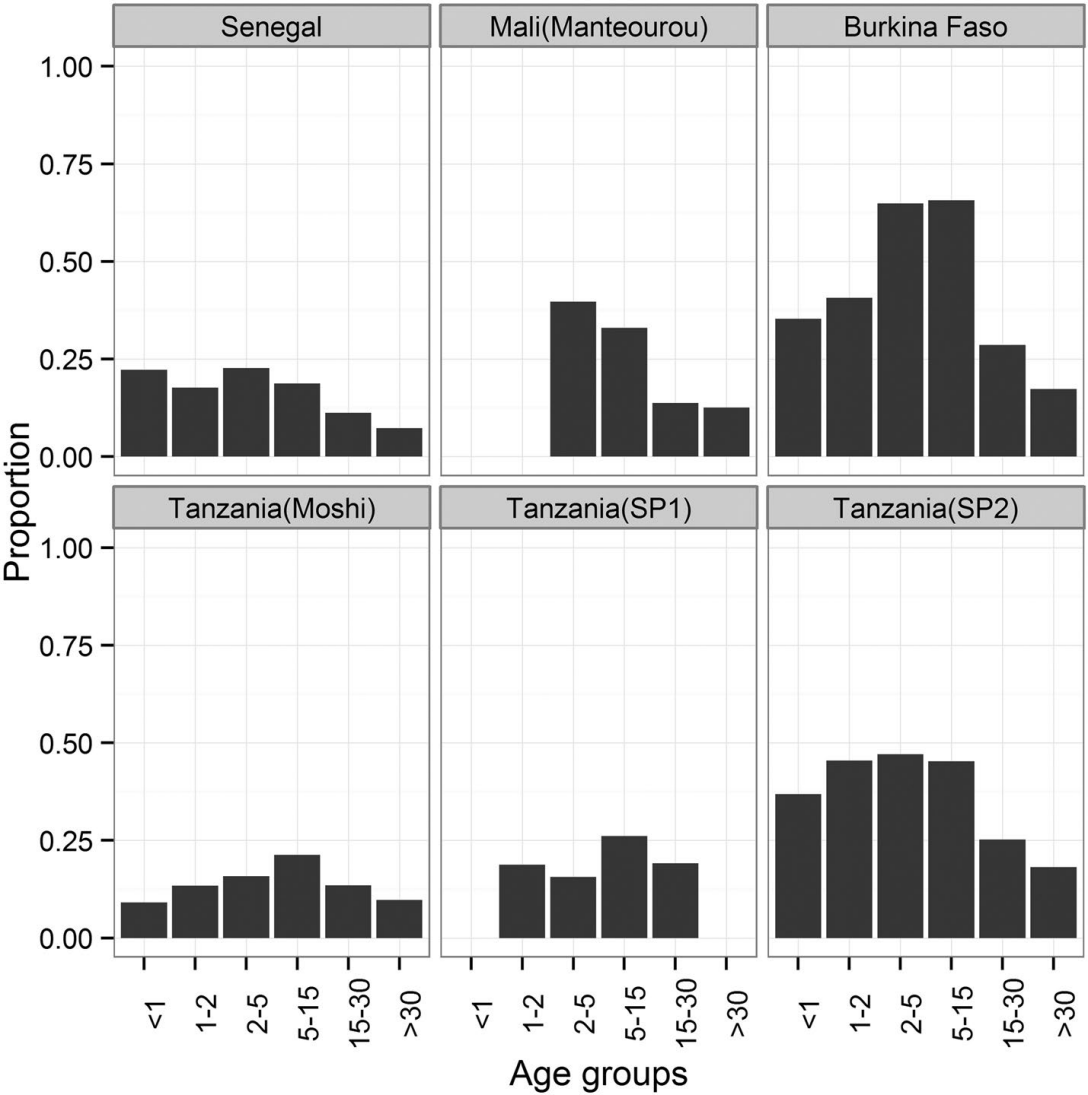
Proportion of individuals microscopically *Plasmodium falciparum* positive. Data shown are for the six sites for which surveys sampled across different age groups. Studies not included here include: Mali (Pongonon) where only malaria positive were included, Kenya (where only one age group was sampled) and Sudan and Sri Lanka were all negative at the time of survey.

A number of study designs were used across sites; Mali (Manteourou), Burkina Faso and Tanzania studies collected data during their malaria transmission season, Mali (Pongonon) included only individuals positive for malaria, Senegal and Sudan based data collection on active case detection, Kenya collected data as part of a birth cohort study at aged 7 years-old, and Sri Lanka followed up individuals that had been malaria positive in 1992/93. Further details can be found in Table 1, Additional file 1 and the MalariaGEN Website [18]. After data curation and quality control (see “Methods”), 13,299 individuals were included for analysis (Table 1) for five antibodies and 178 SNPs. At all sites the ratio of males to females was approximately 1:1. The number of ethnic groups within a site varied, and individuals belonging to the Fulani ethnic group (referred to here as Peulh) were collected in Senegal, Manteourou in Mali and Burkina Faso. Altitude ranged from sea level (0 m) in the coastal town of Kilifi in Kenya to 1,845 m in the mountainous areas of Moshi in Tanzania, and reported bed net usage ranged from 16.5 % in Burkina Faso to 95.7 % in Sri Lanka. In Senegal, Sudan and Mali (Pongonon) the malaria prevalence given in Table 1 was affected by data availability or study design and thereby did not necessarily reflect malaria prevalence in the general population at the time of sampling; at remaining sites the prevalence of microscopically detectable infection ranged from 0 % in Sri Lanka to 44.3 % in Burkina Faso. The prevalence of microscopically positive malaria infection is shown in Fig. 1 for the six sites having age-distributed community data. Overall there was a general increase in prevalence from the <1 year-old age group to a peak in the 5–15 years-old age group (OR = 3.49, P < 0.001; Table 2) with a decrease thereafter; the lowest prevalence was seen in those aged >30 years-old (OR = 0.63, P = 0.01). Compared to the <1 year-old group, parasite density increased in the 1–2 years-olds (beta = 0.26, P = 0.041) was similar in the 2–5 years-old group and was significantly lower in the older age groups with the lowest density seen in >30 years-olds (beta = −0.62, P = <0.001; Additional file 8). No significant difference were found in the odds of having a microscopically positive blood smear between males and females (OR males vs female = 1.09, P = 0.109), although males had a marginally higher parasite density than females (beta males vs females = 0.09, P = 0.005, Additional file 8).

**Table 1:** Details of participation, gender ratio, age distribution, ethnicity breakdown, altitude range, bednet usage, malaria prevalence and study design for each site that provided data to the study

Study location	Number of participants^a^	Gender (%)	Age (%)							Ethnicity^b^ (%)		Altitude range (m)	Bednet usage (%)	Malaria prevalence (%)		Timings of clinical data collection
		Male	<1	1–2	2–5	5–15	15–30	>30	NA					Slide-positive	NA	
Senegal	497	45.7	1.8	3.4	10.7	37.6	21.5	24.9	–	Wolof: Serer: Peulh: Serer Niominka: Mandigue: Other:	35.6 33.4 10.7 10.1 6.0 4.2	15–51	–	14.7	85.3^c^	LS with ACD; TS; 2006/2007
Mali (Pongonon)	312	53.5	–	3.2	36.2	54.5	2.2	3.8	–	Dogon: Other:	95.2 4.8	49–352	–	100	–	CS & CES; TS; 2006/2007
Mali (Manteourou)	643	43.4	–	–	10.6	37.6	21.5	24.9	–	Dogon: Peulh:	51.2 48.8	267–280	–	24.6	–	CS; TS; 2006/2007
Burkina Faso	1,897	43.4	2.7	3.1	13.2	34.9	23.8	22.3	–	Peulh: Mossi: Rimaibe: Other:	38.9 32.4 27.4 1.3	304–305	16.5	44.3	2.5	CS; TS & IDS; 2007/2008
Sudan	84	36.6	–	–	–	44.0	34.5	20.2	1.2	Hausa: Masalit:	51.2 48.8	183–381	–	–	100^c^	LS & CS; TS & IDS; 2007/2008
Kenya	1,809	52.0	–	–	–	100	–	–	–	Giriama: Chonyi: Kauma: Mjikenda: Other:	78.3 13.1 5.4 1.3 1.9	0	90.8	16.6	–	BCS
Tanzania (Moshi)	6,084	40.7	6.2	4.8	16.8	33.9	21.2	16.7	0.4	Pare: Wasambaa: Chagga: Wabondei: Other:	40.9 36.1 14.3 7.9 0.8	196–1,845	–	15.6	0.5	CS; TS; 2006/2007
Tanzania (Tanga SP1)	623	43.2	0.3	2.6	25.7	63.8	7.5	–	–	Wasambaa: Mzigua: Muha: Other:	65.8 10.8 3.4 20.0	223–700	54.6	22.6	–	CS; TS; 2001/2002
Tanzania (Tanga SP2)	552	47.5	3.4	2.0	9.2	51.6	23.7	10.0	–	Wasambaa: Mdigo: Mmakonde: Mzigua: Mseguju: Wabondei: Pare: Other:	35.0 30.2 5.4 5.1 4.7 4.2 4.2 11.2	0–1,009	28.6	37.7	–	CS; TS; 2004
Sri Lanka	798	49.5	–	–	–	0.5	37.1	62.2	0.3	99.4 Other: 0.6	99.4 0.6	55–397	95.7	0	–	LS with ACD; 1992/1993; samples collected 2006/2007

NB: *ACD* active case detection, *BCS* birth cohort study, *CES* chloroquine efficacy study, *CS* cross-sectional study, *IDS* intermittent dry season, *LS* longitudinal study, *TS* transmission season.

^a^Number of participants for whom clinical data, genetic data and antibody data could be matched.

^b^Ethnic groups with fewer than 20 individuals are recoded as “other”.

^c^These studies obtained data on microscopic-detectable infection in few (n = 81; Senegal) or none (Sudan) of their participants at the time of sampling.

**Table 2:** Results of logistic regression analysis investigating the effect of age, gender and HbS genotype on malaria status as determined by microscopy^a^ using combined data.

Factor	Adjusted OR (95 % CI)	p-value	Malaria status by microscopy			
			Individuals positive (total = 1,966)		Individuals negative (total = 7,261)	
			N	(%)	N	(%)
Age (years)						
<1	1		37	(10.9)	303	(89.1)
1–2	1.33 (0.87–2.05)	0.191	47	(17.2)	225	(82.7)
2–5	*2.65 (1.88–3.72)*	*<0.001*	311	(26.7)	854	(73.3)
5–15	*3.49 (2.53–4.86)*	*<0.001*	1,141	(25.6)	3,314	(74.4)
15–30	1.21 (0.87–1.70)	0.263	281	(17.1)	1,365	(82.9)
>30	*0.63 (0.44–0.90)*	*0.010*	147	(11.1)	1,177	(88.9)
Gender						
Female	1		1,044	(20.1)	4,142	(79.9)
Male	1.09 (0.98–1.21)	0.109	922	(22.8)	3,119	(77.2)
HbS						
11	1		1,824	(21.4)	6,718	(78.6)
12	*0.75 (0.61–0.92)*	*0.005*	136	(21.1)	508	(78.9)
22	ND	ND	0	(0)	8	(100)

Data from Senegal, Mali (Pongonon), Sudan and Sri Lanka are not included as participants are either entirely microscopically malaria-positive or malaria-negative.

Reference category is “negative” (*n* = 7261).

Results significant at 0.05 level are highlighted in italics.

ND: Results not shown as unable to obtain estimates for HbSS without any infected individuals.

NB: *CI* confidence interval, *OR* odds ratio.

^a^Also adjusted for altitude, village (>20), ethnicity (>20), sample month (>20) and study; results not shown.

### Correlations between antibody levels

**Fig. 2:**
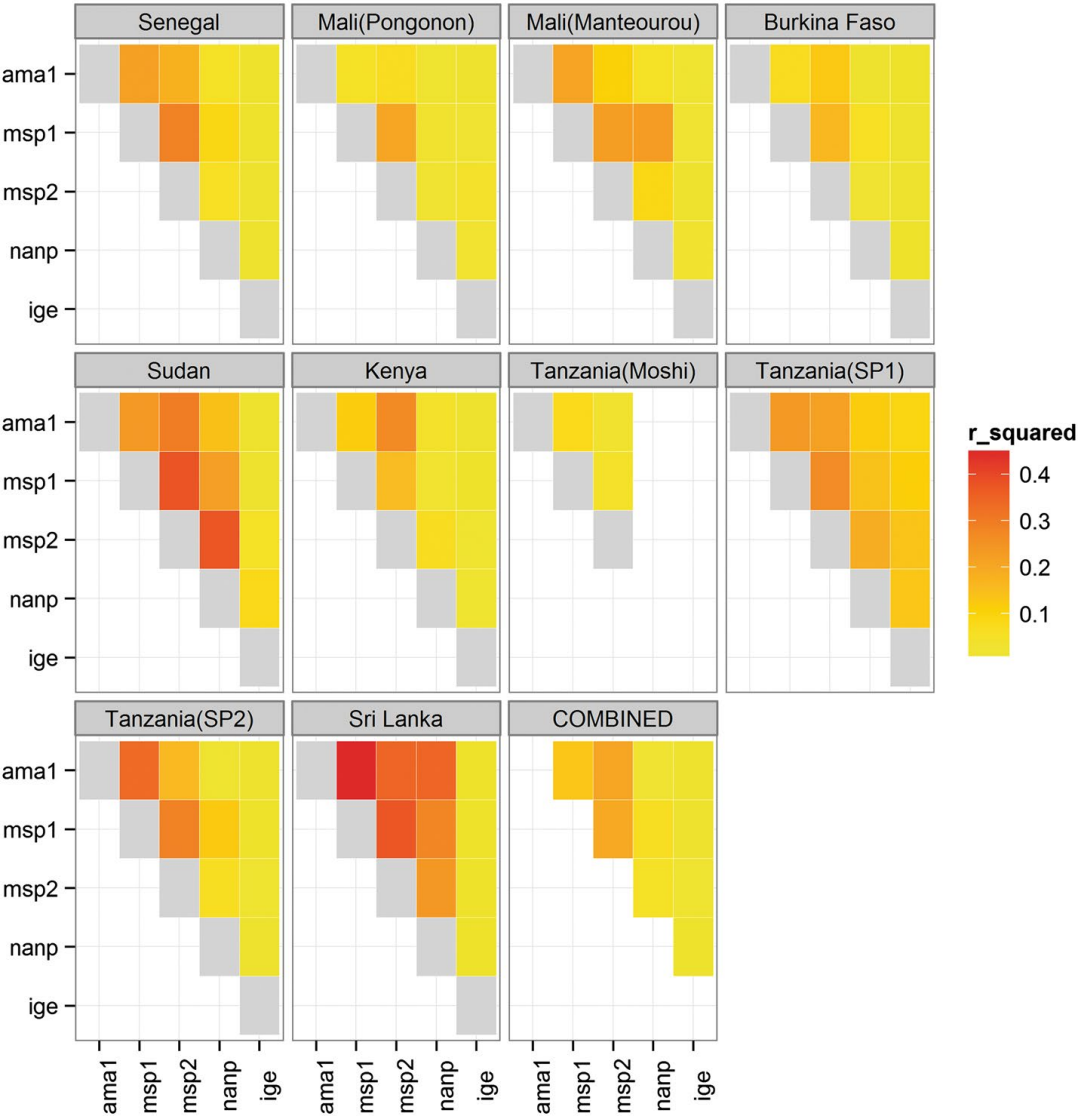
Heatmap matrix plot of correlations between logged antibody titres at each site. Pairwise correlations between logged anti-AMA1, anti-MSP1, anti-MSP2, anti-NANP, and total IgE calculated as R-squared. Strongest correlations shown in *red* and weakest correlations shown in *yellow*. Correlations of antibodies with themselves shown here in *grey.*

The greatest correlations were seen between antibodies to merozoite antigens (r^2^ range = 0.21–0.35), followed by correlations between these antibodies and anti-(NANP)_4_ (r^2^ range = 0.11–0.24), with total IgE showing little or no correlation with any of the anti-malarial antibodies (r^2^ range = 0–0.01, Fig. 2 ‘Combined’ panel). Similar trends were observed at individual sites (Fig. 2), although some variation between sites was visible. This pattern of correlations remained after accounting for other clinical variables as shown by the residuals from the regression analyses (Additional file 9). It is possible that the strengths of correlation between antibodies might depend on the level of infection, and thus depend on study site and the age of the individuals. Therefore, a linear regression model was applied using both age-groups and sites as categorical variables and no significant associations were observed with study-site or for age-group (Additional files 10, 11).

### Factors influencing antibody levels

**Fig. 3:**
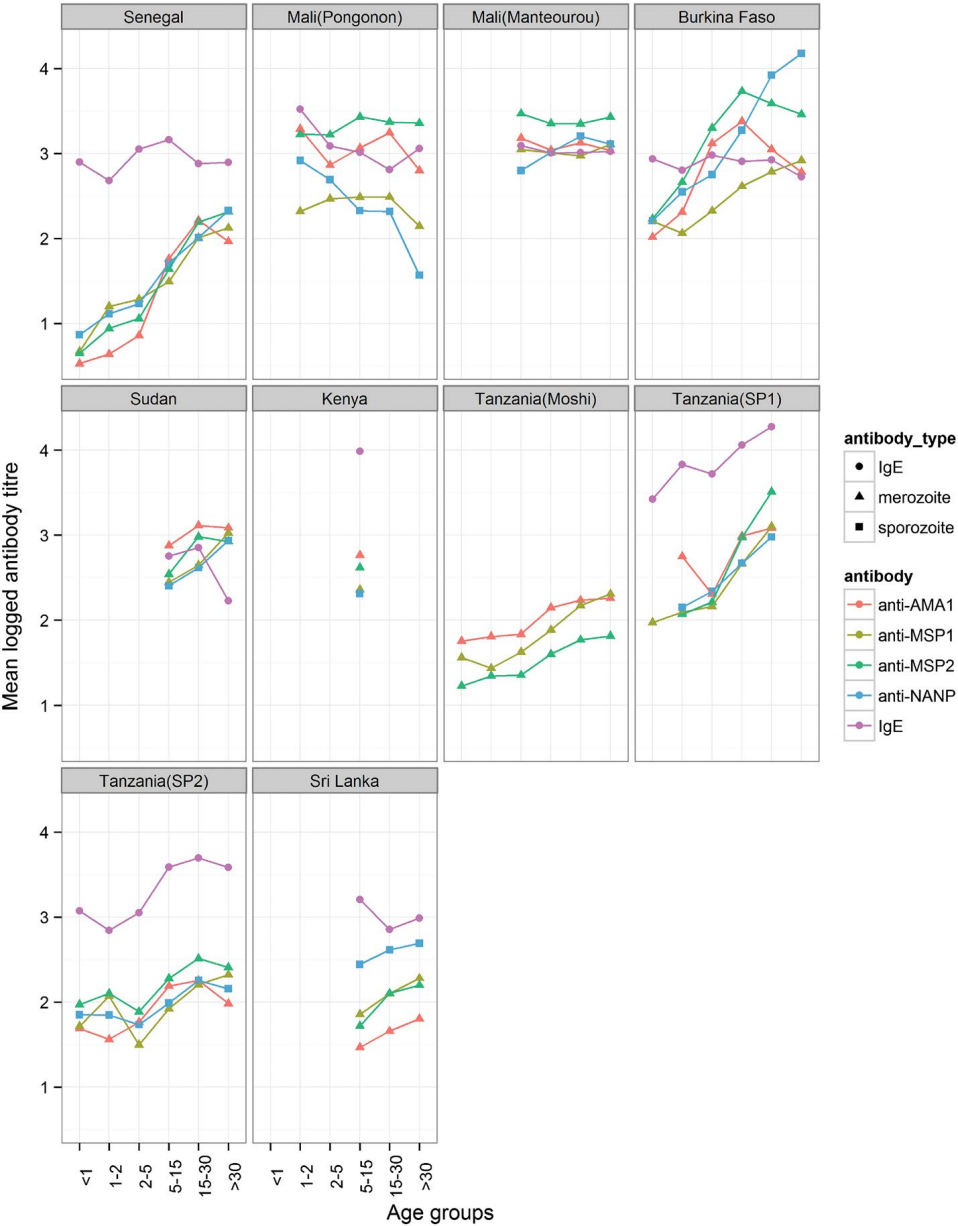
Mean logged antibody titre for the five measured antibodies, for each age-group for each site. Each *colour* represents a different antibody while the shape of the point represents the antibody type: (*triangle*) for anti-merozoite, (*square*) for anti-sporozoite, and (*circle*) for total IgE.

Individuals living in malaria-endemic areas are known to acquire immunity with age, this can be observed in Fig. 3 as the increase in antibody titres to AMA1, MSP1, MSP2, and (NANP)_4_ across the different age groups. The trend for increasing anti-malarial antibody levels with age was confirmed using regression analysis on combined data across all sites, adjusting for gender, malaria infection, altitude, month, village, and ethnicity (Table 3). All anti-malarial antibodies showed significant increases with each age group compared to the <1 year-old group. However, no overall significant change was observed in IgE levels with age, apart from the 15–30 years-old group (beta = 0.22, P = 0.034). Gender was a significant factor for all antibodies (although anti-AMA1 was marginal, Table 3).

**Table 3:** Results of linear regression analysis investigating the effect of age, gender and malaria status as determined by microscopy^b^ on logged antibody levels to AMA1, MSP1, MSP2, NANP and IgE

Factor	Anti-AMA1 levels (n = 10,137)		Anti-MSP1 levels (n = 10,200)		Anti-MSP2 levels (n = 10,283)		Anti-NANP levels (n = 5,531)^a^		Total IgE levels (n = 4,961)^a^	
	Beta (95 % CI)	p-value	Beta (95 % CI)	p-value	Beta (95 % CI)	p-value	Beta (95 % CI)	p-value	Beta (95 % CI)	p-value
Age (years)										
<1	0		0		0		0		0	
1–2	0.04 (−0.09 to 0.17)	0.576	−0.10 (−0.22 to 0.01)	0.083	*0.16 (0.05 to 0.28)*	*0.006*	*0.30 (0.04 to 0.55)*	*0.023*	0.09 (−0.17 to 0.35)	0.508
2–5	*0.25 (0.15 to 0.35)*	*<0.001*	0.08 (−0.01 to 0.17)	0.087	*0.29 (0.20 to 0.38)*	*<0.001*	*0.50 (0.30 to 0.71)*	*<0.001*	0.09 (−0.12 to 0.30)	0.398
5–15	*0.57 (0.47 to 0.67)*	*<0.001*	*0.37 (0.28 to 0.45)*	*<0.001*	*0.61 (0.52 to 0.69)*	*<0.001*	*0.87 (0.67 to 1.07)*	*<0.001*	0.19 (−0.01 to 0.40)	0.066
15–30	*0.55 (0.46 to 0.65)*	*<0.001*	*0.65 (0.57 to 0.74)*	*<0.001*	*0.72 (0.63 to 0.81)*	*<0.001*	*1.43 (1.23 to 1.63)*	*<0.001*	*0.22 (0.02 to 0.43)*	*0.034*
>30	*0.53 (0.43 to 0.63)*	*<0.001*	*0.81 (0.72 to 0.90)*	*<0.001*	*0.72 (0.63 to 0.81)*	*<0.001*	*1.61 (1.40 to 1.81)*	*<0.001*	0.14 (−0.07 to 0.35)	0.179
Gender										
Female	0		0		0		0		0	
Male	−0.03 (−0.07 to 0)	0.058	*−0.08 (−0.11 to −0.05)*	*<0.001*	*−0.05 (−0.07 to −0.02)*	*0.002*	*−0.07 (−0.11 to −0.03)*	*0.004*	*0.12 (0.09 to 0.16)*	*<0.001*
Microscopy result										
Negative	0		0		0		0		0	
Positive	*0.17 (0.13 to 0.22)*	*<0.001*	*0.15 (0.11 to 0.19)*	*<0.001*	*0.21 (0.18 to 0.25)*	*<0.001*	−0.03 (−0.08 to −0.02)	0.263	*0.06 (0.01 to 0.11)*	*0.021*

Results shown as betas, which indicate the direction of effect of the clinical covariate on antibody levels. Beta < 0 indicate a decrease in antibody levels and beta > 0 indicate an increase in antibody levels. 95 % confidence intervals that do not span 0 indicate an effect that is significant at p = 0.05.

NB: *CI* confidence interval; results significant at 0.05 level are highlighted in italics.

^a^Data not available for Tanzania (Moshi).

^b^ Also adjusted for village (>20), ethnicity (>20), sample month (>20) and study; results not shown but ANOVA p-values were <0.001 for all antibodies.

Across all sites, antibody levels to AMA1, MSP1 and MSP2 were significantly increased in individuals with microscopically detectable infection (Table 3); whilst at the six sites that quantified parasitaemia, increasing parasite density was significantly associated with decreased antibody levels to AMA1, MSP1 and MSP2 (Additional file 12). There was no significant association of microscopic positivity or parasite density on anti-(NANP)_4_ levels. Total IgE levels were slightly raised in infected individuals (beta = 0.06, P = 0.021; Table 3) but did not differ significantly with increasing parasite density (Additional file 12). Similar trends were observed when this analysis was run for each site separately, although significance was influenced by sample size (Additional file 13). At five sites that recorded bed-net use at the time of sampling, no observed significant effect was seen for antibody levels (Additional file 14).

### Allele frequency of SNPs

Allele frequencies in Sri Lanka were generally different to those observed at African sites (Additional file 15). Most notably, the *DARC* gene SNP-derived allele (rs2814778) was not observed in Sri Lanka but was at a frequency >0.99 in the African populations. Differences in allele frequencies between East and West African populations were also observed: the *CD36* gene SNP (rs3211938) had a mean derived allele frequency of 28.9 % (range 0.7–61.9 %) in West Africa compared to 7.2 % (range 2.7–9.8 %) in East Africa; the *HBB* gene SNPs (rs334; HbS and rs33930165; HbC) had mean derived allele frequencies of 3.5 % (range 1.5–4.6 %) and 5.5 % (range 0.7–10.2 %), respectively, in West Africa compared to 5.9 % (range 1.6–7.7 %) and 0.006 % (range 0.0–0.003 %) respectively in East Africa. SNPs that were polymorphic in East Africa but not in West Africa included three *RAD50* gene SNPs (rs28903086, rs28903088, rs28903092), one *IL4* SNP (rs4986964), one *IFNGR1* gene SNP (rs11575936) and two *STAT6* gene SNPs (rs3024978, rs3024952). An *FCER2* gene SNP (rs35825847) was the only SNP polymorphic in West Africa but not in East Africa.

### SNP-antibody association analyses

**Fig. 4:**
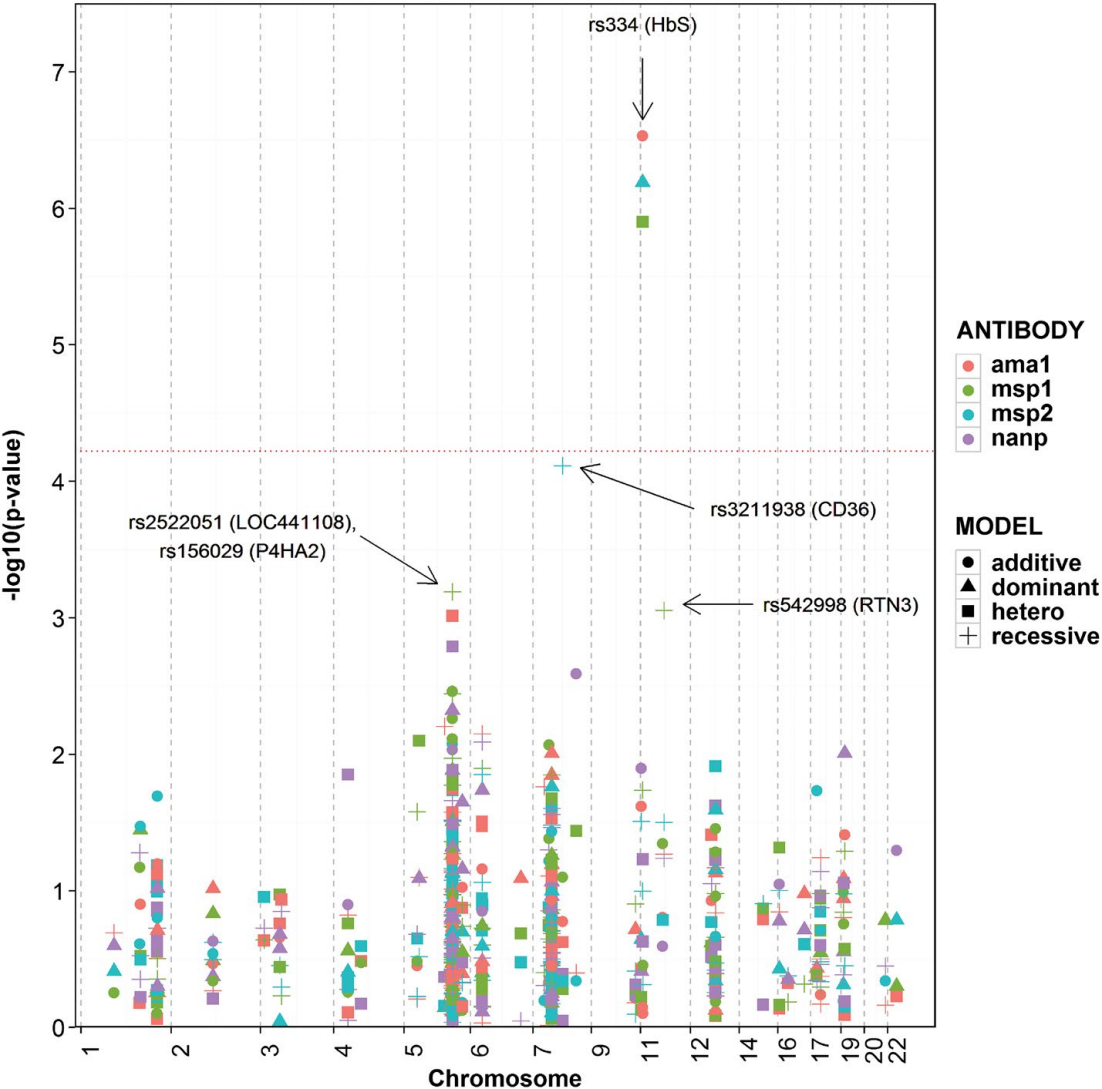
Plot for 178 SNPs with logged anti-malarial antibody levels. Values of –log_10_ p-values plotted against chromosomal positions; only the lowest meta-analysis p-value for each SNP-antibody association is plotted. The red dotted line indicates a Bonferroni threshold p-value of 6 × 10^−5^. Each *colour* represents a different anti-malarial antibody while the shape of the point represents the genetic model of best fit for the SNP-antibody association: (*circle*) for additive, (*triangle*) for dominant, and (*square*) for heterozygote and (*plus*) for recessive. Adjusted for age, gender, microscopy result, village (>20), ethnicity (>20), sample month (>20) and study.

Figure 4 shows the nominal P values from analysis of the SNP-antibody associations across all sites. The only candidate SNP that met the Bonferroni-corrected significance threshold (P = 6 × 10^−5^, see Methods) was rs334 in HBB, encoding sickle haemoglobin. Compared to HbAA, HbAS individuals had lower concentrations of antibodies to merozoite antigens AMA1 (beta = −0.17, P = 2.9 × 10^−7^), MSP1 (beta = −0.15, P = 1.3 × 10^−6^) and MSP2 (beta = -0.14, P = 6.5 × 10^−7^). No association of HbAS was seen for anti-(NANP)_4_ antibodies.

A candidate SNP in CD36 came close to the Bonferroni-corrected threshold, which is probably over-conservative. Compared to ancestral homozygotes and heterozygotes combined, recessive homozygotes at rs3211938 (*CD36*) had significantly lower concentrations of antibodies to MSP2 (beta = −0.23, P = 7.7 × 10^−5^).

Weaker associations were observed at three other loci and, while they are not considered here to be significant, for completeness they were included in the meta-analysis. r2522051 (LOC441108) showed a recessive association with anti-MSP1 levels (beta = −0.06, P = 6.4 × 10^−4^). rs156029 (P4HA2) showed heterozygous association with anti-AMA1 levels (beta = −0.05, P = 9.7×10^−4^). rs542998 (RTN3) showed recessive association with anti-MSP1 levels (beta = 0.06, P = 8.8 × 10^−4^). No associations were found for antibody levels to (NANP)4 (Additional file 16). Including parasite density in the model did not appreciably change these findings (Additional file 17).

**Fig. 5:**
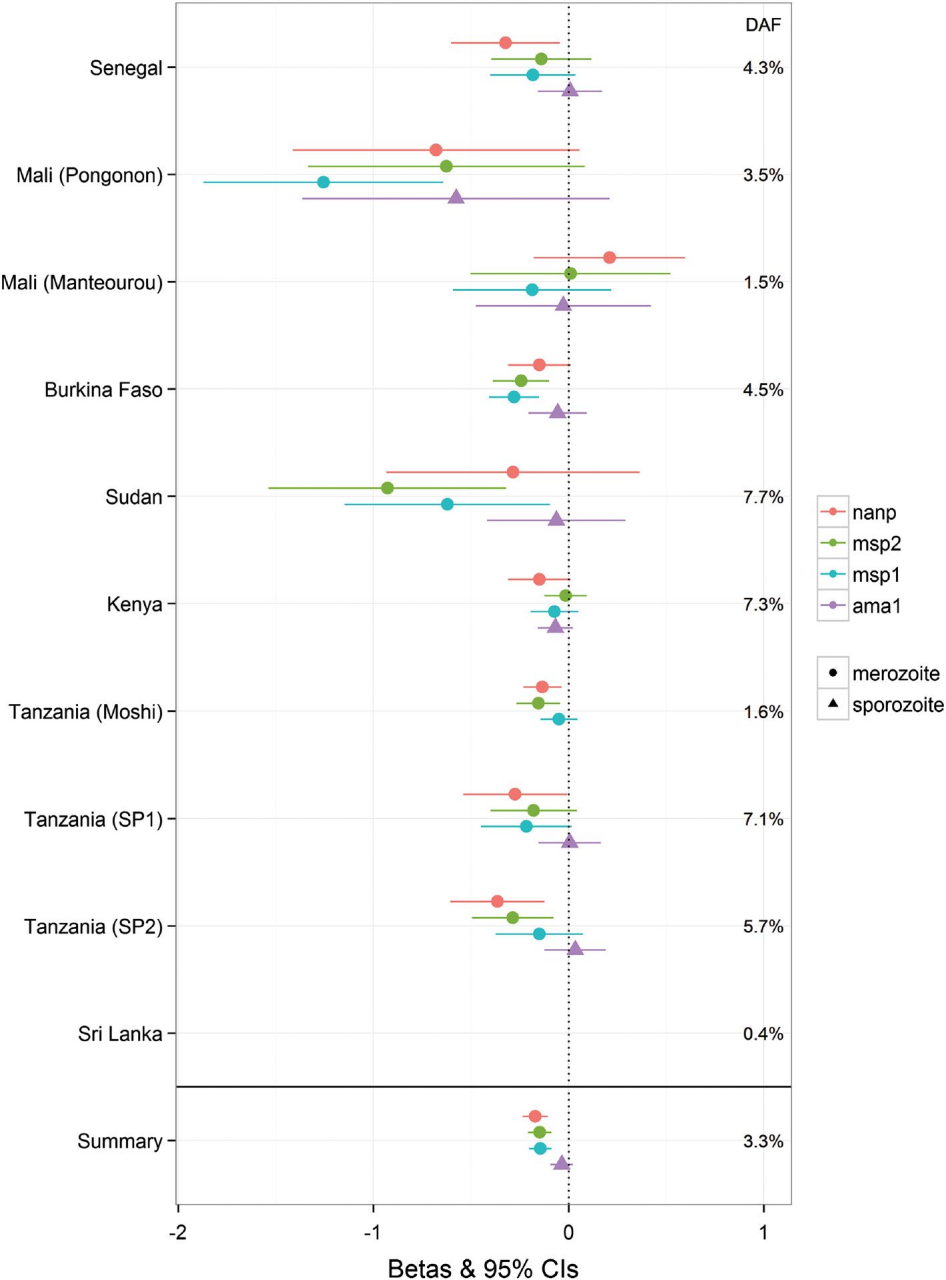
Forest plot of the HbS (rs334) association with antibodies to AMA1, MSP1, MSP2 and NANP. Points correspond to beta values obtained from meta-analysis of results obtained from linear regression models of SNP with logged antibody levels, adjusted for relevant clinical covariates. *Lines* represent 95 % confidence intervals. Each *colour* represents a different antibody while the shape of the point represents the antibody type: (*circle*) for anti-merozoite and (*triangle*) for anti- sporozoite. Summary of meta-analysis betas obtained from combined data are represented as (*square*). Summary P-values: ama1 = 2.9 × 10^−07^; msp1 = 1.2 × 10^−06^; msp2 = 6.5 × 10^−07^; nanp; = 0.2.

In order to look at the consistency of effect we have made forest plots of the betas. Figure 5 shows the beta values, 95 % confidence intervals and p-values for the effect of HbAS on the four anti-malarial antibodies at each site. The direction of effect is mostly consistent across sites for the three merozoite antibodies but no effect was seen at any site for anti-(NANP)_4_ antibodies, except in Mali (Pongonon). The results of meta-analysis for the 4 marginal genes (*CD36* and *LOC441108*, *P4HA2* and *RTN3*) are shown in Additional file 18 as forest plots along with their p-values.

### Association of HbAS and malaria infection

Given the association between sickle and anti-malarial antibody levels (see above), a regression analyses was conducted between malaria infection (microscopy result and parasite density) and sickle cell trait. HbAS individuals were significantly protected from becoming microscopically positive for malaria infection (OR = 0.75, P = 0.005; Table 2). A further analysis of parasitaemia levels at six sites providing information showed that overall there was no difference between HbAA and HbAS individuals (Additional file 8). Compared to infants (<1 year-olds) levels of parasitaemia increased in the 1–2 and 2–5 years-old groups and then decreased into adulthood (Additional file 8).

## Discussion

This dataset highlights considerable differences in malaria prevalence, antibody titres and study design between the sites that provided data. There are challenges associated with combining heterogeneous data; however, after quality control, a large dataset was compiled with 13,299 individuals typed for 178 SNPs, measures for five antibody levels and a range of clinical covariates.

Of the ten sites in the study two found a 0 % prevalence (Sudan and Sri Lanka) at the time of sampling as measured by microscopic positivity and two had an ascertainment design not allowing an analysis of effect of age on malaria prevalence (Mali-Pongonon and Kenya). Across the remaining studies, there was a significant increase in the odds of having microscopically positive malaria from the 1 year-old to 5–15 years-old age groups (OR 3.49, P < 0.001), after which prevalence declined into adulthood (OR 0.63, P = 0.01 30 vs 1 year-old groups). This is similar to that observed at individual site studies [28–31]. Many factors can influence the prevalence of malaria but the age effect is assumed to reflect acquired immunity to the parasite. Infants are protected against clinical infection [32–34], possibly due to the presence of foetal haemoglobin [35–37], maternal antibodies [35, 38] and lactoferrin in breastmilk [39]. The risk of becoming infected with malaria then increases as this protection wanes. During this and subsequent periods, exposure to malaria during childhood induces immunity [40–42], with an increasing repertoire of epitopes recognized [43], such that adults are at lower risk of acquiring or carrying malaria parasites than children and adolescents.

Microscopic malaria prevalence at a site did not always predict the magnitude of the anti-malarial antibody response. In Sri Lanka falciparum and vivax prevalence has been decreasing to almost zero by the time of plasma collection for this study [17] yet anti-falciparum antibody titres were similar to those in the Tanga SP2 study where the prevalence was 37 %. It is believed that once immunity has been acquired then exposure to malaria parasites, even at sub-microscopic parasitaemia [44, 45], may be sufficient to boost antibody levels through long-term memory [46–48], therefore, helping to maintain antibody titres in regions (Sri Lanka and Sudan) of low endemicity in this study.

As expected, antibody titres to asexual blood stage antigens were most highly correlated with each other (r^2^ > 0.3), and showed less correlation with anti-(NANP)_4_ antibodies (r^2^ between 0.2 and 0.3) which are reflective of exposure to pre-erythrocytic parasites (sporozoites and liver stages). All anti-malarial antibodies showed similar distributions and peak titres from 1:100 to 1:1,000. Total IgE levels were not correlated with any of the other antibody levels. Although there was a marginally significant increase in total IgE levels with malaria infection, it was not possible to corroborate whether this was due to polyclonal induction of IgE antibodies during malaria infection [49–51] or changes in specific anti-malarial IgE.

A regression model was used to investigate the association of clinical variables with antibody titres. As reported previously [52–54] increasing age was found to be significantly associated with increased levels of antibodies to AMA1, MSP1, MSP2, and (NANP)_4_. A plateau effect was also seen with anti-AMA1, anti-MSP1 and anti-MSP2 in the older age groups similar to Calissano et al. [55].

As reported previously, being male was associated with decreased antibody levels to malarial antigens. Two contributing factors reported to explain this gender difference are the differential transcription and translation of IFN-γ between males and females [56] or an immunosuppressive effect of testosterone in males [57]. Conversely, being male was significantly associated with an increase in total IgE levels, which is consistent with other studies [58–60]. One other likely explanation is due to the different habits of males and females and their exposure to helminth and other parasitic infections [61–63].

As expected [49, 53], microscopically positive infection was significantly associated with increased antibody levels to the merozoite antigens AMA1, MSP1 and MSP2. Furthermore, the presence of these asexual stages was not associated with antibody titres to the pre-erythrocytic antigen (NANP)_4_ [64].

A regression model was used to investigate SNP associations with antibody levels that included several covariates for parameters that were identified as influencing antibody levels. Of all the SNPs and antibodies the most significant effect was seen for rs334 (HbS) with reduced antibody titres to merozoite antigens. Previous research has been inconsistent regarding the relationship between sickle cell trait and IgG responses to malaria: some studies have reported higher antibody levels in HbAS compared to HbAA individuals [65], whilst others have reported lower antibody levels in HbAS individuals [30], or even no difference between the two [54]. Miura et al. [30] found that HbAS children in Mali had significantly lower IgG levels to EBA175 and MSP2 compared to their HbAA counterparts and also tended to have lower IgG levels to AMA1 and MSP1, although these latter findings were not statistically significant. Several studies measured tetanus toxoid-specific IgG titres in HbAA and HbAS children and found no differences [30, 65], which Verra et al. [65] used to infer that HbAS children had lower IgG titres specifically to merozoite antigens. This specificity is confirmed in this study by finding significant differences in anti-AMA1, anti-MSP1 and anti-MSP2 levels but not in anti-(NANP)_4_ or total IgE levels. Verra et al. [65] concluded that the lowered IgG levels in HbAS children found in their study suggest that the malaria-protective effects of HbAS are not due to malaria-specific IgG responses.

The most likely mechanism for lowered antibody levels in HbAS individuals is by reducing the net exposure of the immune system to parasites; for example, by reducing invasion and development of parasites in HbAS erythrocytes [30, 54], enhancing phagocytosis of infected HbAS erythrocytes [30, 54, 65, 66], or by accelerating the removal of parasitized erythrocytes by the spleen through sickling [66]. These are all consistent with the observation (n = 9,227 individuals) that HbAS significantly reduced the odds of being microscopically positive compared to HbAA. Taken together with a significant reduction in anti-merozoite antibodies and no change in antibodies to pre-erythrocytic stages, the data suggest that HbAS affects asexual blood-stages only. One possible explanation would be for HbAS to exert its effect early after the release of merozoites from the liver by blocking or delaying growth in infected erythrocytes. If this were the case, then the time to establish a patent infection would be increased/extended thereby reducing exposure to the immune system. However, once a blood-stage infection is established the data (n = 2,272) suggest that HbAS erythrocytes are no longer able to attenuate parasite growth.

Indeed across 21 published studies, the majority having less than 1,000 individuals, there is no consensus on the effect of HbAS on parasite density or prevalence, although the weight of evidence is probably for no effect (review and meta-analysis [67]). It is still unknown whether parasites infecting HbAS and HbAA erythrocytes differ in virulence, but it is clear that in the absence of controlling parasitaemia, HbAS is highly protective against severe malaria phenotypes [21, 67].

There are several features of this study that increase the reliability of the associations found between HbAS and merozoite antibodies. Firstly, a large dataset was generated with over 13,000 individuals, 12,380 of whom were HbAA and 868 HbAS. Secondly, a similar effect size was found for HbS in association with anti-AMA1, anti-MSP1 and anti-MSP2 levels, using the heterozygote model. Thirdly, the p-values for each HbAS-antibody association were similar and highly significant even after adjusting for multiple testing. Finally, not including an interaction term between SNP and study in the model meant that all significant outcomes represented effects that were consistent across sites, as demonstrated in the Forest plots for HbAS with the merozoite antibodies. The only exceptions were the Manteourou study in Mali and the Sri Lanka study, which had opposite directions of effect to the other sites for anti-AMA1. Neither of these associations were significant and were likely due to relatively small sample sizes (Mali = 643, Sri Lanka = 497) combined with low derived allele frequencies for HbAS (Mali = 1.5 %; Sri Lanka = 0.4 %).

One SNP was found to be marginally associated with antibody levels to merozoite antigens. The SNP in CD36 (rs3211938) on chromosome 7 was associated with a reduction in anti-MSP2 levels using the recessive genetic model, which is consistent with the reduced anti-MSP1 levels observed in individuals recessive homozygous for this SNP in another area of Tanzania [68]. None of the other loci tested here showed evidence of association with antibodies in this multi-centre analysis with a significance of P < 10^−4^.

## Conclusion

A major strength of this study was its large sample size (n = 13,299), which enabled the detection of an effect of SNPs on antibody levels even at a significance level adjusted for multiple testing. The sample size also allowed adjustment for a multitude of potential confounding factors, and having participants from ten sites in seven different countries sampled using standardized methodologies allowed an analysis for consistency of SNP effect across sites. This study finds an association of HbS (rs334) carriage with lowered antibody levels to merozoite antigens AMA1, MSP1 and MSP2, that is highly significant and consistent across study sites. This study demonstrates the feasibility of combining data from heterogeneous sources and the findings support the notion that genetic factors can determine an individual’s immune response to malaria.

## Additional files

Additional file 1: Additional Table ST1 Details of Principal Investigators and Ethics Review Committees for each site. Details of Principal Investigators and Ethics Review Committees for each site. Additional file 2: Additional Table ST2: Details of covariates adjusted for in linear regression analysis of non-genetic factors with logged antibody levels, their relevance to the study and their previous association(s) with malaria or anti-malarial antibodies. Information on the covariates used in this study as con-variates in the various analyses. Additional file 3: Additional File LFST1A: Details of gene regions selected for genotyping and the SNP assay design details. This file contains a list of the genes from which SNPs were selected and typed for this study. Information is provided on the gene and its genomic location with respect to the Human Reference genome build GRCh37. Also provided is a table containing the assay design details for each SNP used in this study for the Agena Biosciences iPLEX genotyping platform. SNPs are grouped into multiplexes as assigned by the assay design software. Additional file 4: MalariaGEN Supplementary Sample Handling Procedures. This file includes more detail on sample handling procedures including extracting DNA, genotyping methodology, ELISA protocols with ELISA data processing. Additional file 5: Additional Figure SF1: Histograms of logged antibody levels before further manipulations were made. Histograms of the logged antibody titres across all samples for each antibody measured in this study. Additional file 6: Additional Figure SF2: Histograms of residuals from antibody linear regression models with non-genetic factors. Histograms of the residuals from the antibody linear regression model analysis with non-genetic factors. Additional file 7: Additional Table ST3A-D: Details of the linear regression models used in this study. Model formulae for the various linear regression analyses showing how the different co-variates were used. Additional file 8: Additional Table ST4: Results of linear regression analysis investigating the effect of age, gender and HbS genotype on parasite density in malaria-positive individuals using combined site data. Results of linear regression analysis investigating the effect of age, gender and HbS genotype on parasite density in malaria-positive individuals using combined data. This is an extension to that shown in the main text and is a reduced dataset as not all sites provided parasitaemia data. Additional file 9: Additional figure SF3: Heatmap matrix plot of the correlations between residuals from the antibody linear regression models with non-genetic factors at each site. Matrix plot of the pairwise correlations as r^2^ between antibody titres using data from all sites. Additional file 10: Additional figure SF4: Scatter plot of the correlations between residuals from the antibody linear regression models with non-genetic factors at each site and age-group. This plot shows the pairwise correlations (as r^2^) for each malaria–malaria antibody pair as a function of each study site. The sites are ordered according to malaria microscopically-positive-prevalence recorded at sample collection (low to high). Several panels are shown according to age-group. Additional file 11: Additional table ST5: Linear regression analysis of the correlations between residuals from the malaria antibody pairs. This data shows the results of the analysis of the correlations between antibodies. Additional file 12: Additional Table ST6: Results of linear regression analysis investigating the effect of age, gender and parasite density on logged antibody levels to AMA1, MSP1, MSP2, NANP and IgE. Results of linear regression analysis investigating the effect of age, gender and parasite density using combined data for each antibody measured in this study. This is an extension to that shown in the main text and is a reduced dataset as not all sites provided parasitaemia data. Additional file 13: Additional Table ST7A–E: Results of site-specific linear regression analysis investigating the effect of age, gender and malaria status as determined by microscopy on antibody levels to the different antibodies measured in the study. This data shows the site-specific analyses of the various epidemiological parameters and antibody measurements. Additional file 14: Additional Table ST8: Results of linear regression analysis investigating the effect of age, gender, malaria status as determined by microscopy and bednet use* on logged antibody levels to AMA1, MSP1, MSP2, NANP and IgE. This table shows the results of the analysis of antibody levels with bednet use. Results shown as betas indicating the direction of effect of the clinical covariates on antibody levels. Additional file 15: Additional Table ST9: Details of 196 genotyped SNPs and their derived allele frequencies at each site. This table shows allele frequencies for each SNP analysed in this study according to each study site. Additional file 16: Additional Table ST10: Meta-analysis p-values obtained for 178 SNPs passing QC criteria detailed in materials and methods. Details of the analyses for each SNP and antibody across all sites for different genetic inheritance models. Additional file 17: Additional Figure SF5: Plot for 178 SNPs with logged anti-malarial antibody levels. Plot for the P-values of the analyses of 178 SNPs with logged anti-malarial antibody levels Adjusted for age, gender, parasite density, village (>20), ethnicity (>20), sample month (>20) and study. This is an extension to that shown in the main text and is a reduced dataset as not all sites provided parasitaemia data. Additional file 18: Additional Figure SF6: Forest plot of the association of A: *CD36* (rs3211938), B: *LOC441108* (rs2522051), C: *RTN3* (rs542998) and D: *P4HA2* (rs156029) with antibody levels to AMA1, MSP1, MSP2 and NANP. Forest plots of the betas obtained from meta-analysis of results obtained from linear regression models of SNP with logged antibody levels, adjusted for relevant clinical covariates.
