# Supplementary material for: Genetic determinants of anti-malarial acquired immunity in a large multi-centre study
Source: Malar J. 2015 Aug 28;14:333. doi: 10.1186/s12936-015-0833-x (PMC4552443; doi:10.1186/s12936-015-0833-x)
Supplement: Additional file 1: — Additional Table ST1 Details of Principal Investigators and Ethics Review Committees for each site. Details of Principal Investigators and Ethics Review Committees for each site. [file 12936_2015_833_MOESM1_ESM.pdf]

ADDITIONAL FILE 1: SUPPLEMENTARY TABLES

Genetic Determinants Of Anti-Malarial Acquired Immunity In A Large Multi-Centre Study

Jennifer M.G. Shelton, Patrick Corran, Paul Risley, Nilupa Silva, Christina Hubbart, Anna Jeffreys, Kate Rowlands, Rachel Craik, Victoria Cornelius, Meike Hensmann, Sile Molloy, Nuno Sepulveda, Taane G. Clark, Gavin Band, Geraldine M. Clarke, Christopher C.A. Spencer, Angeliki Kerasidou, Susana Campino, Sarah Auburn, Adama Tall, Alioune Badara Ly, Odile Mercereau-Puijalon, Anavaj Sakuntabhai, Abdoulaye Djimde, Boubacar Maiga, Ousmane Toure, Ogobara Doumbo, Amagana Dolo, Marita Troye-Blomberg, Valentina D. Mangano, Frederica Verra, David Modiano, Edith Bougouma, Sodiomon B. Sirima, Muntaser Ibrahim, Ayman Hussain, Nahid Eid, Abier Elzein, Hiba Mohamed, Ahmed Elhassan, Ibrahim Elhassan, Thomas N. Williams, Carolyne Ndila, Alexander Macharia, Kevin Marsh, Alphaxard Manjurano, Hugh Reyburn, Martha Lemnge, Deus Ishengoma, Richard Carter, Nadira Karunaweera, Deepika Fernando, Rajika Dewasurendra, Christopher J. Drakeley, Eleanor M. Riley, Dominic P. Kwiatkowski, and Kirk A. Rockett, in collaboration with the MalariaGEN Consortium,

Corresponding authors Kirk A. Rockett and Dominic P. Kwiatkowski
Wellcome Trust Centre for Human Genetics, University of Oxford, Roosevelt Drive, Oxford, UK

This file contains **Additional Table ST1:** Details of Principal Investigators and Ethics Review Committees for each site.

Additional Table ST1: Details of Principal Investigators and Ethics Review Committees for each site.

Country	ISO country code	Principal Investigator	Ethics Committee	Institution	Study identifier	Study description
Senegal	SN	Dr A. Tall	Direction de la Sante, Ministere de la Sante et de la Prevention Medicale	Unité d'épidémiologie, Institut Pasteur-Dakar	No. 1971/MSPM/DS/DER	Longitudinal survey with active case detection during transmission seasons of 2006/2007.
Mali	ML	Dr A. Djimdé	Comité d'Ethique, Faculte de Medicine de Pharmacie et d'Odonto-Stromatologie	Centre de Recherche et de Formation sur le Paludisme, Département d'Epidémiologie des Affections Parasitaires, Bamako	No. 25/FMPOS	Two sites conducted studies in 2006/2007. Manteorou conducted three cross-sectional studies during the transmission seasons of 2006/2007 and the intermittent dry season. Pongonon conducted a cross-sectional and a chloroquine efficacy study during the transmission seasons of 2006/2007.
Burkina Faso	BF	Dr S. B. Sirima	Comite d'Ethique pour la Recherche en Sante, Ministere de la Sante	Centre National de Recherché et de Formation sur le Paludisme	No. 2007-048	Five cross-sectional studies during two consecutive rainy seasons (2007/2008) and the intermittent dry season.
Sudan	SD	Professor M. E. Ibrahim	Ethics Committee, Institute of Endemic Diseases, University of Khartoum	Institute of Endemic Diseases, University of Khartoum	IEND/07/04	Longitudinal studies during transmission seasons of 2007/2008, and cross-sectional surveys before and after each season, as well as in the intermittent dry season.
Kenya	KE	Professor T. N. Williams	KEMRI/ National Ethics Review Committee	Kenya Medical Research Institute	KEMRI/RES/7/3/1<br>SSC NO. 1484	Birth cohort study; longitudinal data provided from birth (1992-1995) until sample date ~7 years later.
Tanzania (Moshi)	TZ	Professor C. Drakeley/ Professor E. Riley	Research Ethics Committee KCMC/ Ethics Committee LSHTM	Tumaini University, Kilimanjaro Christian Medical College/ London School of Hygiene & Tropical Medicine	- / 4093	Two cross-sectional surveys: one during the short rains in 2001 and one during the long rains in 2002.
Tanzania (Tanga SP1)	TZ	Dr M. Lemnge	Medical Research Coordinating Committee NIMR	National Institute for Medical Research, Dar as Salaam	NIMR/HQ/R.8a/VOl.IX/529	Cross-sectional studies at the end of two consecutive rainy seasons (2006/2007).
Tanzania (Tanga SP2)	TZ	Dr M. Lemnge	Medical Research Coordinating Committee NIMR	National Institute for Medical Research, Dar as Salaam	NIMR/HQ/R.8a/VOl.IX/529	Cross-sectional survey at the end of the long rainy season of 2004.
Sri Lanka	LK	Professor N. D. Karunweera	Ethical Review Committee, Faculty of Medicine, University of Colombo	Department of Parasitology, Faculty of Medicine, University of Colombo	EC/06/044	Cohort study with active case detective during 1992/1993; follow-up in 2006/2007 to collect samples.
