# Supplementary material for: Genetic determinants of anti-malarial acquired immunity in a large multi-centre study
Source: Malar J. 2015 Aug 28;14:333. doi: 10.1186/s12936-015-0833-x (PMC4552443; doi:10.1186/s12936-015-0833-x)
Supplement: Additional file 2: — Additional Table ST2: Details of covariates adjusted for in linear regression analysis of non-genetic factors with logged antibody levels, their relevance to the study and their previous association(s) with malaria or anti-malarial antibodies. Information on the covariates used in this study as con-variates in the various analyses. [file 12936_2015_833_MOESM2_ESM.pdf]

ADDITIONAL FILE 2: SUPPLEMENTARY TABLES

Genetic Determinants Of Anti-Malarial Acquired Immunity In A Large Multi-Centre Study

Jennifer M.G. Shelton, Patrick Corran, Paul Risley, Nilupa Silva, Christina Hubbart, Anna Jeffreys, Kate Rowlands, Rachel Craik, Victoria Cornelius, Meike Hensmann, Sile Molloy, Nuno Sepulveda, Taane G. Clark, Gavin Band, Geraldine M. Clarke, Christopher C.A. Spencer, Angeliki Kerasidou, Susana Campino, Sarah Auburn, Adama Tall, Alioune Badara Ly, Odile Mercereau-Puijalon, Anavaj Sakuntabhai, Abdoulaye Djimde, Boubacar Maiga, Ousmane Toure, Ogobara Doumbo, Amagana Dolo, Marita Troye-Blomberg, Valentina D. Mangano, Frederica Verra, David Modiano, Edith Bougouma, Sodiomon B. Sirima, Muntaser Ibrahim, Ayman Hussain, Nahid Eid, Abier Elzein, Hiba Mohamed, Ahmed Elhassan, Ibrahim Elhassan, Thomas N. Williams, Carolyne Ndila, Alexander Macharia, Kevin Marsh, Alphaxard Manjurano, Hugh Reyburn, Martha Lemnge, Deus Ishengoma, Richard Carter, Nadira Karunaweera, Deepika Fernando, Rajika Dewasurendra, Christopher J. Drakeley, Eleanor M. Riley, Dominic P. Kwiatkowski, and Kirk A. Rockett, in collaboration with the MalariaGEN Consortium,

Corresponding authors Kirk A. Rockett and Dominic P. Kwiatkowski
Wellcome Trust Centre for Human Genetics, University of Oxford, Roosevelt Drive, Oxford, UK

This file contains **Additional Table ST2:** Details of covariates adjusted for in linear regression analysis of non-genetic factors with logged antibody levels, their relevance to the study and their previous association(s) with malaria or anti-malarial antibodies.

Additional Table ST2: Details of covariates adjusted for in linear regression analysis of non-genetic factors with logged antibody levels, their relevance to the study and their previous association(s) with malaria or anti-malarial antibodies.

Factor	Relevance to study	Association with malaria	Association with anti-malarial antibodies	Covariate adjusted for in analysis
Altitude	Altitudes range from sea level (0m) in Kilifi, Kenya to 1845m in the Kilimanjaro region of Tanzania.	• Prevalence and duration of infection decreased with altitude[1].<br>• Incidence of malaria infection 2.5 times higher in lowland compared to highland areas[2].<br>• Living at higher altitude found to be protective against malaria infection[3].	• Higher MSP-1 levels seen in lowland than in highland residents[4].	Village [categorical]
Topography	Senegal data collected from two villages; a small river is present in one (Dielmo) and not the other (Ndiop). Information not collected at other sites.	• Close proximity to vector breeding grounds (i.e. swamps, rivers, vegetation) increases malaria transmission[5].		Village [categorical]
Age	Age of study participants ranges from 0-96 years.	• Risk of infection and disease decreased with age[6].	• Anti-malarial IgG levels increase with age[6].	Age [categorical]:<br>- <1 year<br>- 1-2 years<br>- 2-5 years<br>- 5-15 years<br>- 15-30 years<br>- >30 years
Ethnicity	Study comprised of 76 different ethnic groups.	• Fulani less susceptible to disease than sympatric ethnic groups in Mali[7].	• Fulani have higher IgG and IgE levels against malaria antigen than sympatric ethnic groups in Mali[7].	Ethnicity [categorical]
Season	Samples collected in dry and rainy seasons.	• Malaria transmission higher in rainy season than dry season[8].	• Anti-malarial antibody levels higher in rainy season than dry season[9].	Month [categorical]
Site	Data collected from 10 different sites across Africa and Asia.	• Countries across Africa and Asia have inherently different malaria prevalence, which needs to be adjusted for.	• Countries across Africa and Asia may have inherent differences in anti-malarial antibody levels, which needs to be adjusted for.	Site [categorical]
Gender	Study comprises both male and female participants.	• Frequently higher malaria prevalence in males than females[10].	• Higher IgG levels to *P.falciparum* antigen seen in males aged >30 years compared to females[11].	Gender [categorical]

1. Baliraine FN, Afrane YA, Amenya DA, Bonizzoni M, Menge DM, Zhou G, Zhong D, Vardo-Zalik AM, Githeko AK, Yan G: **High prevalence of asymptomatic plasmodium falciparum infections in a highland area of western Kenya: a cohort study.** *J Infect Dis* 2009, **200**:66-74.
2. Maxwell CA, Chambo W, Mwaimu M, Magogo F, Carneiro IA, Curtis CF: **Variation of malaria transmission and morbidity with altitude in Tanzania and with introduction of alphacypermethrin treated nets.** *Malar J* 2003, **2**:28.
3. Graves P, Richards F, Ngondi J, Emerson P, Shargie E, Endeshaw T, Ceccato P, Ejigsemahu Y, Mosher A, Hailemariam A, Zerihun M, Teferi T, Ayele B, Mesele A, Yohannes G, Tilahun A, Gebre T: **Individual, household and environmental risk factors for malaria infection in Amhara, Oromia and SNNP regions of Ethiopia.** *Trans R Soc Trop Med Hyg* 2009, **103**:1211-1220.
4. Badu K, Afrane YA, Larbi J, Stewart VA, Waitumbi J, Angov E, Ong'echa JM, Perkins DJ, Zhou G, Githeko A, Yan G: **Marked variation in MSP-119 antibody responses to malaria in western Kenyan highlands.** *BMC Infect Dis* 2012, **12**:50.
5. Amek N, Bayoh N, Hamel M, Lindblade KA, Gimnig JE, Odhiambo F, Laserson KF, Slutsker L, Smith T, Vounatsou P: **Spatial and temporal dynamics of malaria transmission in rural Western Kenya.** *Parasit Vectors* 2012, **5**:86.
6. Snow RW, Omumbo JA, Lowe B, Molyneux CS, Obiero JO, Palmer A, Weber MW, Pinder M, Nahlen B, Obonyo C, et al: **Relation between severe malaria morbidity in children and level of Plasmodium falciparum transmission in Africa.** *Lancet* 1997, **349:**1650-1654.
7. Dolo A, Coulibaly M, Maiga B, Daou M, Arama C, Troye-Blomberg M, Doumbo O: **[Humoral immune anti-Plasmodium falciparum AMA1 and MSP1 response in two ethnic groups living in sympatry in Mali].** *Bull Soc Pathol Exot* 2012, **105:**364-369.
8. Noland GS, Jansen P, Vulule JM, Park GS, Ondigo BN, Kazura JW, Moormann AM, John CC: **Effect of transmission intensity and age on subclass antibody responses to Plasmodium falciparum pre-erythrocytic and blood-stage antigens.** *Acta Trop* 2015, **142:**47-56.
9. McGregor IA, Rowe DS, Wilson ME, Billewicz WZ: **Plasma immunoglobulin concentrations in an African (Gambian) community in relation to season, malaria and other infections and pregnancy.** *Clin Exp Immunol* 1970, **7:**51-74.
10. Incardona S, Vong S, Chiv L, Lim P, Nhem S, Sem R, Khim N, Doung S, Mercereau-Puijalon O, Fandeur T: **Large-scale malaria survey in Cambodia: Novel insights on species distribution and risk factors.** *Malar J* 2007, **6**:37.
11. Ettling MB, Thimasarn K, Krachaiklin S, Bualombai P: **Evaluation of malaria clinics in Maesot, Thailan: use of serology to asses coverage.** *Trans R Soc Trop Med Hyg* 1989, **83**:325-331.
