# Supplementary material for: Genetic determinants of anti-malarial acquired immunity in a large multi-centre study
Source: Malar J. 2015 Aug 28;14:333. doi: 10.1186/s12936-015-0833-x (PMC4552443; doi:10.1186/s12936-015-0833-x)
Supplement: Additional file 3: — Additional File LFST1A: Details of gene regions selected for genotyping and the SNP assay design details. This file contains a list of the genes from which SNPs were selected and typed for this study. Information is provided on the gene and its genomic location with respect to the Human Reference genome build GRCh37. Also provided is a table containing the assay design details for each SNP used in this study for the Agena Biosciences iPLEX genotyping platform. SNPs are grouped into multiplexes as assigned by the assay design software. [file 12936_2015_833_MOESM3_ESM.pdf]

ADDITIONAL FILE 3: LARGE FORMAT SUPPLEMENTARY TABLES

Genetic Determinants Of Anti-Malarial Acquired Immunity In A Large Multi-Centre Study

Jennifer M.G. Shelton, Patrick Corran, Paul Risley, Nilupa Silva, Christina Hubbart, Anna Jeffreys, Kate Rowlands, Rachel Craik, Victoria Cornelius, Meike Hensmann, Sile Molloy, Nuno Sepulveda, Taane G. Clark, Gavin Band, Geraldine M. Clarke, Christopher C.A. Spencer, Angeliki Kerasidou, Susana Campino, Sarah Auburn, Adama Tall, Alioune Badara Ly, Odile Mercereau-Puijalon, Anavaj Sakuntabhai, Abdoulaye Djimde, Boubacar Maiga, Ousmane Toure, Ogobara Doumbo, Amagana Dolo, Marita Troye-Blomberg, Valentina D. Mangano, Frederica Verra, David Modiano, Edith Bougouma, Sodiomon B. Sirima, Muntaser Ibrahim, Ayman Hussain, Nahid Eid, Abier Elzein, Hiba Mohamed, Ahmed Elhassan, Ibrahim Elhassan, Thomas N. Williams, Carolyne Ndila, Alexander Macharia, Kevin Marsh, Alphaxard Manjurano, Hugh Reyburn, Martha Lemnge, Deus Ishengoma, Richard Carter, Nadira Karunaweera, Deepika Fernando, Rajika Dewasurendra, Christopher J. Drakeley, Eleanor M. Riley, Dominic P. Kwiatkowski, and Kirk A. Rockett, in collaboration with the MalariaGEN Consortium,

Corresponding authors Kirk A. Rockett and Dominic P. Kwiatkowski
Wellcome Trust Centre for Human Genetics, University of Oxford, Roosevelt Drive, Oxford, UK

This file contains:

Additional File LFST1A: Details of gene regions selected for genotyping. Gene positions are with respect to Ensembl Release 75 and human genome build GRCh37(http://feb2014.archive.ensembl.org/index.html)

Additional File LFST1B: Details of the SNP assays and multiplexes (SNP groups) designed for the Sequenom (Agena Bioscience; http://agenabio.com/iplex-genotyping) iPLEX platform. Each multiplex is highlighted by colour and are separated by blank rows. SNPs are ordered according to multiplex and the universal extemsion primer (UEP) mass (Daltons). Headings with an orange background provide details of each SNP in the human genome, while headings with a grey background are part of the assay design file. Further details of the assay design file specification can be obtained from Sequenom (Agena Bioscience).

HUGO gene symbol	synonym	Gene name/details	gene transcription strand	chr	gene_region	SNPS per gene[a]. Initial 202 SNPs	Analysis 196 SNPs	Final 178 SNPs	Core MalariaGEN gens/SNPs[b]	Notes/ key reference
GBP7		guanylate binding protein 7 [Source:HGNC Symbol;Acc:29606]	-1	1	89597434-89641723	1	1	1	GBP7	1
DARC	duffy	Duffy blood group, atypical chemokine receptor [Source:HGNC Symbol;Acc:4035]	1	1	159173097-159176290	1	1	1	DARC	1
FCER1A		Fc fragment of IgE, high affinity I, receptor for; alpha polypeptide [Source:HGNC Symbol;Acc:3609]	1	1	159259504-159278014	1	1	1		Antibody receptor
FCGR2A		Fc fragment of IgG, low affinity IIa, receptor (CD32) [Source:HGNC Symbol;Acc:3616]	1	1	161475220-161493803	1	1	1		Antibody receptor
FCGR3B	CD16	Fc fragment of IgG, low affinity IIIb, receptor (CD16b) [Source:HGNC Symbol;Acc:3620]	-1	1	161592986-161601753	1	1	1		Antibody receptor
RGS2		regulator of G-protein signaling 2, 24kDa [Source:HGNC Symbol;Acc:9998]	1	1	192778169-192781403	1	**0**	**0**		G-protein signalling
IL10		interleukin 10 [Source:HGNC Symbol;Acc:5962]	-1	1	206940947-206945839	6	6	6	IL10	1
CR1		complement component (3b/4b) receptor 1 (Knops blood group) [Source:HGNC Symbol;Acc:2334]	1	1	207669492-207813992	2	2	2	CR1	1
IL1A		interleukin 1, alpha [Source:HGNC Symbol;Acc:5991]	-1	2	113531492-113542167	2	2	2	IL1A	1
IL1B		interleukin 1, beta [Source:HGNC Symbol;Acc:5992]	-1	2	113587328-113594480	1	1	1	IL1B	1
IL17RE		interleukin 17 receptor E [Source:HGNC Symbol;Acc:18439]	1	3	9944296-9958086	1	1	1	IL17RE	1
TLR9		toll-like receptor 9 [Source:HGNC Symbol;Acc:15633]	-1	3	52255096-52273183	2	2	2	TLR9	1
IL17RD		interleukin 17 receptor D [Source:HGNC Symbol;Acc:17616]	-1	3	57124010-57204334	1	1	1	IL17RD	1
TLR1		toll-like receptor 1 [Source:HGNC Symbol;Acc:11847]	-1	4	38792298-38858438	2	2	2	TLR1	1
TLR6		toll-like receptor 6 [Source:HGNC Symbol;Acc:16711]	-1	4	38825336-38858438	2	2	2	TLR6	1
IL8		interleukin 8 [Source:HGNC Symbol;Acc:6025]	1	4	74606223-74609433	1	1	1		cytokine
IL7R		interleukin 7 receptor [Source:HGNC Symbol;Acc:6024]	1	5	35852797-35879705	2	2	2		cytokine
C6		complement component 6 [Source:HGNC Symbol;Acc:1339]	-1	5	41142336-41261540	1	1	1	C6	1
TSLP		thymic stromal lymphopoietin [Source:HGNC Symbol;Acc:30743]	1	5	110405760-110413722	1	1	1		
IL3		interleukin 3 (colony-stimulating factor, multiple) [Source:HGNC Symbol;Acc:6011]	1	5	131396222-131398897	7	7	6		2
CSF2		colony stimulating factor 2 (granulocyte-macrophage) [Source:HGNC Symbol;Acc:2434]	1	5	131409483-131411859	5	5	4		2
P4HA2		prolyl 4-hydroxylase, alpha polypeptide II [Source:HGNC Symbol;Acc:8547]	-1	5	131527531-131631008	4	4	4		2
PDLIM4		PDZ and LIM domain 4 [Source:HGNC Symbol;Acc:16501]	1	5	131593364-131609147	4	4	3		2
SLC22A4		solute carrier family 22 (organic cation/zwitterion transporter), member 4 [Source:HGNC Symbol;Acc:10968]	1	5	131630136-131679899	8	8	5		2
SLC22A5		solute carrier family 22 (organic cation/carnitine transporter), member 5 [Source:HGNC Symbol;Acc:10969]	1	5	131705444-131731306	4	4	2		2
C5orf56	LOC441108	chromosome 5 open reading frame 56 [Source:HGNC Symbol;Acc:33838]	1	5	131746328-131811736	5	5	5		2
IRF1		interferon regulatory factor 1 [Source:HGNC Symbol;Acc:6116]	-1	5	131817301-131826490	5	5	5	IRF1	1,2
IL5		interleukin 5 (colony-stimulating factor, eosinophil) [Source:HGNC Symbol;Acc:6016]	-1	5	131877136-131892530	3	3	2		2
RAD50		RAD50 homolog (S. cerevisiae) [Source:HGNC Symbol;Acc:9816]	1	5	131891711-131980313	17	17	9		2
IL13		interleukin 13 [Source:HGNC Symbol;Acc:5973]	1	5	131991955-131996802	5	5	5	IL13	1,2
IL4		interleukin 4 [Source:HGNC Symbol;Acc:6014]	1	5	132009678-132018368	6	6	6	IL4	1,2
IL12B		interleukin 12B (natural killer cell stimulatory factor 2, cytotoxic lymphocyte maturation factor 2, p40) [Source:HGNC Symbol;Acc:5970]	-1	5	158741791-158757895	4	4	4		cytokine
LTA		lymphotoxin alpha [Source:HGNC Symbol;Acc:6709]	1	6	31527243-31529513	2	2	2	LTA	1
TNF		tumor necrosis factor [Source:HGNC Symbol;Acc:11892]	1	6	31555632-31557434	5	5	5	TNF	1
SLC44A4	CTL4	solute carrier family 44, member 4 [Source:HGNC Symbol;Acc:13941]	-1	6	31870631-31870874	1	1	1	CTL4	1
IL20RA		interleukin 20 receptor, alpha [Source:HGNC Symbol;Acc:6003]	-1	6	137321108-137366298	1	1	1	IL20RA	1
IFNGR1		interferon gamma receptor 1 [Source:HGNC Symbol;Acc:5439]	-1	6	137518621-137540586	1	1	1		cytokine receptor
NOD1		nucleotide-binding oligomerization domain containing 1 [Source:HGNC Symbol;Acc:16390]	-1	7	30464143-30518400	1	1	1	NOD1	1
GLI3		GLI family zinc finger 3 [Source:HGNC Symbol;Acc:4319]	-1	7	42000548-42277469	1	1	1		
HECW1-IT1	HECW	HECW1 intronic transcript 1 (non-protein coding) [Source:HGNC Symbol;Acc:41465]	1	7	43157495-43202786	2	2	2		
IKZF1		IKAROS family zinc finger 1 (Ikaros) [Source:HGNC Symbol;Acc:13176]	1	7	50343720-50472799	7	6	6		3
DDC		dopa decarboxylase (aromatic L-amino acid decarboxylase) [Source:HGNC Symbol;Acc:2719]	-1	7	50526134-50633154	24	24	24		3
CD36		CD36 molecule (thrombospondin receptor) [Source:HGNC Symbol;Acc:1663]	1	7	79998891-80308593	2	2	2	CD36	1
CFTR		cystic fibrosis transmembrane conductance regulator (ATP-binding cassette sub-family C, member 7) [Source:HGNC Symbol;Acc:1884]	1	7	117105838-117356025	1	1	1	CFTR	1
TLR4		toll-like receptor 4 [Source:HGNC Symbol;Acc:11850]	1	9	120466610-120479149	2	2	2	TLR4	1
ABO		ABO blood group (transferase A, alpha 1-3-N-acetylgalactosaminyltransferase; transferase B, alpha 1-3-galactosyltransferase) [Source:HGNC Symbol;Acc:79]	-1	9	136125788-136150617	2	2	2	ABO	1
HBB		hemoglobin, beta [Source:HGNC Symbol;Acc:4827]	-1	11	5246694-5250625	3	3	2	HBB	1
TRIM5		tripartite motif containing 5 [Source:HGNC Symbol;Acc:16276]	-1	11	5684425-5959849	1	1	1	TRIM5	1
MS4A2		membrane-spanning 4-domains, subfamily A, member 2 [Source:HGNC Symbol;Acc:7316]	1	11	59855734-59863444	1	1	1		
RTN3		reticulon 3 [Source:HGNC Symbol;Acc:10469]	1	11	63448918-63527363	1	1	1	RTN3	1
STAT6		signal transducer and activator of transcription 6, interleukin-4 induced [Source:HGNC Symbol;Acc:11368]	-1	12	57489191-57525922	5	5	5		IL4 inducer
IFNG		interferon, gamma [Source:HGNC Symbol;Acc:5438]	-1	12	68548548-68553527	5	5	5		cytokine
IL22		interleukin 22 [Source:HGNC Symbol;Acc:14900]	-1	12	68642022-68647387	5	5	5	IL22	1
SPTB		spectrin, beta, erythrocytic [Source:HGNC Symbol;Acc:11274]	-1	14	65213002-65346601	1	1	1	SPTB	1
ADCY9		adenylate cyclase 9 [Source:HGNC Symbol;Acc:240]	-1	16	4003388-4166186	2	2	2	ADCY9	1
IL4R		interleukin 4 receptor [Source:HGNC Symbol;Acc:6015]	1	16	27324989-27376099	1	1	1	IL4R	1
HP		haptoglobin [Source:HGNC Symbol;Acc:5141]	1	16	72088491-72094954	1	1	1		Haptoglobin
ADORA2B		adenosine A2b receptor [Source:HGNC Symbol;Acc:264]	1	17	15848231-15879060	1	1	1	ADORA2B	1
NOS2		nitric oxide synthase 2, inducible [Source:HGNC Symbol;Acc:7873]	-1	17	26083792-26127525	5	5	5	NOS2	1
EMR1		egf-like module containing, mucin-like, hormone receptor-like 1 [Source:HGNC Symbol;Acc:3336]	1	19	6887577-6940470	2	2	2	EMR1	1
FCER2		Fc fragment of IgE, low affinity II, receptor for (CD23) [Source:HGNC Symbol;Acc:3612]	-1	19	7753644-7767032	1	1	1		Antibody receptor
ICAM1		intercellular adhesion molecule 1 [Source:HGNC Symbol;Acc:5344]	1	19	10381511-10397291	2	2	2	ICAM1	1
GNAS		GNAS complex locus [Source:HGNC Symbol;Acc:4392]	1	20	57414773-57486247	2	1	1	GNAS	1
DERL3		derlin 3 [Source:HGNC Symbol;Acc:14236]	-1	22	24176690-24181315	1	1	1	DERL3	1
AMELX		amelogenin, X-linked [Source:HGNC Symbol;Acc:461]	1	X	11311533-11318881	3	**0**	**0**	AMELX	1, used for determining genetic gender

Additional File LFST1A: Details of gene regions selected for genotyping. Gene positions are with respect to Ensembl Release 75 and human genome build GRCh37(http://feb2014.archive.ensembl.org/index.html)

[a:] SNP multiplexes can be found in Table 2 below

[b:] A description of the selection and analysis of these SNPs in severe malaria can be found in reference 1.

Further details of the assaociations with malaria can be found here or from references cited in the main text:

1: Malaria Genomic Epidemiology Network. (2014) Reappraisal of known malaria resistance loci in a large multicenter study. **Nat Genet. 46:1197-204**. (http://www.ncbi.nlm.nih.gov/pubmed/25261933)

2: Rihet P1, Traoré Y, Abel L, Aucan C, Traoré-Leroux T, Fumoux F. (1998) Malaria in humans: Plasmodium falciparum blood infection levels are linked to chromosome 5q31-q33. **Am. J. Hum. Genet. 63:498-505**. (http://www.ncbi.nlm.nih.gov/pubmed/9683598)

3: Jallow M, *et al.* (2008) Genome-wide and fine-resolution association analysis of malaria in West Africa. **Nat Genet. 41:657-65**. (http://www.ncbi.nlm.nih.gov/pubmed/19465909)

SNP_ID	chr_valid	coord_valid	rsnumber	gene_symbol	genome_build	ensembl_version	strand	single_letter_code	reference_allele	full_202	analysis_196	final_178	WELL	term	SNP_ID	1stPCRP	2ndPCRP	amp_len	up_conf	mp_conf	tmgc	pcgc	pwarn	uep_dir	uep_mass	UEPseq	ext1_call	ext1_mass	ext1_seq	ext2_call	ext2_mass	ext2_seq
12-72251611	12	68648622	rs2227478	IL22	dbSNP137/GRch37	73	1	R	G	1	1	1	W1	iPLEX	12-72251611	ACGTTGGATGTGAGATGGCACAGACCTAAG	ACGTTGGATGTCTGGCCACCTTCACAAATG	104	99.9	70.5	48	60		R	4534.9	GTGGTTCCGACCCTT	G	4782.1	GTGGTTCCGACCCTTC	A	4862	GTGGTTCCGACCCTTT
hTNF-238	6	31543101	rs361525	TNF	dbSNP137/GRch37	73	1	R	G	1	1	1	W1	iPLEX	hTNF-238	ACGTTGGATGCAGGGTCCTACACACAAATC	ACGTTGGATGAAGCATCAAGGATACCCCTC	115	99.1	70.3	52	68.8	d	R	4689	CCCATCCTCCCTGCTC	G	4936.2	CCCATCCTCCCTGCTCC	A	5016.1	CCCATCCTCCCTGCTCT
hICAM-1codon469	19	10395683	rs5498	ICAM1	dbSNP137/GRch37	73	1	R	A	1	1	1	W1	iPLEX	hICAM-1codon469	ACGTTGGATGTGTCACTCGAGATCTTGAGG	ACGTTGGATGACTCACAGAGCACATTCACG	115	99.1	70.3	48	50	Sd	R	4801.1	ACATTCACGGTCACCT	G	5048.3	ACATTCACGGTCACCTC	A	5128.2	ACATTCACGGTCACCTT
hIL-10-3533	1	206949365	rs1800890	IL10	dbSNP137/GRch37	73	1	W	A	1	1	1	W1	iPLEX	hIL-10-3533	ACGTTGGATGCAAGCCCAGATGCATAGTAG	ACGTTGGATGCTGATTTCCCAGTACATCCC	110	99.6	70.5	46	50	D	R	4819.2	CCCCCACTGGAAAAAT	T	5090.4	CCCCCACTGGAAAAATA	A	5146.3	CCCCCACTGGAAAAATT
hIL-10-1082	1	206946897	rs1800896	IL10	dbSNP137/GRch37	73	1	Y	T	1	1	1	W1	iPLEX	hIL-10-1082	ACGTTGGATGCCAAGACAACACTACTAAGG	ACGTTGGATGATTCCATGGAGGCTGGATAG	111	98.1	70.5	47	58.8		F	4977.2	CCTATCCCTACTTCCCC	C	5224.4	CCTATCCCTACTTCCCCC	T	5304.3	CCTATCCCTACTTCCCCT
amelogenin_XY_SNP6	X	11316650		AMELX	dbSNP137/GRch37	73	1	M	C	1	0	0	W1	iPLEX	amelogenin_XY_SNP6	ACGTTGGATGGGGCTCGTAACCATAGGAAG	ACGTTGGATGGCTTCTCTGGTTGGAGTCAC	103	97.1	70.5	49	52.9	d	F	5179.4	GCCAATGGTAAACCTGC	C	5426.6	GCCAATGGTAAACCTGCC	A	5450.6	GCCAATGGTAAACCTGCA
GNAS_8386	20	57485812	rs8386	GNAS	dbSNP137/GRch37	73	1	Y	C	1	1	1	W1	iPLEX	GNAS_8386	ACGTTGGATGTCATTTCACCTGCGCTGTGG	ACGTTGGATGTGCATGCGCTGAATGATGTC	98	98.5	70.5	52	56.3	D	R	5244.4	gTGAACACACGGCGGAT	T	5515.6	gTGAACACACGGCGGATA	C	5531.6	gTGAACACACGGCGGATG
rs17411697	2	113537223	rs17561	IL1A	dbSNP137/GRch37	73	-1	K	G	1	1	1	W1	iPLEX	rs17411697	ACGTTGGATGTTTCACATTGCTCAGGAAGC	ACGTTGGATGATCTGCACTTGTGATCATGG	96	85.9	70.5	48	44.4	Dh	F	5467.6	ATCATCAAGCCTAGGTCA	G	5754.8	ATCATCAAGCCTAGGTCAG	T	5794.7	ATCATCAAGCCTAGGTCAT
rs9282799	17	26128728	rs9282799	NOS2	dbSNP137/GRch37	73	-1	Y	C	1	1	1	W1	iPLEX	rs9282799	ACGTTGGATGTGGACAGTGGTAGCAAAGTG	ACGTTGGATGTTAGGGAGAAGTTGAGAAGC	98	98.5	70.5	48	52.9	d	R	5649.7	ttTCACCCTTGATCTCACC	T	5920.9	ttTCACCCTTGATCTCACCA	C	5936.9	ttTCACCCTTGATCTCACCG
hHbS	11	5248232	rs334	HBB	dbSNP137/GRch37	73	-1	W	A	1	1	1	W1	iPLEX	hHbS	ACGTTGGATGTCAAACAGACACCATGGTGC	ACGTTGGATGCAACTTCATCCACGTTCACC	106	99.9	70.5	49	52.9	d	R	5723.7	ctTAACGGCAGACTTCTCC	T	5994.9	ctTAACGGCAGACTTCTCCA	A	6050.8	ctTAACGGCAGACTTCTCCT
hTNF-376	6	31542963	rs1800750	TNF	dbSNP137/GRch37	73	1	R	G	1	1	1	W1	iPLEX	hTNF-376	ACGTTGGATGCTCCCAGTTCTAGTTCTATC	ACGTTGGATGCCAGGTCTGTGGTCTGTTTC	91	96.8	70.5	47	42.1		R	5775.8	GTGGTCTGTTTCCTTCTAA	G	6022.9	GTGGTCTGTTTCCTTCTAAC	A	6102.9	GTGGTCTGTTTCCTTCTAAT
rs229587	14	65263300	rs229587	SPTB	dbSNP137/GRch37	73	1	Y	T	1	1	1	W1	iPLEX	rs229587	ACGTTGGATGAAGGCCGCAATGAGAGAGAC	ACGTTGGATGCAAAGCATGTCCCTCATTAC	94	97	70.5	56	57.9	D	F	5819.8	CACGAGGCGCTGGTTTTCA	C	6067	CACGAGGCGCTGGTTTTCAC	T	6146.9	CACGAGGCGCTGGTTTTCAT
IL10_232424450	1	206940831	rs3024500	IL10	dbSNP137/GRch37	73	1	R	A	1	1	1	W1	iPLEX	IL10_232424450	ACGTTGGATGTCTCTGATCAGGTTTGGAGC	ACGTTGGATGGAGTGGGTGAATGAATTCTG	111	98.1	70.5	52	68.8	d	R	5967.9	ccctGAAGCCAGCTACCCCC	G	6215.1	ccctGAAGCCAGCTACCCCCC	A	6295	ccctGAAGCCAGCTACCCCCT
12-72250702	12	68647713	rs2227485	IL22	dbSNP137/GRch37	73	1	R	G	1	1	1	W1	iPLEX	12-72250702	ACGTTGGATGGTTTTGTCTTAGTAGAGTTC	ACGTTGGATGCTGGTAGGAAAATGAGTCCG	116	86.9	70.5	48	40	D	R	6085	TGACCAAAATGCTTACTCAG	G	6332.2	TGACCAAAATGCTTACTCAGC	A	6412.1	TGACCAAAATGCTTACTCAGT
hLT-alpha_NcoI	6	31540313	rs909253	LTA	dbSNP137/GRch37	73	-1	Y	T	1	1	1	W1	iPLEX	hLT-alpha_NcoI	ACGTTGGATGGTCAGAGAAACCCCAAGGTG	ACGTTGGATGTCTCTGTCACACATTCTCTG	115	92	70.5	49	45	DH	R	6314.1	cCATTCTCTGTTTCTGCCATG	T	6585.3	cCATTCTCTGTTTCTGCCATGA	C	6601.3	cCATTCTCTGTTTCTGCCATGG
hCD36_T1264G	7	80300449	rs3211938	CD36	dbSNP137/GRch37	73	1	K	T	1	1	1	W1	iPLEX	hCD36_T1264G	ACGTTGGATGCTCCAGTTGAAAACCCAGAC	ACGTTGGATGTTGCATTTGCTGATGTCTAG	117	87.4	70.5	47	52.9	dh	R	6372.2	gtttCTGATGTCTAGCACACC	G	6619.3	gtttCTGATGTCTAGCACACCC	T	6643.4	gtttCTGATGTCTAGCACACCA
hICAM-1codon241	19	10394792	rs1799969	ICAM1	dbSNP137/GRch37	73	1	R	G	1	1	1	W1	iPLEX	hICAM-1codon241	ACGTTGGATGAACCTCTGGTCCCCCAGTGC	ACGTTGGATGTCCTAGAGGTGGACACGCAG	120	54.4	70.5	53	61.1	DH	F	6389.1	ccaGTGGTCTGTTCCCTGGAC	A	6660.3	ccaGTGGTCTGTTCCCTGGACA	G	6676.4	ccaGTGGTCTGTTCCCTGGACG
12-72249510	12	68646521	rs2227491	IL22	dbSNP137/GRch37	73	1	Y	T	1	1	1	W1	iPLEX	12-72249510	ACGTTGGATGTTCCACGGAGTCAGTGTAAG	ACGTTGGATGATCACCACCACCCCAAGTAC	98	89.3	70.5	61	76.5	Dh	F	6458.2	gggcTGGACGGCACACGGCCC	C	6705.4	gggcTGGACGGCACACGGCCCC	T	6785.3	gggcTGGACGGCACACGGCCCT
hTNF_plus851	6	31544189	rs3093662	TNF	dbSNP137/GRch37	73	1	R	A	1	1	1	W1	iPLEX	hTNF_plus851	ACGTTGGATGGGAAAGAGCTGTTGAATGCC	ACGTTGGATGGTGTCTGGTTTTCTCTCTCC	84	99	70.5	46	40	d	R	6568.31	ttGTTTTCTCTCTCCATTCATC	G	6815.5	ttGTTTTCTCTCTCCATTCATCC	A	6895.4	ttGTTTTCTCTCTCCATTCATCT
rs17047661	1	207782889	rs17047661	CR1	dbSNP137/GRch37	73	1	R	A	1	1	1	W1	iPLEX	rs17047661	ACGTTGGATGATGCACAGCTCCAGAAGTTG	ACGTTGGATGCCCGGGCTGACATCTAAATC	117	97.4	70.5	49	52.6	D	R	6925.5	aaacCTCCTGTTTCCTGGTACTC	G	7172.7	aaacCTCCTGTTTCCTGGTACTCC	A	7252.6	aaacCTCCTGTTTCCTGGTACTCT
hCD36_G1439C	7	80302110	rs201346212	CD36	dbSNP137/GRch37	73	1	S	G	1	1	1	W1	iPLEX	hCD36_G1439C	ACGTTGGATGTGACCAATAGGTTGACCTGC	ACGTTGGATGGTTCTCTTTTTAGATAACTGG	88	83.2	70.5	47	26.1		F	6987.6	TAACTGGATTCACTTTACAATTT	C	7234.8	TAACTGGATTCACTTTACAATTTC	G	7274.8	TAACTGGATTCACTTTACAATTTG
rs4986791	9	120475602	rs4986791	TLR4	dbSNP137/GRch37	73	1	Y	C	1	1	1	W1	iPLEX	rs4986791	ACGTTGGATGGGTAATAACACCATTGAAGC	ACGTTGGATGAGGTTGCTGTTCTCAAAGTG	96	92.9	70.5	52	34.8	dH	F	7077.6	TTCTCAAAGTGATTTTGGGACAA	C	7324.8	TTCTCAAAGTGATTTTGGGACAAC	T	7404.7	TTCTCAAAGTGATTTTGGGACAAT
rs1555498	6	137325847	rs1555498	IL20RA	dbSNP137/GRch37	73	1	Y	C	1	1	1	W1	iPLEX	rs1555498	ACGTTGGATGCAATCATCAGAGTTCAAGGC	ACGTTGGATGGATGGAATAGCCCATCACAG	113	97.9	70.5	46	26.1		F	7130.7	AAAAAGAAACACGGTAATAGATA	C	7377.9	AAAAAGAAACACGGTAATAGATAC	T	7457.8	AAAAAGAAACACGGTAATAGATAT
hTNFa-1031	6	31542308	rs1799964	TNF	dbSNP137/GRch37	73	1	Y	T	1	1	1	W1	iPLEX	hTNFa-1031	ACGTTGGATGGGGAAGCAAAGGAGAAGCTG	ACGTTGGATGTACATGTGGCCATATCTCCC	112	98	70.5	57	54.2	s	R	7150.6	CCTCCAGACCCTGACTTTTCCTTC	T	7421.9	CCTCCAGACCCTGACTTTTCCTTCA	C	7437.9	CCTCCAGACCCTGACTTTTCCTTCG
LTA_plus77	6	31540141	rs2239704	LTA	dbSNP137/GRch37	73	-1	K	G	1	1	1	W1	iPLEX	LTA_plus77	ACGTTGGATGAGGTGCAGGAGGGACCGAG	ACGTTGGATGGTGCTTCGTGCTTTGGACTA	91	80.5	70.5	59	65	D	R	7296.7	gctgCGTGCTTTGGACTACCGCCC	G	7543.9	gctgCGTGCTTTGGACTACCGCCCC	T	7567.9	gctgCGTGCTTTGGACTACCGCCCA
rs2297518	17	26096597	rs2297518	NOS2	dbSNP137/GRch37	73	1	R	G	1	1	1	W1	iPLEX	rs2297518	ACGTTGGATGTTGTTGTTGAGCTCTTTCAG	ACGTTGGATGTTGAGAACTCTGTCATTCCC	95	92.9	70.5	47	30.4		R	7359.8	gCTCTTTCTAGAAACTGAAGAAAT	G	7607	gCTCTTTCTAGAAACTGAAGAAATC	A	7686.9	gCTCTTTCTAGAAACTGAAGAAATT
12-72247607	12	68644618	rs1012356	IL22	dbSNP137/GRch37	73	1	W	A	1	1	1	W1	iPLEX	12-72247607	ACGTTGGATGGCTTACCAATTCAGACTTCC	ACGTTGGATGCCCCGATCTCTTTTATACAG	107	92.7	70.5	49	34.8		F	7510.9	tcCCAATTCAGACTTCCATTTAACT	A	7782.1	tcCCAATTCAGACTTCCATTTAACTA	T	7838	tcCCAATTCAGACTTCCATTTAACTT
TLR9_rs187084	3	52261031	rs187084	TLR9	dbSNP137/GRch37	73	-1	Y	C	1	1	1	W1	iPLEX	TLR9_rs187084	ACGTTGGATGTATTCCCCTGCTGGAATGTC	ACGTTGGATGTGCTGGGCACTGTACTGGAT	100	91.8	70.5	56	44	ds	F	7659	GATGCAGATAAAAGATCACTGCCCT	C	7906.2	GATGCAGATAAAAGATCACTGCCCTC	T	7986.1	GATGCAGATAAAAGATCACTGCCCTT
rs2230739	16	4033436	rs2230739	ADCY9	dbSNP137/GRch37	73	-1	R	A	1	1	1	W1	iPLEX	rs2230739	ACGTTGGATGGAAGGTGGGACTAGCAAACG	ACGTTGGATGTCCCAGTCATTCTGCTCCAC	100	97.1	70.5	59	56.5	d	F	7811.1	ctaCACTCCTGCTCCACACAGGTCAT	A	8082.3	ctaCACTCCTGCTCCACACAGGTCATA	G	8098.3	ctaCACTCCTGCTCCACACAGGTCATG
rs2814778	1	159174683	rs2814778	DARC	dbSNP137/GRch37	73	-1	R	A	1	1	1	W1	iPLEX	rs2814778	ACGTTGGATGAGACAGAAGGGCTGGGACG	ACGTTGGATGAACCTGATGGCCCTCATTAG	97	89.3	70.5	60	50		R	7879.1	TGGCCCTCATTAGTCCTTGGCTCTTA	G	8126.3	TGGCCCTCATTAGTCCTTGGCTCTTAC	A	8206.2	TGGCCCTCATTAGTCCTTGGCTCTTAT
rs2535611	17	15861332	rs2535611	ADORA2B	dbSNP137/GRch37	73	1	Y	T	1	1	1	W1	iPLEX	rs2535611	ACGTTGGATGGAGTGAAACTCTGTCTCAAG	ACGTTGGATGCGACATTGTAGAACCAGGAC	92	98.3	70.5	57	56.5	D	F	7932.1	ggcCCAGGACCTGTCAGGTACACTTC	C	8179.3	ggcCCAGGACCTGTCAGGTACACTTCC	T	8259.2	ggcCCAGGACCTGTCAGGTACACTTCT
GNAS_2057291	20	57472043	rs2057291	GNAS	dbSNP137/GRch37	73	1	R	G	1	0	0	W1	iPLEX	GNAS_2057291	ACGTTGGATGTTCAGAGCAAGGCAGGCAGC	ACGTTGGATGGATGTCATCCTTCTCTAAAC	100	83.8	70.5	48	26.9	h	R	8232.4	gTTGATCTAACAAATTCTAGAATTCTC	G	8479.6	gTTGATCTAACAAATTCTAGAATTCTCC	A	8559.5	gTTGATCTAACAAATTCTAGAATTCTCT
rs4986790	9	120475302	rs4986790	TLR4	dbSNP137/GRch37	73	1	R	A	1	1	1	W1	iPLEX	rs4986790	ACGTTGGATGAGCATACTTAGACTACTACC	ACGTTGGATGCACCAGGGAAAATGAAGAAAC	99	89.2	70.5	45	18.5		R	8291.5	GTCAAACAATTAAATAAGTCAATAATA	G	8538.7	GTCAAACAATTAAATAAGTCAATAATAC	A	8618.6	GTCAAACAATTAAATAAGTCAATAATAT
IL4R-63011	16	27374180	rs1805015	IL4R	dbSNP137/GRch37	73	1	Y	T	1	1	1	W1	iPLEX	IL4R-63011	ACGTTGGATGTCTCTGGGACACGGTGACTG	ACGTTGGATGACCTGACTTGCACAGAGACG	114	92.3	70.5	64	57.1		F	8438.5	CAACCCTGCTTACCGCAGCTTCAGCAAC	C	8685.7	CAACCCTGCTTACCGCAGCTTCAGCAACC	T	8765.6	CAACCCTGCTTACCGCAGCTTCAGCAACT
hTNF-308	6	31543031	rs1800629	TNF	dbSNP137/GRch37	73	1	R	G	1	1	1	W2	iPLEX	hTNF-308	ACGTTGGATGGGAGGCAATAGGTTTTGAGG	ACGTTGGATGCTGATTTGTGTGTAGGACCC	86	99.2	73.9	52	73.3	D	R	4513.9	GGCTGAACCCCGTCC	G	4761.1	GGCTGAACCCCGTCCC	A	4841	GGCTGAACCCCGTCCT
rs2075820	7	30492237	rs2075820	NOD1	dbSNP137/GRch37	73	-1	R	G	1	1	1	W2	iPLEX	rs2075820	ACGTTGGATGAAGCGCAGCAGGAAGGCAAAC	ACGTTGGATGACCTGCTCTTCAAGCACTAC	90	92.5	73.9	55	80	DsH	F	4861.2	aCCAGAGCGGGACCCC	A	5132.4	aCCAGAGCGGGACCCCA	G	5148.4	aCCAGAGCGGGACCCCG
rs2242665	6	31839309	rs2242665	CTL4	dbSNP137/GRch37	73	-1	R	G	1	1	1	W2	iPLEX	rs2242665	ACGTTGGATGGGCTCTCCCTTCCAGCTCT	ACGTTGGATGTGGTGGTGTCATTGGTGATC	107	79.8	73.9	54	62.5	h	R	4971.2	AGCGCCGGTGGAGTAA	G	5218.4	AGCGCCGGTGGAGTAAC	A	5298.3	AGCGCCGGTGGAGTAAT
amelogenin_XY_SNP1	X	11313735		AMELX	dbSNP137/GRch37	73	1	R	G	1	0	0	W2	iPLEX	amelogenin_XY_SNP1	ACGTTGGATGTGATACAACCAGAAGCCAGC	ACGTTGGATGCTTGGTTTTGTGGGTGAGAG	89	99.5	73.9	46	56.3	D	R	5093.3	AGAGGAAGAGAGGAGG	G	5340.5	AGAGGAAGAGAGGAGGC	A	5420.4	AGAGGAAGAGAGGAGGT
TLR9_rs352140_exonic	3	52231737	rs352140	TLR9	dbSNP137/GRch37	73	-1	R	A	1	1	1	W2	iPLEX	TLR9_rs352140_exonic	ACGTTGGATGTGGCTGTTGTAGCTGAGGTC	ACGTTGGATGATAAGCTGGACCTCTACCAC	99	98.6	73.9	47	56.3	D	R	5115.3	cCATTCACGGAGCTACC	T	5386.5	cCATTCACGGAGCTACCA	C	5402.5	cCATTCACGGAGCTACCG
hNOS2-954	17	26128509	rs1800482	NOS2	dbSNP137/GRch37	73	-1	S	G	1	1	1	W2	iPLEX	hNOS2-954	ACGTTGGATGGGGCAGATCACTTGAGCTTC	ACGTTGGATGCCACCACACCCAGCTAATAT	119	94.7	73.9	49	50	D	R	5198.4	tTGTTGTCCATGCTGGT	G	5445.6	tTGTTGTCCATGCTGGTC	C	5485.6	tTGTTGTCCATGCTGGTG
rs6780995	3	57138419	rs6780995	IL17RD	dbSNP137/GRch37	73	1	R	G	1	1	1	W2	iPLEX	rs6780995	ACGTTGGATGTCCCCTGGAGAAACATTTTG	ACGTTGGATGGTTCTTTGGTCTGTTGCAGG	92	94	73.9	46	47.1	d	R	5261.5	GGAGCAAACTACAGAGA	G	5508.6	GGAGCAAACTACAGAGAC	A	5588.6	GGAGCAAACTACAGAGAT
rs542998	11	63487386	rs542998	RTN3	dbSNP137/GRch37	73	1	Y	T	1	1	1	W2	iPLEX	rs542998	ACGTTGGATGACTCTTTGGGTTCTGGAGTG	ACGTTGGATGCTCTGCCAGTCCATTTCATC	99	100	73.9	46	47.1	d	R	5274.4	GGTGGTCATCAGGTAAA	T	5545.7	GGTGGTCATCAGGTAAAA	C	5561.7	GGTGGTCATCAGGTAAAG
rs33950507	11	5248173	rs33950507	HBB	dbSNP137/GRch37	73	-1	D	G	1	1	0	W2	iPLEX	rs33950507	ACGTTGGATGCAAGGTGAACGTGGATGAAG	ACGTTGGATGGTCTCCTTAAACCTGTCTTG	98	98.5	73.9	46	50	Ds	F	5361.5	gGGATGAAGTTGGTGGT	A	5632.7	gGGATGAAGTTGGTGGTA	G	5648.7	gGGATGAAGTTGGTGGTG
rs17047660	1	207782856	rs17047660	CR1	dbSNP137/GRch37	73	1	R	A	1	1	1	W2	iPLEX	rs17047660	ACGTTGGATGATCAAGTTGGTGTTTGGAGC	ACGTTGGATGGCATTTCAACTTCTGGAGC	99	97.1	73.9	46	53.3	d	R	5466.6	cccCTGGAGCTGTGCATT	G	5713.7	cccCTGGAGCTGTGCATTC	A	5793.6	cccCTGGAGCTGTGCATTT
rs461645	19	6919753	rs461645	EMR1	dbSNP137/GRch37	73	-1	Y	T	1	1	1	W2	iPLEX	rs461645	ACGTTGGATGGTTCCCAACAGGTAGACAAG	ACGTTGGATGAAAGACGGCTTCTCAGATCC	115	99.1	73.9	45	44.4	D	R	5467.6	ATCTACACTCTGGAGAAC	T	5738.8	ATCTACACTCTGGAGAACA	C	5754.8	ATCTACACTCTGGAGAACG
hNOS2-1659	17	26129212	rs8078340	NOS2	dbSNP137/GRch37	73	-1	Y	C	1	1	1	W2	iPLEX	hNOS2-1659	ACGTTGGATGTCTGTAGTGATGGAGGGATG	ACGTTGGATGGTGTCCTCTCCCTTGTAAAC	101	100	73.9	46	47.1	d	R	5525.6	aCTTGAACAAGGCAGAAC	C	5772.8	aCTTGAACAAGGCAGAACC	T	5852.7	aCTTGAACAAGGCAGAACT
rs7537937	1	89582690	rs1803632	GBP7	dbSNP137/GRch37	73	1	S	G	1	1	1	W2	iPLEX	rs7537937	ACGTTGGATGTCCTCAGCCATTCCAATGTC	ACGTTGGATGTAGAGAGAGTGACTCACGAG	94	98.7	73.9	53	61.1	d	F	5813.8	gCACGAGGCCCATTGACTG	C	6061	gCACGAGGCCCATTGACTGC	G	6101	gCACGAGGCCCATTGACTGG
hIL-4-589	5	132009154	rs2243250	IL4	dbSNP137/GRch37	73	1	Y	C	1	1	1	W2	iPLEX	hIL-4-589	ACGTTGGATGTAACAGGCAGACTCTCCTAC	ACGTTGGATGTGATACGACCTGTCCTTCTC	102	100	73.9	47	38.9	DH	F	5891.9	gAACTTGGGAGAACATTGT	C	6139	gAACTTGGGAGAACATTGTC	T	6218.9	gAACTTGGGAGAACATTGTT
amelogenin_XY_SNP2	X	11316106		AMELX	dbSNP137/GRch37	73	1	Y	T	1	0	0	W2	iPLEX	amelogenin_XY_SNP2	ACGTTGGATGCAGTCAAGTTAATGAATCTC	ACGTTGGATGCTTATATGCTAGGAGGGCA	103	67.7	73.9	48	44.4	h	R	5907.9	gTGCTAGGAGGCAAGTATT	T	6179.1	gTGCTAGGAGGCAAGTATTA	C	6195.1	gTGCTAGGAGGCAAGTATTG
rs2179652	1	192769826	rs2179652	RGS2	dbSNP137/GRch37	73	1	Y	T	1	0	0	W2	iPLEX	rs2179652	ACGTTGGATGTGGTGGAATCTGTATTGAGG	ACGTTGGATGGCAAGTCTGTAGATTCTAGC	95	97	73.9	49	50	Dh	F	6018.9	ctTTTCATAGTCCAGCCCTG	C	6266.1	ctTTTCATAGTCCAGCCCTGC	T	6346	ctTTTCATAGTCCAGCCCTGT
5-131349023	5	131826880	rs2706384	IRF1	dbSNP137/GRch37	73	-1	M	C	1	1	1	W2	iPLEX	5-131349023	ACGTTGGATGGGTATATCTCCCGAACGCAG	ACGTTGGATGGAGATTCGGCCCAGTGTTC	99	94.6	73.9	57	70.6	dH	F	6118	attCAAGTGCCCGGGCGATC	C	6365.2	attCAAGTGCCCGGGCGATCC	A	6389.2	attCAAGTGCCCGGGCGATCA
rs1801033	5	41199959	rs1801033	C6	dbSNP137/GRch37	73	-1	M	A	1	1	1	W2	iPLEX	rs1801033	ACGTTGGATGGGAATGCATGGTTGAAAGGC	ACGTTGGATGGTTAGATCTGTCTTGCGTCC	100	99	73.9	54	64.7	dH	F	6158	tgtGGGACAGCCATGCACTG	C	6405.2	tgtGGGACAGCCATGCACTGC	A	6429.2	tgtGGGACAGCCATGCACTGA
rs708567	3	9960070	rs708567	IL17RE	dbSNP137/GRch37	73	-1	R	G	1	1	1	W2	iPLEX	rs708567	ACGTTGGATGGTTCTCCTCACCATTCCTAG	ACGTTGGATGGAGGAAAAGTTTGGAGGAGC	83	92.6	73.9	53	58.8	Dh	R	6247.1	aggTGGAGGAGCAGCTGACT	G	6494.2	aggTGGAGGAGCAGCTGACTC	A	6574.2	aggTGGAGGAGCAGCTGACTT
rs7935564	11	5718517	rs7935564	TRIM5	dbSNP137/GRch37	73	1	R	G	1	1	1	W2	iPLEX	rs7935564	ACGTTGGATGACAGGAAAAGCTGCAGGTAG	ACGTTGGATGTTCCAGGCGGTTCTCTCTTG	119	97.1	73.9	49	50		R	6284.1	cccGCTTCTCAGCCTCTTGAT	G	6531.3	cccGCTTCTCAGCCTCTTGATC	A	6611.2	cccGCTTCTCAGCCTCTTGATT
rs1143634	2	113590390	rs1143634	IL1B	dbSNP137/GRch37	73	-1	Y	C	1	1	1	W2	iPLEX	rs1143634	ACGTTGGATGCAGTTCAGTGATCGTACAGG	ACGTTGGATGGTGCTCCACATTTCAGAACC	106	99.9	73.9	46	33.3	D	F	6306.1	CATTTCAGAACCTATCTTCTT	C	6553.3	CATTTCAGAACCTATCTTCTTC	T	6633.2	CATTTCAGAACCTATCTTCTTT
rs8176746	9	136131322	rs8176746	ABO	dbSNP137/GRch37	73	-1	M	C	1	1	1	W2	iPLEX	rs8176746	ACGTTGGATGTTGCACCGACCCCCCGAAGAA	ACGTTGGATGCCCAGTCCCAGGCCTACAT	97	75.3	73.9	52	50		F	6695.4	ccACGAGGGCGATTTCTACTAC	C	6942.5	ccACGAGGGCGATTTCTACTACC	A	6966.6	ccACGAGGGCGATTTCTACTACA
rs17140229	7	117230283	rs17140229	CFTR	dbSNP137/GRch37	73	1	Y	T	1	1	1	W2	iPLEX	rs17140229	ACGTTGGATGACAATATGGTCACCACATCG	ACGTTGGATGAAGTGCTACTTCTGCACCAC	88	98	73.9	48	31.8		F	6748.4	ACCACTTTTGAGAATAGTGTTA	C	6995.6	ACCACTTTTGAGAATAGTGTTAC	T	7075.5	ACCACTTTTGAGAATAGTGTTAT
rs3177244	22	24179132	rs1128127	DERL3	dbSNP137/GRch37	73	1	R	G	1	1	1	W2	iPLEX	rs3177244	ACGTTGGATGGGCAGGTAATTGGGGTCTTC	ACGTTGGATGTGTGCTGGCCTGTGCTCAAC	103	93	73.9	55	52.4	Dg	F	6768.4	aACAGAAAGCTGCTCCTGGATG	G	7015.6	aACAGAAAGCTGCTCCTGGATGC	A	7095.5	aACAGAAAGCTGCTCCTGGATGT
rs373533	19	6919624	rs373533	EMR1	dbSNP137/GRch37	73	-1	K	G	1	1	1	W2	iPLEX	rs373533	ACGTTGGATGTCCTTTGTGGGCATGGAATC	ACGTTGGATGTGATCTCAGAGGTGGTCAAG	99	100	73.9	57	60	DH	F	6847.4	ggCAAGGGAGCCTGGTGGTCTT	G	7134.6	ggCAAGGGAGCCTGGTGGTCTTG	T	7174.5	ggCAAGGGAGCCTGGTGGTCTTT
12-72245636	12	68642647	rs2227507	IL22	dbSNP137/GRch37	73	1	Y	T	1	1	1	W2	iPLEX	12-72245636	ACGTTGGATGAACAGCAAATCCAGTTCTCC	ACGTTGGATGGAAAACATGGCATAAATGCTC	119	87.6	73.9	50	40.9		R	7053.6	aCTTATTTTCACAGCTTGGAGAG	T	7324.8	aCTTATTTTCACAGCTTGGAGAGA	C	7340.8	aCTTATTTTCACAGCTTGGAGAGG
tlr6_rs5743810	4	38830350	rs5743810	TLR6	dbSNP137/GRch37	73	-1	Y	C	1	1	1	W2	iPLEX	tlr6_rs5743810	ACGTTGGATGATTTTTATCAGAACTCACC	ACGTTGGATGAGGCATTTCCAAGTCGTTTC	98	78.3	73.7	50	39.1	d	R	7191.7	GAGGGTAAAATTCAGTAAGGTTG	T	7462.9	GAGGGTAAAATTCAGTAAGGTTGA	C	7478.9	GAGGGTAAAATTCAGTAAGGTTGG
hIL-13_46457	5	131995964	rs20541	IL13	dbSNP137/GRch37	73	-1	Y	C	1	1	1	W2	iPLEX	hIL-13_46457	ACGTTGGATGCCAGTTTGTAAAGGACCTGC	ACGTTGGATGTGATGCTTCGAAGTTTCAG	100	94.4	73.9	51	40.9	dh	F	7358.8	cgGCTTTCGAAGTTTCAGTTGAAC	C	7606	cgGCTTTCGAAGTTTCAGTTGAACC	T	7685.9	cgGCTTTCGAAGTTTCAGTTGAACT
rs10775349	16	4079823	rs10775349	ADCY9	dbSNP137/GRch37	73	-1	S	C	1	1	1	W2	iPLEX	rs10775349	ACGTTGGATGGTTGGCTCTTCAGTGAAGTG	ACGTTGGATGAAAACCAGACCACTCAGTAG	86	97.8	73.9	51	33.3		R	7377.8	ACCACTCAGTAGAAAAATGTGTAA	G	7625	ACCACTCAGTAGAAAAATGTGTAAC	C	7665.1	ACCACTCAGTAGAAAAATGTGTAAG
TLR6_rs5743809	4	38830514	rs5743809	TLR6	dbSNP137/GRch37	73	-1	Y	T	1	1	1	W2	iPLEX	TLR6_rs5743809	ACGTTGGATGGCGAATAAACTAGTTGGGTG	ACGTTGGATGAATGAGACAGAAAGTCTAC	94	86.5	73.7	49	33.3	D	F	7707.1	gATGAGACAGAAAGTCTACAAATTC	C	7954.2	gATGAGACAGAAAGTCTACAAATTCC	T	8034.1	gATGAGACAGAAAGTCTACAAATTCT
tlr1_rs4833095	4	38799710	rs4833095	TLR1	dbSNP137/GRch37	73	1	Y	C	1	1	1	W2	iPLEX	tlr1_rs4833095	ACGTTGGATGACTTCCTAAGTATTCTGGCG	ACGTTGGATGCCAAGTTGTTTCAATGTTGT	100	84.3	73.7	50	28	Ds	F	7741.1	TGTTTCAATGTTGTTTAAGGTAAGA	C	7988.2	TGTTTCAATGTTGTTTAAGGTAAGAC	T	8068.2	TGTTTCAATGTTGTTTAAGGTAAGAT
tlr1_rs5743611	4	38800214	rs5743611	TLR1	dbSNP137/GRch37	73	-1	S	G	1	1	1	W2	iPLEX	tlr1_rs5743611	ACGTTGGATGGGTTGAATTTGAAAACACTG	ACGTTGGATGCACTGTCAAAACTGAGGATT	98	80.7	73.7	47	22.2		F	8302.4	TGAGGATTTTGATAATTTCTCATAATA	C	8549.6	TGAGGATTTTGATAATTTCTCATAATAC	G	8589.6	TGAGGATTTTGATAATTTCTCATAATAG
IL-4_2243251	5	132009787	rs2243251	IL4	dbSNP132/GRch37	63	-1	Y	T	1	1	1	W3	iPLEX	IL-4_2243251	ACGTTGGATGTGTCCGTGGACAAAGTTGCC	ACGTTGGATGCTATTAATGGGTCTCACCTC	100	97.1	61.2	45	50	D	R	4765.1	CTCTGTTCTTCCTGCT	T	5036.3	CTCTGTTCTTCCTGCTA	C	5052.3	CTCTGTTCTTCCTGCTG
rs5470	16	72088421	rs5470	HP	dbSNP132/GRch37	63	1	S	C	1	1	1	W3	iPLEX	rs5470	ACGTTGGATGAACAGGGGTATTGTTTGTGG	ACGTTGGATGGTGTCTACAAACTTTGGCCC	92	98.3	61.2	47	56.3	Dg	R	4888.2	CTTTGGCCCTGGTAAG	G	5135.4	CTTTGGCCCTGGTAAGC	C	5175.4	CTTTGGCCCTGGTAAGG
IL10_232428480	1	206944861	rs1518110	IL10	dbSNP132/GRch37	63	1	M	C	1	1	1	W3	iPLEX	IL10_232428480	ACGTTGGATGATGCGGTCTTTTTGATGCCC	ACGTTGGATGTGATTTTTTTGGGCCAGAGC	95	98.5	61.2	50	56.3	d	R	4906.2	GGGCCAGAGCCAATTT	C	5193.4	GGGCCAGAGCCAATTTG	A	5233.3	GGGCCAGAGCCAATTTT
rs455649	5	131649306	rs455649	SLC22A4	dbSNP132/GRch37	63	1	K	G	1	1	1	W3	iPLEX	rs455649	ACGTTGGATGAACACACCTTGGCAACCTAC	ACGTTGGATGCGAAGAGAACGTTCTTCCTG	101	100	61.2	45	50	d	R	5096.3	tGTTCTTCCTGCCAAAC	G	5343.5	tGTTCTTCCTGCCAAACC	T	5367.5	tGTTCTTCCTGCCAAACA
rs10479007	5	131917726	rs10479007	RAD50	dbSNP132/GRch37	63	1	R	A	1	1	1	W3	iPLEX	rs10479007	ACGTTGGATGGTCATGGCCAAATTCAGTCC	ACGTTGGATGGGAGCTCTGAGGATAAAGAA	99	96.1	61.2	50	58.8	D	F	5324.5	TGAGGTAGCCAGAGAGG	A	5595.7	TGAGGTAGCCAGAGAGGA	G	5611.7	TGAGGTAGCCAGAGAGGG
rs1801274	1	161479745	rs1801274	FCGR2a	dbSNP137/GRch37	73	-1	Y	T	1	1	1	W3	iPLEX	rs1801274	ACGTTGGATGCTGTGACTGTGGTTTGCTTG	ACGTTGGATGCTTCCAGAATGGAAAATCCC	98	98.5	61.2	47	44.4	D	R	5387.5	AATCCCAGAAATTCTCCC	T	5658.7	AATCCCAGAAATTCTCCCA	C	5674.7	AATCCCAGAAATTCTCCCG
hNOS2-1026	17	26128581	rs2779249	NOS2A	dbSNP132/GRch37	63	-1	K	G	1	1	1	W3	iPLEX	hNOS2-1026	ACGTTGGATGGGGAATACTGTATTTCAGGC	ACGTTGGATGGTGCTAGGATTACAAGGGTT	98	94.7	61.2	45	50		R	5468.6	ccTACAAGGGTTAGCCAC	G	5715.8	ccTACAAGGGTTAGCCACC	T	5739.8	ccTACAAGGGTTAGCCACA
hIL1a-889	2	113542960	rs1800587	IL1A	dbSNP132/GRch37	63	-1	Y	C	1	1	1	W3	iPLEX	hIL1a-889	ACGTTGGATGGGATTTTTACATATGAGCC	ACGTTGGATGCAGGAATTATAAAAGCTGAG	97	77.1	61.2	46	38.9	d	F	5509.6	AATAGTAACCAGGCAACA	C	5756.8	AATAGTAACCAGGCAACAC	T	5836.7	AATAGTAACCAGGCAACAT
IFNg_plus3234	12	68550162	rs2069718	IFNG	dbSNP132/GRch37	63	-1	Y	T	1	1	1	W3	iPLEX	IFNg_plus3234	ACGTTGGATGCTCACTCTAACCAATAGGGC	ACGTTGGATGCAGAGCCAAGAGGAAGGTAA	93	97.3	61.2	46	38.9	Dh	F	5531.6	AAGGTAAATGGTCCACAT	C	5778.8	AAGGTAAATGGTCCACATC	T	5858.7	AAGGTAAATGGTCCACATT
IFNg_plus4640	12	68548756	rs2234687	IFNG	dbSNP132/GRch37	63	-1	Y	C	1	1	1	W3	iPLEX	IFNg_plus4640	ACGTTGGATGCAAGTTCTGTCTGACATGCC	ACGTTGGATGATACTATCCAGTTACTGCCG	99	98.6	61.2	46	38.9		F	5553.6	TGCCGGTTTGAAAATATG	C	5800.8	TGCCGGTTTGAAAATATGC	T	5880.7	TGCCGGTTTGAAAATATGT
rs28903086	5	131915022	rs28903086	RAD50	dbSNP132/GRch37	63	1	R	G	1	1	1	W3	iPLEX	rs28903086	ACGTTGGATGTTCTCGGTCAATTTCTGCAC	ACGTTGGATGCTTGATTTTCATTTTCTGTAGGC	93	66.6	61.2	48	44.4	D	F	5627.7	TGTAGGCATGGTGAAAAG	A	5898.9	TGTAGGCATGGTGAAAAGA	G	5914.9	TGTAGGCATGGTGAAAAGG
rs35182390	12	57493602	rs35182390	STAT6	dbSNP132/GRch37	63	1	R	G	1	1	1	W3	iPLEX	rs35182390	ACGTTGGATGTTCTTCTCAATGAGCCCGAC	ACGTTGGATGCAATGGTGATGCCCCCAATC	92	98.3	61.2	54	57.9	D	F	5699.7	TGCCCCCAATCTCTGAGTC	A	5970.9	TGCCCCCAATCTCTGAGTCA	G	5986.9	TGCCCCCAATCTCTGAGTCG
rs2289276	5	110407507	rs2289276	TSLP	dbSNP132/GRch37	63	1	Y	C	1	1	1	W3	iPLEX	rs2289276	ACGTTGGATGTGGCAATGAGAGGCAAAACC	ACGTTGGATGCCACGAGTGTAAGAGATCTG	95	99.9	61.2	50	55.6	d	R	5819.8	gCTGTAAGGCCTGCCTTAG	T	6091	gCTGTAAGGCCTGCCTTAGA	C	6107	gCTGTAAGGCCTGCCTTAGG
IFNg_plus5173	12	68548223	rs2069727	IFNG	dbSNP132/GRch37	63	-1	R	A	1	1	1	W3	iPLEX	IFNg_plus5173	ACGTTGGATGCCCTTCTCCACCTTCCTATT	ACGTTGGATGACTGGTGTTTGCCAGCATTG	95	97.4	61.2	46	42.1	D	R	5949.9	GGAGAGGAAGAATTCTGAAA	G	6197.1	GGAGAGGAAGAATTCTGAAAC	A	6277	GGAGAGGAAGAATTCTGAAAT
rs11575936	6	137540425	rs11575936	IFNGR1	dbSNP132/GRch37	63	1	Y	C	1	1	1	W3	iPLEX	rs11575936	ACGTTGGATGATCCGCGGTGCCCATCTCA	ACGTTGGATGCAGCATGGCTCTCCTCTTTC	93	86.9	61.2	55	61.1	D	R	6059.9	tcCCCCTTGTCATGCAGGGT	T	6331.1	tcCCCCTTGTCATGCAGGGTA	C	6347.1	tcCCCCTTGTCATGCAGGGTG
rs10127939	1	161518333	rs10127939	CD16	dbSNP132/GRch37	63	1	M	A	1	1	1	W3	iPLEX	rs10127939	ACGTTGGATGAGCGTCAATGAAGTAGCTCG	ACGTTGGATGCCCTGAGGACAATTCCACAC	99	98.6	61.2	52	50	D	R	6157	GTGGTTTCACAATGAGAGCC	C	6444.2	GTGGTTTCACAATGAGAGCCG	A	6484.1	GTGGTTTCACAATGAGAGCCT
hIL-10-592	1	206946407	rs1800872	IL10	dbSNP132/GRch37	63	1	K	G	1	1	1	W3	iPLEX	hIL-10-592	ACGTTGGATGCCTGGAACACATCCTGTGAC	ACGTTGGATGAAGCAGCCCTTCCATTTTAC	94	97	61.2	51	52.6	DH	F	6415.2	caAGAGACTGGCTTCCTACAG	G	6702.4	caAGAGACTGGCTTCCTACAGG	T	6742.3	caAGAGACTGGCTTCCTACAGT
5-131530786	5	132008644	rs2243248	IL4	dbSNP132/GRch37	63	1	K	T	1	1	1	W3	iPLEX	5-131530786	ACGTTGGATGGAACCTAAACACATCCTCAG	ACGTTGGATGTGACTAGGAGGGCTGATTTG	96	98.5	61.2	46	33.3		F	6515.3	TGATTTGTAAGTTGGTAAGAC	G	6802.5	TGATTTGTAAGTTGGTAAGACG	T	6842.4	TGATTTGTAAGTTGGTAAGACT
hIL-10-819	1	206946634	rs1800871	IL10	dbSNP132/GRch37	63	1	R	G	1	1	1	W3	iPLEX	hIL-10-819	ACGTTGGATGTGACCCCTACCGTCTCTATT	ACGTTGGATGGGTGTACCCTTGTACAGGTG	96	91.8	61.2	50	45		R	6725.4	tcCCCTTGTACAGGTGATGTAA	G	6972.6	tcCCCTTGTACAGGTGATGTAAC	A	7052.5	tcCCCTTGTACAGGTGATGTAAT
IFNg_plus2200	12	68551196	rs1861493	IFNG	dbSNP132/GRch37	63	-1	Y	T	1	1	1	W3	iPLEX	IFNg_plus2200	ACGTTGGATGTTGGAGCAAAGAAGGTCATC	ACGTTGGATGGGCTCAGTTTCCTCATCTTA	92	94.4	61.2	46	33.3	d	F	6782.4	cTCTTAAAAGGAAACTGTTAGG	C	7029.6	cTCTTAAAAGGAAACTGTTAGGC	T	7109.5	cTCTTAAAAGGAAACTGTTAGGT
RAD50-rs1047380	5	131930613	rs1047380	RAD50	dbSNP132/GRch37	63	1	R	A	1	1	0	W3	iPLEX	RAD50-rs1047380	ACGTTGGATGAGGAACTAGCTTCATCTGAG	ACGTTGGATGTCGTAACTGGACAACTGCTC	102	98.5	61.2	54	43.5	Ds	R	6931.5	GGACAACTGCTCTTCCTTTCTTT	G	7178.7	GGACAACTGCTCTTCCTTTCTTTC	A	7258.6	GGACAACTGCTCTTCCTTTCTTTT
rs3194051	5	35876274	rs3194051	IL7R	dbSNP132/GRch37	63	1	R	A	1	1	1	W3	iPLEX	rs3194051	ACGTTGGATGAGGCATGTGAGGGATGAATC	ACGTTGGATGCCAACTGCCCATCTGAGGA	90	93.2	61.2	54	52.4	D	F	7079.6	gaTGCCCATCTGAGGATGTAGTC	A	7350.8	gaTGCCCATCTGAGGATGTAGTCA	G	7366.8	gaTGCCCATCTGAGGATGTAGTCG
rs13166954	5	131396709	rs13166954	IL3	dbSNP132/GRch37	63	1	Y	C	1	1	1	W3	iPLEX	rs13166954	ACGTTGGATGATGACATGGCTGAAGTCACC	ACGTTGGATGGGGAAGACCAAGACATTCTG	104	99.9	61.2	49	40.9	s	F	7136.7	gCAAGACATTCTGATGGTAAGAG	C	7383.8	gCAAGACATTCTGATGGTAAGAGC	T	7463.8	gCAAGACATTCTGATGGTAAGAGT
IFNg-1616	12	68555011	rs2069705	IFNG	dbSNP132/GRch37	63	-1	Y	C	1	1	1	W3	iPLEX	IFNg-1616	ACGTTGGATGTTCCTGACTCCTCTACTATC	ACGTTGGATGAGGAGACTGAGTCATAGAAG	92	96.9	61.2	46	30.4	d	F	7159.7	TGAGTCATAGAAGATTTAAGAAG	C	7406.9	TGAGTCATAGAAGATTTAAGAAGC	T	7486.8	TGAGTCATAGAAGATTTAAGAAGT
hIL-8-251	4	74606024	rs4073	IL8	dbSNP132/GRch37	63	1	W	A	1	1	1	W3	iPLEX	hIL-8-251	ACGTTGGATGGGTACTATGATAAAGTTATCT	ACGTTGGATGCTGAAGCTCCACAATTTGGT	91	70.6	61.2	50	33.3	D	R	7310.8	CTCCACAATTTGGTGAATTATCAA	T	7582	CTCCACAATTTGGTGAATTATCAAA	A	7637.9	CTCCACAATTTGGTGAATTATCAAT
rs739718	5	131873073	rs739718	IL5	dbSNP132/GRch37	63	1	Y	T	1	1	1	W3	iPLEX	rs739718	ACGTTGGATGGGAAGTCCCATTCTTACCAA	ACGTTGGATGGTTAAATATGAGGTCTGAGC	100	90.7	61.2	48	34.8	d	F	7424.9	gAAATATGAGGTCTGAGCTAATAC	C	7672	gAAATATGAGGTCTGAGCTAATACC	T	7752	gAAATATGAGGTCTGAGCTAATACT
hIL-13_50012	5	131992409	rs1881457	IL13	dbSNP132/GRch37	63	-1	K	T	1	1	1	W3	iPLEX	hIL-13_50012	ACGTTGGATGTGCCTGGAGTGCCGCTACTT	ACGTTGGATGGGCCCTCTACTACAGATTAG	90	91.6	61.2	54	50	d	R	7443.9	aaAGATTAGGAAACAGGCCCGTAG	G	7691.1	aaAGATTAGGAAACAGGCCCGTAGC	T	7715.1	aaAGATTAGGAAACAGGCCCGTAGA
rs156112	5	131610232	rs156112	PDLIM4	dbSNP132/GRch37	63	1	Y	C	1	1	1	W3	iPLEX	rs156112	ACGTTGGATGCATGTCATGGGATTAAGGTG	ACGTTGGATGCAGAAACTCGACTCTCCCAC	92	96.9	61.2	61	58.3	D	F	7564.9	aACTGGCCACCACAGATCCACTAGC	C	7812.1	aACTGGCCACCACAGATCCACTAGCC	T	7892	aACTGGCCACCACAGATCCACTAGCT
rs168681	5	131402450	rs168681	IL3	dbSNP132/GRch37	63	1	R	G	1	1	1	W3	iPLEX	rs168681	ACGTTGGATGGCCCAACTTTCCCCCTTGAA	ACGTTGGATGGCAGCAGCAACTTTGCTGAC	87	62.7	61.2	59	56.5	D	R	7608.9	gtCTGACCCACCTCGTTGAGCTTGA	G	7856.1	gtCTGACCCACCTCGTTGAGCTTGAC	A	7936	gtCTGACCCACCTCGTTGAGCTTGAT
rs10040427	5	131714106	rs10040427	SLC22A5	dbSNP132/GRch37	63	1	Y	C	1	1	1	W3	iPLEX	rs10040427	ACGTTGGATGCACACCCACGAAGAACAAGG	ACGTTGGATGTGCTCATCTTGCAGTGGAAC	100	98.6	61.2	64	62.5	h	F	7732	tTGTGAGGACGACTGGAAGGCCCCA	C	7979.2	tTGTGAGGACGACTGGAAGGCCCCAC	T	8059.1	tTGTGAGGACGACTGGAAGGCCCCAT
rs2522051	5	131797578	rs2522051	LOC441108	dbSNP132/GRch37	63	1	Y	C	1	1	1	W3	iPLEX	rs2522051	ACGTTGGATGTAAAGCTGAATTATGTTCC	ACGTTGGATGACAACCCAGATGTCCATGAC	99	79.5	61.2	45	26.1	d	F	7786.1	tgGGATAAACAAAATATGGTGAATA	C	8033.3	tgGGATAAACAAAATATGGTGAATAC	T	8113.2	tgGGATAAACAAAATATGGTGAATAT
rs2706348	5	131905810	rs2706348	RAD50	dbSNP132/GRch37	63	1	R	A	1	1	1	W3	iPLEX	rs2706348	ACGTTGGATGCTTTCGTGGTAAAAACCCTC	ACGTTGGATGACACCTAACGGATTTTGGAC	100	97.1	61.2	48	28	DH	R	7919.2	gTATTCAGGAATTAATATTCCTCCAT	G	8166.4	gTATTCAGGAATTAATATTCCTCCATC	A	8246.3	gTATTCAGGAATTAATATTCCTCCATT
rs3798134	5	131965179	rs3798134	RAD50	dbSNP132/GRch37	63	1	R	G	1	1	1	W3	iPLEX	rs3798134	ACGTTGGATGACAGGAGTGCTTTCTAGCAA	ACGTTGGATGTGAGTACCAGGTAAGAGAAC	99	94.7	61.2	56	41.7		R	7961.2	ggAAACAGCCTGGCTTTGTTCCTTAA	G	8208.4	ggAAACAGCCTGGCTTTGTTCCTTAAC	A	8288.3	ggAAACAGCCTGGCTTTGTTCCTTAAT
IRF1rs2070722	5	131824486	rs2070722	IRF1	dbSNP132/GRch37	63	-1	K	G	1	1	1	W3	iPLEX	IRF1rs2070722	ACGTTGGATGTTTAATCCTCGCAGTAGTCC	ACGTTGGATGCTCTCAGTGCCTCTGTTTAC	99	98.6	61.2	49	25.9	d	R	8354.5	TGAAAATGATGACATTAGTAAGATCTA	G	8601.7	TGAAAATGATGACATTAGTAAGATCTAC	T	8625.7	TGAAAATGATGACATTAGTAAGATCTAA
rs28903088	5	131915673	rs28903088	RAD50	dbSNP132/GRch37	63	1	R	G	1	1	1	W3	iPLEX	rs28903088	ACGTTGGATGCCTTTGAAGATGTTAACTGG	ACGTTGGATGGGAACTAAAATATCTGAAGC	112	82.3	61.2	51	29.6	D	F	8379.5	AATATAAGGAAAAAGCTTGTGAGATTC	A	8650.7	AATATAAGGAAAAAGCTTGTGAGATTCA	G	8666.7	AATATAAGGAAAAAGCTTGTGAGATTCG
rs2069640	5	131411454	rs2069640	CSF2	dbSNP132/GRch37	63	1	Y	C	1	1	0	W3	iPLEX	rs2069640	ACGTTGGATGAGTCCTTCAGGTTCTCTTTG	ACGTTGGATGGCCTGGACTCAAGTGTTTTT	120	93.2	61.2	53	29.6	Dh	F	8567.6	tTTTTTTAAAGGAAACTTCCTGTGCAA	C	8814.8	tTTTTTTAAAGGAAACTTCCTGTGCAAC	T	8894.7	tTTTTTTAAAGGAAACTTCCTGTGCAAT
rs2626577	12	57500509	rs2626577	STAT6	dbSNP132/GRch37	63	1	S	C	1	1	1	W4	iPLEX	rs2626577	ACGTTGGATGCCACCTTGGCCAGCCTCAG	ACGTTGGATGAGGCTGCAGCACCGAGTAG	97	75.1	68.5	47	56.3		R	4777.1	GATCCACCTTCTCCGA	G	5024.3	GATCCACCTTCTCCGAC	C	5064.3	GATCCACCTTCTCCGAG
rs2706381	5	131810619	rs2706381	LOC441108	dbSNP132/GRch37	63	1	Y	C	1	1	1	W4	iPLEX	rs2706381	ACGTTGGATGGGTAGAAAGGACCTCTCTG	ACGTTGGATGTCCTCAGGCATAGACAGCCG	97	92.9	68.5	45	50	Dsh	F	4881.2	ACAGTCAGGATGTTCC	C	5128.4	ACAGTCAGGATGTTCCC	T	5208.3	ACAGTCAGGATGTTCCT
rs156029	5	131532634	rs156029	P4HA2	dbSNP132/GRch37	63	1	R	A	1	1	1	W4	iPLEX	rs156029	ACGTTGGATGTTGTGCTGTGTACATGCTGG	ACGTTGGATGAGTCTCTCATATTGGACCTC	92	98.3	68.5	47	56.3	D	F	4884.2	GGACCTCAAGCACAAG	A	5155.4	GGACCTCAAGCACAAGA	G	5171.4	GGACCTCAAGCACAAGG
rs3900945	5	131592870	rs3900945	PDLIM4	dbSNP132/GRch37	63	1	Y	C	1	1	1	W4	iPLEX	rs3900945	ACGTTGGATGTTTCTGTGTCCACTCCCACG	ACGTTGGATGCTTGCGCAGCACTGGGCCG	96	72.3	68.5	55	68.8	Dg	R	5238.4	aGGGGACGCAGACCCAA	T	5509.6	aGGGGACGCAGACCCAAA	C	5525.6	aGGGGACGCAGACCCAAG
rs2243206	5	132001065	rs2243206	IL13	dbSNP132/GRch37	63	1	Y	C	1	1	1	W4	iPLEX	rs2243206	ACGTTGGATGGTGGCTGAAGTAACAACACT	ACGTTGGATGGCTCAGGGTGAAACTAGTCC	93	94.5	68.5	52	58.8	D	F	5444.5	gAGTCCCAGCTTCAAGCC	C	5691.7	gAGTCCCAGCTTCAAGCCC	T	5771.6	gAGTCCCAGCTTCAAGCCT
rs25887	5	131416061	rs25887	CSF2	dbSNP132/GRch37	63	1	M	C	1	1	1	W4	iPLEX	rs25887	ACGTTGGATGTTTACAGCTGGGCAGGTTTG	ACGTTGGATGTTGCAACCACAACTTAGCCC	100	100	68.5	54	55.6	D	F	5477.6	AACTTAGCCCAGGCCAGA	C	5724.8	AACTTAGCCCAGGCCAGAC	A	5748.8	AACTTAGCCCAGGCCAGAA
CSF2_2069614	5	131407601	rs2069614	CSF2	dbSNP132/GRch37	63	1	Y	C	1	1	1	W4	iPLEX	CSF2_2069614	ACGTTGGATGTTTCCAATCCACACCAGGAC	ACGTTGGATGAGCCTCAAGGGCCAAATGTG	99	98.6	68.5	57	66.7		F	5571.6	CAGTGGGAGGGTCCCGTT	C	5818.8	CAGTGGGAGGGTCCCGTTC	T	5898.7	CAGTGGGAGGGTCCCGTTT
rs2069744	5	131994669	rs2069744	IL13	dbSNP132/GRch37	63	1	Y	C	1	1	1	W4	iPLEX	rs2069744	ACGTTGGATGAAGTGACAGAGGCTGGATTC	ACGTTGGATGTGAGGGAACACTGCAGTTAG	96	99.9	68.5	47	52.9	dH	R	5579.6	gCTGCAGTTAGGAGCATG	T	5850.8	gCTGCAGTTAGGAGCATGA	C	5866.8	gCTGCAGTTAGGAGCATGG
5-130913891	5	131391749	rs3091336	IL3	dbSNP132/GRch37	63	1	R	A	1	1	1	W4	iPLEX	5-130913891	ACGTTGGATGTGAGCTCTCTGCTCAGGTG	ACGTTGGATGAAGGCAGCTCAGGGTTCTTG	93	94.4	68.5	57	61.1	D	R	5791.8	cGCCAGCCAAGCAGTGCAA	G	6039	cGCCAGCCAAGCAGTGCAAC	A	6118.9	cGCCAGCCAAGCAGTGCAAT

rs11568525	5	131729935	rs11568525	SLC22A5	dbSNP132/GRch37	63	1	Y	C	1	1	1	W4	iPLEX	rs11568525	ACGTTGGATGGAAGCGATGTTAGAAGGCTG	ACGTTGGATGCTCCAAGTCACACAAGGATG	100	100	68.5	46	36.8	D	F	5942.9	AAAAGATGGTCAAGAAAGG	C	6190.1	AAAAGATGGTCAAGAAAGGC	T	6270	AAAAGATGGTCAAGAAAGGT
rs569108	11	59863104	rs569108	MS4A2	dbSNP132/GRch37	63	1	R	A	1	1	1	W4	iPLEX	rs569108	ACGTTGGATGCTGGACACGTGATTCTTATA	ACGTTGGATGTTACAGTGAGTTGGAAGACC	87	90	68.5	55	63.2	DgH	F	5942.9	GAGTTGGAAGACCCAGGGG	A	6214.1	GAGTTGGAAGACCCAGGGGA	G	6230.1	GAGTTGGAAGACCCAGGGGG
IL-3_40401	5	131396478	rs40401	IL3	dbSNP132/GRch37	63	1	Y	T	1	1	1	W4	iPLEX	IL-3_40401	ACGTTGGATGGACTCCAAGCTCCCATGAC	ACGTTGGATGCATCGATCATGTTAGAGCAG	92	94.3	68.5	53	52.6	D	R	6068	tACCCAGCTTGTCTTCAAGG	T	6339.2	tACCCAGCTTGTCTTCAAGGA	C	6355.2	tACCCAGCTTGTCTTCAAGGG
rs2548999	5	131831058	rs2548999	IRF1	dbSNP132/GRch37	63	1	K	G	1	1	1	W4	iPLEX	rs2548999	ACGTTGGATGCCAGGTTTCTGTGAAAATCG	ACGTTGGATGTCCAGGTCCAGAAGTAGTAG	90	98.2	68.5	48	42.1	D	F	6085	aGCTCTCTGAAATGCTCAAA	G	6372.2	aGCTCTCTGAAATGCTCAAAG	T	6412.1	aGCTCTCTGAAATGCTCAAAT
rs11568510	5	131647954	rs11568510	SLC22A4	dbSNP132/GRch37	63	1	R	A	1	1	0	W4	iPLEX	rs11568510	ACGTTGGATGTTCCTCCCCTCCCATGTGCT	ACGTTGGATGAAGGTGCCCCTCACCACCTC	120	75.9	68.5	55	68.8	DH	F	6174	aggtTCCGGGCAGCTGTCAG	A	6445.2	aggtTCCGGGCAGCTGTCAGA	G	6461.2	aggtTCCGGGCAGCTGTCAGG
5-131342063	5	131819921	rs2070729	IRF1	dbSNP132/GRch37	63	1	M	A	1	1	1	W4	iPLEX	5-131342063	ACGTTGGATGCTTCAGGCAAACCTTAAGGC	ACGTTGGATGAGGGCGGTAAAGCATCTGG	95	95.9	68.5	47	38.1	D	R	6429.2	GACACATTGTGAATTAGCTAC	C	6716.4	GACACATTGTGAATTAGCTACG	A	6756.3	GACACATTGTGAATTAGCTACT
IL5-rs2069818	5	131877524	rs2069818	IL5	dbSNP132/GRch37	63	-1	M	C	1	1	1	W4	iPLEX	IL5-rs2069818	ACGTTGGATGAGACTACCTGCAAGAGTTTC	ACGTTGGATGGCTGCAACAAACCAGTTTAG	100	97.1	68.5	46	36.4	d	R	6555.3	CTCAACTTTCTATTATCCACTC	C	6842.5	CTCAACTTTCTATTATCCACTCG	A	6882.4	CTCAACTTTCTATTATCCACTCT
rs10463891	5	131597392	rs10463891	PDLIM4	dbSNP132/GRch37	63	1	R	G	1	1	1	W4	iPLEX	rs10463891	ACGTTGGATGTCTTCAGTTGACCAGCATTG	ACGTTGGATGACCTCAGTGCCACTATTGAC	99	98.6	68.5	51	50	d	R	6670.3	ctgtTCAAGCAGTGTTCACACC	G	6917.5	ctgtTCAAGCAGTGTTCACACCC	A	6997.4	ctgtTCAAGCAGTGTTCACACCT
IL-4_2243270	5	132014109	rs2243270	IL4	dbSNP132/GRch37	63	-1	Y	C	1	1	1	W4	iPLEX	IL-4_2243270	ACGTTGGATGAAGTGTGACGATATTTACC	ACGTTGGATGACATTCACTCATCCCACCAG	88	87.4	68.5	54	55		F	6697.4	taCCACCAGCCAGAGGTAACTA	C	6944.6	taCCACCAGCCAGAGGTAACTAC	T	7024.5	taCCACCAGCCAGAGGTAACTAT
rs6897932	5	35874575	rs6897932	IL7R	dbSNP132/GRch37	63	1	Y	C	1	1	1	W4	iPLEX	rs6897932	ACGTTGGATGAAGATGACCAACAGAGCGAC	ACGTTGGATGCAGGGGAGATGGATCCTATC	93	94	68.5	46	36.4	g	F	6733.4	GAGATGGATCCTATCTTACTAA	C	6980.6	GAGATGGATCCTATCTTACTAAC	T	7060.5	GAGATGGATCCTATCTTACTAAT
rs35415145	5	131396406	rs35415145	IL3	dbSNP132/GRch37	63	1	Y	C	1	1	1	W4	iPLEX	rs35415145	ACGTTGGATGGAAGGACCAGAACAAGACAG	ACGTTGGATGAGGAGTTGGAGCAGGAGCAG	100	94.4	68.5	65	76.2	Dh	R	6860.4	cGCAGGAGCAGGACGGGCAGGC	T	7131.7	cGCAGGAGCAGGACGGGCAGGCA	C	7147.7	cGCAGGAGCAGGACGGGCAGGCG
rs2706379	5	131805735	rs2706379	LOC441108	dbSNP132/GRch37	63	1	Y	C	1	1	1	W4	iPLEX	rs2706379	ACGTTGGATGGCATATACACACATGAATC	ACGTTGGATGATCTGCTGCCCTATCCATTC	83	86.9	68.5	51	47.4	D	R	6901.5	caccTCCATTCACTGTGCTGTTC	T	7172.7	caccTCCATTCACTGTGCTGTTCA	C	7188.7	caccTCCATTCACTGTGCTGTTCG
rs7704457	5	131744790	rs7704457	LOC441108	dbSNP132/GRch37	63	1	Y	T	1	1	1	W4	iPLEX	rs7704457	ACGTTGGATGACAGTGCTAATCGACTCTGG	ACGTTGGATGTCCAGGTTTGGTCCTTGAC	96	98.4	68.5	51	47.4	Dg	R	7088.6	gacgTTGTGGGGCCAAAAATCTC	T	7359.8	gacgTTGTGGGGCCAAAAATCTCA	C	7375.8	gacgTTGTGGGGCCAAAAATCTCG
rs272867	5	131681057	rs272867	SLC22A4	dbSNP132/GRch37	63	1	R	G	1	1	1	W4	iPLEX	rs272867	ACGTTGGATGCCTGTTTCCTAAAAATGGCT	ACGTTGGATGCCAATCTAACTGGTTTGTGC	100	90.7	68.5	46	29.2	D	R	7289.8	CAGTTTTTCTGATTTTGTATCTAC	G	7536.9	CAGTTTTTCTGATTTTGTATCTACC	A	7616.8	CAGTTTTTCTGATTTTGTATCTACT
rs4621555	5	131982808	rs4621555	RAD50	dbSNP132/GRch37	63	1	Y	C	1	1	1	W4	iPLEX	rs4621555	ACGTTGGATGCAAACAAATACATACCTGTG	ACGTTGGATGCACCTTTTTCCAACAGTGAG	84	85.6	68.5	47	35	D	R	7406.8	ggatTGAGAAACTCAGTTCGTATT	T	7678	ggatTGAGAAACTCAGTTCGTATTA	C	7694	ggatTGAGAAACTCAGTTCGTATTG
CSF2-rs25882	5	131411460	rs25882	CSF2	dbSNP132/GRch37	63	1	Y	T	1	1	1	W4	iPLEX	CSF2-rs25882	ACGTTGGATGTTAAAGGAAACTTCCTGTGC	ACGTTGGATGGCAGAAAGTCCTTCAGGTTC	93	94.2	68.5	45	30	ds	R	7439.9	gatgTGAAACTTTCAAAGGTGATA	T	7711.1	gatgTGAAACTTTCAAAGGTGATAA	C	7727.1	gatgTGAAACTTTCAAAGGTGATAG
rs17851430	5	131606632	rs17851430	PDLIM4	dbSNP132/GRch37	63	1	S	C	1	1	0	W4	iPLEX	rs17851430	ACGTTGGATGAGGCTTGGTCTGCCATCTTC	ACGTTGGATGTAATAGGACGGCAGCCCAAC	97	97.1	68.5	65	66.7	dh	F	7640	cGACGGCAGCCCAACAACCAGCAGG	C	7887.2	cGACGGCAGCCCAACAACCAGCAGGC	G	7927.2	cGACGGCAGCCCAACAACCAGCAGGG
RAD50-rs1047386	5	131944869	rs1047386	RAD50	dbSNP132/GRch37	63	1	Y	T	1	1	0	W4	iPLEX	RAD50-rs1047386	ACGTTGGATGTATAGTCGTCTTTCCCATC	ACGTTGGATGGGTTAAAAATATTCATGGC	88	74.5	68.5	51	32	d	F	7753.1	TGGCTATATGAAAGACATTGAGAAT	C	8000.3	TGGCTATATGAAAGACATTGAGAATC	T	8080.2	TGGCTATATGAAAGACATTGAGAATT
rs2251746	1	159272060	rs2251746	FCER1A	dbSNP132/GRch37	63	1	Y	T	1	1	1	W4	iPLEX	rs2251746	ACGTTGGATGCTGGAGAGATCTAAGGCTTC	ACGTTGGATGAGGCACAGCTGATGGGTTAA	115	96.6	68.5	47	40	D	F	7779.1	aagcgGATGGGTTAACCAGATATGA	C	8026.3	aagcgGATGGGTTAACCAGATATGAC	T	8106.2	aagcgGATGGGTTAACCAGATATGAT
RAD50-rs1047387	5	131951770	rs1047387	RAD50	dbSNP132/GRch37	63	1	R	A	1	1	0	W4	iPLEX	RAD50-rs1047387	ACGTTGGATGGAGGAACTAAAAGAAGTTG	ACGTTGGATGACCTGCATTTGACCCATTTC	85	85.9	68.5	52	36	d	R	7813.1	cCCCATTTCCTTCAAATGTTGTTTTC	G	8060.3	cCCCATTTCCTTCAAATGTTGTTTTCC	A	8140.2	cCCCATTTCCTTCAAATGTTGTTTTCT
rs1804669	5	131978166	rs1804669	RAD50	dbSNP132/GRch37	63	1	K	G	1	1	0	W4	iPLEX	rs1804669	ACGTTGGATGTTCTCATATCAACTTAGTC	ACGTTGGATGCCTCTCAACATCCAAAATCC	98	78.3	68.5	54	34.6	D	R	8180.4	cACATCCAAAATCCTAGACACAATGTT	G	8427.5	cACATCCAAAATCCTAGACACAATGTTC	T	8451.6	cACATCCAAAATCCTAGACACAATGTTA
rs28903092	5	131931472	rs28903092	RAD50	dbSNP132/GRch37	63	1	R	G	1	1	1	W4	iPLEX	rs28903092	ACGTTGGATGCAAGTCAACAGAATCAGAGC	ACGTTGGATGTTACCTCATGGGCACAAGTC	96	98.5	68.5	62	48.1	D	R	8228.4	ATGGGCACAAGTCCCAGCATTTCATCA	G	8475.5	ATGGGCACAAGTCCCAGCATTTCATCAC	A	8555.5	ATGGGCACAAGTCCCAGCATTTCATCAT
IL-4-rs4986964	5	132009821	rs4986964	IL4	dbSNP132/GRch37	63	1	Y	T	1	1	1	W4	iPLEX	IL-4-rs4986964	ACGTTGGATGCTGTGAGGCTGTTCAAAGTT	ACGTTGGATGATGTGCCGGCAACTTTGTCC	100	94.7	68.5	64	59.3		F	8254.4	GCCGGCAACTTTGTCCACGGACACAAG	C	8501.6	GCCGGCAACTTTGTCCACGGACACAAGC	T	8581.5	GCCGGCAACTTTGTCCACGGACACAAGT
rs28903093	5	131940498	rs28903093	RAD50	dbSNP132/GRch37	63	1	Y	T	1	1	0	W4	iPLEX	rs28903093	ACGTTGGATGCCTGTATAAGCTTACGATTC	ACGTTGGATGGCCCTTACATTAATTACTGTG	99	85.1	68.5	48	26.9	D	F	8365.4	gTACTGTGATAATATGTTTTTGTGTAG	C	8612.6	gTACTGTGATAATATGTTTTTGTGTAGC	T	8692.5	gTACTGTGATAATATGTTTTTGTGTAGT
rs730691	5	158756227	rs730691	IL12B	dbSNP132/GRch37	63	1	Y	T	1	1	1	W4	iPLEX	rs730691	ACGTTGGATGTGTTTGTTACCTTTAATTAG	ACGTTGGATGATGTGCCACCCATGCCATC	100	66.8	71.4	46	17.9		R	8523.6	TGTGACTATATCTTTTTATTCTTTAATA	T	8794.8	TGTGACTATATCTTTTTATTCTTTAATAA	C	8810.8	TGTGACTATATCTTTTTATTCTTTAATAG
rs6874639	5	131778716	rs6874639	LOC441108	dbSNP132/GRch37	63	1	R	A	1	1	1	W5	iPLEX	rs6874639	ACGTTGGATGGACACAGTTTACACTGATAG	ACGTTGGATGGGCTACAAATAATCCTGATG	100	89	65.4	46	43.8		R	4847.2	TAGTGCCTTGCTTCAA	G	5094.4	TAGTGCCTTGCTTCAAC	A	5174.3	TAGTGCCTTGCTTCAAT
5-131344214	5	131822072	rs2070724	IRF1	dbSNP132/GRch37	63	1	R	G	1	1	1	W5	iPLEX	5-131344214	ACGTTGGATGAGAGTGCTGCTGACAGCACA	ACGTTGGATGGAAAGCACACAACCTTGACC	93	95.9	65.4	50	56.3	d	R	4870.2	CTGTGGCTTCTGCTGT	G	5117.3	CTGTGGCTTCTGCTGTC	A	5197.3	CTGTGGCTTCTGCTGTT
hIL-13_45921	5	131996500	rs848	IL13	dbSNP132/GRch37	63	-1	K	T	1	1	1	W5	iPLEX	hIL-13_45921	ACGTTGGATGCAGTACTTATTACCAGGGAC	ACGTTGGATGTTGGAGCCAAGGGTTCAGAG	97	97.1	65.4	48	52.9	D	R	5228.4	GCACTAAAGCAGTGGAC	G	5475.6	GCACTAAAGCAGTGGACC	T	5499.6	GCACTAAAGCAGTGGACA
rs11544587	5	131706005	rs11544587	SLC22A5	dbSNP132/GRch37	63	1	Y	T	1	1	0	W5	iPLEX	rs11544587	ACGTTGGATGTCACAATGGTGGACAGGTAG	ACGTTGGATGGGGCAGCTGGAGCAGGAGA	93	80.6	65.4	52	64.7	dh	F	5300.5	GGAGCAGGAGAGCTGTC	C	5547.6	GGAGCAGGAGAGCTGTCC	T	5627.5	GGAGCAGGAGAGCTGTCT
rs4646201	5	131671634	rs4646201	SLC22A4	dbSNP132/GRch37	63	1	R	G	1	1	1	W5	iPLEX	rs4646201	ACGTTGGATGCACTCTGGAGGCCGTGGAT	ACGTTGGATGTCTTCACTGCTGAGCTCTAC	94	88.2	65.4	52	62.5	D	F	5315.5	tAGGAACATGGCGGTGG	A	5586.7	tAGGAACATGGCGGTGGA	G	5602.7	tAGGAACATGGCGGTGGG
rs159903	5	131540053	rs159903	P4HA2	dbSNP132/GRch37	63	1	Y	C	1	1	1	W5	iPLEX	rs159903	ACGTTGGATGGAGAAGGGATGAGCAGTGAC	ACGTTGGATGAGGCCTCCACATTCCCAATC	91	96.8	65.4	51	50	d	F	5403.5	CCCAATCCGCAATCAGTT	C	5650.7	CCCAATCCGCAATCAGTTC	T	5730.6	CCCAATCCGCAATCAGTTT
rs3805685	5	131528153	rs3805685	P4HA2	dbSNP132/GRch37	63	1	K	T	1	1	1	W5	iPLEX	rs3805685	ACGTTGGATGGGGTCTTGGATTTCAGACCT	ACGTTGGATGCCTACTTTCCAGAAAAGGAC	94	95.9	65.4	46	44.4	D	F	5565.7	ACTTAGAGGGAACACAAG	G	5852.9	ACTTAGAGGGAACACAAGG	T	5892.8	ACTTAGAGGGAACACAAGT
rs2243283	5	132016593	rs2243283	IL4	dbSNP132/GRch37	63	1	S	C	1	1	1	W5	iPLEX	rs2243283	ACGTTGGATGAAACAGTACTGACCATCGCC	ACGTTGGATGGGTAAGATACAGGGGAGGAA	94	93	65.4	49	50	Dg	F	5670.7	AGGGGAGGAAAAGATGAC	C	5917.9	AGGGGAGGAAAAGATGACC	G	5957.9	AGGGGAGGAAAAGATGACG
rs919766	5	158747564	rs919766	IL12B	dbSNP132/GRch37	63	1	M	A	1	1	1	W5	iPLEX	rs919766	ACGTTGGATGGGGTCAGAAGAGCTGAAGT	ACGTTGGATGGCTACAATCACTAGGAACTC	97	94.5	65.4	50	52.6	D	R	5692.7	CTAGGAACTCTCTCCCCAA	C	5979.9	CTAGGAACTCTCTCCCCAAG	A	6019.8	CTAGGAACTCTCTCCCCAAT
rs11955347	5	131567924	rs11955347	P4HA2	dbSNP132/GRch37	63	1	R	G	1	1	1	W5	iPLEX	rs11955347	ACGTTGGATGTGCCTTGACCACTCTATGAC	ACGTTGGATGATGTTTAAGGCCCCAGCTTG	97	100	65.4	48	42.1	d	R	5789.8	GCCTACAGGAAAAAACCTT	G	6037	GCCTACAGGAAAAAACCTTC	A	6116.9	GCCTACAGGAAAAAACCTTT
5-131185205	5	131663062	rs272893	SLC22A4	dbSNP132/GRch37	63	-1	R	A	1	1	1	W5	iPLEX	5-131185205	ACGTTGGATGCCTCCACAGAATCAAATATC	ACGTTGGATGGAGGCTGAAGATATCATCCA	99	90.6	65.4	48	36.8	d	R	5822.8	GCTGCAAAAATGAACAACA	G	6070	GCTGCAAAAATGAACAACAC	A	6149.9	GCTGCAAAAATGAACAACAT
rs3024952	12	57500112	rs3024952	STAT6	dbSNP132/GRch37	63	1	M	A	1	1	1	W5	iPLEX	rs3024952	ACGTTGGATGAGGCCCCTCTCTGCCCATAG	ACGTTGGATGTTTGGCTGCCTCTAGCTCTC	91	82.4	65.4	49	62.5	Ds	F	6053.9	ccacGGTCTCCTGCAGTAGC	C	6301.1	ccacGGTCTCCTGCAGTAGCC	A	6325.1	ccacGGTCTCCTGCAGTAGCA
RAD50-rs4526098	5	131892979	rs4526098	RAD50	dbSNP132/GRch37	63	1	R	G	1	1	1	W5	iPLEX	RAD50-rs4526098	ACGTTGGATGTTCGATCCGGGACATGTTTG	ACGTTGGATGCTTCGGCCTCAGTTAAGCCT	99	96.1	65.4	51	50	D	F	6099	CCTCAGTTAAGCCTTTGTGG	A	6370.2	CCTCAGTTAAGCCTTTGTGGA	G	6386.2	CCTCAGTTAAGCCTTTGTGGG
RAD50-rs1047382	5	131931385	rs1047382	RAD50	dbSNP132/GRch37	63	1	Y	T	1	1	0	W5	iPLEX	RAD50-rs1047382	ACGTTGGATGGTTTATCTGGAGCAAGTCGC	ACGTTGGATGTCAGACAGAGGCTGAGTTAC	91	99.7	65.4	48	47.4	D	F	6230.1	tGAGGCTGAGTTACAAGAAG	C	6477.3	tGAGGCTGAGTTACAAGAAGC	T	6557.2	tGAGGCTGAGTTACAAGAAGT
IL-3_31481	5	131397202	rs31481	IL3	dbSNP132/GRch37	63	1	R	G	1	1	1	W5	iPLEX	IL-3_31481	ACGTTGGATGAACACAGATGGCCCAGGAAC	ACGTTGGATGCTGTCTCACCTTTGCATGAC	98	97	65.4	51	45	D	F	6248.1	GGGTCAAACAAGAAGTGGAA	A	6519.3	GGGTCAAACAAGAAGTGGAAA	G	6535.3	GGGTCAAACAAGAAGTGGAAG
rs398064	5	131647902	rs398064	SLC22A4	dbSNP132/GRch37	63	1	S	C	1	1	0	W5	iPLEX	rs398064	ACGTTGGATGAACTGGAAGGTGCCCCTCA	ACGTTGGATGCTGACAGCTGCCCGGACA	99	64.3	65.4	49	52.9	dh	R	6353.2	ccatCACGCCTACGAAGAACA	G	6600.3	ccatCACGCCTACGAAGAACAC	C	6640.4	ccatCACGCCTACGAAGAACAG
rs17772565	5	131952405	rs17772565	RAD50	dbSNP132/GRch37	63	1	Y	C	1	1	1	W5	iPLEX	rs17772565	ACGTTGGATGTCCTGCCTTGACTGCCTTAG	ACGTTGGATGTACCAGTCTAGTGCCTTAGC	99	98.6	65.4	51	42.9	d	R	6414.2	TAGTGCCTTAGCATCAACAAG	T	6685.4	TAGTGCCTTAGCATCAACAAGA	C	6701.4	TAGTGCCTTAGCATCAACAAGG
rs35482671	5	131396676	rs35482671	IL3	dbSNP132/GRch37	63	1	R	A	1	1	0	W5	iPLEX	rs35482671	ACGTTGGATGACCATCAGAATGTCTTGGTC	ACGTTGGATGCTCATGGGCCTTTCTCTCC	97	94.5	65.4	47	47.1	d	F	6617.3	acaccGGACTTCAACAACCTCA	A	6888.5	acaccGGACTTCAACAACCTCAA	G	6904.5	acaccGGACTTCAACAACCTCAG
rs3024978	12	57490378	rs3024978	STAT6	dbSNP132/GRch37	63	1	R	G	1	1	1	W5	iPLEX	rs3024978	ACGTTGGATGCAATCTGGGATCTCAATGTC	ACGTTGGATGTCTTTGGGTTCTCCCTCCAG	100	97.1	65.4	54	58.8	g	F	6726.4	tacacACTGGGGTTGGCCCTTA	A	6997.6	tacacACTGGGGTTGGCCCTTAA	G	7013.6	tacacACTGGGGTTGGCCCTTAG
rs4551059	5	131719999	rs4551059	SLC22A5	dbSNP132/GRch37	63	1	R	G	1	1	0	W5	iPLEX	rs4551059	ACGTTGGATGCTATGTGGCAGCATTTGTCC	ACGTTGGATGGGATGATGGTGAAATACAGG	96	98.5	65.4	49	50	DH	R	6728.4	ggaaTCAACTCCAACCTGATGG	G	6975.6	ggaaTCAACTCCAACCTGATGGC	A	7055.5	ggaaTCAACTCCAACCTGATGGT
5-131399841	5	131877699	rs2069823	IL5	dbSNP132/GRch37	63	1	Y	T	1	1	0	W5	iPLEX	5-131399841	ACGTTGGATGAGAAATAGGCACAGCCAGAC	ACGTTGGATGATACATTGACGGCCAAAAAG	92	94.1	65.4	48	38.9	d	R	6807.5	ggttACGGCCAAAAAGTAAGTT	T	7078.7	ggttACGGCCAAAAAGTAAGTTA	C	7094.7	ggttACGGCCAAAAAGTAAGTTG
rs10072253	5	131409768	rs10072253	CSF2	dbSNP132/GRch37	63	1	Y	C	1	1	1	W5	iPLEX	rs10072253	ACGTTGGATGGCCTGTCAGCTTGATAACAT	ACGTTGGATGGGAGGTCAAACATTTCTGAG	100	94.7	65.4	45	33.3		R	6921.5	tcCTTCTACTGTTTCATTCTGTA	T	7192.7	tcCTTCTACTGTTTCATTCTGTAA	C	7208.7	tcCTTCTACTGTTTCATTCTGTAG
rs2304081	5	131667548	rs2304081	SLC22A4	dbSNP132/GRch37	63	1	R	G	1	1	1	W5	iPLEX	rs2304081	ACGTTGGATGTATCTGCATTTCCAGGTCAC	ACGTTGGATGTCAGGACTCGGAATATTGCC	100	98.6	65.4	46	38.9	D	F	6956.5	ccctcTCTTTGCTGCTATGGTAA	A	7227.7	ccctcTCTTTGCTGCTATGGTAAA	G	7243.7	ccctcTCTTTGCTGCTATGGTAAG
rs3212227	5	158742950	rs3212227	IL12B	dbSNP132/GRch37	63	1	K	T	1	1	1	W5	iPLEX	rs3212227	ACGTTGGATGCTGATTGTTTCAATGAGC	ACGTTGGATGGGATCACAATGATATCTTTGC	85	74.9	65.4	46	27.3	d	R	7031.6	tTCTTTGCTGTATTTGTATAGTT	G	7278.8	tTCTTTGCTGTATTTGTATAGTTC	T	7302.8	tTCTTTGCTGTATTTGTATAGTTA
rs28903090	5	131923673	rs28903090	RAD50	dbSNP132/GRch37	63	1	K	G	1	1	0	W5	iPLEX	rs28903090	ACGTTGGATGGGGACTGATGAGCAACTAAA	ACGTTGGATGCAGTCTACCAATTTCCTTTC	100	90.7	65.4	47	35		R	7154.7	aaacTTTCCTTTCTTTCTCCCTTA	G	7401.9	aaacTTTCCTTTCTTTCTCCCTTAC	T	7425.9	aaacTTTCCTTTCTTTCTCCCTTAA
rs3181216	5	158752978	rs3181216	IL12B	dbSNP132/GRch37	63	1	W	A	1	1	1	W5	iPLEX	rs3181216	ACGTTGGATGAGGATGGGCAGAACTCCTTA	ACGTTGGATGTTATTATCCCAATGATCAG	100	77.5	65.4	46	25		R	7327.8	TATCCCAATGATCAGTAAAAATTA	T	7599	TATCCCAATGATCAGTAAAAATTAA	A	7654.9	TATCCCAATGATCAGTAAAAATTAT
rs35861031	5	131976461	rs35861031	RAD50	dbSNP132/GRch37	63	1	R	G	1	1	0	W5	iPLEX	rs35861031	ACGTTGGATGCATCATTGCCTTGGATGAGC	ACGTTGGATGTTACTCAACCAGAGCATGTG	99	98.4	65.4	52	33.3	D	R	7372.8	TGTGCAAGAGATTCAATGTTTTCT	G	7620	TGTGCAAGAGATTCAATGTTTTCTC	A	7699.9	TGTGCAAGAGATTCAATGTTTTCTT
rs35825847	19	7754284	rs35825847	FCER2	dbSNP132/GRch37	63	1	Y	C	1	1	1	W5	iPLEX	rs35825847	ACGTTGGATGAAGCCTTTTGCTCCAGCAAC	ACGTTGGATGGCATCATCACGCAGTCCTC	95	95.9	65.4	62	66.7	dh	F	7562.9	cataATCACGCAGTCCTCGCCCTGG	C	7810.1	cataATCACGCAGTCCTCGCCCTGGC	T	7890	cataATCACGCAGTCCTCGCCCTGGT
rs11568499	5	131649300	rs11568499	SLC22A4	dbSNP132/GRch37	63	1	Y	T	1	1	0	W5	iPLEX	rs11568499	ACGTTGGATGAAGAGAACGTTCTTCCTGCC	ACGTTGGATGAACACACCTTGGCAACCTAC	99	100	65.4	46	30	D	F	7579.9	cctccTGTTTTGTGTTATACTGCAT	C	7827.1	cctccTGTTTTGTGTTATACTGCATC	T	7907	cctccTGTTTTGTGTTATACTGCATT
rs12314983	12	57492308	rs12314983	STAT6	dbSNP132/GRch37	63	1	R	G	1	1	1	W5	iPLEX	rs12314983	ACGTTGGATGACTCCATCCCCCCGTATCAA	ACGTTGGATGTTCACTTACTCCTGGAAGGC	96	96	65.4	55	55	D	F	7675	gttaaTCCTGGAAGGCTGACAACAC	A	7946.2	gttaaTCCTGGAAGGCTGACAACACA	G	7962.2	gttaaTCCTGGAAGGCTGACAACACG
rs7809758	7	50573333	rs7809758	DDC	dbSNP132/GRch37	63	1	R	G	1	1	1	W6	iPLEX	rs7809758	ACGTTGGATGTTCATGCCCAGGTAGCACAG	ACGTTGGATGTGAGAAGTACCTTGTCCAGC	120	97	71.7	46	56.3	D	R	4906.2	GGTGAGACGAACCTTC	G	5153.4	GGTGAGACGAACCTTCC	A	5233.3	GGTGAGACGAACCTTCT
rs9942705	7	42445655	rs9942705	GLI3	dbSNP132/GRch37	63	1	R	G	1	1	1	W6	iPLEX	rs9942705	ACGTTGGATGGTGAAGTAAGACTGGTGTGC	ACGTTGGATGCAAACTCTCATGTTCTGTCC	88	98	71.7	47	56.3	d	R	5041.3	tTCCATCCATCCTCCAG	G	5288.5	tTCCATCCATCCTCCAGC	A	5368.4	tTCCATCCATCCTCCAGT
rs11575522	7	50535395	rs11575522	DDC	dbSNP132/GRch37	63	1	Y	C	1	1	1	W6	iPLEX	rs11575522	ACGTTGGATGATCGAGTTGCTCCTCTGTG	ACGTTGGATGATCTTCTGTGTTGTGGGCTG	98	98.4	71.7	56	66.7	Ds	R	5531.6	GGCTGTCCTGAGGATGCC	T	5802.8	GGCTGTCCTGAGGATGCCA	C	5818.8	GGCTGTCCTGAGGATGCCG
rs8176719	9	136132909	rs8176719	ABO	dbSNP137/GRch37	73	-1	I	I	1	1	1	W6	iPLEX	rs8176719	ACGTTGGATGTGTCGATGTTGAATGTGCCC	ACGTTGGATGCATGTGCAGTAGGAAGGATG	99	100	71.7	52	55.6	d	F	5570.6	GAAGGATGTCCTCGTGGT	D	5841.8	GAAGGATGTCCTCGTGGTA	I	5857.8	GAAGGATGTCCTCGTGGTG
rs6964823	7	50460096	rs6964823	IKZF1	dbSNP132/GRch37	63	1	R	G	1	1	1	W6	iPLEX	rs6964823	ACGTTGGATGTTTTGCCTAATGCTCTGCGG	ACGTTGGATGTGCTCCTAGAGAGACGAATG	91	99.7	71.7	47	35	D	R	6045.9	ATGGTCTTGTTCTTCTTCTT	G	6293.1	ATGGTCTTGTTCTTCTTCTTC	A	6373	ATGGTCTTGTTCTTCTTCTTT
rs11982772	7	50533062	rs11982772	DDC	dbSNP132/GRch37	63	1	M	A	1	1	1	W6	iPLEX	rs11982772	ACGTTGGATGTGGTGATGGCTGCACAATTC	ACGTTGGATGGTCTCAAAAAAAAAGGCTACG	86	89.4	71.7	48	33.3	D	R	6502.3	AAAAAAGGCTACGTTTGAGTA	C	6789.5	AAAAAAGGCTACGTTTGAGTAG	A	6829.4	AAAAAAGGCTACGTTTGAGTAT
rs10271341	7	50591583	rs10271341	DDC	dbSNP132/GRch37	63	1	K	T	1	1	1	W6	iPLEX	rs10271341	ACGTTGGATGAAAACCTGCACACAAATGCT	ACGTTGGATGGTTTGCTTCCAAGTTTTGAC	81	85.5	71.7	47	30.4	D	R	7052.6	CCAAGTTTTGACAGTTATTAATG	G	7299.8	CCAAGTTTTGACAGTTATTAATGC	T	7323.8	CCAAGTTTTGACAGTTATTAATGA
rs1817074	7	50574012	rs1817074	DDC	dbSNP132/GRch37	63	1	Y	C	1	1	1	W6	iPLEX	rs1817074	ACGTTGGATGGCATTTCAAAGGTGAATTTGC	ACGTTGGATGAATGCTATCCCTCCCCACTC	118	87.7	71.7	49	30.4	D	R	7079.6	GTGGAACTCAAACAAATTTGTAA	T	7350.9	GTGGAACTCAAACAAATTTGTAAA	C	7366.9	GTGGAACTCAAACAAATTTGTAAG
rs4947535	7	50531681	rs4947535	DDC	dbSNP132/GRch37	63	1	W	A	1	1	1	W6	iPLEX	rs4947535	ACGTTGGATGCCAGGCAGGACAGCAAGGG	ACGTTGGATGCCCCACCCCTCCCACTACA	97	66.9	71.7	60	62.5	d	F	7147.7	CCCACCCCTCCCACTACACTAGAG	A	7418.9	CCCACCCCTCCCACTACACTAGAGA	T	7474.8	CCCACCCCTCCCACTACACTAGAGT
rs6592961	7	50572890	rs6592961	DDC	dbSNP132/GRch37	63	1	R	A	1	1	1	W6	iPLEX	rs6592961	ACGTTGGATGCAAAGTGCAAATGCTCAGCC	ACGTTGGATGTCTCCCTCCAGACCCATGAT	100	96.1	71.7	61	56	d	F	7515.9	CCTCCAGACCCATGATGCTCCCAAA	A	7787.1	CCTCCAGACCCATGATGCTCCCAAAA	G	7803.1	CCTCCAGACCCATGATGCTCCCAAAG
rs3779074	7	50612202	rs3779074	DDC	dbSNP132/GRch37	63	1	R	G	1	1	1	W6	iPLEX	rs3779074	ACGTTGGATGTGAAAGAGACAAGCTTGCCC	ACGTTGGATGTCCTGAGAGGTCAGCATCAC	96	98.5	71.7	56	44		R	7591.9	AGGTCAGCATCACAGTCTTCATTTC	G	7839.1	AGGTCAGCATCACAGTCTTCATTTCC	A	7919	AGGTCAGCATCACAGTCTTCATTTCT
rs11552046	7	50470059	rs11552046	IKZF1	dbSNP132/GRch37	63	1	R	G	1	0	0	W6	iPLEX	rs11552046	ACGTTGGATGTGACATTTTGGGCTGACTAC	ACGTTGGATGTACTGGCGTCTATTGGGTAG	120	97	71.7	59	52	d	R	7620	ATCCTACAATGCACAGGAGAGCTCC	G	7867.2	ATCCTACAATGCACAGGAGAGCTCCC	A	7947.1	ATCCTACAATGCACAGGAGAGCTCCT
rs11575320	7	50605298	rs11575320	DDC	dbSNP132/GRch37	63	1	Y	C	1	1	1	W6	iPLEX	rs11575320	ACGTTGGATGCTGTAGTTGTTACTTCCACG	ACGTTGGATGCAGGCCCCTAGCTATGGTA	89	91.7	71.7	62	57.7	DS	R	7996.2	GGCCCCTAGCTATGGTAGGTAGCACA	T	8267.4	GGCCCCTAGCTATGGTAGGTAGCACAA	C	8283.4	GGCCCCTAGCTATGGTAGGTAGCACAG
rs2044859	7	50612562	rs2044859	DDC	dbSNP132/GRch37	63	1	R	A	1	1	1	W6	iPLEX	rs2044859	ACGTTGGATGGTGTCTCTTAAAAACACATGA	ACGTTGGATGATTTCCTGAAGCGTGATCCC	95	81.5	71.7	54	33.3	d	F	8186.4	GATCCCAACATTTTTGTCAACAAATCA	A	8457.6	GATCCCAACATTTTTGTCAACAAATCAA	G	8473.6	GATCCCAACATTTTTGTCAACAAATCAG
rs2329371	7	50615440	rs2329371	DDC	dbSNP132/GRch37	63	1	R	G	1	1	1	W6	iPLEX	rs2329371	ACGTTGGATGAGTCACACCCTGATTCTATG	ACGTTGGATGCAGCAATTTACGCTGGGTTG	115	97.7	71.7	47	25	s	R	8569.6	CTAATACATGCTGTTATAATAGCTAATA	G	8816.8	CTAATACATGCTGTTATAATAGCTAATAC	A	8896.7	CTAATACATGCTGTTATAATAGCTAATAT
hHBC	11	5248233	rs33930165	HBB	dbSNP137/GRch37	73	1	R	G	1	1	1	W7	iPLEX	hHBC	ACGTTGGATGTTCATCCACGTTCACCTTGC	ACGTTGGATGTCAAACAGACACCATGGTGC	102	100	69	48	60	D	F	4503.9	GTGCACCTGACTCCT	A	4775.1	GTGCACCTGACTCCTA	G	4791.1	GTGCACCTGACTCCTG
rs4132601	7	50470604	rs4132601	IKZF1	dbSNP132/GRch37	63	1	K	T	1	1	1	W7	iPLEX	rs4132601	ACGTTGGATGACTCTAAGAAGGCTCTCACC	ACGTTGGATGGCTTTAGACAGCACTCCTTC	110	99.6	69	46	53.3	DS	F	4955.2	gGAGAAAGATGCGCCT	G	5242.4	gGAGAAAGATGCGCCTG	T	5282.3	gGAGAAAGATGCGCCTT
rs3779084	7	50568735	rs3779084	DDC	dbSNP132/GRch37	63	1	R	A	1	1	1	W7	iPLEX	rs3779084	ACGTTGGATGAACAGCACACACTGCATAGC	ACGTTGGATGTGCAACTTCTCAAGCTCTGG	100	100	69	55	66.7	d	R	5522.6	GCCGAGGGTCTCTGTGTC	G	5769.8	GCCGAGGGTCTCTGTGTCC	A	5849.7	GCCGAGGGTCTCTGTGTCT
rs880028	7	50570136	rs880028	DDC	dbSNP132/GRch37	63	1	R	G	1	1	1	W7	iPLEX	rs880028	ACGTTGGATGAGACCTCGGCCCTTTAAATC	ACGTTGGATGGACTCAGATGTCAGGCTAAG	111	99.5	69	51	52.6	D	R	5794.8	GCTGGCTGATGTAATTCCC	G	6042	GCTGGCTGATGTAATTCCCC	A	6121.9	GCTGGCTGATGTAATTCCCT
rs1451375	7	50622712	rs1451375	DDC	dbSNP132/GRch37	63	1	M	C	1	1	1	W7	iPLEX	rs1451375	ACGTTGGATGCCTTAGGTAACCGAGGAAGC	ACGTTGGATGTCTCCCATCTCTCCATCCGA	100	94.7	69	45	42.1	D	R	5811.8	CATCATATTGGCCATAGAG	C	6099	CATCATATTGGCCATAGAGG	A	6138.9	CATCATATTGGCCATAGAGT
rs6956737	7	50620781	rs6956737	DDC	dbSNP132/GRch37	63	1	Y	C	1	1	1	W7	iPLEX	rs6956737	ACGTTGGATGCTACCTCACATGCACAGTGG	ACGTTGGATGACAGCCACTGAAGAGAATGC	98	98.5	69	54	68.8	d	F	5976.9	cccaCAGGCCCCTAGCACCA	C	6224.1	cccaCAGGCCCCTAGCACCAC	T	6304	cccaCAGGCCCCTAGCACCAT
rs6944602	7	50473751	rs6944602	IKZF1	dbSNP132/GRch37	63	1	R	G	1	1	1	W7	iPLEX	rs6944602	ACGTTGGATGGGCCTCCTAAGTTTAATTGC	ACGTTGGATGACACAGCTTCACGGTGTGAC	118	95.9	69	54	61.1	D	F	6071	aaCCAGCGGGCATTACACTC	A	6342.2	aaCCAGCGGGCATTACACTCA	G	6358.2	aaCCAGCGGGCATTACACTCG
rs17172184	7	43286129	rs17172184	HECW	dbSNP132/GRch37	63	1	R	G	1	1	1	W7	iPLEX	rs17172184	ACGTTGGATGTGCACGTGAAGCCTGTGTAG	ACGTTGGATGTCTCAACCAGGAGTGCATAC	92	98.3	69	46	47.1	d	R	6175	gacGGAGTGCATACCAGAAT	G	6422.2	gacGGAGTGCATACCAGAATC	A	6502.1	gacGGAGTGCATACCAGAATT
rs7779749	7	50418506	rs7779749	IKZF1	dbSNP132/GRch37	63	1	M	C	1	1	1	W7	iPLEX	rs7779749	ACGTTGGATGCAGGGATAAAGCGCTACCAG	ACGTTGGATGTTGTACAACTGCCGGGTGTC	94	97	69	46	30		F	6468.2	tTGTCAATCTGTGAAAGAAAT	C	6715.4	tTGTCAATCTGTGAAAGAAATC	A	6739.5	tTGTCAATCTGTGAAAGAAATA
rs10249420	7	50623451	rs10249420	DDC	dbSNP132/GRch37	63	1	S	C	1	1	1	W7	iPLEX	rs10249420	ACGTTGGATGAAGAACCTTGTCAGGACTAC	ACGTTGGATGGTTCAGTTGTGCAGCCTCTC	97	97.1	69	50	40.9		R	6651.3	CTCGGTCTTCTCATCTGTTAAA	G	6898.5	CTCGGTCTTCTCATCTGTTAAAC	C	6938.5	CTCGGTCTTCTCATCTGTTAAAG
rs7803788	7	50625898	rs7803788	DDC	dbSNP132/GRch37	63	1	Y	C	1	1	1	W7	iPLEX	rs7803788	ACGTTGGATGAGAGATCATAACCATCTGCC	ACGTTGGATGAAGAAGCAACCAACTAGAAC	91	92.7	69	49	34.8	D	R	6993.6	AGCAACCAACTAGAACATAATTC	T	7264.8	AGCAACCAACTAGAACATAATTCA	C	7280.8	AGCAACCAACTAGAACATAATTCG
rs17172181	7	43283115	rs17172181	HECW	dbSNP132/GRch37	63	1	M	A	1	1	1	W7	iPLEX	rs17172181	ACGTTGGATGAAGAGAAGTTCTGCTCTCCC	ACGTTGGATGTCAGGAGTGGATCATATGTG	92	98.3	69	47	35		F	7068.6	aacTCATATGTGAGGATGCTTTT	C	7315.8	aacTCATATGTGAGGATGCTTTTC	A	7339.8	aacTCATATGTGAGGATGCTTTTA
rs11575483	7	50544663	rs11575483	DDC	dbSNP132/GRch37	63	1	M	C	1	1	1	W7	iPLEX	rs11575483	ACGTTGGATGTGAGGTTTCATCTAGTGCCC	ACGTTGGATGGAATGAATGAATGATGAACGA	91	81.3	69	45	31.6	D	R	7142.7	gggtAATGATGAACGATTTACGT	C	7429.9	gggtAATGATGAACGATTTACGTG	A	7469.8	gggtAATGATGAACGATTTACGTT
rs6593010	7	50629888	rs6593010	DDC	dbSNP132/GRch37	63	1	R	G	1	1	1	W8	iPLEX	rs6593010	ACGTTGGATGACAGAGGCAGCGGCCTTCCA	ACGTTGGATGTTGTACCACCTGCCAGGTC	100	82.1	77	60	63.2	D	R	5733.7	AGCCCCTGCACTCATGGCA	G	5980.9	AGCCCCTGCACTCATGGCAC	A	6060.8	AGCCCCTGCACTCATGGCAT
rs11575527	7	50534327	rs11575527	DDC	dbSNP132/GRch37	63	1	S	G	1	1	1	W8	iPLEX	rs11575527	ACGTTGGATGGTGTGCAGTGCATCTTAGAG	ACGTTGGATGGGGCATAAGCCATCTCTAC	88	97.9	77	48	42.1		R	6365.2	tcAGCCATCTCTACTGAGAAT	G	6612.3	tcAGCCATCTCTACTGAGAATC	C	6652.4	tcAGCCATCTCTACTGAGAATG
rs11575387	7	50567435	rs11575387	DDC	dbSNP132/GRch37	63	1	K	T	1	1	1	W8	iPLEX	rs11575387	ACGTTGGATGATGGTTGGAAAACAGAACCC	ACGTTGGATGCACCACCAGGTGAAGTTCTT	104	96	77	46	33.3	s	F	6595.3	cAGTTCTTTACTTTACACCATC	G	6882.5	cAGTTCTTTACTTTACACCATCG	T	6922.4	cAGTTCTTTACTTTACACCATCT
rs7800827	7	50673171	rs7800827	DDC	dbSNP132/GRch37	63	1	Y	C	1	1	1	W8	iPLEX	rs7800827	ACGTTGGATGTAAGGCAGAGTGGAGTCTTG	ACGTTGGATGAGGTAGAAAGCAGGCATTCC	100	100	77	52	40.9		R	6691.4	TTTCCTGACCGTCTTGATGATA	T	6962.6	TTTCCTGACCGTCTTGATGATAA	C	6978.6	TTTCCTGACCGTCTTGATGATAG
rs10230385	7	50452552	rs10230385	IKZF1	dbSNP132/GRch37	63	1	R	A	1	1	1	W8	iPLEX	rs10230385	ACGTTGGATGGGAGTGATGATGACCTATGG	ACGTTGGATGTATTGCCTTGGGTTAGTGGG	118	98.7	77	49	47.4	D	F	6730.4	aacGTGGAAAACACAGACACTC	A	7001.6	aacGTGGAAAACACAGACACTCA	G	7017.6	aacGTGGAAAACACAGACACTCG
rs11983581	7	50532888	rs11983581	DDC	dbSNP132/GRch37	63	1	W	A	1	1	1	W8	iPLEX	rs11983581	ACGTTGGATGTCTGTCTCTGTAGGTTTGTC	ACGTTGGATGCAGTCATAAAAGGCAATATGT	94	80.3	68.5	45	27.3	D	F	6821.5	ATAAAAGGCAATATGTTGTATG	A	7092.7	ATAAAAGGCAATATGTTGTATGA	T	7148.6	ATAAAAGGCAATATGTTGTATGT
rs11980407	7	50471613	rs11980407	IKZF1	dbSNP132/GRch37	63	1	R	A	1	1	1	W8	iPLEX	rs11980407	ACGTTGGATGCTGGAACTGAAGAAAGGAAG	ACGTTGGATGGCCAAAACAAACGCGAGATG	99	98.6	77	49	34.8		F	7055.6	AACTGGACTTATGTAGACAAATC	A	7326.8	AACTGGACTTATGTAGACAAATCA	G	7342.8	AACTGGACTTATGTAGACAAATCG
rs11575518	7	50535681	rs11575518	DDC	dbSNP132/GRch37	63	1	R	G	1	1	1	W8	iPLEX	rs11575518	ACGTTGGATGAAACGGCCTTCCTCTCATTG	ACGTTGGATGCTAGGATCACATGCTGTCTG	115	99.1	77	47	28		R	7934.2	tAATAGCTTCTGAGTTCTATAACTAT	G	8181.4	tAATAGCTTCTGAGTTCTATAACTATC	A	8261.3	tAATAGCTTCTGAGTTCTATAACTATT

Additional File LFST1B: Details of the SNP assays and multiplexes (SNP groups) designed for the Sequenom (Agena Bioscience; http://agenabio.com/iplex-genotyping) iPLEX platform. Each multiplex is highlighted by colour and are separated by blank rows. SNPs are ordered according to multiplex and the universal extemsion primer (UEP) mass (Daltons). Headings with an orange background provide details of each SNP in the human genome, while headings with a grey background are part of the assay design file. Further details of the assay design file specification can be obtained from Sequenom (Agena Bioscience).
