# Supplementary material for: Genetic determinants of anti-malarial acquired immunity in a large multi-centre study
Source: Malar J. 2015 Aug 28;14:333. doi: 10.1186/s12936-015-0833-x (PMC4552443; doi:10.1186/s12936-015-0833-x)
Supplement: Additional file 4: — MalariaGEN Supplementary Sample Handling Procedures. This file includes more detail on sample handling procedures including extracting DNA, genotyping methodology, ELISA protocols with ELISA data processing. [file 12936_2015_833_MOESM4_ESM.pdf]

Additional File 4: Supplementary Methods

Genetic Determinants Of Anti-Malarial Acquired Immunity In A Large Multi-Centre Study

Jennifer M.G. Shelton, Patrick Corran, Paul Risley, Nilupa Silva, Christina Hubbart, Anna Jeffreys, Kate Rowlands, Rachel Craik, Victoria Cornelius, Meike Hensmann, Sile Molloy, Nuno Sepulveda, Taane G. Clark, Gavin Band, Geraldine M. Clarke, Christopher C.A. Spencer, Angeliki Kerasidou, Susana Campino, Sarah Auburn, Adama Tall, Alioune Badara Ly, Odile Mercereau-Puijalon, Anavaj Sakuntabhai, Abdoulaye Djimde, Boubacar Maiga, Ousmane Toure, Ogobara Doumbo, Amagana Dolo, Marita Troye-Blomberg, Valentina D. Mangano, Frederica Verra, David Modiano, Edith Bougouma, Sodiomon B. Sirima, Muntaser Ibrahim, Ayman Hussain, Nahid Eid, Abier Elzein, Hiba Mohamed, Ahmed Elhassan, Ibrahim Elhassan, Thomas N. Williams, Carolyne Ndila, Alexander Macharia, Kevin Marsh, Alphaxard Manjurano, Hugh Reyburn, Martha Lemnge, Deus Ishengoma, Richard Carter, Nadira Karunaweera, Deepika Fernando, Rajika Dewasurendra, Christopher J. Drakeley, Eleanor M. Riley, Dominic P. Kwiatkowski, and Kirk A. Rockett, in collaboration with the MalariaGEN Consortium,

Corresponding authors Kirk A. Rockett and Dominic P. Kwiatkowski
Wellcome Trust Centre for Human Genetics, University of Oxford, Roosevelt Drive, Oxford, UK

This file contains the MalariaGEN Supplementary Sample Handling Procedures with further details of the genotyping and ELISA methodologies used in this study.

MalariaGEN Sample Handling Procedures

	Page
Sample Archiving	3
• Sample Collection	3
• DNA extraction	3
• Sample Processing	3
Primer-extension Amplification (PEP)	3
• PEP reaction	3
• PEP testing	4
Genotyping	4
• Platform	4
• SNP sets	4
• iPLEX design	5
• Sample preparation	5
• iPLEX primers	5
• First-Round reaction master-mix	5
• First-Round Reaction	5
• Shrimp-alkaline Phosphatase treatment	6
• Primer-Extension Reaction	6
• Agena Biosciences Assay details	6
ELISA Methodology	7
• Antigens	7
• ELISA	7
• Table 1 Antigen Comparison	8
• Figure 1: Comparison of antigens by parallel ELISA	8
• Figure 2: ELISA curve fitting	8
References	9
• References	9

MalariaGEN Sample Handling Procedures

Sample Archiving:

Sample Collection:

Blood was collected from individuals typically by venupuncture into a non-heparin anti-coagulant (typically EDTA); volumes varied between <1ml to 10ml depending on clinical circumstances and ethical permissions.

DNA extraction:

The blood was processed locally to extract DNA using the local method of choice; either Nucleon™ BACC Genomic DNA Extraction Kits (Gen-Probe Life Sciences Ltd, Tepnel Research Products & Services, Manchester, UK [1]), or Qiagen DNeasy Blood kits (Qiagen, Crawley, UK [2]). Extractions were carried out according manufacturers' instructions although some local changes may have been made to the protocols to suit local conditions.

Sample Processing:

DNA was shipped frozen to Oxford. After arrival, the sample manifest was confirmed and all samples were relabeled and recoded with new sample_codes according to a standard format bearing no relationship to the original coding. Sample volumes were recorded and the DNA concentrations were measured using the PicoGreen® reagent (Invitrogen, Paisley, UK [3]). An aliquot from each sample was diluted to 20ng/ul where possible to provide a 'working' sample allowing the remaining stock sample to be stored with little disturbance; an aliquot from samples below 20ng/ul was taken and used 'as is'. All DNAs were stored at -80°C in screw-cap tubes with rubber 'O' ring seals (Greiner Bio-One, Stonehouse, UK; 0.5ml skirted tubes #693201-1, lids #366380-1 and 9x9 format boxes #TR81N [4]).

Primer-extension Amplification (PEP):

PEP reaction:

Samples underwent a whole-genome amplification step using Primer-Extension Pre-Amplification as previously described [5]. gDNA was diluted to 1ng/ul in 96-well plates (Thermo-Fast® 96-skirted, Thermo Fisher Scientific, UK [6]), leaving 2 to 3 empty wells for water controls.

A PCR reagent mixture of 45ul comprising;

2.2ul of 1:10 diluted N15 primers (Genetix Ltd, UK [7]),
1.25ul 8mM pooled dNTP's (Sigma-Aldrich,UK [8]),
2.5ul 50mM $MgCl_2$ (Bioline, UK [9]),
5ul of 10X BioTaq buffer (Bioline, UK [9]),
0.5ul 5U/ul Biotaq polymerase (Bioline [9]),
33.55ul MilliQ water (Sigma-Aldrich,UK [8]).

were added to each well of a 96-well skirted PCR plate (Thermo Fisher Scientific). Five microlitres of gDNA (1ng/ul) was added to the PEP PCR mixture and the plates were sealed with Flat-Cap Strips (Thermo fisher Scientific) before thermocycling using a MJ Tetrad (Bio-Rad, UK) with the following programme:

94°C for 3 min;
50 cycles of: 94 °C for 1min,
37 °C for 2 min
Ramp to 55°C at 0.1/sec
55 °C for 4 min

and a final extension of 72°C for 5 min,
maintain at 4°C

PEP DNA was stored neat at -20°C until used.

PEP testing:

Twelve samples were selected at random from the plate of PEP reactions prepared above. PCR reactions were prepared as described below for Agena Biosciences (formerly Sequenom) genotyping [10] except that the final reaction volume was 20ul; 1ul of neat PEP was used and a single primer pair designed from an existing iPLEX assay design was used:

forward primer: ACGTTGGATGTCTGTAGTGATGGAGGGATG

reverse primer: ACGTTGGATGGTGTCCTCTCCCTTGTAAAC

Samples were run on 2% Agarose gels to check band intensity and fidelity.

Genotyping:

Platform:

The genotyping methodology chosen was SEQUENOM® iPLEX® Gold which allowed up to 40 SNPs to be designed into a single reaction (multiplex) and for up to 384 samples to be processed on one chip [10]. All reagents specific for this process were purchased from SEQUENOM®. Other reagents used were purchased as described below.

SNP sets:

Genotyping was undertaken for all samples upon receipt in Oxford for a set of SNPs designed as part of the QC process or with relevance to malaria.

Our core SNP set plus other SNPs is shown in Additional File 3. These were identified from literature searches in publications showing associations of SNPs with malaria infection/disease severity/antibody/parasite levels. To these were added assays designed to determine gender by comparing the Amelogenin gene between the X and Y chromosomes [11]. Other SNPs from research being undertaken in the laboratory at the time were added to complete the multiplex design process.

iPLEX design:

Polymorphism sequence information was downloaded from Ensembl [12] and reformatted for the Agena Biosciences assay design process [10]. The Agena Biosciences OnLine Tools [13] tools ProxSNP and PreEXTEND were used to identify proximal SNPs in the region of the target SNPs and to mask and design first–round PCR primers (Amplicon Design). Multiplex design for the iPLEX methodology was then undertaken using the MassARRAY® Assay Design v3.1 Software. Common settings for assay design included the addition of a universal 10 base 5’ sequences and then

at least 20 bases of sequence-specific bases. All first round reactions were designed for an average of amplicon of 100-bases pairs and ranging between 80 and 120 bases. Universal extension primers were designed with a mass range of 4500Da to 10,000Da (~15-mer to ~29-mer oligos).

These multiplexes were then tested using a panel of CEPH and YRI HapMap DNAs. Poorly performing assays or poor concordance assays were removed from the multiplex.

Details of the final multiplexes are provided in Additional File 3.

Sample preparation:

PEP DNA samples were diluted 1:10 using a phenol red solution (0.01mg/ml) to aid tracking into 384-well plates (yellow/red to purple colour change); 22.5ul of phenol red solution plated into each well of a 384-well plate and 2.5ul of neat PEP was added. An aliquot of diluted PEP was then immediately used for the first-round PCR reactions as described below. Unused diluted PEP was frozen at -20°C. NB: Diluted PEP kept at 4°C for more than 2 days or freeze-thawed more than twice was discarded as this was found too degraded for genotyping .

iPLEX primers:

All primers were purchased lyophilised from Metabion International AG (Martinsried, Germany [14]).

First-round primers were hydrated at 100 uM and extension primers were hydrated at 300 uM. All primers were stored at -20°C.

First-Round reaction master-mix:

A master-mix comprising the following was prepared for each 384-well plate allowing some extra volume;

3.3 ul of each first-round primer (100mM),
214.5 ul $MgCl_2$ (50mM),
66 ul dNTPs (25mM pooled),
412.5 ul 10X HotStar Taq buffer (Qiagen),
132 ul HotStar Taq (5U/ul) (Qiagen) and
milliQ water to make a final volume of 1980ul.

First-Round Reaction:

PCR master mix (4.5 ul per well) was plated into a 384-well PCR plate (Thermo Fisher Scientific) and 3 ul of 1:10 diluted PEP DNA were added per well. Plates were sealed with Microseal 'A' lids (Bio-Rad) and cycled on an MJ Tetrad with the following conditions:

94 °C for 15 min,
44 cycles of; 94 °C for 20 sec,
56 °C for 30 sec,
72 °C for 1 min,
and 72°C for 3 min
maintain at 4°C

A 1ul sample from each well of a single row was run on a 2% agarose gel to confirm the PCR had worked prior to further processing.

Shrimp-alkaline Phosphatase treatment:

Unincorporated dNTP's were destroyed by adding 2ul of iPLEX shrimp-alkaline phosphatase (SAP) mixture to 5ul of first-round PCR reaction mixture and incubating at 37 °C for 40 min followed by a denaturation step at 85 °C for 5 min and then cooling to 15°C for 15 min.

Primer-Extension Reaction:

Extension-primer final reaction concentrations were dependent on their molecular mass based on Agena Biosciences protocol guidelines;

< 5800Da 0.84 uM,
5800 to 7000Da 1.04 uM,
7000 to 10,000 Da 1.25 uM,
>10,000Da 1.5uM.

Primer extension was carried out in the sample plate by adding 2 ul per well of a mixture containing;

0.2ul iPLEX termination mixture,
0.041ul extension Taq,
0.2ul extension buffer,
Primers (300mM);
    0.025ul per primer up to 5800Da,
    0.0312 ul per primer 5800 to 7000Da,
    0.0375 ul per primer 7000 to 10,000 Da
    0.045 ul per primer >10,000 Da
and water to 2ul.

The final extension reaction volume was 9 ul (5ul first-round reaction, 2ul SAP and 2ul of extension mixture).

Extension cycling was undertaken on an MJ Tetrad using the following conditions:

94 °C for 30 sec,
40 cycles of;
    94°C for 5 sec,
        5 cycles of;
            52 °C for 5 sec,
            80 °C for 5 sec,
    then 72°C for 3 min
    and 15°C for 15 min.

Plates were processed by adding 6 mg ion-exchange resin per well and 16ul MilliQ water. Plates were sealed, rotated for 30min and then centrifuged to pellet the resin prior to 'spotting' samples onto SpectroCHIPS and running on the Mass-Spectrometer. Data were inspected and genotypes checked using the Agena Biosciences Typer 4.03 software. Data were downloaded and stored in a central database where any further curation was undertaken. All genotype data were maintained according to the sequence strand used for the assay design process.

Agena Biociences Assay details:

Additional File 3 contains information on the primers and assays designs.

ELISA methodology

Antigens

For one of the sites, Tanzania (Moshi) immunoassays had been carried out beforehand using different expressed forms of the same antigens [15], but for only three of the assays: AMA1 (3D7), $MSP1_{19}$ (Wellcome) and MSP2 (3D7): the antigens are compared in table 1 below. These antigens were directly compared with the MalariaGEN versions using a pool of 40 African adult sera. The Moshi AMA1 (3D7) and $MSP1_{19}$ (Wellcome) antigens were very closely comparable (OD r^2>=0.95), the MSP2 (3D7) less so, but still well correlated (r^2 = 0.52). In particular, a subset (5/40) of samples showed significantly poorer (20-50%) response to the Moshi MSP-2 antigen than to the MalariaGEN version. With this caveat, the results were incorporated into the MalariaGEN database.

ELISA

ELISA plates (Immulon 4-HBX, Fisher Scientific UK Ltd, Loughborough, UK) were coated with 50µl of antigen (AMA1, MSP2 and IgE at 0.5µg/mL dilution; $MSP1_{19}$ and $(NANP)_4$ at 1µg/mL dilution) or anti-human IgE MAb (M107 from Mabtech AB, Nacka Strand, Sweden) (1µg/ml in 0.2M sodium carbonate pH9.6). Plates were incubated overnight at 4°C and plates were washed x3 with PBS-0.05% Tween 20 (PBS/T) (Sigma, Gillingham, Dorset, UK). Blocking solution (200µl of 2% skimmed milk powder in PBS/T) was added to each well and plates were incubated for 3 hours at ambient temperature and plates washed 3 times with PBS/T. Ten microlitres of each characterisation sample was diluted with 190 µl blocking solution in a 0.5 ml deep-well plate (total sample dilution 1:200). Aliquots of these wells were then added in duplicate to the coated plates to which an appropriate volume of dilutent blocking buffer had previously been added to give an additional in-well dilution as follows : For $(NANP)_4$ 50 µl was added with no diluent blocking solution (final dilution 1:200); for MSP2, $MSP1_{19}$ and IgE, 10 µl was added to 40 µl blocking solution (final dilution 1:1000); and for AMA1 10 µl was added to 90 µl (final dilution 1:2000). All plates contained (in duplicate) a fivefold diution series standard curve, a negative serum control and a blank (without serum). Plates were incubated overnight 4°C and next day were washed six times with PBS/T, horseradish peroxidise-conjugated rabbit anti-human IgG (DAKO) (50µl of 1:5000 in PBS/T) added to each well and plates incubated for 3 hours at room temperature. After washing six times with PBS/T, Sigma-Fast o-phenylenediamine dihydrochloride (OPD) reagent solution (Sigma) (100µl) was added to each well. Plates were left at room temperature for 10-15 minutes (20-30 minutes for $(NANP)_4$ ELISA) before the addition of 25µl 2M H_2SO_4 to stop the reaction. Plates were read in a plate reader (Molecular Devices, Wokingham, Berkshire, UK) at 492nm. The averaged OD readings of the standard dilution series were used to construct a standard curve for each plate using a simple three parameter ligand-binding model [16] : maximum, minimum and mid-point values of the curve were fitted by least-squares minimisation using the Solver plugin in Excel® (Microsoft, Reading, UK). To minimise plate-to-plate variations, all plate ODs were normalised by scaling the maximum OD to 2.5. The fitted parameters were then used to calculate the antibody concentration of each sample in terms of the reference standard, which in the case of the malaria antigens was given an arbitrary value of 1000 U/ml for all antigens. Significant numbers of samples gave OD<minimum resulting in apparent small negative concentrations: these represented natural variability around zero, and were allowed to remain in order to avoid perturbing the distribution. However some diluted sera gave OD>estimated maximum of the standard curve: in cases where OD was too high for reliable determination (>0.975 × maximum) a dummy value of 50000 × midpoint concentration for that plate was assigned to the sample.

Table 1 antigen comparison

Antigen	AMA1		$MSP1_{19}$		MSP2	
	**MalariaGEN**	**Moshi**	**MalariaGEN**	**Moshi**	**MalariaGEN**	**Moshi**
Sequence	3D7	3D7	Wellcome	Wellcome	3D7	3D7
Expression system	Yeast *(Pichia pastoris)*	Bacterial *(E.coli)*	Baculovirus/insect cell	Bacterial *(E.coli)*	Bacterial *(E.coli)*	Bacterial *(E.coli)*
Modifications	none	His-tag	none	GST fusion	His-tag	GST fusion
References	[17]	[18]	[19]	[20]	[21]	[22]

Figure 1: Comparison of antigens by parallel ELISA

Plates were coated with the above antigens as described below and the same 1:1000 dilutions of a series of 40 East and West African samples used to fill each plate in duplicate. No standard was included. All other details were as below. Averaged duplicate results for each pair of antigens are plotted against each other.

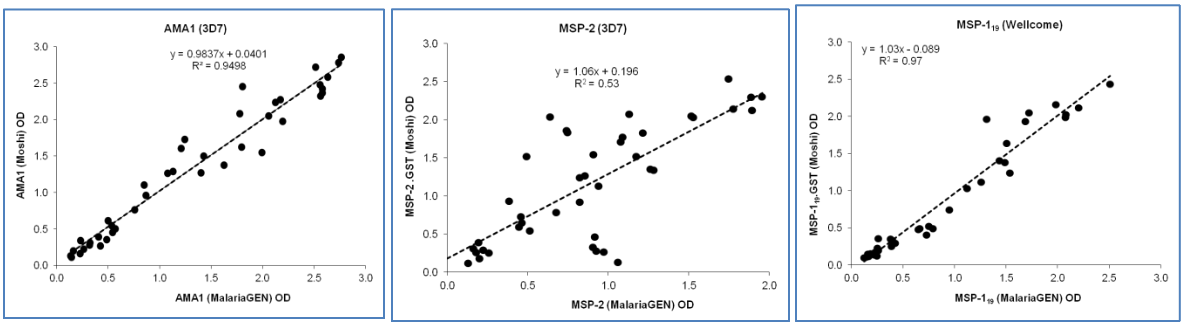

Figure 2: ELISA curve fitting

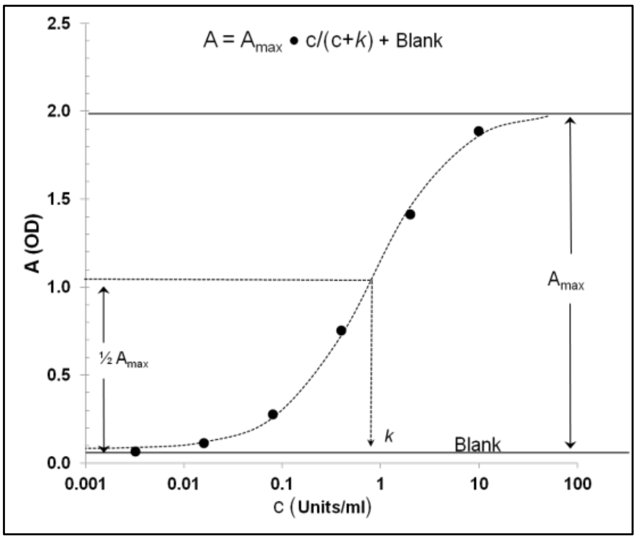

Where A is observed OD, A_{max} the OD at saturated antibody concentration, c is the concentration of the antibody at *k* the mean equilibrium dissociation constant of antibody and antigen equal to the antibody concentration at half maximum. The points are five-fold dilution series of the reference standard vs MSP-2 fitted as described above.

References

1. **Tepnel BACC DNA extraction kits** [http://www.gen-probe.com/pdfs/downloads/protocol%20BACC%20123%5B1%5D.pdf]
2. **QIAGEN** [https://www.qiagen.com/]
3. **Invitrogen** [http://www.probes.com]
4. **Greiner Bio-One** [http://www.greinerbioone.com]
5. Zhang L, Cui X, Schmitt K, Hubert R, Navidi W, Arnheim N: **Whole genome amplification from a single cell: implications for genetic analysis.** *Proc Natl Acad Sci U S A* 1992, **89:**5847-5851.
6. **ThermoFisher** [http://www.thermofisher.com/en/home.html]
7. **Genetix Ltd** [http://www.genetix.com]
8. **Sigma-Aldrich** [https://www.sigmaaldrich.com]
9. **Bioline** [http://www.bioline.com]
10. **Agena Biosciences** [http://agenabio.com/]
11. Eng B, Ainsworth P, Waye JS: **Anomalous migration of PCR products using nondenaturing polyacrylamide gel electrophoresis: the amelogenin sex-typing system.** *J Forensic Sci* 1994, **39:**1356-1359.
12. **Ensembl** [http://www.ensembl.org]
13. **Agena Biosciences OnLine Tools** [https://mysequenom.com/Home]
14. **Metabion** [http://www.metabion.com/home/index.php]
15. Drakeley CJ, Corran PH, Coleman PG, Tongren JE, McDonald SL, Carneiro I, et al: **Estimating medium- and long-term trends in malaria transmission by using serological markers of malaria exposure.** *Proc Natl Acad Sci U S A* 2005, **102:**5108-5113.
16. Corran PH, Cook J, Lynch C, Leendertse H, Manjurano A, Griffin J, et al: **Dried blood spots as a source of anti-malarial antibodies for epidemiological studies.** *Malar J* 2008, **7:**195.
17. Kennedy MC, Wang J, Zhang Y, Miles AP, Chitsaz F, Saul A, et al: **In vitro studies with recombinant Plasmodium falciparum apical membrane antigen 1 (AMA1): production and activity of an AMA1 vaccine and generation of a multiallelic response.** *Infect Immun* 2002, **70:**6948-6960.
18. Riley EM, Wagner GE, Ofori MF, Wheeler JG, Akanmori BD, Tetteh K, et al: **Lack of association between maternal antibody and protection of African infants from malaria infection.** *Infect Immun* 2000, **68:**5856-5863.
19. Garraud O, Diouf A, Holm I, Nguer CM, Spiegel A, Perraut R, et al: **Secretion of parasite-specific immunoglobulin G by purified blood B lymphocytes from immune individuals after in vitro stimulation with recombinant Plasmodium falciparum merozoite surface protein-119 antigen.** *Immunology* 1999, **97:**204-210.
20. Burghaus PA, Holder AA: **Expression of the 19-kilodalton carboxy-terminal fragment of the Plasmodium falciparum merozoite surface protein-1 in Escherichia coli as a correctly folded protein.** *Mol Biochem Parasitol* 1994, **64:**165-169.
21. Saul A, Lawrence G, Smillie A, Rzepczyk CM, Reed C, Taylor D, et al: **Human phase I vaccine trials of 3 recombinant asexual stage malaria antigens with Montanide ISA720 adjuvant.** *Vaccine* 1999, **17:**3145-3159.
22. Tongren JE, Drakeley CJ, McDonald SL, Reyburn HG, Manjurano A, Nkya WM, et al: **Target antigen, age, and duration of antigen exposure independently regulate immunoglobulin G subclass switching in malaria.** *Infect Immun* 2006, **74:**257-264.
