# Supplementary material for: Genetic determinants of anti-malarial acquired immunity in a large multi-centre study
Source: Malar J. 2015 Aug 28;14:333. doi: 10.1186/s12936-015-0833-x (PMC4552443; doi:10.1186/s12936-015-0833-x)

ADDITIONAL FILE 5: SUPPLEMENTARY FIGURES

# Genetic Determinants Of Anti-Malarial Acquired Immunity In A Large Multi-Centre Study

Jennifer M.G. Shelton, Patrick Corran, Paul Risley, Nilupa Silva, Christina Hubbart, Anna Jeffreys, Kate Rowlands, Rachel Craik, Victoria Cornelius, Meike Hensmann, Sile Molloy, Nuno Sepulveda, Taane G. Clark, Gavin Band, Geraldine M. Clarke, Christopher C.A. Spencer, Angeliki Kerasidou, Susana Campino, Sarah Auburn, Adama Tall, Alioune Badara Ly, Odile Mercereau-Puijalon, Anavaj Sakuntabhai, Abdoulaye Djimde, Boubacar Maiga, Ousmane Toure, Ogobara Doumbo, Amagana Dolo, Marita Troye-Blomberg, Valentina D. Mangano, Frederica Verra, David Modiano, Edith Bougouma, Sodiomon B. Sirima, Muntaser Ibrahim, Ayman Hussain, Nahid Eid, Abier Elzein, Hiba Mohamed, Ahmed Elhassan, Ibrahim Elhassan, Thomas N. Williams, Carolyne Ndila, Alexander Macharia, Kevin Marsh, Alphaxard Manjurano, Hugh Reyburn, Martha Lemnge, Deus Ishengoma, Richard Carter, Nadira Karunaweera, Deepika Fernando, Rajika Dewasurendra, Christopher J. Drakeley, Eleanor M. Riley, Dominic P. Kwiatkowski, and Kirk A. Rockett,  in collaboration with the MalariaGEN Consortium,

Corresponding authors Kirk A. Rockett  and Dominic P. Kwiatkowski
Wellcome Trust Centre for Human Genetics, University of Oxford, Roosevelt Drive, Oxford, UK

This file contains **Additional Figure SF1:** Histograms of logged antibody levels before further manipulations were made.

*The group of individuals (n=216) in the MSP-1 dataset at $\log_{10}$ 4.85 were identified as outliers almost certainly due to difficulties in measuring their titres.  These samples were removed from the dataset and any further analyses.

**Additional Figure SF1:** Histograms of logged antibody levels before further manipulations were made.
*The group of individuals (n=216) in the MSP-1 dataset at $log_{10}$ 4.85 were identified as outliers almost certainly due to difficulties in measuring their titres. These samples were removed from the dataset and any further analyses.

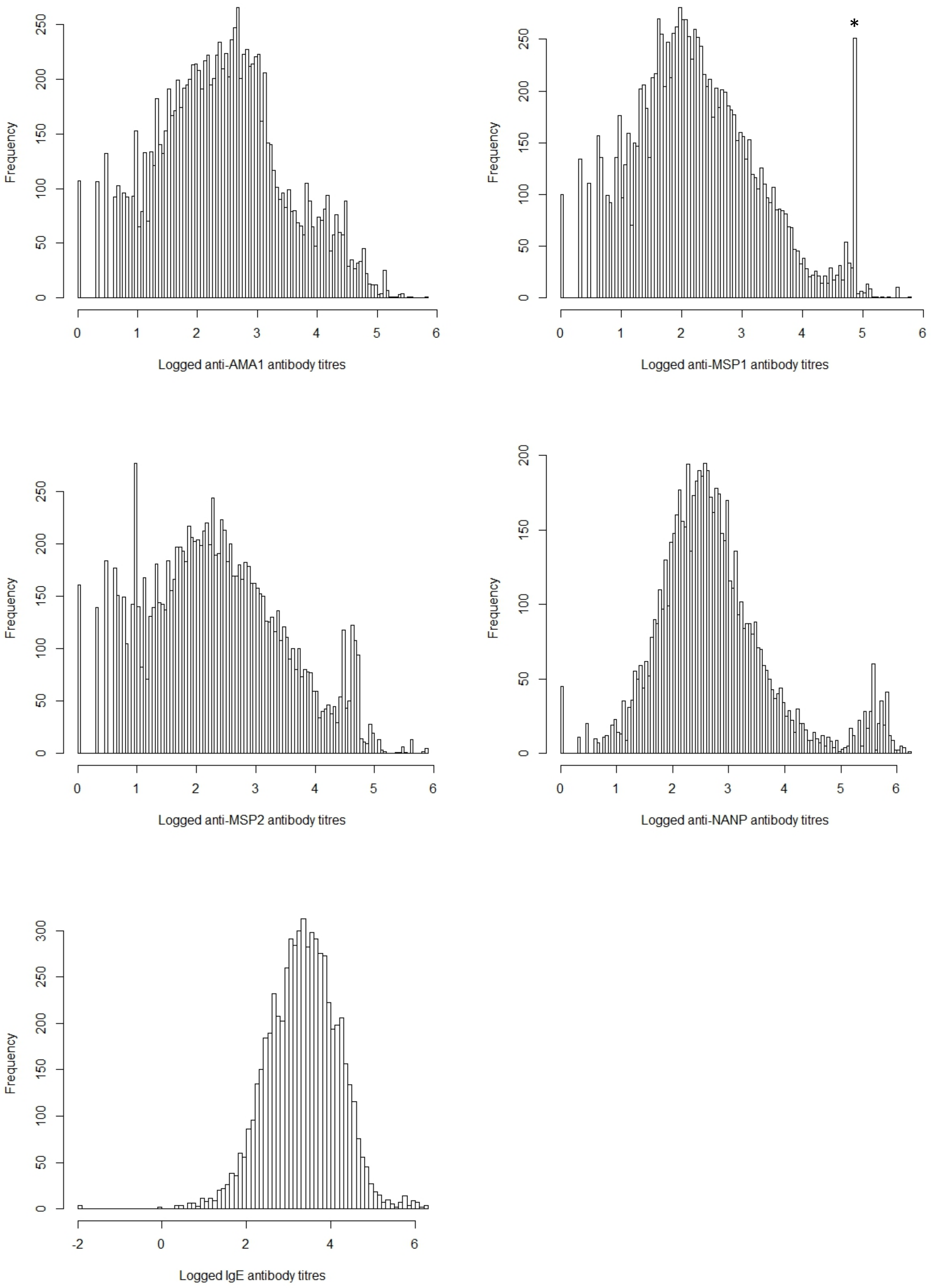

Supplement: Additional file 5: — Additional Figure SF1: Histograms of logged antibody levels before further manipulations were made. Histograms of the logged antibody titres across all samples for each antibody measured in this study. [file 12936_2015_833_MOESM5_ESM.pdf]
