# Supplementary material for: Genetic determinants of anti-malarial acquired immunity in a large multi-centre study
Source: Malar J. 2015 Aug 28;14:333. doi: 10.1186/s12936-015-0833-x (PMC4552443; doi:10.1186/s12936-015-0833-x)
Supplement: Additional file 7: — Additional Table ST3A-D: Details of the linear regression models used in this study. Model formulae for the various linear regression analyses showing how the different co-variates were used. [file 12936_2015_833_MOESM7_ESM.pdf]

ADDITIONAL FILE 7: SUPPLEMENTARY TABLES

Genetic Determinants Of Anti-Malarial Acquired Immunity In A Large Multi-Centre Study

Jennifer M.G. Shelton, Patrick Corran, Paul Risley, Nilupa Silva, Christina Hubbart, Anna Jeffreys, Kate Rowlands, Rachel Craik, Victoria Cornelius, Meike Hensmann, Sile Molloy, Nuno Sepulveda, Taane G. Clark, Gavin Band, Geraldine M. Clarke, Christopher C.A. Spencer, Angeliki Kerasidou, Susana Campino, Sarah Auburn, Adama Tall, Alioune Badara Ly, Odile Mercereau-Puijalon, Anavaj Sakuntabhai, Abdoulaye Djimde, Boubacar Maiga, Ousmane Toure, Ogobara Doumbo, Amagana Dolo, Marita Troye-Blomberg, Valentina D. Mangano, Frederica Verra, David Modiano, Edith Bougouma, Sodiomon B. Sirima, Muntaser Ibrahim, Ayman Hussain, Nahid Eid, Abier Elzein, Hiba Mohamed, Ahmed Elhassan, Ibrahim Elhassan, Thomas N. Williams, Carolyne Ndila, Alexander Macharia, Kevin Marsh, Alphaxard Manjurano, Hugh Reyburn, Martha Lemnge, Deus Ishengoma, Richard Carter, Nadira Karunaweera, Deepika Fernando, Rajika Dewasurendra, Christopher J. Drakeley, Eleanor M. Riley, Dominic P. Kwiatkowski, and Kirk A. Rockett, in collaboration with the MalariaGEN Consortium,

Corresponding authors Kirk A. Rockett and Dominic P. Kwiatkowski
Wellcome Trust Centre for Human Genetics, University of Oxford, Roosevelt Drive, Oxford, UK

This file contains:

Additional Table ST3A: Details of linear regression models used to investigate effect of non-genetic factors on logged antibody levels. All covariates were categorical except for parasite density, which was continuous.

Additional Table ST3B: Details of site-specific linear regression models used to investigate SNP-antibody associations. Models were formulated for each site separately based on data collected by site. Models were run for each SNP-antibody combination separately using four genetic models (additive, dominant, heterozygote and recessive) and results were combined using meta-analysis to give an overall beta and p-value for SNP effect. All covariates were categorical.

Additional Table ST3C: Details of logistic regression model used to investigate effect of SNPs with significant SNP-antibody associations on malaria infection. All covariates were categorical.

Additional Table ST3D: Details of linear regression model used to investigate effect of SNPs with significant SNP-antibody associations on parasite density. All covariates were categorical except for parasite density, which was continuous.

Additional Table ST3A: Details of linear regression models used to investigate effect of non-genetic factors on logged antibody levels. All covariates were categorical except for parasite density, which was continuous.

Regression model type	Data used	Outcome	Full model
Linear	*All data*	antibody levels	$\beta_1$age + $\beta_2$gender + $\beta_3$malaria status + $\beta_4$ethnicity + $\beta_5$village + $\beta_6$month + $\beta_7$study
	BJ, KM, TG, TP, SL	antibody levels	$\beta_1$age + $\beta_2$gender + $\beta_3$malaria status + $\beta_4$ethnicity + $\beta_5$village + $\beta_6$month + $\beta_7$study + $\beta_8$bednet use
	DP, DQ, BJ, TN, TG, TP	antibody levels	$\beta_1$age + $\beta_2$gender + $\beta_4$ethnicity + $\beta_5$village + $\beta_6$month + $\beta_7$study + $\beta_8$parasite density

Additional Table ST3B: Details of site-specific linear regression models used to investigate SNP-antibody associations. Models were formulated for each site separately based on data collected by site. Models were run for each SNP-antibody combination separately using four genetic models (additive, dominant, heterozygote and recessive) and results were combined using meta-analysis to give an overall beta and p-value for SNP effect. All covariates were categorical.

Regression model type	Data used	Outcome	Full model
Linear	*SE*	antibody levels	β_1SNP + $\beta_2$age + $\beta_3$gender + $\beta_4$ethnicity + $\beta_5$village
	DP	antibody levels	β_1SNP + $\beta_2$age + $\beta_3$gender + $\beta_4$ethnicity + $\beta_5$village + $\beta_6$month
	DQ	antibody levels	β_1SNP + $\beta_2$age + $\beta_3$gender + $\beta_4$malaria status + $\beta_5$ethnicity + $\beta_6$village
	BJ	antibody levels	β_1SNP + $\beta_2$age + $\beta_3$gender + $\beta_4$malaria status + $\beta_5$ethnicity + $\beta_6$village + $\beta_7$month
	SA	antibody levels	β_1SNP + $\beta_2$age + $\beta_3$gender + $\beta_4$ethnicity + $\beta_5$village
	KM	antibody levels	β_1SNP + $\beta_3$gender + $\beta_4$malaria status + $\beta_5$ethnicity + $\beta_6$month
	TN	antibody levels	β_1SNP + $\beta_2$age + $\beta_3$gender + $\beta_4$malaria status + $\beta_5$ethnicity + $\beta_6$village + $\beta_7$month
	TG	antibody levels	β_1SNP + $\beta_2$age + $\beta_3$gender + $\beta_4$malaria status + $\beta_5$ethnicity + $\beta_6$village + $\beta_7$month
	TP	antibody levels	β_1SNP + $\beta_2$age + $\beta_3$gender + $\beta_4$malaria status + $\beta_5$ethnicity + $\beta_6$village
	SL	antibody levels	β_1SNP + $\beta_2$age + $\beta_3$gender + $\beta_4$malaria status + $\beta_5$village + $\beta_6$month

Additional Table ST3C: Details of logistic regression model used to investigate effect of SNPs with significant SNP-antibody associations on malaria infection. All covariates were categorical.

Regression model type	Data used	Outcome	Full model
Logistic	*DQ, BJ, KM, TN, TG, TP*	malaria infection	β_1SNP + $\beta_2$age + $\beta_3$gender + $\beta_4$ethnicity + $\beta_5$village + $\beta_6$month + $\beta_7$study

Additional Table ST3D: Details of linear regression model used to investigate effect of SNPs with significant SNP-antibody associations on parasite density. All covariates were categorical except for parasite density, which was continuous.

Regression model type	Data used	Outcome	Full model
Linear	*DP, DQ, BJ, TN, TG, TP*	parasite density	β_1SNP + $\beta_2$age + $\beta_3$gender + $\beta_4$ethnicity + $\beta_5$village + $\beta_6$month + $\beta_7$study
