# Supplementary material for: Genetic determinants of anti-malarial acquired immunity in a large multi-centre study
Source: Malar J. 2015 Aug 28;14:333. doi: 10.1186/s12936-015-0833-x (PMC4552443; doi:10.1186/s12936-015-0833-x)
Supplement: Additional file 8: — Additional Table ST4: Results of linear regression analysis investigating the effect of age, gender and HbS genotype on parasite density in malaria-positive individuals using combined site data. Results of linear regression analysis investigating the effect of age, gender and HbS genotype on parasite density in malaria-positive individuals using combined data. This is an extension to that shown in the main text and is a reduced dataset as not all sites provided parasitaemia data. [file 12936_2015_833_MOESM8_ESM.pdf]

ADDITIONAL FILE 8: SUPPLEMENTARY TABLES

Genetic Determinants Of Anti-Malarial Acquired Immunity In A Large Multi-Centre Study

Jennifer M.G. Shelton, Patrick Corran, Paul Risley, Nilupa Silva, Christina Hubbart, Anna Jeffreys, Kate Rowlands, Rachel Craik, Victoria Cornelius, Meike Hensmann, Sile Molloy, Nuno Sepulveda, Taane G. Clark, Gavin Band, Geraldine M. Clarke, Christopher C.A. Spencer, Angeliki Kerasidou, Susana Campino, Sarah Auburn, Adama Tall, Alioune Badara Ly, Odile Mercereau-Puijalon, Anavaj Sakuntabhai, Abdoulaye Djimde, Boubacar Maiga, Ousmane Toure, Ogobara Doumbo, Amagana Dolo, Marita Troye-Blomberg, Valentina D. Mangano, Frederica Verra, David Modiano, Edith Bougouma, Sodiomon B. Sirima, Muntaser Ibrahim, Ayman Hussain, Nahid Eid, Abier Elzein, Hiba Mohamed, Ahmed Elhassan, Ibrahim Elhassan, Thomas N. Williams, Carolyne Ndila, Alexander Macharia, Kevin Marsh, Alphaxard Manjurano, Hugh Reyburn, Martha Lemnge, Deus Ishengoma, Richard Carter, Nadira Karunaweera, Deepika Fernando, Rajika Dewasurendra, Christopher J. Drakeley, Eleanor M. Riley, Dominic P. Kwiatkowski, and Kirk A. Rockett, in collaboration with the MalariaGEN Consortium,

Corresponding authors Kirk A. Rockett and Dominic P. Kwiatkowski
Wellcome Trust Centre for Human Genetics, University of Oxford, Roosevelt Drive, Oxford, UK

This file contains **Additional Table ST4: Results of linear regression analysis investigating the effect of age, gender and HbS genotype on parasite density in malaria-positive individuals* using combined data.** Data from Senegal, Kenya, Sudan and Sri Lanka are not included as parasite density was not recorded at these sites.

Additional Table ST4: Results of linear regression analysis investigating the effect of age, gender and HbS genotype on parasite density in malaria-positive individuals* using combined data. Data from Senegal, Kenya, Sudan and Sri Lanka are not included as parasite density was not recorded at these sites.

			Log_{10} Parasite density (n=2272)	
**Factor**	**beta (95% CI)**	**p-value**	**mean (95% CI)**	**no. of individuals**
Age (years)				
<1	0		2.80 (0.98 to 4.62)	61
1-2	**0.26 (0.01 to 0.52)**	**0.041**	3.35 (1.65 to 5.04)	65
2-5	0.07 (-0.13 to 0.28)	0.473	3.33 (1.57 to 5.10)	377
5-15	**-0.32 (-0.52 to -0.13)**	**0.001**	2.78 (1.15 to 4.42)	1126
15-30	**-0.55 (-0.75 to -0.34)**	**<0.001**	2.41 (1.02 to 3.80)	379
>30	**-0.62 (-0.84 to -0.41)**	**<0.001**	2.27 (0.61 to 3.94)	259
Gender				
Female	0		2.76 (0.96 to 4.57)	1210
Male	**0.09 (0.03 to 0.15)**	**0.005**	2.89 (1.19 to 4.60)	1062
HbS				
11	0		2.82 (1.05 to 4.59)	2115
12	0.09 (-0.04 to 0.22)	0.158	2.90 (1.21 to 4.59)	151
22	ND	ND	ND	1

NB: CI = confidence interval.
Results significant at 0.05 level are highlighted in bold.
* Also adjusted for village (>20), ethnicity (>20), sample month (>20) and study; results not shown.
ND: Results not shown as unable to obtain estimates from one HbSS individual.
