# Supplementary material for: Genetic determinants of anti-malarial acquired immunity in a large multi-centre study
Source: Malar J. 2015 Aug 28;14:333. doi: 10.1186/s12936-015-0833-x (PMC4552443; doi:10.1186/s12936-015-0833-x)

ADDITIONAL FILE 9: SUPPLEMENTARY FIGURES

Genetic Determinants Of Anti-Malarial Acquired Immunity In A Large Multi-Centre Study

Jennifer M.G. Shelton, Patrick Corran, Paul Risley, Nilupa Silva, Christina Hubbart, Anna Jeffreys, Kate Rowlands, Rachel Craik, Victoria Cornelius, Meike Hensmann, Sile Molloy, Nuno Sepulveda, Taane G. Clark, Gavin Band, Geraldine M. Clarke, Christopher C.A. Spencer, Angeliki Kerasidou, Susana Campino, Sarah Auburn, Adama Tall, Alioune Badara Ly, Odile Mercereau-Puijalon, Anavaj Sakuntabhai, Abdoulaye Djimde, Boubacar Maiga, Ousmane Toure, Ogobara Doumbo, Amagana Dolo, Marita Troye-Blomberg, Valentina D. Mangano, Frederica Verra, David Modiano, Edith Bougouma, Sodiomon B. Sirima, Muntaser Ibrahim, Ayman Hussain, Nahid Eid, Abier Elzein, Hiba Mohamed, Ahmed Elhassan, Ibrahim Elhassan, Thomas N. Williams, Carolyne Ndila, Alexander Macharia, Kevin Marsh, Alphaxard Manjurano, Hugh Reyburn, Martha Lemnge, Deus Ishengoma, Richard Carter, Nadira Karunaweera, Deepika Fernando, Rajika Dewasurendra, Christopher J. Drakeley, Eleanor M. Riley, Dominic P. Kwiatkowski, and Kirk A. Rockett, in collaboration with the MalariaGEN Consortium,

Corresponding authors Kirk A. Rockett and Dominic P. Kwiatkowski
Wellcome Trust Centre for Human Genetics, University of Oxford, Roosevelt Drive, Oxford, UK

This file contains **Additional figure SF3: Heatmap matrix plot of the correlations between residuals from the antibody linear regression models with non-genetic factors at each site.** Pairwise correlations between residuals calculated as R-squared. Strongest correlations shown in red and weakest correlations shown in yellow. Correlations of residuals with themselves shown here in grey.

Additional figure SF3: Heatmap matrix plot of the correlations between residuals from the antibody linear regression models with non-genetic factors at each site. Pairwise correlations between residuals calculated as R-squared. Strongest correlations shown in red and weakest correlations shown in yellow. Correlations of residuals with themselves shown here in grey.

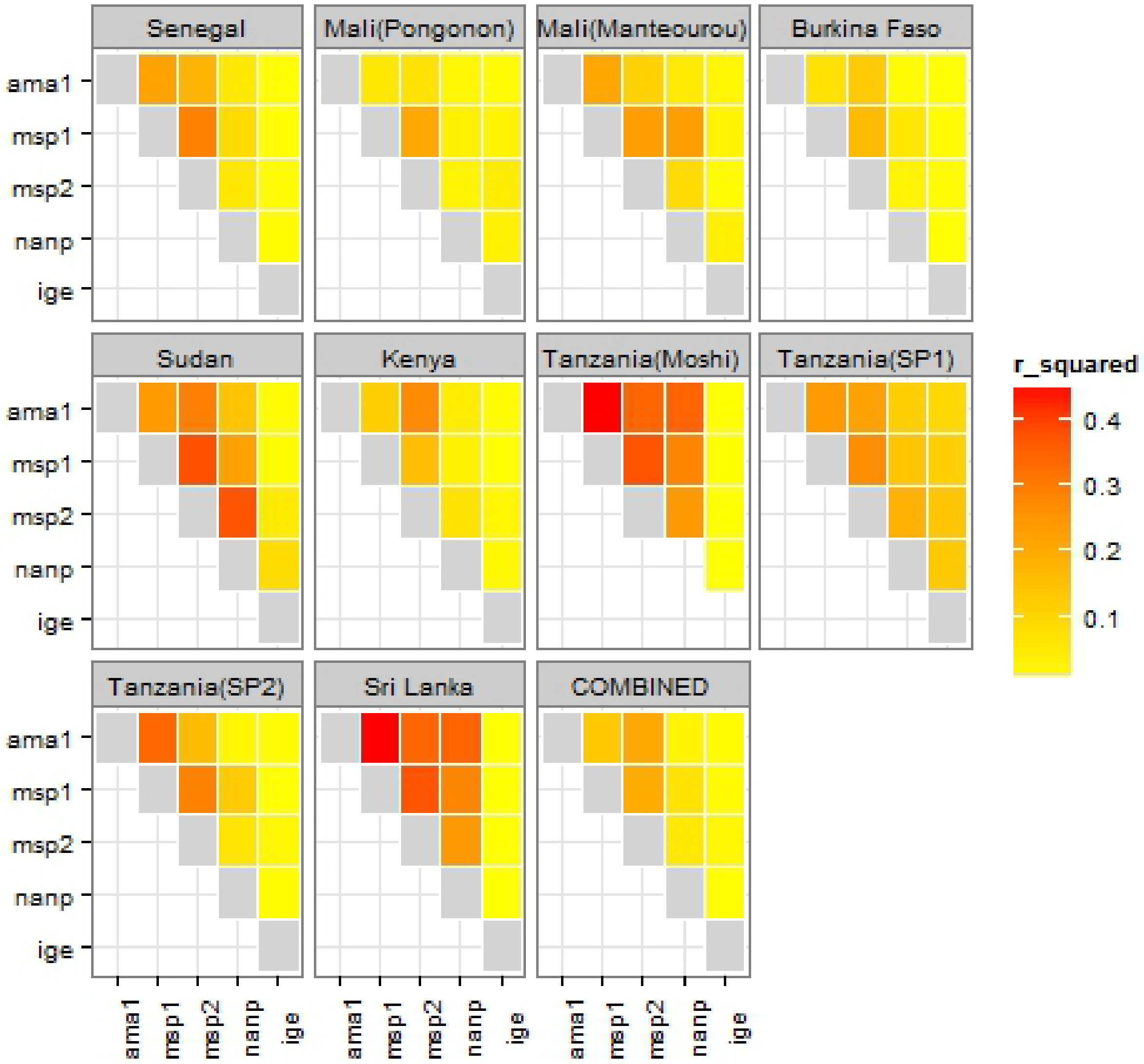

Supplement: Additional file 9: — Additional figure SF3: Heatmap matrix plot of the correlations between residuals from the antibody linear regression models with non-genetic factors at each site. Matrix plot of the pairwise correlations as r2 between antibody titres using data from all sites. [file 12936_2015_833_MOESM9_ESM.pdf]
