# Supplementary material for: Genetic determinants of anti-malarial acquired immunity in a large multi-centre study
Source: Malar J. 2015 Aug 28;14:333. doi: 10.1186/s12936-015-0833-x (PMC4552443; doi:10.1186/s12936-015-0833-x)

ADDITIONAL FILE 10: SUPPLEMENTARY FIGURES

Genetic Determinants Of Anti-Malarial Acquired Immunity In A Large Multi-Centre Study

Jennifer M.G. Shelton, Patrick Corran, Paul Risley, Nilupa Silva, Christina Hubbart, Anna Jeffreys, Kate Rowlands, Rachel Craik, Victoria Cornelius, Meike Hensmann, Sile Molloy, Nuno Sepulveda, Taane G. Clark, Gavin Band, Geraldine M. Clarke, Christopher C.A. Spencer, Angeliki Kerasidou, Susana Campino, Sarah Auburn, Adama Tall, Alioune Badara Ly, Odile Mercereau-Puijalon, Anavaj Sakuntabhai, Abdoulaye Djimde, Boubacar Maiga, Ousmane Toure, Ogobara Doumbo, Amagana Dolo, Marita Troye-Blomberg, Valentina D. Mangano, Frederica Verra, David Modiano, Edith Bougouma, Sodiomon B. Sirima, Muntaser Ibrahim, Ayman Hussain, Nahid Eid, Abier Elzein, Hiba Mohamed, Ahmed Elhassan, Ibrahim Elhassan, Thomas N. Williams, Carolyne Ndila, Alexander Macharia, Kevin Marsh, Alphaxard Manjurano, Hugh Reyburn, Martha Lemnge, Deus Ishengoma, Richard Carter, Nadira Karunaweera, Deepika Fernando, Rajika Dewasurendra, Christopher J. Drakeley, Eleanor M. Riley, Dominic P. Kwiatkowski, and Kirk A. Rockett, in collaboration with the MalariaGEN Consortium,

Corresponding authors Kirk A. Rockett and Dominic P. Kwiatkowski
Wellcome Trust Centre for Human Genetics, University of Oxford, Roosevelt Drive, Oxford, UK

This file contains **Additional figure SF4: Scatter plot of the correlations between residuals from the antibody linear regression models with non-genetic factors at each site and age-group.** Pairwise correlations between residuals are transformed to R-squared and are shown for malaria-malaria antibody pairs only. The malaria prevalence at each site (as percent microscopy positive blood smears) recorded at the time of the study are shown in square brackets after each site name. Sites are ordered according to malaria prevalence (low to high). Each panel show the data from each age-group. Both Kenya and Mali(Pongonon) are shown here but were omitted from the regression analysis (Additional File 11; Kenya has only 1 age-group and Mali[Pononon] individuals were all selected for being malaria positive).

Additional figure SF4: Scatter plot of the correlations between residuals from the antibody linear regression models with non-genetic factors at each site and age-group. Pairwise correlations between residuals are transformed to R-squared and are shown for malaria-malaria antibody pairs only. The malaria prevalence at each site (as percent microscopy positive blood smears) recorded at the time of the study are shown in square brackets after each site name. Sites are ordered according to malaria prevalence (low to high). Each panel show the data from each age-group. Both Kenya and Mali(Pongonon) are shown here but were omitted from the regression analysis (Additional File 11; Kenya has only 1 age-group and Mali[Pononon] individuals were all selected for being malaria positive).

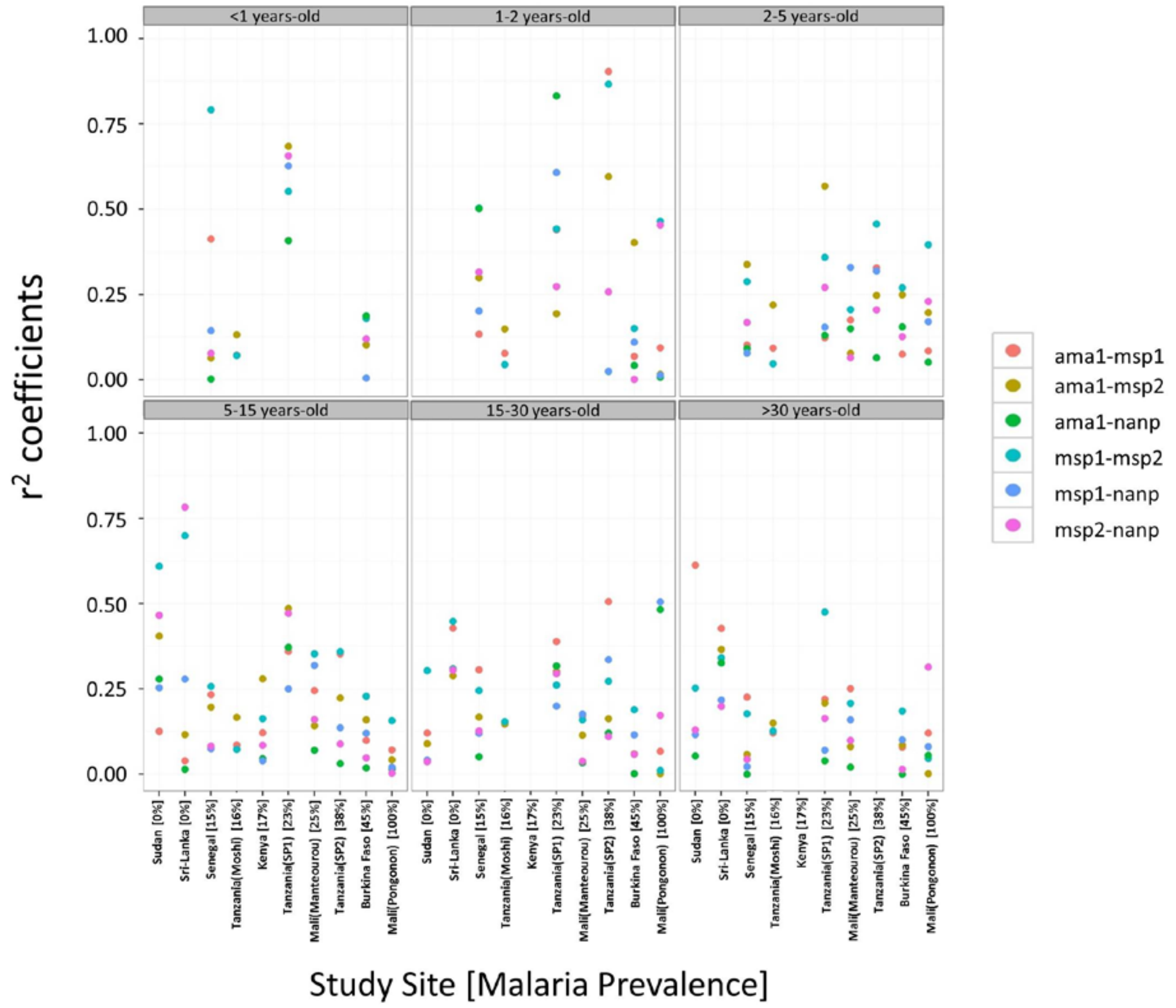

Supplement: Additional file 10: — Additional figure SF4: Scatter plot of the correlations between residuals from the antibody linear regression models with non-genetic factors at each site and age-group. This plot shows the pairwise correlations (as r2) for each malaria–malaria antibody pair as a function of each study site. The sites are ordered according to malaria microscopically-positive-prevalence recorded at sample collection (low to high). Several panels are shown according to age-group. [file 12936_2015_833_MOESM10_ESM.pdf]
