# Supplementary material for: Genetic determinants of anti-malarial acquired immunity in a large multi-centre study
Source: Malar J. 2015 Aug 28;14:333. doi: 10.1186/s12936-015-0833-x (PMC4552443; doi:10.1186/s12936-015-0833-x)
Supplement: Additional file 11: — Additional table ST5: Linear regression analysis of the correlations between residuals from the malaria antibody pairs. This data shows the results of the analysis of the correlations between antibodies. [file 12936_2015_833_MOESM11_ESM.pdf]

ADDITIONAL FILE 11: SUPPLEMENTARY TABLES

Genetic Determinants Of Anti-Malarial Acquired Immunity In A Large Multi-Centre Study

Jennifer M.G. Shelton, Patrick Corran, Paul Risley, Nilupa Silva, Christina Hubbart, Anna Jeffreys, Kate Rowlands, Rachel Craik, Victoria Cornelius, Meike Hensmann, Sile Molloy, Nuno Sepulveda, Taane G. Clark, Gavin Band, Geraldine M. Clarke, Christopher C.A. Spencer, Angeliki Kerasidou, Susana Campino, Sarah Auburn, Adama Tall, Alioune Badara Ly, Odile Mercereau-Puijalon, Anavaj Sakuntabhai, Abdoulaye Djimde, Boubacar Maiga, Ousmane Toure, Ogobara Doumbo, Amagana Dolo, Marita Troye-Blomberg, Valentina D. Mangano, Frederica Verra, David Modiano, Edith Bougouma, Sodiomon B. Sirima, Muntaser Ibrahim, Ayman Hussain, Nahid Eid, Abier Elzein, Hiba Mohamed, Ahmed Elhassan, Ibrahim Elhassan, Thomas N. Williams, Carolyne Ndila, Alexander Macharia, Kevin Marsh, Alphaxard Manjurano, Hugh Reyburn, Martha Lemnge, Deus Ishengoma, Richard Carter, Nadira Karunaweera, Deepika Fernando, Rajika Dewasurendra, Christopher J. Drakeley, Eleanor M. Riley, Dominic P. Kwiatkowski, and Kirk A. Rockett, in collaboration with the MalariaGEN Consortium,

Corresponding authors Kirk A. Rockett and Dominic P. Kwiatkowski
Wellcome Trust Centre for Human Genetics, University of Oxford, Roosevelt Drive, Oxford, UK

This file contains **Additional table ST5: Linear regression analysis of the correlations between residuals from the malaria antibody pairs.** Pairwise correlations between residuals were transformed to R-squared prior to analysis. The model was analysed as detailed in the table header with the study site included as a categorical variable orderedby the microscopically positive prevalence at each site (coeeficient = study site). Age groups were used as their categories (category [year range]_Years-old). Malaria antibody pairs are indicated in the left-hand column of the table. Kenya was not included as it only had a single age-group and Mali(Pongonon) was also not included because all individuals were selected for being malaria positive.

Additional table ST5: Linear regression analysis of the correlations between residuals from the malaria antibody pairs. Pairwise correlations between residuals were transformed to R-squared prior to analysis. The model was analysed as detailed in the table header with the study site included as a categorical variable orderedby the microscopically positive prevalence at each site (coeeficient = study site). Age groups were used as their categories (category [year range]_Years-old). Malaria antibody pairs are indicated in the left-hand column of the table. Kenya was not included as it only had a single age-group and Mali(Pongonon) was also not included because all individuals were selected for being malaria positive.

	**lm(r_squared ~ study_site [categorical] + age_group [categorical])**				
**antibody pair**	**coefficient**	Estimate	Std_Error	t-value	P-value
ama1-msp1	study Site	-0.005776	0.015137	-0.382	0.7054
	1-2_Years-old	0.102351	0.131576	0.778	0.4425
	2-5_Years-old	-0.0725	0.126701	-0.572	0.5713
	5-15_Years-old	-0.034891	0.120223	-0.29	0.7736
	15-30_Years-old	0.038485	0.120223	0.32	0.751
	>30_Years-old	0.047235	0.123492	0.382	0.7047
ama1-msp2	study Site	-0.005347	0.012123	-0.441	0.66223
	1-2_Years-old	0.08417	0.10538	0.799	0.43052
	2-5_Years-old	0.040132	0.101476	0.395	0.69519
	5-15_Years-old	-0.011408	0.096287	-0.118	0.90645
	15-30_Years-old	-0.081853	0.096287	-0.85	0.40179
	>30_Years-old	-0.098452	0.098905	-0.995	0.32724
msp1-msp2	study Site	-0.01984	0.01533	-1.294	0.205063
	1-2_Years-old	0.01074	0.13326	0.081	0.936307
	2-5_Years-old	-0.11759	0.12832	-0.916	0.366552
	5-15_Years-old	-0.03954	0.12176	-0.325	0.74755
	15-30_Years-old	-0.15428	0.12176	-1.267	0.214544
	>30_Years-old	-0.16343	0.12507	-1.307	0.200911
ama1-nanp	study Site	-0.03153	0.01664	-1.895	0.0697
	1-2_Years-old	0.16128	0.13215	1.22	0.2337
	2-5_Years-old	-0.07073	0.12637	-0.56	0.5806
	5-15_Years-old	-0.09698	0.11981	-0.809	0.4259
	15-30_Years-old	-0.09593	0.11981	-0.801	0.4309
	>30_Years-old	-0.15741	0.12337	-1.276	0.2137
msp1-nanp	study Site	-0.01268	0.01479	-0.857	0.39947
	1-2_Years-old	-0.01846	0.11751	-0.157	0.87641
	2-5_Years-old	-0.0531	0.11237	-0.473	0.64063
	5-15_Years-old	-0.06259	0.10653	-0.588	0.56211
	15-30_Years-old	-0.08205	0.10653	-0.77	0.44838
	>30_Years-old	-0.1569	0.1097	-1.43	0.16501
msp2-nanp	study Site	-0.0419	0.0158	-2.652	0.0137
	1-2_Years-old	-0.05866	0.12551	-0.467	0.6443
	2-5_Years-old	-0.10374	0.12002	-0.864	0.3956
	5-15_Years-old	-0.01242	0.11379	-0.109	0.914
	15-30_Years-old	-0.17387	0.11379	-1.528	0.1391
	>30_Years-old	-0.21811	0.11717	-1.861	0.0745
