# Supplementary material for: Genetic determinants of anti-malarial acquired immunity in a large multi-centre study
Source: Malar J. 2015 Aug 28;14:333. doi: 10.1186/s12936-015-0833-x (PMC4552443; doi:10.1186/s12936-015-0833-x)
Supplement: Additional file 12: — Additional Table ST6: Results of linear regression analysis investigating the effect of age, gender and parasite density on logged antibody levels to AMA1, MSP1, MSP2, NANP and IgE. Results of linear regression analysis investigating the effect of age, gender and parasite density using combined data for each antibody measured in this study. This is an extension to that shown in the main text and is a reduced dataset as not all sites provided parasitaemia data. [file 12936_2015_833_MOESM12_ESM.pdf]

Additional File 12: Supplementary Tables

Genetic Determinants Of Anti-Malarial Acquired Immunity In A Large Multi-Centre Study

Jennifer M.G. Shelton, Patrick Corran, Paul Risley, Nilupa Silva, Christina Hubbart, Anna Jeffreys, Kate Rowlands, Rachel Craik, Victoria Cornelius, Meike Hensmann, Sile Molloy, Nuno Sepulveda, Taane G. Clark, Gavin Band, Geraldine M. Clarke, Christopher C.A. Spencer, Angeliki Kerasidou, Susana Campino, Sarah Auburn, Adama Tall, Alioune Badara Ly, Odile Mercereau-Puijalon, Anavaj Sakuntabhai, Abdoulaye Djimde, Boubacar Maiga, Ousmane Toure, Ogobara Doumbo, Amagana Dolo, Marita Troye-Blomberg, Valentina D. Mangano, Frederica Verra, David Modiano, Edith Bougouma, Sodiomon B. Sirima, Muntaser Ibrahim, Ayman Hussain, Nahid Eid, Abier Elzein, Hiba Mohamed, Ahmed Elhassan, Ibrahim Elhassan, Thomas N. Williams, Carolyne Ndila, Alexander Macharia, Kevin Marsh, Alphaxard Manjurano, Hugh Reyburn, Martha Lemnge, Deus Ishengoma, Richard Carter, Nadira Karunaweera, Deepika Fernando, Rajika Dewasurendra, Christopher J. Drakeley, Eleanor M. Riley, Dominic P. Kwiatkowski, and Kirk A. Rockett, in collaboration with the MalariaGEN Consortium,

Corresponding authors Kirk A. Rockett and Dominic P. Kwiatkowski
Wellcome Trust Centre for Human Genetics, University of Oxford, Roosevelt Drive, Oxford, UK

This file contains **Additional Table ST6: Results of linear regression analysis investigating the effect of age, gender and parasite density* on logged antibody levels to AMA1, MSP1, MSP2, NANP and IgE.** Results shown as betas, which indicate the direction of effect of the clinical covariate on antibody levels. Beta<0 indicate a decrease in antibody levels and beta>0 indicate an increase in antibody levels. 95% confidence intervals that do not span 0 indicate an effect that is significant at p=0.05. Data from Senegal, Kenya, Sudan and Sri Lanka are not included as parasite density was not recorded at these sites.

Additional Table ST6: Results of linear regression analysis investigating the effect of age, gender and parasite density* on logged antibody levels to AMA1, MSP1, MSP2, NANP and IgE. Results shown as betas, which indicate the direction of effect of the clinical covariate on antibody levels. Beta<0 indicate a decrease in antibody levels and beta>0 indicate an increase in antibody levels. 95% confidence intervals that do not span 0 indicate an effect that is significant at p=0.05. Data from Senegal, Kenya, Sudan and Sri Lanka are not included as parasite density was not recorded at these sites.

	**anti-AMA1 levels (n=7767)**		**anti-MSP1 levels (n=7665)**		**anti-MSP2 levels (n=7727)**		**anti-NANP levels (n=2996)[a]**		**total IgE levels (n=2642)[a]**	
Factor	beta (95% CI)	p-value	beta (95% CI)	p-value	beta (95% CI)	p-value	beta (95% CI)	p-value	beta (95% CI)	p-value
Age (years)										
<1	0		0		0		0		0	
1-2	**0.04 (-0.09 to 0.16)**	**0.557**	-0.10 (-0.22 to 0.02)	0.091	**0.17 (0.06 to 0.28)**	**0.003**	0.28 (-0.02 to 0.58)	0.067	0.09 (-0.18 to 0.36)	0.506
2-5	**0.25 (0.16 to 0.35)**	**<0.001**	0.08 (-0.01 to 0.18)	0.069	**0.30 (0.21 to 0.38)**	**<0.001**	**0.50 (0.27 to 0.74)**	**<0.001**	0.09 (-0.13 to 0.31)	0.410
5-15	**0.56 (0.47 to 0.65)**	**<0.001**	**0.36 (0.27 to 0.44)**	**<0.001**	**0.60 (0.52 to 0.68)**	**<0.001**	**0.87 (0.64 to 1.10)**	**<0.001**	0.18 (-0.03 to 0.40)	0.089
15-30	**0.57 (0.48 to 0.66)**	**<0.001**	**0.65 (0.56 to 0.74)**	**<0.001**	**0.72 (0.64 to 0.81)**	**<0.001**	**1.39 (1.16 to 1.62)**	**<0.001**	**0.29 (0.07 to 0.50)**	**0.008**
>30	**0.51 (0.42 to 0.61)**	**<0.001**	**0.80 (0.71 to 0.89)**	**<0.001**	**0.70 (0.61 to 0.79)**	**<0.001**	**1.62 (1.38 to 1.86)**	**<0.001**	0.07 (-0.15 to 0.29)	0.524
Gender										
Female	0		0		0		0		0	
Male	**-0.04 (-0.07 to 0)**	**0.042**	**-0.10 (-0.14 to -0.06)**	**<0.001**	**-0.05 (-0.08 to -0.02)**	**0.002**	**-0.14 (-0.20 to -0.08)**	**<0.001**	**0.14 (0.08 to 0.19)**	**<0.001**
Parasite density (parasites/μl)										
0	0		0		0		0		0	
1-10	**0.52 (0.15 to 0.88)**	**0.005**	**0.50 (0.14 to 0.86)**	**0.007**	**0.56 (0.24 to 0.89)**	**0.001**	-0.30 (-0.70 to 0.11)	0.150	0.27 (-0.09 to 0.62)	0.143
11-100	**0.16 (0.09 to 0.24)**	**<0.001**	**0.23 (0.15 to 0.31)**	**<0.001**	**0.25 (0.18 to 0.31)**	**<0.001**	0.01 (-0.11 to 0.14)	0.828	0.04 (-0.06 to 0.14)	0.408
101-1000	**0.23 (0.17 to 0.29)**	**<0.001**	**0.18 (0.13 to 0.24)**	**<0.001**	**0.24 (0.19 to 0.30)**	**<0.001**	**-0.10 (-0.19 to -0.01)**	**0.033**	**0.09 (0.02 to 0.17)**	**0.014**
1001-10,000	**0.22 (0.14 to 0.30)**	**<0.001**	**0.12 (0.04 to 0.20)**	**0.004**	**0.20 (0.13 to 0.28)**	**<0.001**	-0.07 (-0.19 to 0.04)	0.202	0.09 (-0.01 to 0.18)	0.076
10,001-100,000	0.10 (-0.03 to 0.23)	0.135	0 (-0.013 to 0.13)	0.994	0.03 (-0.09 to 0.15)	0.632	-0.05 (-0.23 to 0.12)	0.557	0.05 (-0.09 to 0.20)	0.483
>100,000	**-0.89 (-1.66 to -0.13)**	**0.022**	0.28 (to 0.48 to 1.05)	0.465	-0.45 (-1.14 to 0.24)	0.198	-0.22 (-1.21 to 0.77)	0.662	0.43 (-0.89 to 1.76)	0.522

NB: CI = confidence interval; results significant at 0.05 level are highlighted in bold.

* Also adjusted for village (>20), ethnicity (>20), sample month (>20) and study; results not shown but ANOVA p-values were <0.001 for all antibodies.

[a] Data also not available for Tanzania (Moshi)
