# Supplementary material for: Genetic determinants of anti-malarial acquired immunity in a large multi-centre study
Source: Malar J. 2015 Aug 28;14:333. doi: 10.1186/s12936-015-0833-x (PMC4552443; doi:10.1186/s12936-015-0833-x)
Supplement: Additional file 13: — Additional Table ST7A–E: Results of site-specific linear regression analysis investigating the effect of age, gender and malaria status as determined by microscopy on antibody levels to the different antibodies measured in the study. This data shows the site-specific analyses of the various epidemiological parameters and antibody measurements. [file 12936_2015_833_MOESM13_ESM.pdf]

ADDITIONAL FILE 13: SUPPLEMENTARY TABLES

Genetic Determinants Of Anti-Malarial Acquired Immunity In A Large Multi-Centre Study

Jennifer M.G. Shelton, Patrick Corran, Paul Risley, Nilupa Silva, Christina Hubbart, Anna Jeffreys, Kate Rowlands, Rachel Craik, Victoria Cornelius, Meike Hensmann, Sile Molloy, Nuno Sepulveda, Taane G. Clark, Gavin Band, Geraldine M. Clarke, Christopher C.A. Spencer, Angeliki Kerasidou, Susana Campino, Sarah Auburn, Adama Tall, Alioune Badara Ly, Odile Mercereau-Puijalon, Anavaj Sakuntabhai, Abdoulaye Djimde, Boubacar Maiga, Ousmane Toure, Ogobara Doumbo, Amagana Dolo, Marita Troye-Blomberg, Valentina D. Mangano, Frederica Verra, David Modiano, Edith Bougouma, Sodiomon B. Sirima, Muntaser Ibrahim, Ayman Hussain, Nahid Eid, Abier Elzein, Hiba Mohamed, Ahmed Elhassan, Ibrahim Elhassan, Thomas N. Williams, Carolyne Ndila, Alexander Macharia, Kevin Marsh, Alphaxard Manjurano, Hugh Reyburn, Martha Lemnge, Deus Ishengoma, Richard Carter, Nadira Karunaweera, Deepika Fernando, Rajika Dewasurendra, Christopher J. Drakeley, Eleanor M. Riley, Dominic P. Kwiatkowski, and Kirk A. Rockett, in collaboration with the MalariaGEN Consortium,

Corresponding authors Kirk A. Rockett and Dominic P. Kwiatkowski
Wellcome Trust Centre for Human Genetics, University of Oxford, Roosevelt Drive, Oxford, UK

This file contains:

Additional Table ST7A: Results of site-specific linear regression analysis investigating the effect of age, gender and malaria status as determined by microscopy on antibody levels to AMA1. Also adjusted for village (>20), ethnicity (>20) and sample month (>20), where data were available for site.

Additional Table ST7B: Results of linear regression analysis investigating the effect of age, gender and malaria status as determined by microscopy on antibody levels to MSP1 at each site.

Additional Table ST7C: Results of site-specific linear regression analysis investigating the effect of age, gender and malaria status as determined by microscopy on antibody levels to MSP2 at each site. Also adjusted for village (>20), ethnicity (>20) and sample month (>20), where data were available for site.

Additional Table ST7D: Results of site-specific linear regression analysis investigating the effect of age, gender and malaria status as determined by microscopy on antibody levels to NANP at each site. Also adjusted for village (>20), ethnicity (>20) and sample month (>20), where data were available for site. Data not available for Tanzania (Moshi).

Additional Table ST7E: Results of site-specific linear regression analysis investigating the effect of age, gender and malaria status as determined by microscopy on total IgE levels at each site. Also adjusted for village (>20), ethnicity (>20) and sample month (>20), where data were available for site. Data not available for Tanzania (Moshi).

Additional Table ST7A: Results of site-specific linear regression analysis investigating the effect of age, gender and malaria status as determined by microscopy on antibody levels to AMA1. Also adjusted for village (>20), ethnicity (>20) and sample month (>20), where data were available for site.

	**Senegal (n=489)**		**Mali – Pongonon (n=276)**		**Mali – Manteourou (n=643)**		**Burkina Faso (n=1827)**		**Sudan (n=81)**	
Factor	beta (95% CI)[b]	p-value	beta (95% CI)[b]	p-value	beta (95% CI)[b]	p-value	beta (95% CI)[b]	p-value	beta (95% CI)[b]	p-value
Age (years)										
<1	0		-		-		0		-	
1-2	-0.02 (-0.86 to 0.83)	0.969	0		-		0.42 (0 to 0.84)	0.052	-	
2-5	0.20 (-0.55 to 0.96)	0.599	-0.40 (-1.24 to 0.44)	0.354	0		**1.19 (0.84 to 1.53)**	**<0.001**	-	
5-15	**1.11 (0.39 to 1.83)**	**0.003**	-0.25 (-1.08 to 0.59)	0.565	-0.15 (-0.38 to 0.08)	0.193	**1.48 (1.16 to 1.81)**	**<0.001**	0	
15-30	**1.66 (0.93 to 2.38)**	**<0.001**	0.03 (-1.32 to 1.38)	0.968	-0.10 (-0.35 to 0.15)	0.428	**1.16 (0.83 to 1.49)**	**<0.001**	0.15 (-0.30 to 0.61)	0.513
>30	**1.43 (0.70 to 2.16)**	**<0.001**	-0.46 (-1.51 to 0.60)	0.396	-0.19 (-0.44 to 0.05)	0.127	**0.93 (0.60 to 1.26)**	**<0.001**	0.18 (-0.37 to 0.72)	0.530
Gender										
Female	0		0		0		0		0	
Male	-0.03 (-0.18 to 0.12)	0.693	-0.23 (-0.51 to 0.04)	0.096	0.01 (-0.12 to 0.14)	0.884	**-0.12 (-0.22 to -0.03)**	**0.012**	-0.06 (-0.49 to 0.37)	0.793
Microscopy result										
Negative	-		-		0		0		-	
Positive	-		-		-0.13 (-0.28 to 0.03)	0.120	**0.13 (0.02** to **0.24)**	**0.016**	-	

	**Kenya (n=1808)**		**Tanzania - Moshi (n=6011)**		**Tanzania – SP1 (n=536)**		**Tanzania – SP2 (n=490)**		**Sri Lanka (n=792)**	
Factor	beta (95% CI)[b]	p-value	beta (95% CI)[b]	p-value	beta (95% CI)[b]	p-value	beta (95% CI)[b]	p-value	beta (95% CI)[b]	p-value
Age (years)										
<1	-		0		-		0		-	
1-2	-		0 (-0.11 to 0.10)	0.966	0		-0.13 (-0.95 to 0.69)	0.760	-	
2-5	-		**0.13 (0.04 to 0.21)**	**0.003**	0.51 (-0.54 to 1.55)	0.344	-0.12 (-0.64 to 0.39)	0.638	-	
5-15	-		**0.39 (0.31 to 0.46)**	**<0.001**	**1.19 (0.15 to 2.23)**	**0.025**	**0.67 (0.22 to 1.12)**	**0.004**	0	
15-30	-		**0.49 (0.41 to 0.57)**	**<0.001**	**1.33 (0.25 to 2.41)**	**0.016**	**0.83 (0.36 to 1.30)**	**<0.001**	-0.03 (-0.64 to 0.59)	0.932
>30	-		**0.56 (0.47 to 0.64)**	**<0.001**	-		0.37 (-0.014 to 0.88)	0.156	0.14 (-0.47 to 0.75)	0.660
Gender										
Female	0		0		0		0		0	
Male	-0.02 (-0.13 to 0.09)	0.725	0.02 (-0.02 to 0.05)	0.322	-0.06 (-0.23 to 0.12)	0.545	**-0.27 (-0.44 to -0.10)**	**0.002**	-0.04 (-0.13 to 0.05)	0.407
Microscopy result										
Negative	0		0		0		0		-	
Positive	0.02 (-0.13 to 0.17)	0.775	**0.12 (0.08 to 0.17)**	**<0.001**	0.12 (-0.10 to 0.34)	0.273	**0.39 (0.21 to 0.58)**	**<0.001**	-	

Additional Table ST7B: Results of linear regression analysis investigating the effect of age, gender and malaria status as determined by microscopy on antibody levels to MSP1 at each site.

	**Senegal (n=489)**		**Mali – Pongonon (n=277)**		**Mali – Manteourou (n=643)**		**Burkina Faso (n=1827)**		**Sudan (n=81)**	
Factor	beta (95% CI)[b]	p-value	beta (95% CI)[b]	p-value	beta (95% CI)[b]	p-value	beta (95% CI)[b]	p-value	beta (95% CI)[b]	p-value
Age (years)										
<1	0		-		-		0		-	
1-2	0.59 (-0.11 to 1.30)	0.101	0		-		-0.12 (-0.48 to 0.25)	0.528	-	
2-5	**0.66 (0.04 to 1.29)**	**0.037**	-0.01 (-0.78 to 0.76)	0.984	0		0.15 (-0.15 to 0.44)	0.333	-	
5-15	**0.85 (0.25 to 1.44)**	**0.005**	-0.01 (-0.78 to 0.76)	0.979	-0.03 (-0.33 to 0.26)	0.825	**0.47 (0.19 to 0.75)**	**0.001**	0	
15-30	**1.39 (0.79 to 1.99)**	**<0.001**	-0.32 (-1.56 to 0.92)	0.612	-0.10 (-0.42 to 0.23)	0.558	**0.56 (0.28 to 0.85)**	**<0.001**	0.13 (-0.35 to 0.61)	0.608
>30	**1.50 (0.90 to 2.10)**	**<0.001**	-0.28 (-1.29 to 0.73)	0.589	0.03 (-0.30 to 0.36)	0.863	**0.71 (0.42 to 1.00)**	**<0.001**	**0.56 (0.01 to 1.12)**	**0.049**
Gender										
Female	0		0		0		0		0	
Male	-0.15 (-0.29 to -0.01)	0.040	-0.05 (-0.31 to 0.21)	0.725	-0.06 (-0.24 to 0.11)	0.475	**-0.27 (-0.36 to -0.19)**	**<0.001**	-0.25 (-0.69 to 0.19)	0.273
Microscopy result										
Negative	-		-		0		0		-	
Positive	-		-		-0.01 (-0.22 to 0.20)	0.922	0.01 (-0.09 to 0.10)	0.882	-	

	**Kenya (n=1808)**		**Tanzania - Moshi (n=6004)**		**Tanzania – SP1 (n=536)**		**Tanzania – SP2 (n=505)**		**Sri Lanka (n=790)**	
Factor	beta (95% CI)[b]	p-value	beta (95% CI)[b]	p-value	beta (95% CI)[b]	p-value	beta (95% CI)[b]	p-value	beta (95% CI)[b]	p-value
Age (years)										
<1	-		0		0		0		-	
1-2	-		-0.12 (-0.24 to 0.01)	0.063	-0.52 (-2.17 to 1.12)	0.532	0.20 (-0.46 to 0.86)	0.553	-	
2-5	-		0.05 (-0.04 to 0.15)	0.262	-0.37 (-1.85 to 1.11)	0.626	-0.27 (-0.71 to 0.17)	0.226	-	
5-15	-		**0.34 (0.25 to 0.43)**	**<0.001**	0 (-1.48 to 1.47)	0.997	0.36 (-0.02 to 0.74)	0.061	0	
15-30	-		**0.68 (0.59 to 0.78)**	**<0.001**	0.34 (-1.15 to 1.84)	0.652	**0.71 (0.31 to 1.10)**	**<0.001**	0.07 (-0.51 to 0.64)	0.825
>30	-		**0.85 (0.76 to 0.94)**	**<0.001**	-		**0.72 (0.29 to 1.15)**	**0.001**	0.26 (-0.32 to 0.83)	0.378
Gender										
Female	0		0		0		0		0	
Male	-0.03 (-0.10 to 0.05)	0.475	-0.01 (-0.05 to 0.04)	0.776	-0.11 (-0.26 to 0.03)	0.136	**-0.35 (-0.49 to -0.21)**	**<0.001**	0.01 (-0.07 to 0.10)	0.737
Microscopy result										
Negative	0		0		0		0		-	
Positive	0.03 (-0.08 to 0.13)	0.602	**0.25 (0.19 to 0.31)**	**<0.001**	0.17 (-0.01 to 0.35)	0.059	**0.23 (0.07 to 0.39)**	**0.004**	-	

Additional Table ST7C: Results of site-specific linear regression analysis investigating the effect of age, gender and malaria status as determined by microscopy on antibody levels to MSP2 at each site. Also adjusted for village (>20), ethnicity (>20) and sample month (>20), where data were available for site.

	Senegal (n=489)		Mali – Pongonon (n=275)		Mali – Manteourou (n=643)		Burkina Faso (n=1824)		Sudan (n=81)	
Factor	beta (95% CI)[b]	p-value	beta (95% CI)[b]	p-value	beta (95% CI)[b]	p-value	beta (95% CI)[b]	p-value	beta (95% CI)[b]	p-value
Age (years)										
<1	0		-		-		0		-	
1-2	0.22 (-0.37 to 0.81)	0.464	0		-		**0.52 (0.20 to 0.84)**	**0.001**	-	
2-5	0.33 (-0.20 to 0.86)	0.221	-0.04 (-0.77 to 0.69)	0.913	0		**1.11 (0.85 to 1.37)**	**<0.001**	-	
5-15	**0.94 (0.44 to 1.44)**	**<0.001**	0.16 (-0.56 to 0.89)	0.658	-0.10 (-0.34 to 0.13)	0.386	**1.56 (1.31 to 1.80)**	**<0.001**	0	
15-30	**1.48 (0.97 to 1.99)**	**<0.001**	-0.20 (-1.37 to 0.96)	0.733	-0.10 (-0.36 to 0.15)	0.430	**1.43 (1.18 to 1.68)**	**<0.001**	0.36 (-0.02 to 0.75)	0.070
>30	**1.55 (1.04 to 2.06)**	**<0.001**	-0.14 (-1.05 to 0.77)	0.761	-0.04 (-0.30 to 0.21)	0.739	**1.34 (1.09 to 1.59)**	**<0.001**	0.35 (-0.09 to 0.80)	0.126
Gender										
Female	0		0		0		0		0	
Male	-0.02 (-0.14 to 0.10)	0.756	0.10 (-0.13 to 0.34)	0.396	0.10 (-0.03 to 0.24)	0.138	**-0.17 (-0.24 to -0.09)**	**<0.001**	-0.25 (-0.60 to 0.11)	0.180
Microscopy result										
Negative	-		-		0		0		-	
Positive	-		-		0.01 (-0.15 to 0.18)	0.867	**0.23 (0.14 to 0.31)**	**<0.001**	-	

	Kenya (n=1808)		Tanzania - Moshi (n=6026)		Tanzania – SP1 (n=533)		Tanzania – SP2 (n=491)		Sri Lanka (n=790)	
Factor	beta (95% CI)[b]	p-value	beta (95% CI)[b]	p-value	beta (95% CI)[b]	p-value	beta (95% CI)[b]	p-value	beta (95% CI)[b]	p-value
Age (years)										
<1	-		0		-		0		-	
1-2	-		**0.14 (0.03 to 0.25)**	**0.010**	0		-0.03 (-0.77 to 0.71)	0.936	-	
2-5	-		**0.19 (0.11 to 0.28)**	**<0.001**	0.05 (-0.61 to 0.70)	0.891	-0.16 (-0.63 to 0.30)	0.486	-	
5-15	-		**0.43 (0.35 to 0.51)**	**<0.001**	0.63 (-0.02 to 1.29)	0.057	**0.43 (0.02 to 0.83)**	**0.039**	0	
15-30	-		**0.61 (0.53 to 0.69)**	**<0.001**	**1.10 (0.40 to 1.81)**	**0.002**	**0.79 (0.36 to 1.21)**	**<0.001**	0.26 (-0.32 to 0.85)	0.377
>30	-		**0.66 (0.58 to 0.74)**	**<0.001**	-		**0.49 (0.03 to 0.95)**	**0.037**	0.36 (-0.23 to 0.94)	0.231
Gender										
Female	0		0		0		0		0	
Male	-0.04 (-0.13 to 0.04)	0.339	0.01 (-0.02 to 0.05)	0.427	**-0.18 (-0.33** to **-0.03)**	**0.017**	**-0.21 (-0.36 to -0.06)**	**0.006**	-0.01 (-0.09 to 0.07)	0.823
Microscopy result										
Negative	0		0		0		0		-	
Positive	**0.15 (0.03 to 0.26)**	**0.013**	**0.16 (0.11 to 0.20)**	**<0.001**	**0.29 (0.11 to 0.48)**	**0.002**	**0.37 (0.20 to 0.53)**	**<0.001**	-	

Additional Table ST7D: Results of site-specific linear regression analysis investigating the effect of age, gender and malaria status as determined by microscopy on antibody levels to NANP at each site. Also adjusted for village (>20), ethnicity (>20) and sample month (>20), where data were available for site. Data not available for Tanzania (Moshi).

	**Senegal (n=489)**		**Mali – Pongonon (n=276)**		**Mali – Manteourou (n=643)**		**Burkina Faso (n=1841)**		**Sudan (n=81)**	
Factor	beta (95% CI)[b]	p-value	beta (95% CI)[b]	p-value	beta (95% CI)[b]	p-value	beta (95% CI)[b]	p-value	beta (95% CI)[b]	p-value
Age (years)										
<1	0		-		-		0		-	
1-2	0.21 (-0.26 to 0.67)	0.379	0		-		**0.36 (0.09 to 0.62)**	**0.008**	-	
2-5	0.32 (-0.11 to 0.74)	0.143	0.13 (-0.82 to 1.07)	0.795	0		**0.60 (0.39 to 0.81)**	**<0.001**	-	
5-15	**0.80 (0.40 to 1.21)**	**<0.001**	-0.15 (-1.09 to 0.79)	0.753	0.13 (-0.07 to 0.33)	0.195	**1.02 (0.81 to 1.22)**	**<0.001**	0	
15-30	**1.06 (0.65 to 1.47)**	**<0.001**	-0.57 (-2.02 to 0.89)	0.448	**0.28 (0.06 to 0.49)**	**0.014**	**1.24 (1.03 to 1.45)**	**<0.001**	0.19 (-0.07 to 0.45)	0.165
>30	**1.35 (0.94 to 1.76)**	**<0.001**	-0.70 (-1.88 to 0.48)	0.247	**0.29 (0.07 to 0.51)**	**0.010**	**1.44 (1.23 to 1.65)**	**<0.001**	**0.50 (0.19 to 0.81)**	**0.002**
Gender										
Female	0		0		0		0		0	
Male	0.05 (-0.04 to 0.14)	0.272	-0.20 (-0.49 to 0.09)	0.177	-0.02 (-0.14 to 0.10)	0.747	**-0.10 (-0.16 to -0.03)**	**0.004**	-0.14 (-0.38 to 0.10)	0.269
Microscopy result										
Negative	-		-		0		0		-	
Positive	-		-		0.05 (-0.09 to 0.19)	0.509	0 (-0.08 to 0.07)	0.937	-	

	**Kenya (n=1808)**		**Tanzania – SP1 (n=536)**		**Tanzania – SP2 (n=494)**		**Sri Lanka (n=790)**	
Factor	beta (95% CI)[b]	p-value	beta (95% CI)[b]	p-value	beta (95% CI)[b]	p-value	beta (95% CI)[b]	p-value
Age (years)								
<1	-		-		0		-	
1-2	-		0		-0.06 (-0.57 to 0.45)	0.816	-	
2-5	-		0.13 (-0.25 to 0.51)	0.505	-0.23 (-0.56 to 0.09)	0.164	-	
5-15	-		0.37 (-0.01 to 0.74)	0.055	0.21 (-0.08 to 0.49)	0.158	0	
15-30	-		**0.70 (0.28 to 1.12)**	**0.001**	**0.51 (0.21 to 0.80)**	**0.001**	-0.01 (-0.44 to 0.42)	0.957
>30	-		-		0.24 (-0.08 to 0.57)	0.145	0.09 (-0.34 to 0.52)	0.684
Gender								
Female	0		0		0		0	
Male	0.02 (-0.03 to 0.07)	0.486	0 (-0.11 to 0.10)	0.930	-0.02 (-0.12 to 0.09)	0.736	0.05 (-0.01 to 0.11)	0.097
Microscopy result								
Negative	0		0		0		-	
Positive	0.04 (-0.03 to 0.11)	0.236	0.10 (-0.03 to 0.23)	0.149	-0.01 (-0.13 to 0.10)	0.817	-	

Additional Table ST7E: Results of site-specific linear regression analysis investigating the effect of age, gender and malaria status as determined by microscopy on total IgE levels at each site. Also adjusted for village (>20), ethnicity (>20) and sample month (>20), where data were available for site. Data not available for Tanzania (Moshi).

	**Senegal (n=489)**		**Mali – Pongonon (n=282)**		**Mali – Manteourou (n=643)**		**Burkina Faso (n=1848)**		**Sudan (n=82)**	
Factor	beta (95% CI)[b]	p-value	beta (95% CI)[b]	p-value	beta (95% CI)[b]	p-value	beta (95% CI)[b]	p-value	beta (95% CI)[b]	p-value
Age (years)										
<1	0		-		-		0		-	
1-2	-0.35 (-0.96 to 0.27)	0.268	0		-		-0.09 (-0.41 to 0.22)	0.566	-	
2-5	0.03 (-0.48 to 0.55)	0.894	-0.41 (-0.96 to 0.14)	0.145	0		0.04 (-0.23 to 0.31)	0.761	-	
5-15	0.21 (-0.28 to 0.69)	0.399	-0.49 (-1.04 to 0.05)	0.077	-0.09 (-0.26 to 0.08)	0.309	-0.02 (-0.27 to 0.24)	0.906	0	
15-30	-0.07 (-0.56 to 0.43)	0.795	-0.40 (-1.24 to 0.44)	0.348	-0.08 (-0.27 to 0.11)	0.406	0.09 (-0.16 to 0.35)	0.476	0.21 (-0.40 to 0.81)	0.507
>30	-0.10 (-0.59 to 0.39)	0.690	-0.51 (-1.19 to 0.16)	0.139	-0.06 (-0.25 to 0.13)	0.548	-0.10 (-0.36 to 0.16)	0.431	-0.39 (-1.14 to 0.36)	0.309
Gender										
Female	0		0		0		0		0	
Male	**0.15 (0.01 to 0.29)**	**0.037**	0.13 (-0.05 to 0.31)	0.150	-0.06 (-0.16 to 0.04)	0.238	**0.17 (0.10 to 0.24)**	**<0.001**	0.47 (-0.10 to 1.04)	0.110
Microscopy result										
Negative	-		-		0		0		-	
Positive	-		-		0.05 (-0.07 to 0.18)	0.383	**0.08 (0 to 0.15)**	**0.041**	-	

	**Kenya (n=1808)**		**Tanzania – SP1 (n=509)**		**Tanzania – SP2 (n=553)**		**Sri Lanka (n=793)**	
Factor	beta (95% CI)[b]	p-value	beta (95% CI)[b]	p-value	beta (95% CI)[b]	p-value	beta (95% CI)[b]	p-value
Age (years)								
<1	-		0		0		-	
1-2	-		0.34 (-0.95 to 1.64)	0.602	0.23 (-0.76 to 1.21)	0.655	-	
2-5	-		0.38 (-0.81 to 1.57)	0.529	-0.04 (-0.54 to 0.47)	0.883	-	
5-15	-		0.73 (-0.46 to 1.92)	0.230	**0.51 (0.06 to 0.96)**	**0.028**	0	
15-30	-		0.90 (-0.31 to 2.10)	0.146	**0.67 (0.20 to 1.14)**	**0.005**	-0.32 (-0.91 to 0.27)	0.290
>30	-		-		0.45 (-0.05 to 0.96)	0.078	-0.21 (-0.80 to 0.38)	0.483
Gender								
Female	0		0		0		0	
Male	0.01 (-0.06 to 0.07)	0.845	**0.13 (0.01 to 0.25)**	**0.033**	**0.10 (-0.05 to 0.26)**	**0.189**	**0.30 (0.22 to 0.39)**	**<0.001**
Microscopy result								
Negative	0		0		0		-	
Positive	0 (-0.09 to -0.09)	0.989	-0.01 (-0.16 to 0.14)	0.898	0.13 (-0.04 to 0.31)	0.136	-	
