# Supplementary material for: Genetic determinants of anti-malarial acquired immunity in a large multi-centre study
Source: Malar J. 2015 Aug 28;14:333. doi: 10.1186/s12936-015-0833-x (PMC4552443; doi:10.1186/s12936-015-0833-x)
Supplement: Additional file 15: — Additional Table ST9: Details of 196 genotyped SNPs and their derived allele frequencies at each site. This table shows allele frequencies for each SNP analysed in this study according to each study site. [file 12936_2015_833_MOESM15_ESM.pdf]

ADDITIONAL FILE 15: SUPPLEMENTARY TABLES

Genetic Determinants Of Anti-Malarial Acquired Immunity In A Large Multi-Centre Study

Jennifer M.G. Shelton, Patrick Corran, Paul Risley, Nilupa Silva, Christina Hubbart, Anna Jeffreys, Kate Rowlands, Rachel Craik, Victoria Cornelius, Meike Hensmann, Sile Molloy, Nuno Sepulveda, Taane G. Clark, Gavin Band, Geraldine M. Clarke, Christopher C.A. Spencer, Angeliki Kerasidou, Susana Campino, Sarah Auburn, Adama Tall, Alioune Badara Ly, Odile Mercereau-Puijalon, Anavaj Sakuntabhai, Abdoulaye Djimde, Boubacar Maiga, Ousmane Toure, Ogobara Doumbo, Amagana Dolo, Marita Troye-Blomberg, Valentina D. Mangano, Frederica Verra, David Modiano, Edith Bougouma, Sodiomon B. Sirima, Muntaser Ibrahim, Ayman Hussain, Nahid Eid, Abier Elzein, Hiba Mohamed, Ahmed Elhassan, Ibrahim Elhassan, Thomas N. Williams, Carolyne Ndila, Alexander Macharia, Kevin Marsh, Alphaxard Manjurano, Hugh Reyburn, Martha Lemnge, Deus Ishengoma, Richard Carter, Nadira Karunaweera, Deepika Fernando, Rajika Dewasurendra, Christopher J. Drakeley, Eleanor M. Riley, Dominic P. Kwiatkowski, and Kirk A. Rockett, in collaboration with the MalariaGEN Consortium,

Corresponding authors Kirk A. Rockett and Dominic P. Kwiatkowski
Wellcome Trust Centre for Human Genetics, University of Oxford, Roosevelt Drive, Oxford, UK

This file contains **Additional Table ST9: Details of 196 genotyped SNPs and their derived allele frequencies at each site.** SNPs are shown in chromosome order. SNPs not shown if they were gender-determining (n=3) or had an assay pass rate <80% (n=3).
SE: Senegal; DP Mali (Pongonon) and DQ Mali (Manteourou); BJ Burkina Faso; SA Sudan; KM; Kenya; TN Tanzanaia (Moshi); TG Tanga (SP2); TP Tanga (SP1) and SL Sri Lanka.

Additional Table ST9: Details of 196 genotyped SNPs and their derived allele frequencies at each site. SNPs are shown in chromosome order. SNPs not shown if they were gender-determining (n=3) or had an assay pass rate <80% (n=3).
SE: Senegal; DP Mali (Pongonon) and DQ Mali (Manteourou); BJ Burkina Faso; SA Sudan; KM; Kenya; TN Tanzanaia (Moshi); TG Tanga (SP2); TP Tanga (SP1) and SL Sri Lanka.

				West Africa				East Africa					Asia
**rs number**	**gene**	**chr**	**coordinate**	**SE**	**DP**	**DQ**	**BJ**	**SA**	**KM**	**TN**	**TG**	**TP**	**SL**
rs1803632	GBP7	1	89582690	56.54	51.29	54.6	53.44	40.48	52.02	50.79	50.55	52.66	64.74
rs2814778	DARC	1	159174683	100	100	100	99.71	100	99.97	99.09	99	99.68	0
rs2251746	FCER1A	1	159272060	5.93	3.71	8.64	5.1	2.38	11.85	10.33	9.85	8.94	26.26
rs1801274	FCGR2a	1	161479745	43.08	60.59	59.11	63.41	48.63	60.23	56.54	60.14	57.22	44.1
rs10127939	CD16	1	161518333	9.51	11.53	11.8	6.1	5.13	4.73	3.64	4.27	3.19	3.85
rs3024500	IL10	1	206940831	33.9	38.94	33.23	36.98	51.85	52.63	48.13	47.52	48.56	20.89
rs1518110	IL10	1	206944861	54.08	50.33	52.73	50.98	36.49	34.55	38.03	41.21	37.56	47.54
rs1800872	IL10	1	206946407	54.41	50.32	53.04	50.98	37.5	34.62	38.12	41.67	37.6	51.32
rs1800871	IL10	1	206946634	54.17	50.33	52.96	51.01	38.31	34.45	37.96	41.55	37.56	51.26
rs1800896	IL10	1	206946897	30.75	30.45	29.78	30.66	42.26	36.41	33.58	32.34	34.43	19.82
rs1800890	IL10	1	206949365	20.02	21.63	16.64	17.03	22.62	23.66	22.77	23.32	22.15	15.54
rs17047660	CR1	1	207782856	41.83	34.95	35.96	32.75	40.96	16.44	24.76	20.49	21.89	0
rs17047661	CR1	1	207782889	79.33	73.06	69.3	73.01	76.19	67.26	59.15	62.36	62.46	0.19
rs17561	IL1A	2	113537223	18.15	21.63	26.98	17.03	17.86	17.7	18.74	18.3	20.1	31.41
rs1800587	IL1A	2	113542960	41.73	51.8	50.24	42.95	35.53	37.21	39.42	37.55	41.6	29.69
rs1143634	IL1B	2	113590390	11.52	12.7	19.3	11.44	12.5	12.67	14.65	9.51	12.94	13.19
rs708567	IL17RE	3	9960070	41.77	55.14	50.71	45.91	48.72	48.4	50.84	49.9	50.75	31.58
rs352140	TLR9	3	52231737	77.32	78.46	70.56	73.32	69.05	66.05	68.73	66.45	68.39	61.44
rs187084	TLR9	3	52261031	73.39	73.4	73.56	71.92	71.6	66.53	71.41	68.36	68.06	66.86
rs6780995	IL17RD	3	57138419	59.21	57.53	60.88	57.87	56.55	58.03	56.61	56.96	57.06	75.82
rs4833095	TLR1	4	38799710	12.9	11.15	11.15	9.98	8.43	9.39	7.96	9.48	9.89	49.56
rs5743611	TLR1	4	38800214	0	0	0	0	0	0	0	0	0	0.06
rs5743810	TLR6	4	38830350	0.5	0.97	4.25	1.45	0	1.74	3.12	1.09	2.18	1
rs5743809	TLR6	4	38830514	1.71	4.49	5.46	5.97	7.74	4.9	9.73	7.92	5.5	0
rs4073	IL8	4	74606024	14.85	8.77	11.97	15.14	6.25	14.15	17.48	16.03	16.42	61.42
rs6897932	IL7R	5	35874575	9.51	10.65	8.52	7.61	6.55	5	6.28	6.11	6.97	20.82
rs3194051	IL7R	5	35876274	31.02	33.06	29.83	31.67	28.26	25.7	27.3	28.52	24.36	19.68
rs1801033	C6	5	41199959	36.39	41.16	42.76	43.91	42.26	47.9	45.77	46.66	46.77	35.29
rs2289276	TSLP	5	110407507	15.89	11.85	18.07	14.84	12.99	8.75	13.07	12.45	12.74	39.62
rs3091336	IL3	5	131391749	70.18	65.11	73.4	72.92	68.45	77.14	79.51	75.59	81.02	100
rs35415145	IL3	5	131396406	2.04	2.27	2.44	0.75	3.7	3.38	2.18	4.11	1.54	0.06
rs40401	IL3	5	131396478	57.37	40.97	57.4	52.48	43.45	58.01	56.78	51.74	53.39	62.67
rs35482671	IL3	5	131396676	0	0	0	0	0	0	0	0	0	0
rs13166954	IL3	5	131396709	0	0	0	0	0	0	0.01	0	0	0
rs31481	IL3	5	131397202	11.54	13.62	7.78	11.83	20.63	15.99	18.8	19.95	20.19	36.32
rs168681	IL3	5	131402450	18.39	17.49	17.85	17.51	24.66	22.5	27.58	28.44	26.1	64.37
rs2069614	CSF2	5	131407601	41.97	43.55	50.16	45.68	62.2	54.93	55.14	55.57	55.08	92.6
rs10072253	CSF2	5	131409768	0	0	0	0	0	0	0.01	0	0	0
rs2069640	CSF2	5	131411454	0	0	0	0	0	0	0	0	0	0
rs25882	CSF2	5	131411460	30.43	35.99	25.51	28.79	37.2	31.81	29.15	27.57	26.81	34.22
rs25887	CSF2	5	131416061	48.36	45.97	51.25	49.36	42.77	49.44	44.69	45.35	47.81	36.91
rs3805685	P4HA2	5	131528153	9.13	11.54	4.12	8.1	8.02	6.24	7.58	8.71	7.61	2.01
rs156029	P4HA2	5	131532634	46.25	49.84	54.75	50.21	48.21	43.8	44.65	43.34	46.22	33.94
rs159903	P4HA2	5	131540053	22.47	27.17	25	23.03	29.11	19.1	18.13	18.6	16.88	19.3
rs11955347	P4HA2	5	131567924	26.67	26.6	35.02	25.37	31.25	24.75	35.02	27.19	33.23	7.14
rs3900945	PDLIM4	5	131592870	37.97	42.72	33.54	39.69	25.9	34.73	28.88	33.62	27.93	74.13
rs10463891	PDLIM4	5	131597392	38.53	42.23	33.64	39.85	25.6	34.93	29.05	33.95	27.47	74.02
rs17851430	PDLIM4	5	131606632	0	0	0	0	0	0	0	0	0	0
rs156112	PDLIM4	5	131610232	18.65	26.76	11.54	16.3	15.79	17.35	13.25	14.9	11.81	4.75
rs398064	SLC22A4	5	131647902	0	0	0	0	0	0	0	0	0	0
rs11568510	SLC22A4	5	131647954	0	0	0	0	0	0	0	0	0	0
rs11568499	SLC22A4	5	131649300	0	0	0	0	0	0	0	0	0	0
rs455649	SLC22A4	5	131649306	0	0	0	0	0	0	0.01	0	0	0
rs272893	SLC22A4	5	131663062	77.89	77.24	71.77	78.33	82.89	71.89	72.86	69.05	73.1	35.55
rs2304081	SLC22A4	5	131667548	3.85	2.44	7.94	4.05	3.16	4.7	5.51	4.64	5.82	2.89
rs4646201	SLC22A4	5	131671634	0.1	0	0	0	0	0	0	0	0	0
rs272867	SLC22A4	5	131681057	71.18	62.1	63.44	69.87	76.79	67.94	67.22	64.53	65.24	34.62
rs11544587	SLC22A5	5	131706005	0	0	0	0	0	0	0	0	0	0
rs10040427	SLC22A5	5	131714106	10.98	9.63	5.01	8.93	3.62	9.33	8.28	9.98	9.18	0
rs4551059	SLC22A5	5	131719999	0	0	0	0	0	0	0	0	0	0
rs11568525	SLC22A5	5	131729935	6.46	10.97	7.72	6.37	2.38	2.75	3.41	2.64	3.95	0
rs7704457	LOC441108	5	131744790	14.15	14.95	16.24	18.08	19.88	23.43	15.24	18.41	12.89	38.91
rs6874639	LOC441108	5	131778716	31.64	34.41	33.23	34.13	25.31	23.45	21.04	22.5	20.89	39.46

rs2522051	LOC441108	5	131797578	68.35	59.55	67.71	59.59	63.97	57.46	57.34	61.1	56.81	14.02
rs2706379	LOC441108	5	131805735	22.82	33.17	27.49	30.45	26.19	24.86	25.95	23.63	21.85	37.91
rs2706381	LOC441108	5	131810619	22.47	31.03	25.9	29.45	26.19	24.79	24.91	23.59	22.4	37.69
rs2070729	IRF1	5	131819921	29.94	29.84	37.75	34.89	22.02	26.46	25.46	26.87	26.17	32.91
rs2070724	IRF1	5	131822072	37.32	43.23	52.95	52.81	45.06	39.83	41.35	40.64	40.44	57.35
rs2070722	IRF1	5	131824486	37.34	43.54	53.02	53.14	48.33	39.59	40.63	40.2	39.51	57.29
rs2706384	IRF1	5	131826880	33.23	37.9	49.53	47.4	46.25	36.81	39.22	38.1	37.77	57.99
rs2548999	IRF1	5	131831058	34.69	39.55	46.26	44.81	44.51	38.53	39.96	37.34	38.69	58.71
rs739718	IL5	5	131873073	23.59	28.55	25.82	29.26	29.23	29.75	33.02	32.04	34.03	2.3
rs2069818	IL5	5	131877524	8.98	8.68	11.14	10.18	5.95	5.25	5.43	6.02	6.7	0
rs2069823	IL5	5	131877699	0	0	0	0	0	0	0	0	0	0
rs4526098	RAD50	5	131892979	76.47	67.68	84.37	79.13	75.31	73.98	75.26	75.45	80.84	98.49
rs2706348	RAD50	5	131905810	33.92	35.94	50.83	46.49	40.41	31.54	37.83	38.32	37.42	80.09
rs28903086	RAD50	5	131915022	0	0	0	0	1.35	0	0.01	0	0	0
rs28903088	RAD50	5	131915673	0	0	0	0	0.68	0	0.01	0	0	0
rs10479007	RAD50	5	131917726	22.87	15.74	11.29	17.71	19.18	14.21	11.54	13.7	12.16	0
rs28903090	RAD50	5	131923673	0	0	0	0	0	0	0	0	0	0
rs1047380	RAD50	5	131930613	0	0	0	0	0	0	0	0	0	0
rs1047382	RAD50	5	131931385	0	0	0	0	0	0	0	0	0	0
rs28903092	RAD50	5	131931472	0	0	0	0	0	1.54	0.31	0.45	0.24	0
rs28903093	RAD50	5	131940498	0	0	0	0	0	0	0	0	0	0
rs1047386	RAD50	5	131944869	0	0	0	0	0	0	0	0	0	0
rs1047387	RAD50	5	131951770	0	0	0	0	0	0	0	0	0	0
rs17772565	RAD50	5	131952405	9.6	15.22	17.88	15.16	9.88	9.62	11.75	9.71	11.15	7.8
rs3798134	RAD50	5	131965179	44.57	34.19	35.51	35.42	34	44.15	38.4	39.85	46.39	19.29
rs35861031	RAD50	5	131976461	0	0	0	0	0	0	0	0	0	0
rs1804669	RAD50	5	131978166	0	0	0	0	0	0	0	0	0	0
rs4621555	RAD50	5	131982808	76.21	86.82	89.88	84.41	84.34	89.79	92.57	90.46	93.45	100
rs1881457	IL13	5	131992409	20.94	16.67	17.27	15.5	10.87	27.18	23.4	22.65	26.34	18.37
rs2069744	IL13	5	131994669	42.47	38.26	19.39	29.2	41.57	30.22	33.35	34.96	36.35	0.34
rs20541	IL13	5	131995964	13.97	18.67	18	13.89	16.46	20.57	20.11	18.51	21.63	27.8
rs848	IL13	5	131996500	41.55	48.88	62.79	52.41	40	49.83	49.83	48.27	47.15	74.09
rs2243206	IL13	5	132001065	19.96	14.84	15.1	17.33	27.56	13.15	8.93	10.55	8.33	0
rs2243248	IL4	5	132008644	10.16	13.44	14.91	14.3	18.84	23.95	21.85	18.8	25.21	10.47
rs2243250	IL4	5	132009154	74.09	77.78	64.08	74.17	75.6	78.72	72.09	76.72	71.51	17.76
rs2243251	IL4	5	132009787	17.89	19.06	16.74	17.11	6.72	21.94	17.03	18.78	21.17	0
rs4986964	IL4	5	132009821	0	0	0	0	0	0.03	0	0	0	0
rs2243270	IL4	5	132014109	31.34	23.79	30.73	24.75	21.95	26.38	32.22	27.41	33.58	80.05
rs2243283	IL4	5	132016593	24.95	21.45	19.17	21.91	30.25	16.99	19.39	21.44	18.04	0.63
rs3212227	IL12B	5	158742950	31.05	43.39	32.6	35.11	26.09	39.14	40.2	35.74	40.6	38.59
rs919766	IL12B	5	158747564	29.82	19.23	23.72	27.79	29.38	27.9	20.95	23.14	21.96	6.27
rs3181216	IL12B	5	158752978	19.33	16.99	18.25	16.83	10.63	11.43	15.16	17.61	16.64	33.25
rs730691	IL12B	5	158756227	41.28	26.77	39.42	37.6	44.44	30.94	38.86	40.55	38	54.38
rs2239704	LTA	6	31540141	43.76	40.91	38.79	35.05	17.26	11.19	15.45	16.3	16.02	37.89
rs909253	LTA	6	31540313	37.55	37.02	38.69	37.72	53.57	48.78	54.96	55.72	53.95	29.02
rs1799964	TNF	6	31542308	12.8	15.54	17.13	18.47	22.02	27.93	23.77	21.14	23.75	32.62
rs1800750	TNF	6	31542963	1.11	1.21	2.8	3.11	2.38	4.83	4.28	2.49	3.54	0.5
rs1800629	TNF	6	31543031	17.2	11.86	8.16	12.7	5.95	8.87	8.99	9.87	9.39	9.85
rs361525	TNF	6	31543101	5.84	2.24	5.21	5.4	4.17	6.78	10.11	6.34	8.27	8.52
rs3093662	TNF	6	31544189	9.86	3.37	7.82	10.55	7.83	11.63	11.93	8.61	9.41	9.3
rs2242665	CTL4	6	31839309	66.63	65.53	71.14	72.68	69.05	74.82	78.77	77.59	79.85	51.13
rs1555498	IL20RA	6	137325847	53.84	34.78	45.55	43.56	50	49.03	43.89	50.45	45.18	99.94
rs11575936	IFNGR1	6	137540425	0	0	0	0	0.68	0	0	0	0	0
rs2075820	NOD1	7	30492237	44.14	39.71	38.23	36.85	43.45	38.52	33.91	32.08	34.59	41.85
rs9942705	GLI3	7	42445655	21.88	25	26	26.22	28.31	20.58	27.38	26.68	26.16	7.01
rs17172181	HECW	7	43283115	51.01	60.98	75	59.86	46.99	51.68	46.27	52.31	51.99	70.22
rs17172184	HECW	7	43286129	37.73	47.85	56.53	50.37	41.46	35.06	35.4	39.28	40.02	60.71
rs7779749	IZKF1	7	50418506	73.82	74.55	81.74	69.72	54.88	54.86	59.35	61.32	62.03	76.63
rs10230385	IZKF1	7	50452552	47.27	45.98	44.28	41.39	38.41	36.08	40.44	39.01	42.22	33.4
rs6964823	IZKF1	7	50460096	42.83	43.25	43.17	41.32	40.48	43.77	41.13	41.59	39.11	42.83
rs4132601	IZKF1	7	50470604	15.76	13.53	14.74	17.64	30.12	18.32	19.67	18.47	19.58	23.14
rs11980407	IZKF1	7	50471613	84.01	85	80.45	82.37	72.89	81.69	80.67	81.78	82.03	76.9
rs6944602	IZKF1	7	50473751	3.74	2.93	2.08	3.4	1.2	8.08	8.43	7.52	7.1	14
rs4947535	DDC	7	50531681	46.82	51.76	50.09	45.18	38.69	38.8	40.35	40.93	40.43	34.27
rs11983581	DDC	7	50532888	87.58	83.76	85.3	84.35	75.63	82.3	84.95	86.93	87.93	98.01
rs11982772	DDC	7	50533062	12.7	16.03	14.67	15.58	23.81	17.72	15.16	13.11	12.06	2.15
rs11575527	DDC	7	50534327	12.7	16.34	14.61	15.91	23.49	17.71	15.26	13.07	13.17	2.17
rs11575522	DDC	7	50535395	12.31	15.75	14.47	15.57	23.46	17.8	15.02	13.18	12.42	2.1
rs11575518	DDC	7	50535681	87.22	83.82	85.5	84.24	76.83	82.28	84.84	86.95	87.32	97.85
rs11575483	DDC	7	50544663	0.3	2.14	0.73	4.31	2.41	4.01	2.84	2.31	2.69	0
rs11575387	DDC	7	50567435	22.41	22.42	13.41	16.85	28.31	25.28	20.51	19.32	19	7.4

rs3779084	DDC	7	50568735	22.36	21.85	11.87	16.52	29.01	26.2	20.55	18.87	19.61	13.94
rs880028	DDC	7	50570136	80.97	84.83	92.29	85.7	68.67	76.72	80.47	81.95	79.13	86.1
rs6592961	DDC	7	50572890	70.39	66.23	74.55	68.45	53.61	55.44	62.68	62.91	61.27	81.55
rs7809758	DDC	7	50573333	70.45	69.06	75.28	72.25	55.56	58.34	64.86	65.19	63.33	70.08
rs1817074	DDC	7	50574012	68.7	65.53	73.46	67.57	50	54.18	61.85	61.97	60.14	68.05
rs10271341	DDC	7	50591583	24.79	37.46	35.27	31.63	15.85	14.27	14.33	13.59	13.63	29.01
rs11575320	DDC	7	50605298	24	36.07	33.49	31.51	13.75	14.06	13.3	13.14	12.56	23.96
rs3779074	DDC	7	50612202	42.52	59.62	46.93	51.99	44.05	43.61	42.94	42.03	40.82	42.41
rs2044859	DDC	7	50612562	42.46	59.48	47.32	52.06	43.9	43.62	43.15	41.96	41.21	42.98
rs2329371	DDC	7	50615440	24.56	35.64	33.62	29.42	15.24	13.49	13.35	12.78	13.04	34.96
rs6956737	DDC	7	50620781	10.77	11.73	5.19	13.55	16.87	17.97	18.4	17.9	16.69	2.23
rs1451375	DDC	7	50622712	14.99	20.17	11.31	24.39	14.63	17.88	16	16.08	15.24	39.73
rs10249420	DDC	7	50623451	13.94	20.42	14.66	22.52	12.05	13.05	12.88	12.22	12.27	28.76
rs7803788	DDC	7	50625898	13.62	18.17	9.39	23.09	11.11	14.54	13.13	12.96	13.01	27.92
rs6593010	DDC	7	50629888	72.1	56.63	63.34	62.82	63.25	66.27	64.25	67.67	64.01	57.87
rs7800827	DDC	7	50673171	18.28	8.06	9.96	7.89	9.04	9.2	9.87	8.47	6.55	0
rs3211938	CD36	7	80300449	0.7	61.93	36.63	16.5	9.64	9.76	2.69	8.33	5.63	0
rs201346212	CD36	7	80302110	0.6	1.29	0.16	2.02	0	0	0	0	0	0
rs17140229	CFTR	7	117230283	35.05	37.22	34.46	38.26	30.36	33.62	25.98	28.18	30.13	0
rs4986790	TLR4	9	120475302	7.39	9.42	6.22	8.5	14.2	4.26	7.9	4.55	5	16.37
rs4986791	TLR4	9	120475602	0.81	2.24	0.54	0.88	1.79	0.55	0.75	0.54	0.56	14.2
rs8176746	ABO	9	136131322	16.83	20.85	18.21	21.09	20.24	14.45	14.03	11.26	11.53	16.88
rs8176719	ABO	9	136132909	67.2	67.63	68.2	64.48	69.05	73.11	72.55	73.95	75.88	67.09
rs33950507	HBB	11	5248173	0	0	0	0	0	0	0	0	0	0
rs334	HBB	11	5248232	4.33	3.53	1.48	4.55	7.74	7.3	1.64	7.07	5.65	0.44
rs33930165	HBB	11	5248233	0.71	9.18	2.03	10.16	0	0.03	0	0	0	0
rs7935564	TRIM5	11	5718517	48.99	43.91	49.6	53.59	61.88	44.36	46.49	45.28	46.24	51.31
rs569108	MS4A2	11	59863104	15.11	19.29	17.47	20.07	23.49	21.7	23.75	27.87	26.58	6.12
rs542998	RTN3	11	63487386	42.14	32.74	45.36	45.65	36.14	46.15	38	37.22	38.51	70.47
rs3024978	STAT6	12	57490378	0	0	0	0	0	0.42	0.39	1.27	0.4	0
rs12314983	STAT6	12	57492308	4.46	1.93	2.11	2.39	1.23	0.31	0.56	0.55	0.4	0
rs35182390	STAT6	12	57493602	0	0	0	0	0.66	0	0	0	0	0.13
rs3024952	STAT6	12	57500112	0	0	0	0	0	0.19	0.49	0.18	0	0
rs2626577	STAT6	12	57500509	0	0	0	0.03	0	0	0	0	0	0
rs2069727	IFNG	12	68548223	9.94	15.58	19.45	18.41	11.04	18.07	20.07	19.06	23.18	39
rs2234687	IFNG	12	68548756	1.01	1.14	0.55	0.66	0	0.72	0.07	0	0.08	0
rs2069718	IFNG	12	68550162	24.59	35.31	37.46	42.13	38.14	38.67	41.14	41.93	44.17	43.33
rs1861493	IFNG	12	68551196	7.84	7.19	6.64	7.06	6.16	6.85	6.22	7.62	6.5	13.32
rs2069705	IFNG	12	68555011	42.42	52.31	52.34	59.22	69.44	58.86	58.28	57.36	58.82	70.44
rs2227507	IL22	12	68642647	3.82	1.77	1.01	2.69	4.17	4.59	3.31	3.45	3.95	0.06
rs1012356	IL22	12	68644618	52.74	55.02	46.57	47.06	43.45	45.18	48.82	47.01	48.22	34.48
rs2227491	IL22	12	68646521	72.68	70.74	60.82	63.93	60.24	56.31	58.63	56.2	58.35	43.07
rs2227485	IL22	12	68647713	52.02	53.23	44.87	45.36	38.1	40.01	40.75	38.72	39.07	34.23
rs2227478	IL22	12	68648622	73.89	73.95	62.77	61.62	59.52	59.11	59.84	57.08	56.76	60.92
rs229587	SPTB	14	65263300	22.41	35.42	36.83	37.07	40.96	33.63	36.64	34.77	32.07	53.32
rs2230739	ADCY9	16	4033436	23.59	13.02	20.86	18.94	13.86	8.55	8.02	10.33	8.13	26.19
rs10775349	ADCY9	16	4079823	22.17	14.1	27.15	20.75	21.43	21.01	26.36	23.71	23.09	91.65
rs1805015	IL4R	16	27374180	50.61	50	35.34	44.37	35.54	42.86	41.54	37.61	37.68	11.82
rs5470	HP	16	72088421	18.43	16.45	17.37	16.22	8	13.84	8.71	9.69	8.59	0
rs2535611	ADORA2B	17	15861332	0.91	6.15	5.45	6.15	9.04	11.21	11.93	10.02	12.76	1.38
rs2297518	NOS2	17	26096597	13.46	7.56	14.9	12.3	9.04	13.89	17.26	18.27	16.77	13.95
rs1800482	NOS2	17	26128509	9.56	7.53	7.77	9.48	5.36	7.67	4.51	5.9	5.33	0
rs2779249	NOS2A	17	26128581	49.39	49.03	58.74	48.65	48.1	39.34	38.24	37.27	38.49	22.96
rs9282799	NOS2	17	26128728	5.53	11.09	5.37	6.49	8.93	2.74	4.23	4.35	4.58	0
rs8078340	NOS2	17	26129212	27.27	26.94	34.62	28.43	24.7	22.17	21.28	20.38	21.35	8.98
rs373533	EMR1	19	6919624	48.47	46.39	36.49	43.43	49.4	49.83	46.16	48.41	50.73	29.06
rs461645	EMR1	19	6919753	52.35	55.93	63.24	56.47	50	49.7	53.29	51.46	49.03	69.64
rs35825847	FCER2	19	7754284	1.12	1.12	0.78	0.05	0	0	0	0	0	0
rs1799969	ICAM1	19	10394792	0.1	0	0.39	1.01	0	0	0.03	0.27	0	2.26
rs5498	ICAM1	19	10395683	11.49	15.48	19.42	14.82	6.55	13.58	10.93	12.45	12.83	50.45
rs8386	GNAS	20	57485812	13.39	14.74	14.95	19.25	21.43	18.24	15.23	15.67	15.43	0.25
rs1128127	DERL3	22	24179132	61.38	48.54	49.36	52.11	64.46	45.59	50.04	47.31	47.68	35.63
