# Supplementary material for: Genetic determinants of anti-malarial acquired immunity in a large multi-centre study
Source: Malar J. 2015 Aug 28;14:333. doi: 10.1186/s12936-015-0833-x (PMC4552443; doi:10.1186/s12936-015-0833-x)
Supplement: Additional file 16: — Additional Table ST10: Meta-analysis p-values obtained for 178 SNPs passing QC criteria detailed in materials and methods. Details of the analyses for each SNP and antibody across all sites for different genetic inheritance models. [file 12936_2015_833_MOESM16_ESM.pdf]

## ADDITIONAL FILE 16: SUPPLEMENTARY TABLES

# Genetic Determinants Of Anti-Malarial Acquired Immunity In A Large Multi-Centre Study

Jennifer M.G. Shelton, Patrick Corran, Paul Risley, Nilupa Silva, Christina Hubbart, Anna Jeffreys, Kate Rowlands, Rachel Craik, Victoria Cornelius, Meike Hensmann, Sile Molloy, Nuno Sepulveda, Taane G. Clark, Gavin Band, Geraldine M. Clarke, Christopher C.A. Spencer, Angeliki Kerasidou, Susana Campino, Sarah Auburn, Adama Tall, Alioune Badara Ly, Odile Mercereau-Puijalon, Anavaj Sakuntabhai, Abdoulaye Djimde, Boubacar Maiga, Ousmane Toure, Ogobara Doumbo, Amagana Dolo, Marita Troye-Blomberg, Valentina D. Mangano, Frederica Verra, David Modiano, Edith Bougouma, Sodiomon B. Sirima, Muntaser Ibrahim, Ayman Hussain, Nahid Eid, Abier Elzein, Hiba Mohamed, Ahmed Elhassan, Ibrahim Elhassan, Thomas N. Williams, Carolyne Ndila, Alexander Macharia, Kevin Marsh, Alphaxard Manjurano, Hugh Reyburn, Martha Lemnge, Deus Ishengoma, Richard Carter, Nadira Karunaweera, Deepika Fernando, Rajika Dewasurendra, Christopher J. Drakeley, Eleanor M. Riley, Dominic P. Kwiatkowski, and Kirk A. Rockett, in collaboration with the MalariaGEN Consortium,

Corresponding authors Kirk A. Rockett and Dominic P. Kwiatkowski
Wellcome Trust Centre for Human Genetics, University of Oxford, Roosevelt Drive, Oxford, UK

This file contains **Additional Table ST10: Meta-analysis p-values obtained for 178 SNPs passing QC criteria detailed in materials and methods.** For each SNP-antibody association the genetic model that gave the lowest meta analysis p-value is given. NAs were generated if the MAF was <1% or the SNP was monomorphic at the majority of sites.

**Additional Table ST10: Meta-analysis p-values obtained for 178 SNPs passing QC criteria detailed in materials and methods.** For each SNP-antibody association the genetic model that gave the lowest meta analysis p-value is given. NAs were generated if the MAF was <1% or the SNP was monomorphic at the majority of sites.

| rsnumber | gene | chr | coordinate | anti-AMA1 | | | | anti-MSP1 | | | | anti-MSP2 | | | | anti-(NANP)$_4$ | | | |
|---|---|---|---|---|---|---|---|---|---|---|---|---|---|---|---|---|---|---|---|
| | | | | model | beta | 95%CIs | p-value | model | beta | 95%CIs | p-value | model | beta | 95%CIs | p-value | model | beta | 95%CIs | p-value |
| rs1803632 | GBP7 | 1 | 89582690 | recessive | -0.02 | (-0.05 to 0.01) | 2.03E-01 | additive | 0.01 | (-0.01 to 0.03) | 5.57E-01 | dominant | 0.01 | (-0.02 to 0.04) | 3.88E-01 | dominant | 0.02 | (-0.02 to 0.06) | 2.51E-01 |
| rs2814778 | DARC | 1 | 159174683 | NA | NA | NA | NA | NA | NA | NA | NA | NA | NA | NA | NA | NA | NA | NA | NA |
| rs2251746 | FCER1A | 1 | 159272060 | hetero | 0.01 | (-0.03 to 0.04) | 6.62E-01 | additive | 0.03 | (0 to 0.06) | 6.72E-02 | additive | -0.02 | (-0.05 to 0.01) | 2.45E-01 | recessive | 0.11 | (0 to 0.23) | 5.25E-02 |
| rs1801274 | FCGR2A | 1 | 161479745 | additive | -0.01 | (-0.03 to 0) | 1.25E-01 | dominant | -0.04 | (-0.07 to 0) | 3.58E-02 | additive | -0.02 | (-0.04 to 0) | 3.36E-02 | hetero | 0.01 | (-0.02 to 0.04) | 5.99E-01 |
| rs10127939 | CD16 | 1 | 161518333 | recessive | 0.64 | (-0.57 to 1.85) | 2.98E-01 | hetero | -0.03 | (-0.08 to 0.02) | 3.01E-01 | hetero | -0.02 | (-0.07 to 0.02) | 3.20E-01 | recessive | 0.37 | (-0.58 to 1.31) | 4.44E-01 |
| rs3024500 | IL10 | 1 | 206940831 | hetero | 0 | (-0.02 to 0.03) | 8.66E-01 | additive | 0 | (-0.02 to 0.02) | 7.92E-01 | hetero | 0.02 | (0 to 0.05) | 6.49E-02 | recessive | -0.03 | (-0.07 to 0.01) | 1.87E-01 |
| rs1518110 | IL10 | 1 | 206944861 | additive | 0.01 | (-0.01 to 0.02) | 5.92E-01 | dominant | 0.01 | (-0.02 to 0.04) | 5.21E-01 | hetero | 0.02 | (0 to 0.05) | 9.41E-02 | hetero | 0.02 | (-0.01 to 0.05) | 2.32E-01 |
| rs1800872 | IL10 | 1 | 206946407 | additive | 0.01 | (-0.01 to 0.03) | 5.35E-01 | dominant | 0.01 | (-0.02 to 0.04) | 5.48E-01 | hetero | 0.02 | (0 to 0.05) | 8.37E-02 | hetero | 0.02 | (-0.01 to 0.06) | 1.33E-01 |
| rs1800871 | IL10 | 1 | 206946634 | recessive | 0.01 | (-0.03 to 0.05) | 5.94E-01 | dominant | 0.01 | (-0.02 to 0.04) | 5.44E-01 | hetero | 0.02 | (0 to 0.05) | 1.01E-01 | hetero | 0.02 | (-0.01 to 0.06) | 1.40E-01 |
| rs1800896 | IL10 | 1 | 206946897 | hetero | -0.02 | (-0.05 to 0) | 7.58E-02 | hetero | -0.01 | (-0.03 to 0.02) | 6.57E-01 | hetero | 0.01 | (-0.02 to 0.03) | 5.99E-01 | hetero | 0.01 | (-0.02 to 0.04) | 5.38E-01 |
| rs1800890 | IL10 | 1 | 206949365 | hetero | -0.02 | (-0.04 to 0.01) | 2.80E-01 | hetero | -0.01 | (-0.04 to 0.02) | 5.73E-01 | hetero | -0.01 | (-0.04 to 0.02) | 5.35E-01 | hetero | -0.02 | (-0.05 to 0.02) | 2.75E-01 |
| rs17047660 | CR1 | 1 | 207782856 | additive | -0.02 | (-0.05 to 0) | 6.37E-02 | recessive | -0.03 | (-0.09 to 0.03) | 3.13E-01 | additive | -0.03 | (-0.05 to 0) | 2.03E-02 | dominant | 0.03 | (-0.01 to 0.07) | 9.53E-02 |
| rs17047661 | CR1 | 1 | 207782889 | dominant | -0.03 | (-0.07 to 0.01) | 1.94E-01 | recessive | -0.01 | (-0.04 to 0.02) | 4.41E-01 | additive | -0.01 | (-0.03 to 0.01) | 1.58E-01 | dominant | 0.02 | (-0.04 to 0.08) | 4.99E-01 |
| rs17561 | IL1A | 2 | 113537223 | recessive | 0.02 | (-0.05 to 0.09) | 5.40E-01 | dominant | -0.01 | (-0.04 to 0.01) | 3.25E-01 | recessive | 0.04 | (-0.03 to 0.1) | 2.39E-01 | hetero | -0.01 | (-0.04 to 0.03) | 6.16E-01 |
| rs1800587 | IL1A | 2 | 113542960 | additive | -0.01 | (-0.03 to 0.01) | 3.41E-01 | dominant | 0.02 | (-0.01 to 0.05) | 1.47E-01 | additive | 0.01 | (-0.01 to 0.03) | 2.89E-01 | dominant | 0.01 | (-0.02 to 0.05) | 4.24E-01 |
| rs1143634 | IL1B | 2 | 113590390 | dominant | 0.03 | (0 to 0.06) | 9.61E-02 | additive | 0.01 | (-0.02 to 0.04) | 4.56E-01 | recessive | 0.05 | (-0.05 to 0.14) | 3.19E-01 | additive | -0.02 | (-0.06 to 0.01) | 2.34E-01 |
| rs708567 | IL17RE | 3 | 9960070 | hetero | 0.02 | (-0.01 to 0.04) | 2.31E-01 | recessive | -0.02 | (-0.05 to 0.01) | 2.29E-01 | hetero | -0.02 | (-0.05 to 0) | 1.11E-01 | recessive | -0.03 | (-0.07 to 0.01) | 1.87E-01 |
| rs352140 | TLR9 | 3 | 52231737 | additive | -0.01 | (-0.03 to 0.01) | 2.20E-01 | hetero | 0.02 | (-0.01 to 0.05) | 1.06E-01 | recessive | -0.01 | (-0.04 to 0.01) | 3.52E-01 | dominant | -0.03 | (-0.08 to 0.02) | 2.64E-01 |
| rs187084 | TLR9 | 3 | 52261031 | hetero | 0.02 | (-0.01 to 0.05) | 1.73E-01 | hetero | 0.01 | (-0.02 to 0.04) | 3.61E-01 | dominant | 0 | (-0.04 to 0.05) | 9.05E-01 | dominant | 0.03 | (-0.02 to 0.09) | 2.08E-01 |
| rs6780995 | IL17RD | 3 | 57138419 | hetero | -0.02 | (-0.05 to 0.01) | 1.16E-01 | recessive | 0.01 | (-0.02 to 0.04) | 5.89E-01 | recessive | 0.01 | (-0.02 to 0.04) | 5.07E-01 | recessive | 0.03 | (-0.01 to 0.06) | 1.42E-01 |
| rs4833095 | TLR1 | 4 | 38799710 | recessive | 0.03 | (-0.06 to 0.11) | 5.26E-01 | dominant | 0.02 | (-0.02 to 0.06) | 2.75E-01 | hetero | 0.01 | (-0.02 to 0.05) | 4.30E-01 | recessive | 0.01 | (-0.07 to 0.08) | 8.86E-01 |
| rs5743611 | TLR1 | 4 | 38800214 | NA | NA | NA | NA | NA | NA | NA | NA | NA | NA | NA | NA | NA | NA | NA | NA |
| rs5743810 | TLR6 | 4 | 38830350 | recessive | -0.35 | (-0.84 to 0.13) | 1.50E-01 | hetero | 0.05 | (-0.02 to 0.12) | 1.74E-01 | dominant | 0.03 | (-0.03 to 0.09) | 3.94E-01 | additive | 0.08 | (-0.02 to 0.18) | 1.26E-01 |
| rs5743809 | TLR6 | 4 | 38830514 | hetero | -0.01 | (-0.05 to 0.03) | 7.78E-01 | additive | -0.01 | (-0.05 to 0.03) | 5.51E-01 | hetero | -0.01 | (-0.05 to 0.03) | 5.08E-01 | hetero | -0.08 | (-0.14 to -0.02) | 1.41E-02 |
| rs4073 | IL8 | 4 | 74606024 | hetero | -0.02 | (-0.05 to 0.02) | 3.27E-01 | additive | 0.01 | (-0.01 to 0.04) | 3.35E-01 | hetero | 0.02 | (-0.01 to 0.05) | 2.54E-01 | hetero | 0.01 | (-0.03 to 0.04) | 6.72E-01 |
| rs6897932 | IL7R | 5 | 35874575 | additive | 0.02 | (-0.02 to 0.05) | 3.54E-01 | additive | 0.02 | (-0.02 to 0.06) | 3.27E-01 | hetero | 0.02 | (-0.01 to 0.06) | 2.24E-01 | recessive | 0.04 | (-0.11 to 0.19) | 5.91E-01 |
| rs3194051 | IL7R | 5 | 35876274 | recessive | 0.01 | (-0.04 to 0.07) | 6.19E-01 | recessive | 0.06 | (0.01 to 0.11) | 2.64E-02 | recessive | -0.01 | (-0.06 to 0.04) | 5.94E-01 | recessive | 0.04 | (-0.02 to 0.1) | 2.07E-01 |
| rs1801033 | C6 | 5 | 41199959 | recessive | -0.03 | (-0.07 to 0) | 7.97E-02 | hetero | 0.04 | (0.01 to 0.07) | 7.97E-03 | recessive | -0.02 | (-0.05 to 0.02) | 3.02E-01 | dominant | -0.03 | (-0.07 to 0) | 8.09E-02 |
| rs2289276 | TSLP | 5 | 110407507 | recessive | 0.11 | (0.03 to 0.19) | 6.24E-03 | dominant | 0.01 | (-0.03 to 0.04) | 7.16E-01 | hetero | 0.01 | (-0.02 to 0.04) | 6.95E-01 | hetero | -0.02 | (-0.05 to 0.02) | 4.27E-01 |
| rs3091336 | IL3 | 5 | 131391749 | dominant | -0.05 | (-0.12 to 0.01) | 9.48E-02 | hetero | -0.02 | (-0.05 to 0.01) | 1.58E-01 | hetero | -0.03 | (-0.06 to 0) | 3.59E-02 | hetero | 0.03 | (-0.01 to 0.07) | 9.69E-02 |
| rs35415145 | IL3 | 5 | 131396406 | hetero | -0.03 | (-0.1 to 0.04) | 4.18E-01 | hetero | -0.06 | (-0.14 to 0.01) | 9.95E-02 | dominant | -0.04 | (-0.11 to 0.02) | 2.12E-01 | additive | 0.02 | (-0.06 to 0.11) | 5.60E-01 |
| rs40401 | IL3 | 5 | 131396478 | dominant | -0.01 | (-0.04 to 0.03) | 6.44E-01 | dominant | 0.03 | (-0.01 to 0.06) | 1.23E-01 | dominant | 0.03 | (-0.01 to 0.06) | 1.19E-01 | hetero | -0.01 | (-0.04 to 0.02) | 5.86E-01 |
| rs13166954 | IL3 | 5 | 131396709 | NA | NA | NA | NA | NA | NA | NA | NA | NA | NA | NA | NA | NA | NA | NA | NA |
| rs31481 | IL3 | 5 | 131397202 | additive | -0.01 | (-0.03 to 0.02) | 5.66E-01 | additive | -0.02 | (-0.05 to 0) | 8.05E-02 | dominant | -0.02 | (-0.04 to 0.01) | 2.49E-01 | additive | -0.01 | (-0.04 to 0.02) | 5.13E-01 |
| rs168681 | IL3 | 5 | 131402450 | hetero | -0.01 | (-0.04 to 0.02) | 4.34E-01 | recessive | 0.07 | (0.02 to 0.12) | 3.61E-03 | additive | 0.03 | (0.01 to 0.05) | 8.42E-03 | recessive | 0.07 | (0.01 to 0.12) | 1.68E-02 |
| rs2069614 | CSF2 | 5 | 131407601 | hetero | -0.03 | (-0.06 to 0) | 2.66E-02 | additive | 0.02 | (0 to 0.04) | 6.43E-02 | additive | 0.02 | (0 to 0.04) | 1.37E-02 | hetero | -0.01 | (-0.05 to 0.02) | 4.61E-01 |
| rs10072253 | CSF2 | 5 | 131409768 | NA | NA | NA | NA | NA | NA | NA | NA | NA | NA | NA | NA | NA | NA | NA | NA |
| rs25882 | CSF2 | 5 | 131411460 | recessive | 0.03 | (-0.01 to 0.08) | 1.54E-01 | hetero | 0.03 | (0 to 0.06) | 4.74E-02 | recessive | 0.03 | (-0.01 to 0.08) | 1.50E-01 | hetero | -0.01 | (-0.05 to 0.02) | 3.91E-01 |
| rs25887 | CSF2 | 5 | 131416061 | dominant | -0.01 | (-0.04 to 0.02) | 5.66E-01 | additive | -0.01 | (-0.03 to 0.01) | 3.01E-01 | recessive | 0 | (-0.03 to 0.04) | 8.02E-01 | additive | -0.01 | (-0.03 to 0.01) | 4.47E-01 |
| rs3805685 | P4HA2 | 5 | 131528153 | recessive | -0.09 | (-0.21 to 0.03) | 1.43E-01 | recessive | -0.13 | (-0.26 to 0) | 5.85E-02 | hetero | 0.02 | (-0.02 to 0.06) | 2.57E-01 | dominant | 0.01 | (-0.04 to 0.06) | 7.62E-01 |
| rs156029 | P4HA2 | 5 | 131532634 | hetero | -0.05 | (-0.07 to -0.02) | 9.68E-04 | hetero | -0.03 | (-0.06 to 0) | 5.64E-02 | dominant | -0.02 | (-0.05 to 0.01) | 1.58E-01 | additive | -0.01 | (-0.03 to 0.01) | 4.16E-01 |
| rs159903 | P4HA2 | 5 | 131540053 | recessive | 0.06 | (-0.01 to 0.13) | 7.13E-02 | recessive | -0.06 | (-0.13 to 0.01) | 1.07E-01 | hetero | -0.01 | (-0.04 to 0.01) | 3.42E-01 | recessive | -0.03 | (-0.11 to 0.04) | 3.82E-01 |
| rs11955347 | P4HA2 | 5 | 131567924 | recessive | -0.01 | (-0.05 to 0.04) | 7.32E-01 | additive | -0.02 | (-0.04 to 0.01) | 1.51E-01 | additive | -0.01 | (-0.03 to 0.01) | 2.21E-01 | recessive | -0.06 | (-0.12 to 0.01) | 1.05E-01 |
| rs3900945 | PDLIM4 | 5 | 131592870 | dominant | -0.01 | (-0.04 to 0.01) | 3.40E-01 | recessive | 0.02 | (-0.02 to 0.06) | 3.97E-01 | dominant | 0.02 | (-0.01 to 0.04) | 1.96E-01 | recessive | -0.03 | (-0.07 to 0.01) | 1.69E-01 |

| | | | | | | | | | | | | | | | | | | | |
|---|---|---|---|---|---|---|---|---|---|---|---|---|---|---|---|---|---|---|---|
| rs10463891 | PDLIM4 | 5 | 131597392 | hetero | -0.01 | (-0.04 to 0.02) | 4.02E-01 | recessive | 0.03 | (-0.01 to 0.07) | 1.82E-01 | additive | 0.01 | (0 to 0.03) | 1.30E-01 | recessive | -0.02 | (-0.07 to 0.02) | 2.49E-01 |
| rs156112 | PDLIM4 | 5 | 131610232 | recessive | 0.09 | (0 to 0.19) | 5.33E-02 | recessive | -0.1 | (-0.19 to 0) | 4.36E-02 | dominant | -0.02 | (-0.05 to 0.01) | 1.64E-01 | recessive | -0.03 | (-0.13 to 0.07) | 5.68E-01 |
| rs455649 | SLC22A4 | 5 | 131649306 | NA | NA | NA | NA | NA | NA | NA | NA | NA | NA | NA | NA | NA | NA | NA | NA |
| rs272893 | SLC22A4 | 5 | 131663062 | recessive | -0.02 | (-0.04 to 0.01) | 2.63E-01 | additive | -0.02 | (-0.04 to 0) | 5.34E-02 | recessive | -0.02 | (-0.05 to 0) | 6.87E-02 | recessive | -0.02 | (-0.05 to 0.02) | 2.85E-01 |
| rs2304081 | SLC22A4 | 5 | 131667548 | hetero | 0.02 | (-0.03 to 0.06) | 5.13E-01 | recessive | 0.05 | (-0.19 to 0.29) | 6.95E-01 | recessive | 0.19 | (-0.05 to 0.43) | 1.21E-01 | additive | -0.02 | (-0.08 to 0.03) | 3.87E-01 |
| rs4646201 | SLC22A4 | 5 | 131671634 | NA | NA | NA | NA | NA | NA | NA | NA | NA | NA | NA | NA | NA | NA | NA | NA |
| rs272867 | SLC22A4 | 5 | 131681057 | recessive | -0.02 | (-0.05 to 0.01) | 2.27E-01 | dominant | -0.03 | (-0.07 to 0.01) | 1.73E-01 | recessive | -0.02 | (-0.05 to 0) | 8.37E-02 | recessive | -0.02 | (-0.05 to 0.02) | 2.92E-01 |
| rs10040427 | SLC22A5 | 5 | 131714106 | recessive | 0.18 | (0.02 to 0.34) | 2.64E-02 | dominant | -0.02 | (-0.06 to 0.02) | 3.35E-01 | hetero | 0.01 | (-0.03 to 0.05) | 6.22E-01 | recessive | -0.07 | (-0.25 to 0.11) | 4.45E-01 |
| rs11568525 | SLC22A5 | 5 | 131729935 | hetero | 0.02 | (-0.03 to 0.08) | 4.02E-01 | additive | -0.04 | (-0.09 to 0.02) | 2.04E-01 | hetero | -0.03 | (-0.08 to 0.02) | 2.67E-01 | additive | -0.03 | (-0.1 to 0.03) | 2.82E-01 |
| rs7704457 | LOC441108 | 5 | 131744790 | recessive | -0.03 | (-0.1 to 0.04) | 4.12E-01 | dominant | 0.03 | (0 to 0.06) | 7.28E-02 | hetero | 0.02 | (0 to 0.05) | 1.05E-01 | dominant | 0.03 | (0 to 0.07) | 4.73E-02 |
| rs6874639 | LOC441108 | 5 | 131778716 | hetero | 0 | (-0.03 to 0.03) | 8.80E-01 | additive | 0.03 | (0.01 to 0.05) | 7.72E-03 | additive | 0.01 | (-0.01 to 0.03) | 1.75E-01 | dominant | 0.03 | (-0.01 to 0.06) | 1.18E-01 |
| rs2522051 | LOC441108 | 5 | 131797578 | dominant | -0.01 | (-0.04 to 0.03) | 7.70E-01 | recessive | -0.06 | (-0.09 to - 0.02) | 6.44E-04 | recessive | -0.01 | (-0.03 to 0.02) | 6.57E-01 | dominant | -0.04 | (-0.08 to 0) | 7.48E-02 |
| rs2706379 | LOC441108 | 5 | 131805735 | dominant | 0.01 | (-0.02 to 0.04) | 5.04E-01 | additive | 0.03 | (0.01 to 0.06) | 3.46E-03 | additive | 0.02 | (0 to 0.04) | 1.08E-01 | recessive | 0.06 | (0.01 to 0.12) | 3.04E-02 |
| rs2706381 | LOC441108 | 5 | 131810619 | additive | 0.01 | (-0.01 to 0.03) | 4.62E-01 | additive | 0.03 | (0.01 to 0.05) | 5.47E-03 | additive | 0.02 | (0 to 0.04) | 6.88E-02 | recessive | 0.06 | (0.01 to 0.12) | 3.02E-02 |
| rs2070729 | IRF1 | 5 | 131819921 | dominant | 0.01 | (-0.02 to 0.04) | 5.46E-01 | hetero | 0.01 | (-0.02 to 0.04) | 5.41E-01 | hetero | 0 | (-0.02 to 0.03) | 7.26E-01 | recessive | 0.03 | (-0.03 to 0.08) | 2.97E-01 |
| rs2070724 | IRF1 | 5 | 131822072 | recessive | 0.02 | (-0.02 to 0.05) | 3.08E-01 | hetero | 0.02 | (-0.01 to 0.05) | 1.86E-01 | hetero | 0.01 | (-0.01 to 0.04) | 3.30E-01 | dominant | 0.03 | (-0.01 to 0.06) | 1.53E-01 |
| rs2070722 | IRF1 | 5 | 131824486 | recessive | 0.03 | (-0.01 to 0.06) | 1.74E-01 | dominant | 0.01 | (-0.02 to 0.05) | 4.37E-01 | hetero | 0.01 | (-0.01 to 0.04) | 3.19E-01 | dominant | 0.03 | (-0.01 to 0.06) | 1.31E-01 |
| rs2706384 | IRF1 | 5 | 131826880 | recessive | 0.02 | (-0.01 to 0.06) | 2.34E-01 | dominant | 0.03 | (0 to 0.06) | 3.10E-02 | hetero | 0.03 | (0 to 0.05) | 4.09E-02 | dominant | 0.05 | (0.02 to 0.09) | 4.75E-03 |
| rs2548999 | IRF1 | 5 | 131831058 | recessive | 0.03 | (-0.01 to 0.06) | 1.67E-01 | hetero | 0.03 | (0.01 to 0.06) | 1.59E-02 | hetero | 0.03 | (0.01 to 0.06) | 1.84E-02 | dominant | 0.04 | (0.01 to 0.08) | 1.31E-02 |
| rs739718 | IL5 | 5 | 131873073 | hetero | -0.02 | (-0.05 to 0.01) | 1.80E-01 | hetero | 0.01 | (-0.02 to 0.04) | 4.34E-01 | recessive | -0.03 | (-0.07 to 0.02) | 2.37E-01 | additive | -0.02 | (-0.04 to 0.01) | 2.61E-01 |
| rs2069818 | IL5 | 5 | 131877524 | recessive | -0.08 | (-0.29 to 0.14) | 4.96E-01 | recessive | 0.17 | (-0.05 to 0.4) | 1.32E-01 | additive | 0.03 | (-0.01 to 0.07) | 1.65E-01 | recessive | -0.06 | (-0.29 to 0.18) | 6.38E-01 |
| rs4526098 | RAD50 | 5 | 131892979 | hetero | 0.01 | (-0.02 to 0.04) | 4.31E-01 | hetero | 0.01 | (-0.02 to 0.05) | 3.82E-01 | dominant | 0.05 | (0 to 0.11) | 7.35E-02 | additive | -0.02 | (-0.05 to 0.01) | 2.23E-01 |
| rs2706348 | RAD50 | 5 | 131905810 | recessive | -0.02 | (-0.06 to 0.02) | 2.43E-01 | additive | -0.01 | (-0.03 to 0.01) | 3.80E-01 | additive | -0.01 | (-0.03 to 0.01) | 2.56E-01 | recessive | -0.05 | (-0.09 to -0.01) | 2.18E-02 |
| rs28903086 | RAD50 | 5 | 131915022 | hetero | -0.62 | (-1.93 to 0.69) | 3.54E-01 | hetero | 0.57 | (-1.43 to 2.57) | 5.77E-01 | hetero | 0.85 | (-0.72 to 2.43) | 2.87E-01 | hetero | -1.2 | (-1.94 to -0.45) | 1.62E-03 |
| rs28903088 | RAD50 | 5 | 131915673 | NA | NA | NA | NA | NA | NA | NA | NA | NA | NA | NA | NA | NA | NA | NA | NA |
| rs10479007 | RAD50 | 5 | 131917726 | hetero | 0.02 | (-0.02 to 0.05) | 3.78E-01 | recessive | -0.07 | (-0.17 to 0.04) | 2.34E-01 | recessive | -0.01 | (-0.1 to 0.09) | 9.18E-01 | additive | -0.01 | (-0.05 to 0.02) | 4.94E-01 |
| rs28903092 | RAD50 | 5 | 131931472 | hetero | -0.32 | (-0.68 to 0.04) | 8.46E-02 | recessive | -0.36 | (-1.15 to 0.43) | 3.76E-01 | recessive | -0.26 | (-1.14 to 0.62) | 5.59E-01 | hetero | 0.1 | (-0.09 to 0.3) | 2.97E-01 |
| rs17772565 | RAD50 | 5 | 131952405 | additive | -0.02 | (-0.05 to 0.01) | 1.81E-01 | recessive | 0.07 | (-0.06 to 0.2) | 3.07E-01 | recessive | 0.05 | (-0.07 to 0.16) | 4.37E-01 | hetero | -0.04 | (-0.08 to 0) | 5.51E-02 |
| rs3798134 | RAD50 | 5 | 131965179 | additive | 0.01 | (-0.01 to 0.03) | 5.91E-01 | dominant | 0.01 | (-0.02 to 0.04) | 5.03E-01 | additive | 0.02 | (0 to 0.04) | 5.96E-02 | additive | 0.02 | (-0.01 to 0.04) | 1.42E-01 |
| rs4621555 | RAD50 | 5 | 131982808 | hetero | 0.02 | (-0.02 to 0.06) | 3.70E-01 | additive | 0.03 | (-0.01 to 0.07) | 1.08E-01 | dominant | 0.09 | (-0.03 to 0.22) | 1.43E-01 | additive | 0.02 | (-0.02 to 0.06) | 3.11E-01 |
| rs1881457 | IL13 | 5 | 131992409 | recessive | 0.03 | (-0.04 to 0.09) | 4.37E-01 | additive | 0.01 | (-0.01 to 0.04) | 3.20E-01 | additive | 0.02 | (-0.01 to 0.04) | 1.40E-01 | additive | 0.04 | (0.01 to 0.07) | 9.25E-03 |
| rs2069744 | IL13 | 5 | 131994669 | dominant | 0.02 | (-0.01 to 0.05) | 1.85E-01 | hetero | -0.01 | (-0.04 to 0.02) | 6.20E-01 | dominant | 0.03 | (0 to 0.06) | 4.76E-02 | hetero | 0 | (-0.03 to 0.04) | 8.77E-01 |
| rs20541 | IL13 | 5 | 131995964 | dominant | 0.02 | (-0.01 to 0.06) | 1.24E-01 | additive | 0.03 | (0 to 0.05) | 5.03E-02 | dominant | 0.02 | (-0.01 to 0.05) | 2.00E-01 | hetero | 0.05 | (0.01 to 0.08) | 1.30E-02 |
| rs848 | IL13 | 5 | 131996500 | recessive | -0.01 | (-0.04 to 0.02) | 5.15E-01 | dominant | 0.02 | (-0.02 to 0.05) | 2.89E-01 | hetero | 0.01 | (-0.01 to 0.04) | 3.11E-01 | dominant | 0.02 | (-0.02 to 0.06) | 2.26E-01 |
| rs2243206 | IL13 | 5 | 132001065 | recessive | -0.06 | (-0.19 to 0.06) | 3.21E-01 | recessive | -0.16 | (-0.28 to - 0.04) | 1.07E-02 | recessive | -0.12 | (-0.23 to - 0.01) | 3.08E-02 | recessive | -0.11 | (-0.23 to 0.01) | 7.32E-02 |
| rs2243248 | IL4 | 5 | 132008644 | hetero | -0.04 | (-0.07 to -0.01) | 1.79E-02 | recessive | 0.04 | (-0.04 to 0.11) | 3.31E-01 | additive | 0.01 | (-0.01 to 0.03) | 4.71E-01 | dominant | 0.04 | (0 to 0.07) | 4.79E-02 |
| rs2243250 | IL4 | 5 | 132009154 | hetero | 0.01 | (-0.02 to 0.04) | 4.33E-01 | dominant | 0.05 | (0 to 0.09) | 5.37E-02 | hetero | 0.02 | (0 to 0.05) | 8.50E-02 | hetero | 0.02 | (-0.02 to 0.05) | 2.81E-01 |
| rs2243251 | IL4 | 5 | 132009787 | hetero | 0.03 | (0 to 0.06) | 5.82E-02 | additive | -0.01 | (-0.04 to 0.02) | 4.94E-01 | recessive | -0.04 | (-0.11 to 0.03) | 3.18E-01 | hetero | 0.03 | (-0.01 to 0.07) | 1.54E-01 |
| rs4986964 | IL4 | 5 | 132009821 | NA | NA | NA | NA | NA | NA | NA | NA | NA | NA | NA | NA | NA | NA | NA | NA |
| rs2243270 | IL4 | 5 | 132014109 | dominant | 0.01 | (-0.02 to 0.04) | 4.49E-01 | recessive | -0.02 | (-0.07 to 0.02) | 3.38E-01 | additive | 0 | (-0.02 to 0.02) | 8.30E-01 | hetero | 0.04 | (0 to 0.07) | 3.24E-02 |
| rs2243283 | IL4 | 5 | 132016593 | recessive | 0.06 | (-0.01 to 0.14) | 9.69E-02 | hetero | 0.04 | (0.01 to 0.07) | 1.67E-02 | recessive | -0.02 | (-0.09 to 0.05) | 5.67E-01 | recessive | -0.04 | (-0.13 to 0.05) | 3.74E-01 |
| rs3212227 | IL12B | 5 | 158742950 | dominant | 0.01 | (-0.02 to 0.04) | 4.03E-01 | additive | 0 | (-0.02 to 0.02) | 7.54E-01 | dominant | 0.02 | (-0.01 to 0.04) | 2.00E-01 | dominant | 0.03 | (0 to 0.06) | 6.92E-02 |
| rs919766 | IL12B | 5 | 158747564 | hetero | -0.02 | (-0.05 to 0.01) | 1.33E-01 | recessive | 0.05 | (-0.01 to 0.12) | 1.27E-01 | additive | -0.01 | (-0.03 to 0.01) | 2.97E-01 | hetero | -0.02 | (-0.05 to 0.02) | 3.34E-01 |
| rs3181216 | IL12B | 5 | 158752978 | additive | -0.02 | (-0.05 to 0) | 9.45E-02 | recessive | -0.05 | (-0.13 to 0.02) | 1.82E-01 | additive | -0.01 | (-0.03 to 0.02) | 6.58E-01 | recessive | -0.03 | (-0.11 to 0.05) | 4.71E-01 |
| rs730691 | IL12B | 5 | 158756227 | hetero | 0.01 | (-0.02 to 0.03) | 7.01E-01 | dominant | 0.02 | (-0.01 to 0.05) | 2.82E-01 | recessive | 0.01 | (-0.02 to 0.05) | 4.67E-01 | dominant | 0.04 | (0.01 to 0.08) | 2.24E-02 |
| rs2239704 | LTA | 6 | 31540141 | hetero | -0.03 | (-0.06 to 0) | 3.36E-02 | recessive | 0.04 | (-0.02 to 0.1) | 1.88E-01 | hetero | -0.02 | (-0.05 to 0.01) | 1.95E-01 | dominant | -0.01 | (-0.04 to 0.03) | 7.68E-01 |
| rs909253 | LTA | 6 | 31540313 | recessive | 0.04 | (0.01 to 0.08) | 7.07E-03 | recessive | 0.02 | (-0.01 to 0.05) | 2.50E-01 | recessive | 0.03 | (0 to 0.06) | 8.64E-02 | recessive | 0.06 | (0.01 to 0.1) | 8.13E-03 |
| rs1799964 | TNF | 6 | 31542308 | additive | -0.01 | (-0.03 to 0.02) | 5.67E-01 | recessive | -0.08 | (-0.13 to - | 1.27E-02 | recessive | -0.02 | (-0.08 to 0.03) | 4.52E-01 | dominant | -0.04 | (-0.07 to -0.01) | 1.83E-02 |

| | | | | | | | | | | | | | | | | | | | |
|---|---|---|---|---|---|---|---|---|---|---|---|---|---|---|---|---|---|---|---|
| | | | | | | | | | | 0.02) | | | | | | | | | |
| rs1800750 | TNF | 6 | 31542963 | dominant | 0.03 | (-0.03 to 0.1) | 3.29E-01 | recessive | -0.07 | (-0.46 to 0.31) | 7.08E-01 | hetero | -0.05 | (-0.11 to 0.01) | 1.13E-01 | hetero | 0.02 | (-0.06 to 0.09) | 6.71E-01 |
| rs1800629 | TNF | 6 | 31543031 | hetero | -0.04 | (-0.08 to 0) | 3.10E-02 | hetero | -0.02 | (-0.05 to 0.02) | 3.68E-01 | recessive | 0.15 | (0.03 to 0.27) | 1.40E-02 | recessive | 0.03 | (-0.11 to 0.16) | 6.98E-01 |
| rs361525 | TNF | 6 | 31543101 | recessive | -0.01 | (-0.15 to 0.14) | 9.29E-01 | hetero | -0.01 | (-0.05 to 0.03) | 5.25E-01 | dominant | -0.02 | (-0.05 to 0.02) | 3.96E-01 | recessive | -0.1 | (-0.31 to 0.1) | 3.10E-01 |
| rs3093662 | TNF | 6 | 31544189 | hetero | -0.02 | (-0.05 to 0.02) | 3.65E-01 | hetero | -0.01 | (-0.05 to 0.02) | 4.45E-01 | dominant | -0.02 | (-0.05 to 0.01) | 2.54E-01 | hetero | 0.01 | (-0.03 to 0.05) | 6.48E-01 |
| rs2242665 | CTL4 | 6 | 31839309 | additive | 0.02 | (0 to 0.04) | 6.93E-02 | dominant | 0.04 | (-0.02 to 0.09) | 1.80E-01 | hetero | -0.02 | (-0.05 to 0.01) | 1.26E-01 | additive | 0.02 | (-0.01 to 0.04) | 1.41E-01 |
| rs1555498 | IL20RA | 6 | 137325847 | dominant | 0.03 | (0 to 0.06) | 8.10E-02 | hetero | 0.02 | (-0.01 to 0.05) | 2.05E-01 | hetero | 0.01 | (-0.01 to 0.04) | 3.34E-01 | recessive | 0 | (-0.05 to 0.04) | 8.99E-01 |
| rs11575936 | IFNGR1 | 6 | 137540425 | NA | NA | NA | NA | NA | NA | NA | NA | NA | NA | NA | NA | NA | NA | NA | NA |
| rs2075820 | NOD1 | 7 | 30492237 | recessive | -0.05 | (-0.09 to -0.01) | 1.73E-02 | recessive | 0.02 | (-0.02 to 0.06) | 3.97E-01 | additive | 0 | (-0.01 to 0.02) | 6.38E-01 | recessive | 0.02 | (-0.03 to 0.06) | 4.92E-01 |
| rs9942705 | GLI3 | 7 | 42445655 | recessive | 0 | (-0.06 to 0.06) | 9.69E-01 | hetero | -0.02 | (-0.05 to 0.01) | 1.78E-01 | hetero | -0.02 | (-0.05 to 0.01) | 1.32E-01 | recessive | 0.08 | (0 to 0.15) | 4.99E-02 |
| rs17172181 | HECW | 7 | 43283115 | recessive | -0.02 | (-0.05 to 0.01) | 1.40E-01 | additive | -0.03 | (-0.05 to -0.01) | 8.53E-03 | additive | -0.02 | (-0.04 to 0) | 6.03E-02 | recessive | -0.02 | (-0.05 to 0.02) | 3.53E-01 |
| rs17172184 | HECW | 7 | 43286129 | recessive | -0.03 | (-0.07 to 0) | 7.78E-02 | additive | -0.02 | (-0.04 to 0) | 4.13E-02 | additive | -0.01 | (-0.03 to 0.01) | 1.88E-01 | recessive | -0.03 | (-0.07 to 0.02) | 2.25E-01 |
| rs7779749 | IZKF1 | 7 | 50418506 | dominant | -0.04 | (-0.08 to 0) | 6.40E-02 | hetero | -0.01 | (-0.04 to 0.02) | 7.10E-01 | hetero | 0.02 | (-0.01 to 0.05) | 1.16E-01 | dominant | 0.02 | (-0.03 to 0.07) | 4.07E-01 |
| rs10230385 | IZKF1 | 7 | 50452552 | dominant | 0.01 | (-0.02 to 0.04) | 4.24E-01 | additive | 0.02 | (0 to 0.04) | 5.64E-02 | dominant | 0.01 | (-0.01 to 0.04) | 3.59E-01 | recessive | -0.01 | (-0.06 to 0.03) | 5.69E-01 |
| rs6964823 | IZKF1 | 7 | 50460096 | hetero | 0.01 | (-0.02 to 0.04) | 3.99E-01 | dominant | -0.03 | (-0.06 to 0) | 5.50E-02 | additive | -0.02 | (-0.04 to 0) | 3.66E-02 | recessive | 0.05 | (0 to 0.09) | 3.44E-02 |
| rs4132601 | IZKF1 | 7 | 50470604 | hetero | 0.02 | (-0.01 to 0.05) | 2.48E-01 | hetero | 0.02 | (-0.01 to 0.05) | 2.35E-01 | dominant | 0.03 | (0.01 to 0.06) | 1.73E-02 | hetero | -0.01 | (-0.05 to 0.02) | 4.08E-01 |
| rs11980407 | IZKF1 | 7 | 50471613 | hetero | 0.02 | (-0.01 to 0.05) | 1.10E-01 | hetero | 0.02 | (-0.01 to 0.05) | 2.34E-01 | recessive | -0.03 | (-0.06 to 0) | 3.30E-02 | hetero | -0.02 | (-0.05 to 0.02) | 3.37E-01 |
| rs6944602 | IZKF1 | 7 | 50473751 | recessive | -0.05 | (-0.19 to 0.1) | 5.34E-01 | hetero | 0 | (-0.04 to 0.05) | 8.61E-01 | hetero | 0.02 | (-0.02 to 0.06) | 2.82E-01 | hetero | -0.04 | (-0.09 to 0.01) | 1.48E-01 |
| rs4947535 | DDC | 7 | 50531681 | hetero | 0.01 | (-0.01 to 0.04) | 3.24E-01 | hetero | -0.01 | (-0.04 to 0.02) | 5.15E-01 | recessive | -0.01 | (-0.04 to 0.03) | 6.68E-01 | dominant | -0.03 | (-0.06 to 0.01) | 1.09E-01 |
| rs11983581 | DDC | 7 | 50532888 | hetero | 0.02 | (-0.02 to 0.05) | 3.04E-01 | dominant | -0.07 | (-0.17 to 0.02) | 1.37E-01 | dominant | -0.04 | (-0.13 to 0.04) | 3.27E-01 | dominant | -0.03 | (-0.15 to 0.09) | 5.95E-01 |
| rs11982772 | DDC | 7 | 50533062 | hetero | 0.02 | (-0.02 to 0.05) | 2.90E-01 | recessive | 0.06 | (-0.03 to 0.16) | 1.95E-01 | recessive | 0.04 | (-0.05 to 0.13) | 3.51E-01 | hetero | -0.01 | (-0.05 to 0.03) | 7.13E-01 |
| rs11575527 | DDC | 7 | 50534327 | hetero | 0.02 | (-0.02 to 0.05) | 3.31E-01 | recessive | 0.07 | (-0.02 to 0.17) | 1.38E-01 | recessive | 0.04 | (-0.04 to 0.13) | 3.28E-01 | hetero | -0.01 | (-0.05 to 0.03) | 7.14E-01 |
| rs11575522 | DDC | 7 | 50535395 | hetero | 0.02 | (-0.02 to 0.05) | 3.59E-01 | recessive | 0.06 | (-0.04 to 0.16) | 2.25E-01 | recessive | 0.04 | (-0.04 to 0.13) | 3.15E-01 | hetero | -0.01 | (-0.05 to 0.03) | 6.26E-01 |
| rs11575518 | DDC | 7 | 50535681 | hetero | 0.02 | (-0.02 to 0.05) | 3.09E-01 | dominant | -0.07 | (-0.17 to 0.03) | 1.51E-01 | dominant | -0.04 | (-0.13 to 0.04) | 3.30E-01 | hetero | -0.01 | (-0.05 to 0.03) | 6.86E-01 |
| rs11575483 | DDC | 7 | 50544663 | recessive | 0.21 | (-0.22 to 0.63) | 3.42E-01 | hetero | -0.05 | (-0.12 to 0.01) | 1.01E-01 | hetero | -0.07 | (-0.13 to -0.01) | 2.56E-02 | hetero | -0.02 | (-0.11 to 0.06) | 5.83E-01 |
| rs11575387 | DDC | 7 | 50567435 | dominant | 0.04 | (0.01 to 0.07) | 9.82E-03 | hetero | -0.03 | (-0.06 to 0) | 5.92E-02 | hetero | -0.01 | (-0.04 to 0.02) | 5.18E-01 | hetero | -0.03 | (-0.07 to 0) | 6.94E-02 |
| rs3779084 | DDC | 7 | 50568735 | dominant | 0.04 | (0.01 to 0.07) | 1.42E-02 | hetero | -0.03 | (-0.06 to 0) | 9.18E-02 | dominant | 0 | (-0.03 to 0.02) | 7.85E-01 | hetero | -0.02 | (-0.05 to 0.02) | 2.96E-01 |
| rs880028 | DDC | 7 | 50570136 | hetero | 0.03 | (0 to 0.06) | 8.35E-02 | hetero | -0.04 | (-0.07 to -0.01) | 2.11E-02 | recessive | 0.01 | (-0.02 to 0.04) | 4.92E-01 | additive | 0 | (-0.02 to 0.03) | 7.56E-01 |
| rs6592961 | DDC | 7 | 50572890 | hetero | 0.02 | (0 to 0.05) | 8.72E-02 | recessive | 0.01 | (-0.02 to 0.04) | 4.57E-01 | hetero | 0.02 | (0 to 0.05) | 1.12E-01 | hetero | 0.03 | (-0.01 to 0.06) | 1.09E-01 |
| rs7809758 | DDC | 7 | 50573333 | hetero | 0.03 | (0 to 0.06) | 2.94E-02 | recessive | 0.01 | (-0.02 to 0.04) | 4.19E-01 | hetero | 0.02 | (-0.01 to 0.04) | 2.17E-01 | hetero | 0.01 | (-0.03 to 0.04) | 6.87E-01 |
| rs1817074 | DDC | 7 | 50574012 | hetero | 0.02 | (-0.01 to 0.05) | 1.75E-01 | additive | 0.01 | (-0.01 to 0.03) | 2.30E-01 | hetero | 0.02 | (-0.01 to 0.04) | 1.66E-01 | hetero | 0.01 | (-0.02 to 0.04) | 4.55E-01 |
| rs10271341 | DDC | 7 | 50591583 | additive | -0.01 | (-0.04 to 0.01) | 3.60E-01 | hetero | 0.01 | (-0.02 to 0.04) | 4.21E-01 | hetero | -0.01 | (-0.04 to 0.02) | 3.82E-01 | additive | -0.02 | (-0.05 to 0.01) | 2.33E-01 |
| rs11575320 | DDC | 7 | 50605298 | additive | -0.01 | (-0.04 to 0.01) | 3.64E-01 | dominant | 0.01 | (-0.02 to 0.04) | 6.62E-01 | dominant | -0.02 | (-0.05 to 0) | 1.00E-01 | dominant | -0.03 | (-0.07 to 0) | 8.58E-02 |
| rs3779074 | DDC | 7 | 50612202 | recessive | -0.02 | (-0.06 to 0.01) | 2.05E-01 | recessive | -0.03 | (-0.06 to 0.01) | 1.56E-01 | recessive | -0.02 | (-0.05 to 0.01) | 2.60E-01 | recessive | -0.05 | (-0.09 to -0.01) | 2.48E-02 |
| rs2044859 | DDC | 7 | 50612562 | recessive | -0.02 | (-0.05 to 0.01) | 2.58E-01 | recessive | -0.02 | (-0.06 to 0.01) | 1.67E-01 | recessive | -0.02 | (-0.05 to 0.02) | 3.35E-01 | recessive | -0.05 | (-0.09 to -0.01) | 2.77E-02 |
| rs2329371 | DDC | 7 | 50615440 | hetero | -0.02 | (-0.05 to 0.01) | 2.78E-01 | recessive | 0.04 | (-0.03 to 0.11) | 2.36E-01 | hetero | -0.01 | (-0.04 to 0.02) | 5.09E-01 | hetero | -0.03 | (-0.06 to 0.01) | 1.42E-01 |
| rs6956737 | DDC | 7 | 50620781 | additive | -0.02 | (-0.05 to 0.01) | 1.18E-01 | hetero | -0.02 | (-0.06 to 0.01) | 2.05E-01 | additive | -0.02 | (-0.04 to 0.01) | 1.57E-01 | recessive | 0.06 | (-0.05 to 0.18) | 2.82E-01 |
| rs1451375 | DDC | 7 | 50622712 | hetero | -0.02 | (-0.05 to 0.01) | 2.70E-01 | additive | 0.02 | (0 to 0.05) | 6.88E-02 | recessive | 0.04 | (-0.02 to 0.11) | 1.97E-01 | hetero | -0.01 | (-0.04 to 0.02) | 5.73E-01 |
| rs10249420 | DDC | 7 | 50623451 | additive | -0.01 | (-0.04 to 0.01) | 3.48E-01 | hetero | 0.03 | (0 to 0.06) | 6.04E-02 | recessive | 0 | (-0.08 to 0.08) | 9.76E-01 | recessive | -0.03 | (-0.12 to 0.06) | 4.85E-01 |
| rs7803788 | DDC | 7 | 50625898 | recessive | -0.03 | (-0.12 to 0.05) | 4.64E-01 | hetero | 0.03 | (0 to 0.06) | 7.07E-02 | dominant | 0.01 | (-0.02 to 0.04) | 5.28E-01 | recessive | -0.05 | (-0.14 to 0.04) | 2.85E-01 |
| rs6593010 | DDC | 7 | 50629888 | hetero | 0.02 | (0 to 0.05) | 7.81E-02 | recessive | -0.04 | (-0.07 to -0.01) | 1.41E-02 | hetero | 0.01 | (-0.02 to 0.04) | 4.36E-01 | dominant | 0.01 | (-0.03 to 0.06) | 5.62E-01 |
| rs7800827 | DDC | 7 | 50673171 | recessive | -0.1 | (-0.24 to 0.04) | 1.69E-01 | recessive | 0.05 | (-0.09 to 0.19) | 4.76E-01 | hetero | 0 | (-0.04 to 0.03) | 8.08E-01 | hetero | 0.01 | (-0.04 to 0.06) | 6.25E-01 |
| rs3211938 | CD36 | 7 | 80300449 | additive | -0.03 | (-0.08 to 0.01) | 1.68E-01 | additive | -0.04 | (-0.08 to 0) | 7.95E-02 | recessive | -0.24 | (-0.35 to -0.12) | 7.70E-05 | hetero | 0.02 | (-0.03 to 0.07) | 4.06E-01 |
| hcd36 | G1439C | 7 | 80302110 | hetero | -0.14 | (-0.38 to 0.09) | 2.38E-01 | hetero | -0.07 | (-0.28 to 0.14) | 5.25E-01 | hetero | -0.07 | (-0.26 to 0.12) | 4.59E-01 | hetero | 0.01 | (-0.21 to 0.24) | 8.97E-01 |
| rs17140229 | CFTR | 7 | 117230283 | recessive | -0.02 | (-0.07 to 0.03) | 3.98E-01 | hetero | 0.03 | (0 to 0.06) | 3.64E-02 | additive | 0.01 | (-0.01 to 0.03) | 4.57E-01 | additive | 0.04 | (0.01 to 0.07) | 2.58E-03 |

| | | | | | | | | | | | | | | | | | | | |
|---|---|---|---|---|---|---|---|---|---|---|---|---|---|---|---|---|---|---|---|
| rs4986790 | TLR4 | 9 | 120475302 | dominant | -0.03 | (-0.06 to 0.01) | 1.92E-01 | hetero | -0.01 | (-0.05 to 0.03) | 5.80E-01 | recessive | -0.06 | (-0.21 to 0.08) | 3.87E-01 | additive | -0.01 | (-0.05 to 0.03) | 6.12E-01 |
| rs4986791 | TLR4 | 9 | 120475602 | recessive | 0.07 | (-0.25 to 0.39) | 6.58E-01 | recessive | 0.23 | (-0.06 to 0.53) | 1.25E-01 | recessive | 0.04 | (-0.26 to 0.33) | 7.98E-01 | hetero | 0.03 | (-0.05 to 0.11) | 4.86E-01 |
| rs8176746 | ABO | 9 | 136131322 | additive | -0.03 | (-0.06 to 0) | 2.41E-02 | hetero | 0.01 | (-0.02 to 0.04) | 6.02E-01 | recessive | -0.09 | (-0.17 to - 0.01) | 3.09E-02 | additive | -0.04 | (-0.07 to -0.01) | 1.26E-02 |
| rs8176719 | ABO | 9 | 136132909 | hetero | -0.01 | (-0.04 to 0.02) | 3.76E-01 | hetero | 0 | (-0.02 to 0.03) | 7.45E-01 | dominant | 0.03 | (-0.02 to 0.07) | 2.26E-01 | dominant | -0.02 | (-0.08 to 0.03) | 3.87E-01 |
| rs334 | HBB | 11 | 5248232 | additive | -0.17 | (-0.24 to -0.11) | 2.94E-07 | hetero | -0.15 | (-0.21 to - 0.09) | 1.26E-06 | dominant | -0.14 | (-0.2 to -0.09) | 6.46E-07 | hetero | -0.04 | (-0.09 to 0.02) | 2.35E-01 |
| rs33930165 | HBB | 11 | 5248233 | additive | -0.02 | (-0.11 to 0.08) | 7.14E-01 | recessive | 0.31 | (0.05 to 0.56) | 1.83E-02 | recessive | 0.19 | (-0.04 to 0.42) | 1.01E-01 | additive | 0.09 | (0 to 0.18) | 5.84E-02 |
| rs7935564 | TRIM5 | 11 | 5718517 | additive | 0 | (-0.02 to 0.02) | 7.91E-01 | additive | -0.01 | (-0.03 to 0.01) | 3.51E-01 | recessive | 0.01 | (-0.02 to 0.04) | 4.87E-01 | hetero | -0.03 | (-0.06 to 0) | 5.87E-02 |
| rs569108 | MS4A2 | 11 | 59863104 | additive | 0.02 | (-0.01 to 0.04) | 1.57E-01 | additive | 0.03 | (0 to 0.05) | 4.50E-02 | hetero | 0.02 | (-0.01 to 0.05) | 1.64E-01 | additive | 0.02 | (-0.01 to 0.05) | 2.55E-01 |
| rs542998 | RTN3 | 11 | 63487386 | recessive | 0.04 | (0 to 0.07) | 5.37E-02 | recessive | 0.06 | (0.03 to 0.1) | 8.82E-04 | recessive | 0.04 | (0 to 0.07) | 3.15E-02 | recessive | 0.04 | (0 to 0.08) | 5.76E-02 |
| rs3024978 | STAT6 | 12 | 57490378 | hetero | -0.53 | (-1.03 to -0.03) | 3.89E-02 | hetero | -0.25 | (-0.67 to 0.18) | 2.53E-01 | hetero | -0.24 | (-0.69 to 0.22) | 3.04E-01 | hetero | -0.16 | (-0.47 to 0.15) | 3.04E-01 |
| rs12314983 | STAT6 | 12 | 57492308 | additive | -0.11 | (-0.26 to 0.03) | 1.18E-01 | dominant | -0.09 | (-0.23 to 0.06) | 2.40E-01 | hetero | -0.09 | (-0.22 to 0.04) | 1.69E-01 | recessive | -0.45 | (-0.97 to 0.07) | 8.84E-02 |
| rs35182390 | STAT6 | 12 | 57493602 | NA | NA | NA | NA | NA | NA | NA | NA | NA | NA | NA | NA | NA | NA | NA | NA |
| rs3024952 | STAT6 | 12 | 57500112 | NA | NA | NA | NA | NA | NA | NA | NA | NA | NA | NA | NA | NA | NA | NA | NA |
| rs2626577 | STAT6 | 12 | 57500509 | NA | NA | NA | NA | NA | NA | NA | NA | NA | NA | NA | NA | NA | NA | NA | NA |
| rs2069727 | IFNG | 12 | 68548223 | additive | -0.01 | (-0.04 to 0.01) | 2.20E-01 | hetero | -0.03 | (-0.06 to 0) | 5.39E-02 | recessive | 0.02 | (-0.04 to 0.07) | 5.56E-01 | hetero | -0.01 | (-0.05 to 0.02) | 4.70E-01 |
| rs2234687 | IFNG | 12 | 68548756 | hetero | 0.06 | (-0.46 to 0.58) | 8.23E-01 | hetero | -0.05 | (-0.51 to 0.4) | 8.16E-01 | hetero | 0.5 | (0.11 to 0.88) | 1.22E-02 | hetero | 0.11 | (-0.2 to 0.41) | 4.85E-01 |
| rs2069718 | IFNG | 12 | 68550162 | dominant | 0 | (-0.02 to 0.03) | 7.50E-01 | recessive | 0.03 | (-0.01 to 0.07) | 1.04E-01 | recessive | 0.02 | (-0.02 to 0.05) | 3.80E-01 | additive | -0.01 | (-0.03 to 0.02) | 6.01E-01 |
| rs1861493 | IFNG | 12 | 68551196 | recessive | 0.05 | (-0.13 to 0.23) | 5.89E-01 | additive | 0.04 | (0 to 0.08) | 3.50E-02 | dominant | 0.01 | (-0.02 to 0.05) | 4.57E-01 | hetero | 0.05 | (0.01 to 0.1) | 2.37E-02 |
| rs2069705 | IFNG | 12 | 68555011 | dominant | 0.03 | (0 to 0.07) | 7.40E-02 | hetero | 0.01 | (-0.02 to 0.04) | 3.91E-01 | additive | 0.01 | (-0.01 to 0.03) | 3.48E-01 | hetero | 0.03 | (0 to 0.06) | 5.95E-02 |
| rs2227507 | IL22 | 12 | 68642647 | recessive | 0.12 | (-0.32 to 0.56) | 5.92E-01 | additive | -0.06 | (-0.12 to 0) | 5.19E-02 | hetero | -0.03 | (-0.08 to 0.03) | 3.38E-01 | hetero | -0.04 | (-0.11 to 0.03) | 2.49E-01 |
| rs1012356 | IL22 | 12 | 68644618 | recessive | -0.02 | (-0.06 to 0.01) | 1.45E-01 | additive | 0.02 | (0 to 0.04) | 1.09E-01 | dominant | 0.03 | (0 to 0.05) | 6.99E-02 | additive | -0.01 | (-0.03 to 0.01) | 3.58E-01 |
| rs2227491 | IL22 | 12 | 68646521 | hetero | 0.01 | (-0.02 to 0.04) | 5.38E-01 | recessive | 0.02 | (-0.01 to 0.05) | 2.18E-01 | dominant | 0.04 | (0 to 0.07) | 2.55E-02 | hetero | -0.01 | (-0.05 to 0.02) | 3.66E-01 |
| rs2227485 | IL22 | 12 | 68647713 | recessive | -0.02 | (-0.05 to 0.02) | 4.06E-01 | hetero | -0.01 | (-0.04 to 0.01) | 3.29E-01 | recessive | 0.02 | (-0.02 to 0.05) | 3.38E-01 | recessive | -0.02 | (-0.06 to 0.02) | 4.03E-01 |
| rs2227478 | IL22 | 12 | 68648622 | recessive | -0.03 | (-0.05 to 0) | 6.78E-02 | additive | 0 | (-0.03 to 0.02) | 6.46E-01 | additive | 0.01 | (-0.01 to 0.03) | 2.17E-01 | hetero | 0.01 | (-0.02 to 0.04) | 5.55E-01 |
| rs229587 | SPTB | 14 | 65263300 | hetero | 0.02 | (-0.01 to 0.05) | 1.62E-01 | hetero | 0.02 | (-0.01 to 0.05) | 1.35E-01 | recessive | -0.03 | (-0.07 to 0.01) | 1.24E-01 | hetero | -0.01 | (-0.04 to 0.03) | 6.82E-01 |
| rs2230739 | ADCY9 | 16 | 4033436 | recessive | 0.08 | (-0.03 to 0.18) | 1.43E-01 | hetero | -0.04 | (-0.07 to 0) | 4.81E-02 | recessive | 0.08 | (-0.01 to 0.17) | 9.92E-02 | additive | -0.03 | (-0.06 to 0) | 8.95E-02 |
| rs10775349 | ADCY9 | 16 | 4079823 | hetero | 0.01 | (-0.02 to 0.03) | 7.28E-01 | hetero | 0.01 | (-0.02 to 0.04) | 6.83E-01 | dominant | -0.01 | (-0.04 to 0.01) | 3.73E-01 | dominant | 0.03 | (-0.01 to 0.06) | 1.66E-01 |
| rs1805015 | IL4R | 16 | 27374180 | hetero | -0.01 | (-0.04 to 0.02) | 4.74E-01 | recessive | -0.01 | (-0.05 to 0.03) | 6.52E-01 | recessive | 0.01 | (-0.02 to 0.05) | 4.21E-01 | dominant | -0.01 | (-0.05 to 0.02) | 4.45E-01 |
| rs5470 | HP | 16 | 72088421 | dominant | -0.03 | (-0.07 to 0.01) | 1.05E-01 | recessive | -0.04 | (-0.17 to 0.08) | 4.81E-01 | hetero | 0.02 | (-0.01 to 0.05) | 2.46E-01 | dominant | -0.03 | (-0.07 to 0.01) | 1.94E-01 |
| rs2535611 | ADORA2B | 17 | 15861332 | dominant | 0.02 | (-0.02 to 0.05) | 3.68E-01 | hetero | 0.02 | (-0.02 to 0.06) | 4.16E-01 | additive | 0.04 | (0.01 to 0.07) | 1.85E-02 | recessive | 0.07 | (-0.11 to 0.26) | 4.54E-01 |
| rs2297518 | NOS2 | 17 | 26096597 | recessive | -0.02 | (-0.1 to 0.06) | 6.74E-01 | hetero | 0.02 | (-0.01 to 0.05) | 1.95E-01 | additive | -0.01 | (-0.04 to 0.01) | 2.54E-01 | recessive | -0.06 | (-0.17 to 0.06) | 3.46E-01 |
| rs1800482 | NOS2 | 17 | 26128509 | recessive | 0.12 | (-0.12 to 0.36) | 3.14E-01 | hetero | 0.04 | (-0.01 to 0.09) | 1.14E-01 | hetero | 0.03 | (-0.01 to 0.08) | 1.42E-01 | recessive | -0.16 | (-0.36 to 0.05) | 1.33E-01 |
| rs2779249 | NOS2A | 17 | 26128581 | additive | 0.01 | (-0.01 to 0.03) | 5.77E-01 | recessive | 0.01 | (-0.03 to 0.05) | 5.06E-01 | recessive | 0.01 | (-0.02 to 0.05) | 4.62E-01 | hetero | 0.03 | (-0.01 to 0.06) | 1.09E-01 |
| rs9282799 | NOS2 | 17 | 26128728 | recessive | -0.11 | (-0.38 to 0.16) | 4.21E-01 | dominant | -0.03 | (-0.08 to 0.02) | 2.84E-01 | additive | -0.03 | (-0.07 to 0.02) | 1.95E-01 | hetero | -0.04 | (-0.1 to 0.03) | 2.51E-01 |
| rs8078340 | NOS2 | 17 | 26129212 | recessive | -0.06 | (-0.13 to 0) | 5.70E-02 | recessive | -0.06 | (-0.12 to 0.01) | 1.05E-01 | recessive | -0.03 | (-0.09 to 0.03) | 3.31E-01 | recessive | -0.07 | (-0.15 to 0.01) | 7.23E-02 |
| rs373533 | EMR1 | 19 | 6919624 | recessive | 0.02 | (-0.01 to 0.06) | 1.57E-01 | recessive | -0.03 | (-0.06 to 0.01) | 1.43E-01 | dominant | 0.01 | (-0.02 to 0.04) | 4.88E-01 | dominant | -0.02 | (-0.05 to 0.02) | 2.78E-01 |
| rs461645 | EMR1 | 19 | 6919753 | dominant | -0.03 | (-0.06 to 0.01) | 1.14E-01 | additive | 0.02 | (0 to 0.04) | 1.02E-01 | additive | 0 | (-0.02 to 0.01) | 6.45E-01 | recessive | 0.01 | (-0.02 to 0.05) | 4.12E-01 |
| rs35825847 | FCER2 | 19 | 7754284 | dominant | -0.5 | (-1.07 to 0.06) | 8.16E-02 | additive | -0.23 | (-0.55 to 0.1) | 1.75E-01 | recessive | -0.49 | (-1.08 to 0.1) | 1.05E-01 | hetero | 0.65 | (-0.1 to 1.4) | 8.75E-02 |
| rs1799969 | ICAM1 | 19 | 10394792 | hetero | 0.02 | (-0.16 to 0.21) | 8.10E-01 | hetero | 0.09 | (-0.07 to 0.26) | 2.67E-01 | hetero | 0.03 | (-0.13 to 0.19) | 7.08E-01 | hetero | 0.03 | (-0.11 to 0.18) | 6.47E-01 |
| rs5498 | ICAM1 | 19 | 10395683 | additive | 0.03 | (0 to 0.06) | 3.88E-02 | recessive | 0.07 | (0 to 0.15) | 5.10E-02 | recessive | 0.03 | (-0.04 to 0.11) | 3.53E-01 | dominant | 0.05 | (0.01 to 0.09) | 9.84E-03 |
| rs8386 | GNAS | 20 | 57485812 | recessive | -0.02 | (-0.1 to 0.07) | 6.86E-01 | dominant | 0.02 | (-0.01 to 0.06) | 1.64E-01 | additive | 0.01 | (-0.02 to 0.04) | 4.57E-01 | recessive | 0.05 | (-0.05 to 0.15) | 3.55E-01 |
| rs1128127 | DERL3 | 22 | 24179132 | hetero | 0.01 | (-0.02 to 0.03) | 5.92E-01 | dominant | 0.01 | (-0.02 to 0.04) | 4.99E-01 | dominant | 0.02 | (-0.01 to 0.05) | 1.63E-01 | additive | 0.02 | (0 to 0.05) | 5.05E-02 |
