# Supplementary material for: Genetic determinants of anti-malarial acquired immunity in a large multi-centre study
Source: Malar J. 2015 Aug 28;14:333. doi: 10.1186/s12936-015-0833-x (PMC4552443; doi:10.1186/s12936-015-0833-x)

ADDITIONAL FILE 18: SUPPLEMENTARY FIGURES

Genetic Determinants Of Anti-Malarial Acquired Immunity In A Large Multi-Centre Study

Jennifer M.G. Shelton, Patrick Corran, Paul Risley, Nilupa Silva, Christina Hubbart, Anna Jeffreys, Kate Rowlands, Rachel Craik, Victoria Cornelius, Meike Hensmann, Sile Molloy, Nuno Sepulveda, Taane G. Clark, Gavin Band, Geraldine M. Clarke, Christopher C.A. Spencer, Angeliki Kerasidou, Susana Campino, Sarah Auburn, Adama Tall, Alioune Badara Ly, Odile Mercereau-Puijalon, Anavaj Sakuntabhai, Abdoulaye Djimde, Boubacar Maiga, Ousmane Toure, Ogobara Doumbo, Amagana Dolo, Marita Troye-Blomberg, Valentina D. Mangano, Frederica Verra, David Modiano, Edith Bougouma, Sodiomon B. Sirima, Muntaser Ibrahim, Ayman Hussain, Nahid Eid, Abier Elzein, Hiba Mohamed, Ahmed Elhassan, Ibrahim Elhassan, Thomas N. Williams, Carolyne Ndila, Alexander Macharia, Kevin Marsh, Alphaxard Manjurano, Hugh Reyburn, Martha Lemnge, Deus Ishengoma, Richard Carter, Nadira Karunaweera, Deepika Fernando, Rajika Dewasurendra, Christopher J. Drakeley, Eleanor M. Riley, Dominic P. Kwiatkowski, and Kirk A. Rockett, in collaboration with the MalariaGEN Consortium,

Corresponding authors Kirk A. Rockett and Dominic P. Kwiatkowski
Wellcome Trust Centre for Human Genetics, University of Oxford, Roosevelt Drive, Oxford, UK

This file contains **Additional Figure SF5: Plot for 178 SNPs with logged anti-malarial antibody levels*.** Values of $-\log_{10}$ p-values plotted against chromosomal positions; only the lowest meta-analysis p-value for each SNP-antibody association is plotted. The red dotted lines indicates a Bonferroni threshold p-value of 6×10^{-5} (equivalent to a Bonferroni correction). Each colour represents a different anti-malarial antibody whilst shape of point represents the genetic model of best fit for the SNP-antibody association: (circle) for additive, (triangle) for dominant, and (square) for heterozygote and (plus) for recessive.
*Adjusted for age, gender, parasite density, village (>20), ethnicity (>20), sample month (>20) and study.

Additional Figure SF5: Plot for 178 SNPs with logged anti-malarial antibody levels*. Values of $-\log_{10}$ p-values plotted against chromosomal positions; only the lowest meta-analysis p-value for each SNP-antibody association is plotted. The red dotted lines indicates a Bonferroni threshold p-value of 6×10^{-5} (equivalent to a Bonferroni correction). Each colour represents a different anti-malarial antibody whilst shape of point represents the genetic model of best fit for the SNP-antibody association: (circle) for additive, (triangle) for dominant, and (square) for heterozygote and (plus) for recessive.

*Adjusted for age, gender, parasite density, village (>20), ethnicity (>20), sample month (>20) and study.

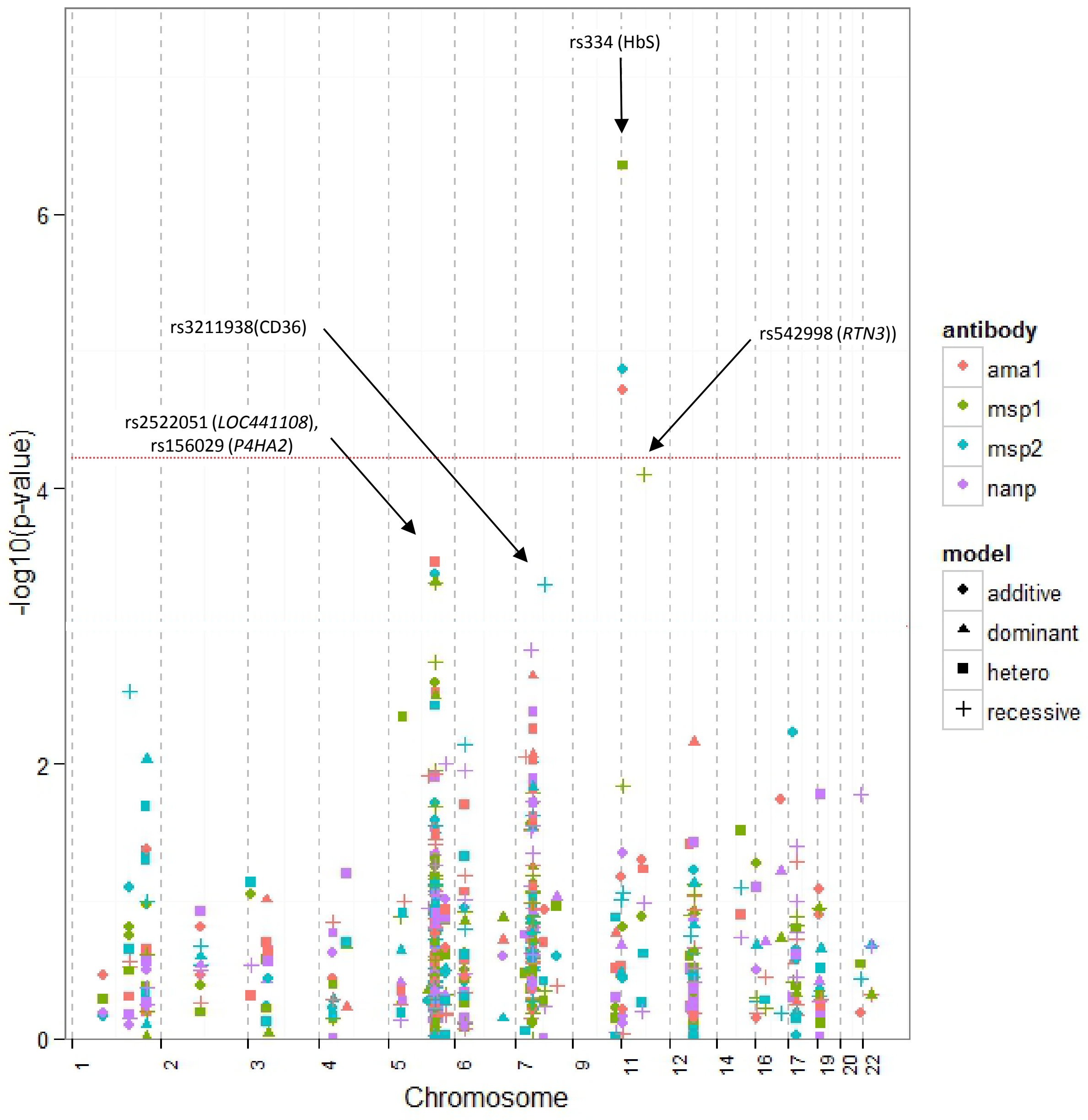

Supplement: Additional file 17: — Additional Figure SF5: Plot for 178 SNPs with logged anti-malarial antibody levels. Plot for the P-values of the analyses of 178 SNPs with logged anti-malarial antibody levels Adjusted for age, gender, parasite density, village (>20), ethnicity (>20), sample month (>20) and study. This is an extension to that shown in the main text and is a reduced dataset as not all sites provided parasitaemia data. [file 12936_2015_833_MOESM17_ESM.pdf]
