# Supplementary material for: Genetic determinants of anti-malarial acquired immunity in a large multi-centre study
Source: Malar J. 2015 Aug 28;14:333. doi: 10.1186/s12936-015-0833-x (PMC4552443; doi:10.1186/s12936-015-0833-x)
Supplement: Additional file 18: — Additional Figure SF6: Forest plot of the association of A: CD36 (rs3211938), B: LOC441108 (rs2522051), C: RTN3 (rs542998) and D: P4HA2 (rs156029) with antibody levels to AMA1, MSP1, MSP2 and NANP. Forest plots of the betas obtained from meta-analysis of results obtained from linear regression models of SNP with logged antibody levels, adjusted for relevant clinical covariates. [file 12936_2015_833_MOESM18_ESM.pdf]

ADDITIONAL FILE 18: SUPPLEMENTARY FIGURES

Genetic Determinants Of Anti-Malarial Acquired Immunity In A Large Multi-Centre Study

Jennifer M.G. Shelton, Patrick Corran, Paul Risley, Nilupa Silva, Christina Hubbart, Anna Jeffreys, Kate Rowlands, Rachel Craik, Victoria Cornelius, Meike Hensmann, Sile Molloy, Nuno Sepulveda, Taane G. Clark, Gavin Band, Geraldine M. Clarke, Christopher C.A. Spencer, Angeliki Kerasidou, Susana Campino, Sarah Auburn, Adama Tall, Alioune Badara Ly, Odile Mercereau-Puijalon, Anavaj Sakuntabhai, Abdoulaye Djimde, Boubacar Maiga, Ousmane Toure, Ogobara Doumbo, Amagana Dolo, Marita Troye-Blomberg, Valentina D. Mangano, Frederica Verra, David Modiano, Edith Bougouma, Sodiomon B. Sirima, Muntaser Ibrahim, Ayman Hussain, Nahid Eid, Abier Elzein, Hiba Mohamed, Ahmed Elhassan, Ibrahim Elhassan, Thomas N. Williams, Carolyne Ndila, Alexander Macharia, Kevin Marsh, Alphaxard Manjurano, Hugh Reyburn, Martha Lemnge, Deus Ishengoma, Richard Carter, Nadira Karunaweera, Deepika Fernando, Rajika Dewasurendra, Christopher J. Drakeley, Eleanor M. Riley, Dominic P. Kwiatkowski, and Kirk A. Rockett, in collaboration with the MalariaGEN Consortium,

Corresponding authors Kirk A. Rockett and Dominic P. Kwiatkowski
Wellcome Trust Centre for Human Genetics, University of Oxford, Roosevelt Drive, Oxford, UK

This file contains **Additional Figure SF6: Forest plot of the association of A: *CD36* (rs3211938), B: *LOC441108* (rs2522051), C: *RTN3* (rs542998) and D: *P4HA2* (rs156029) with antibody levels to AMA1, MSP1, MSP2 and NANP.** Points correspond to betas obtained from meta-analysis of results obtained from linear regression models of SNP with logged antibody levels, adjusted for relevant clinical covariates. Lines represent 95% confidence intervals. Each colour represents a different antibody whilst shape of point represents the antibody type: (circles) for anti-merozoite and (triangles) for anti-sporozoite. Summary meta-analysis p-values obtained from combined data are represented as (squares).

Additional Figure SF6: Forest plot of the association of A: *CD36* (rs3211938), B: *LOC441108* (rs2522051), C: *RTN3* (rs542998) and D: *P4HA2* (rs156029) with antibody levels to AMA1, MSP1, MSP2 and NANP. Points correspond to betas obtained from meta-analysis of results obtained from linear regression models of SNP with logged antibody levels, adjusted for relevant clinical covariates. Lines represent 95% confidence intervals. Each colour represents a different antibody whilst shape of point represents the antibody type: (circles) for anti-merozoite and (triangles) for anti-sporozoite. Summary meta-analysis p-values obtained from combined data are represented as (squares).

A: *CD36* (rs3211938)

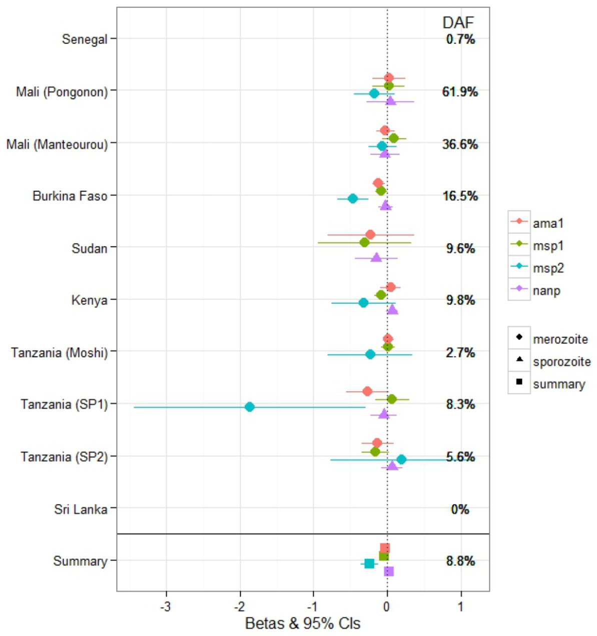

Summary P-values:
ama1 = 0.17 ; msp1 = 0.08 ; msp2 = 7.7 x 10^{-05} ; nanp; = 0.41

B: *LOC441108* (rs2522051)

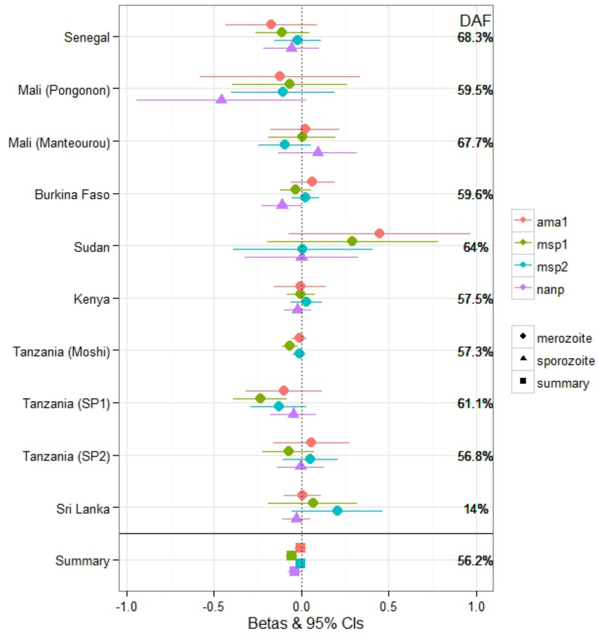

Summary P-values:
ama1 = 0.77 ; msp1 = 6 x 10^{-04} ; msp2 = 0.16 ; nanp; = 0.42

C: *RTN3* (rs542998)

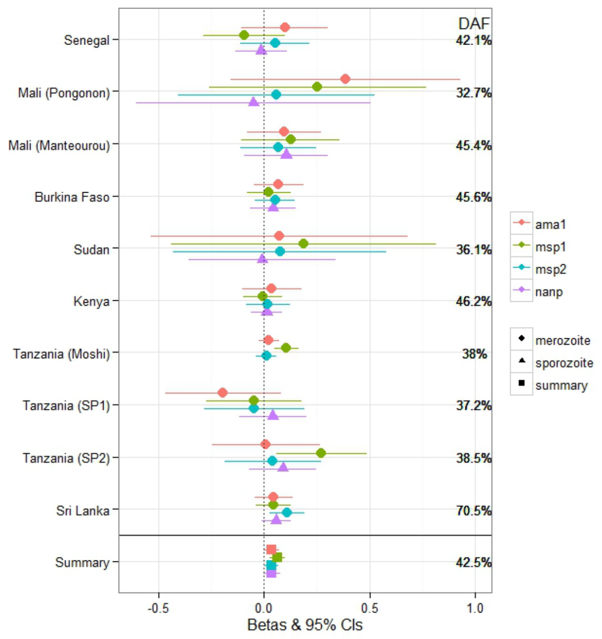

Summary P-values:
ama1 = 0.05 ; msp1 = 8.8x 10^{-04} ; msp2 = 0.03 ; nanp; = 0.06

D: *P4HA2* (rs156029)

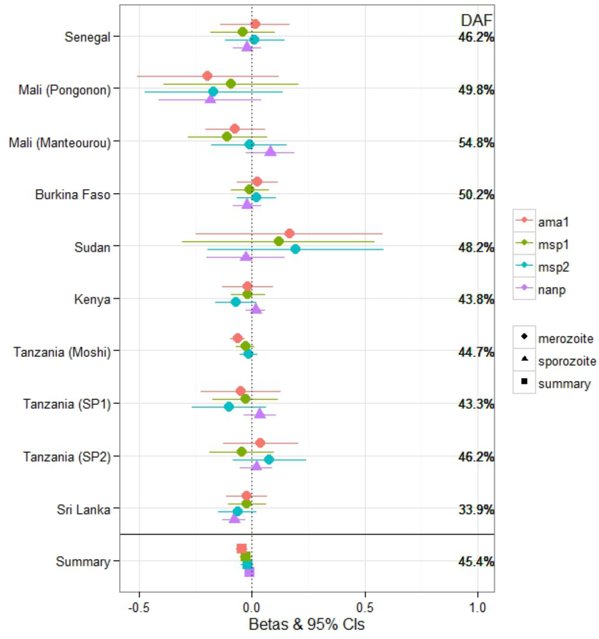

Summary P-values:
ama1 = 9.7 x 10^{-04} ; msp1 = 0.06 ; msp2 = 0.16 ; nanp; = 0.42
